# Evaluating gain and loss-framed mobile messaging to improve maternal health service uptake: A cluster randomized controlled trial in Jimma, Ethiopia

**DOI:** 10.1371/journal.pone.0360474

**Published:** 2026-10-26

**Authors:** Gebeyehu Bulcha Duguma, Hordofa Gutema, Mulusew Gerbaba, Demisew Amenu, Zewdie Birhanu

**Affiliations:** 1 Department of Health, Behaviour, and Society, Faculty of Public Health, Institutes of Health, Jimma University, Jimma, Ethiopia; 2 Oromia Regional State Health Bureau, Jimma Zone Health Office, Department of Maternal, Newborn and Child Health, Jimma, Ethiopia; 3 Data Science and Evaluation, African Population and Health Research Center, Nairobi, Kenya; 4 Department of Obstetrics and Gynecology, Faculty of Medical Sciences, Institutes of Health, Jimma University, Jimma, Ethiopia; Wollega University, ETHIOPIA

## Abstract

**Background:**

Maternal mortality has declined in Ethiopia, yet rural areas still struggle with access to care. Mobile phone-based gain or loss-framed messaging-hold promise for improving maternal health outcomes. Evidence on the comparative impact of these framing strategies in low-resource rural settings remains limited. This study evaluated the effectiveness of gain- versus loss-framed SMS messages in improving maternal health service utilization in Jimma Zone, Ethiopia.

**Methods:**

A three-arm cluster-randomized controlled trial was conducted across 21 clusters, with clusters allocated to gain-framed messaging, loss-framed messaging, or control (usual care). A total of 588 pregnant women (16–20 GA) were recruited in March 2023 and followed from May to December 2023, with endline assessment in February 2024. Participants received 39 messages over a 6–8 month period, aligned with gestational age and postnatal timeline. Primary outcomes were at least one ANC, SBA, and PNC within 7 days. Secondary outcomes included knowledge, attitudes, self-efficacy, and self-reported morbidity. Analyses used generalized and linear mixed models with cluster-level adjustments.

**Result:**

Both framing strategies improved service utilization compared to control. Gain-framed messages increased odds of ANC attendance (OR 5.40, 97.5% CI 1.95–14.94), SBA (OR 7.53, 97.5% CI 2.20–25.79), and PNC (OR 4.83, 97.5% CI 1.71–13.67). Loss-framed messages also improved ANC attendance (OR 7.14, 97.5% CI 2.40–21.26), Skilled Birth Attendance (SBA) (OR 7.31, 97.5% CI 2.13–25.09), and PNC (OR 4.07, 97.5% CI 1.46–11.39). Knowledge improved in both arms, attitudes improved only in the gain-framed arm, and self-efficacy showed no significant change. Self-reported maternal morbidity decreased in both intervention arms.

**Conclusion:**

Both gain- and loss-framed mobile messages led to increased maternal health service utilization. Loss-framed messages were more effective for ANC attendance, knowledge, and morbidity reduction, while gain-framed messages showed greater impact on SBA, PNC, and attitudes.

**Trial registration:**

Pan African Clinical Trials Registry (PACTR), PACTR202201753436676, registered on January 4, 2022; https://tinyurl.com/ykhnpc49

## Background

Maternal health remains a major global public health challenge [1]. According to the World Health Organization, an estimated 287,000 women died from pregnancy-related causes worldwide in 2020, with the majority occurring in low- and middle-income countries. Sub-Saharan Africa accounts for nearly 70% of these deaths, reflecting persistent inequities in access to essential maternal healthcare services [2,3]. In Ethiopia, substantial progress has been made in expanding maternal health services over the past two decades; however, maternal mortality remains high and the country continues to face challenges in meeting the maternal health targets set under the United Nations Sustainable Development Goals [4–7]. Most maternal deaths stem from preventable causes, including infectious diseases and complications during pregnancy, childbirth, and the postpartum period [8].

Despite improvements in the availability of maternal healthcare services, utilization remains suboptimal in many settings. Evidence suggests that behavioral, informational, and motivational barriers continue to influence women’s decisions to seek care during pregnancy, childbirth, and the postnatal period. Addressing these barriers requires not only strengthening health systems but also implementing effective communication strategies that can motivate women to adopt recommended maternal health behaviors [9,10].

Critical components for improving maternal health include strengthening health systems, ensuring skilled birth attendance for every birth, offering facility-based emergency care, and delivering comprehensive antenatal, intrapartum, and postnatal services [11]. Empowering mothers with the knowledge and skills to access appropriate care is a key determinant of maternal health service utilization [12].

Mobile phone–based communication interventions have emerged as promising tools for improving health behaviors in low-resource settings. Short message service (SMS) programs can deliver timely health information and reminders directly to pregnant women, thereby supporting informed decision-making and encouraging service utilization [13]. Recent national survey data show that mobile phone penetration among rural Ethiopian women remains low, with estimates indicating that married men are about five times more likely to own a phone than their wives. This gender gap highlights both opportunities and barriers for SMS-based maternal health interventions [14].

Research has shown that mobile phone messaging has the potential to improve the maternal-infant continuum of care [15], self-management of diabetes, weight loss, physical activity, smoking cessation, and adherence to medication in antiretroviral therapy [16].

However, the effectiveness of health communication depends not only on the content of messages but also on how the information is framed. Message framing refers to presenting the same health information in terms of potential gains (benefits of performing a behavior) or losses (costs of not performing the behavior). This concept is grounded in behavioral decision-making theories such as Prospect Theory, which suggests that individuals respond differently to information depending on whether outcomes are framed as gains or losses [17].

In health communication research, gain-framed messages emphasize the benefits of adopting a recommended behavior, whereas loss-framed messages highlight the negative consequences of failing to take action. These framing strategies may influence individuals’ perceptions of risks and benefits, thereby affecting their motivation to engage in health-related behaviors [18–20].

Although message framing has been widely studied in other health domains, evidence on its effectiveness in promoting maternal healthcare utilization remains limited, particularly in low- and middle-income countries. Most prior studies were conducted in high-income settings, focused on attitudes or intentions rather than actual service use, or relied on non-experimental designs [21].Conversely, another study observed that gain-framed messages promoting healthy parenting practices, such as reducing children’s consumption of sugar-sweetened beverages, were associated with higher parental motivation scores [20]. mHealth interventions like SMS reminders have shown positive impacts on maternal and child health services utilization in Ethiopia [22], few studies have experimentally compared gain-framed versus loss-framed messages. The effectiveness of these framing strategies is context-dependent. Factors such as the type of target behaviour (preventive vs. detection) [23], audience demographics (e.g., rural vs. urban) [24], perceived susceptibility or risk [25], level of involvement [26], past experiences, and the specific health outcome being promoted [18], all influence the relative effectiveness of gain- vs. loss-framing. In the context of maternal health, behaviors such as attending antenatal care visits, delivering in health facilities, and seeking early postnatal care can be considered preventive behaviors because they aim to reduce the risk of maternal and neonatal complications through timely monitoring and skilled care

Furthermore, little evidence exists on the comparative effectiveness of gain-framed versus loss-framed mobile phone messages in improving maternal health service uptake among rural populations. Generating such evidence is particularly important in settings where behavioral barriers contribute to low utilization of maternal health services despite improvements in service availability. Including maternal morbidity as an outcome allows exploration of whether communication interventions influence not only service uptake but also self-reported health experiences.

To address this gap, the present study evaluates the effectiveness of gain-framed and loss-framed SMS messages in promoting maternal healthcare service utilization among pregnant women in rural Ethiopia using a three-arm cluster randomized controlled trial. The primary outcomes include antenatal care attendance, Skilled Birth Attendance (SBA), and early postnatal care utilization. Psychosocial factors such as knowledge, attitudes, and self-efficacy are also examined as potential pathways through which message framing may influence health behaviors.

By integrating behavioral theory with a mobile health intervention, this study aims to provide evidence on whether message framing strategies can enhance the effectiveness of digital communication programs designed to improve maternal health service utilization in low-resource settings.

## Methods and materials

The trial protocol has been published previously [27], and the trial was prospectively registered on January 4, 2022, prior to the enrollment of the first participant and before initiation of the intervention, with Clinical Trials (trial identifier PACTR202201753436676) and can be accessed at https://pactr.samrc.ac.za. The prospective registration occurred more than one year before the baseline survey (March 2023) and well in advance of the intervention period (May–December 2023). The full study protocol approved by the ethics committee is available as supporting information. No deviations from the approved protocol occurred. Minor clarifications presented in this manuscript relate only to additional explanatory detail and do not reflect changes in eligibility criteria, intervention content, outcome definitions, or analytic strategy (S1 Protocol).

### Setting

The study was conducted in three districts of Jimma Zone, Oromia, Ethiopia: Manna, Shebe Sombo, and Dedo. These districts were purposively selected due to no ongoing similar interventions, functional health posts, network coverage, and geographic diversity; limits generalizability to different contexts (. Jimma Zone has – limited health infrastructure, health worker density 3.04/1,000 population according to WHO (World Health Organization) standards 4.45/1000 population [28]. Maternal health indicators in these districts (2023–2024 DHIS2 report) included: early ANC (<12 weeks) 57–68%, syphilis testing 81–99%, four ANC visits 68–89%, and eight ANC visits 14–28% [29].

### Design

A three-arm, parallel-group, cluster-randomized controlled trial (cRCT) with a 1:1:1 allocation ratio was conducted to evaluate the effect of mobile phone messaging-based gain- and loss-framed interventions on improving maternal health service utilization. The baseline survey occurred in March 2023, recruitment and enrollment in March 2023, the intervention was implemented from May to December 2023, and the endline survey was conducted in February 2024. Detailed methods appear in the published protocol [27]. This study adhered to the CONSORT (Consolidated Standards of Reporting Trials) guidelines for clinical trials reporting. These guidelines were followed to ensure transparency and comprehensiveness in both methodology and reporting. The CONSORT checklist for this study is provided as Supporting Information (S2 Checklist). The study was conducted as originally planned, with no modifications to the eligibility criteria, intervention protocols, or data collection procedures. The study followed a prospective cohort design within clusters, where the same participants were followed from baseline to endline. An amendment to the outcome definition was made after protocol registration and documented in the trial records. No replacement sampling was conducted during the follow-up period except in cases of attrition, which were documented in the participant flow diagram.

### Participants

Twenty-one health posts (clusters) were randomly selected from the three districts chosen for the study. Pregnant women with a gestational age (GA) of 16–20 weeks regardless of their current antenatal care status – were eligible if (a) they (or a family member) owned a mobile phone, (b) they could provide written informed consent, and (c) they had lived in the selected Health posts for more than six months. Pregnant women who planned to relocate from the study cluster during the follow-up period were excluded from the intervention. We did not exclude participants unable to read SMS messages themselves, as family members who could read the messages for them were included. Baseline data were collected from primiparous women (who had given birth once to a fetus at or beyond the viable gestational age; this status remains until she completes her second birth) and multiparous women (who had given birth two or more times), ensuring that baseline obstetric characteristics reflected previous childbirth experiences. There were no changes to trial outcomes, eligibility criteria, or study procedures after commencement of recruitment.

### Intervention development

We developed the intervention messages and the messaging system, which automatically dispatched these messages. Participants in the intervention arms received 39 gain-framed or 39 loss-framed messages over 6–8 months at a frequency of 1–2 messages per week. Initially, 120 messages (60 per frame) were drafted in English, each within a 160-character limit, based on Ethiopian Ministry of Health and WHO maternal and newborn health guidelines. After internal review, 32 messages were discarded. The remaining 88 messages were translated into Afan Oromo and evaluated by mothers using card sorting activities; 10 were removed. Eight experts evaluated the remaining 78 messages using 10 evaluation statements. Fourteen messages were revised based on expert feedback. Finally, 39 messages per frame were retained. Messages were delivered two times per week (with occasional single-message weeks depending on gestational scheduling), totaling 39 messages per participant over the intervention period. The intervention was rolled out from May 2023 to December 2023. Participant recruitment occurred from March 1 to March 14, 2023, prior to intervention initiation. Both intervention arms used the same digital platform and delivery schedule, differing only in message framing, allowing isolation of the framing effect.

### Gain-framed message

Automated gain-framed messages were delivered to the women in this group. These messages were personalized based on each woman’s gestational age (GA), expected delivery date (EDD), and postnatal timeline and were sent from the point of ANC enrolment through the end of the postnatal period to the designated phone numbers. Additionally, women in this group received the standard maternal care offered at the health facilities. A summary of message types and frequencies is provided in Supporting Information (S3 Messages).

### Loss-framed messages

Automated loss-framed messages were delivered to the women in this group. These messages were personalized based on each woman’s gestational age, expected delivery date, and postnatal timeline and were sent from the point of ANC enrolment through the end of the postnatal period to the designated phone numbers. Additionally, women in this group received the standard maternal care offered at the health facilities (S3 Messages).

### Control arm

Participants in this arm received routine maternal health services currently practiced in the study districts, which include ANC and PNC counseling delivered through health facilities. Although national guidelines recommend eight ANC visits, actual utilization in the districts remains substantially lower, which justifies evaluating interventions designed to increase service uptake. These visits should include counseling on lifestyle changes, recognizing danger signs and symptoms, birth preparedness, and readiness for complications, as well as other topics like family planning, cord care, bathing, breastfeeding, child immunization, and cervical screening [30]. No additional messaging or contact beyond standard care was provided to the control group.

### Sample size determination

We used methods proposed by Hooper and Bourke for sample size calculation [31], assuming a simple parallel group with a baseline outcome assessment and one follow-up assessment. Although the intervention was implemented simultaneously across all clusters, Hooper and Bourke’s framework was appropriate, as it accounts for both within-cluster correlation at each time point and cluster-period autocorrelation over time, even with only two measurement points. In our repeated measures design, outcome data were collected at baseline and endline from the same individuals within each cluster, requiring adjustment for secular trends and intracluster dependencies across time. Hooper and Bourke’s method enabled us to incorporate these correlation structures into sample size estimation, thereby improving the precision of effect estimates and strengthening the robustness of our cluster-randomized trial design. Three trial arms (gain-framed, loss-framed, and control) were included in the study. The sample size for individual randomization (no) was calculated for comparison of two proportions using the standard formula and assumptions:


$$\begin{array}{c} no=\frac{\left(Z\alpha /\text{2}+\text{Z}\beta /\text{2}\right)\cdot p\text{1}(\text{1}-p\text{1})+p\text{2}(\text{1}-p\text{2})}{(p\text{1}-p\text{2}{)}^{\text{2}}} \end{array}$$

Assumptions:

- Control proportion (baseline), p1 = 0.2327 (23.27%); the proportion of women attending ANC4+among the control arm
- Expected intervention proportion, p2 = 0.3827 (38.27%); absolute difference Δ = p2 − p1 = 0.15
- Two-sided alpha after Bonferroni adjustment for multiple comparisons: α = 0.05; overall the adjusted Zα/2 = 2.24
- Power 1 − β = 0.80 ⇒ Zβ = 0.84

*Thus:*

n0 =(9.4864×0.4149)/ 0.0225=175 per arm under individual randomization.

Because the trial is cluster randomized with repeated (baseline + endline) measures, two design effects were applied to inflate n0, considering the intracluster correlation coefficient (ICC) within each period and the ICC between periods.

(1) Design effect due to clustering within a period (dc): The design effect (dc) due to cluster randomization was calculated by considering the following parameters: Cluster (health-post) sample size m = 28 (women per health post), and within-period intracluster correlation ρ = 0.003 [32].


$$\begin{array}{c} \text{dc}=\text{1}+(\text{m}-\text{1}uprho \end{array}$$

$$\begin{array}{c} \text{dc}=\text{1}+(\text{28}-\text{1})\times \text{0}.\text{003}=\text{1}.\text{081} \end{array}$$

(2) Design effect due to repeated assessments across periods (dr); First we computed the correlation r between cluster means at different times using the Hooper & Bourke formula: r= $\frac{\text{m}\rho \pi }{\text{1}+(\text{m}-\text{1}uprho}$, where π is the cluster autocorrelation (between-period autocorrelation), and here π = 0.8, accounting for a 20% decrease in correlation from within to between different periods [33].

denominator=1+(28−1)×0.003=1.081,


$$\begin{array}{c} \text{r}=\;\frac{\text{28x0}.\text{003x0}.\text{8}}{\text{1}.\text{081}}=\text{0}.\text{0621647} \end{array}$$

The repeated-measures design effect is then


$$\begin{array}{c} \text{dr}=\text{1}-{\text{r}}^{\text{2}}=\text{1}-(\text{0}.\text{0621647}{)}^{\text{2}}=\text{0}.\text{9961356}. \end{array}$$

To obtain the final sample size per arm, we multiplied the sample size for individual randomization with both design effects as:


$$\begin{array}{c} (\text{no}\times \text{dc}\times \text{dr})=\text{174}.\text{88}\times \text{1}.\text{081}\times \text{0}.\text{9961356}=\text{188} \end{array}$$

Considering a 4% loss to follow-up, the final sample size for each arm. n_arm_,final = 188 × 1.04 = 196 participants per arm. This resulted in 28 women per health post for a total sample size of 588.

The assumed effect size was an absolute increase of 15 percentage points in ANC4 + attendance (from 23.27% in the control arm to 38.27% in the intervention arms). This prespecified effect size was used in the sample size calculation and guided the determination of 196 participants per arm.

All parameters used in the sample size calculation (ICC, cluster autocorrelation, expected effect size, and attrition rate) were prespecified in the trial protocol prior to recruitment. No post hoc modifications were made. No formal stopping guidelines were established for this trial. The study was designed to run until the planned end date, and it was completed as scheduled.

### Randomization

#### Random sequence generation.

Random selection was used to choose six primary healthcare units (two from each district), with the health posts (clusters) within these units serving as the randomization units for the trial. Clusters were randomized using simple randomization with a 1:1:1 allocation ratio. From 30 eligible health posts, 21 were randomly selected for the trial. The research team designated the gain frame group as Arm 1, the loss frame group as Arm 2, and the control arm as Arm 3 before the randomization. Randomization was completed before participant recruitment began.

#### Allocation concealment.

During cluster randomization, we listed the 21 clusters alphabetically and numbered them from 1 to 21. Random numbers were generated using Microsoft Excel, and clusters were sorted in ascending order according to these numbers. The first 7 clusters were allocated to Arm 1, the next 7 to Arm 2, and the remaining 7 to Arm 3, ensuring random allocation. Allocation concealment was maintained during participant recruitment. Midwives responsible for recruiting participants were unaware of the cluster allocation. Participants were also not informed about the specific allocation group they belonged to at the time of recruitment. This approach minimized the risk of selection bias, as neither the recruiters nor the participants had any information about the intervention group at recruitment. However, given the nature of the intervention, complete blinding was not feasible during the follow-up period, which could introduce some degree of performance or detection bias [34].

#### Blinding.

A statistician who was unaware of the group assignments randomly assigned clusters to Arm 1, Arm 2, or Arm 3. Within the randomized clusters, midwives, supported by local volunteers, conducted home-to-home screenings to assess pregnant women’s eligibility. Gestational age (GA) was determined using either the last menstrual period (LMP) or, if LMP was unknown, fundal height measurement. After confirming eligibility, a list of eligible pregnant women was prepared for each cluster, and 28 participants were randomly selected for the intervention. Eligible pregnant women were identified through home-to-home screening conducted by trained midwives and community volunteers. Pregnant women were enrolled after providing informed consent, sharing a phone number, and agreeing to receive text messages on their or their family’s phone throughout the intervention period. The interviewers who collected the data were blinded to the intervention assignments; however, blinding the participants was not feasible due to the nature of the intervention as participants could easily identify their arm based on message tone (`gain`vs `loss): however, outcome assessors and data analysts remained blinded to allocation during primary analyses

### Outcomes

#### Primary outcomes.

The primary outcomes were at least one antenatal care, skilled birth attendance, and postnatal service utilization. ANC utilization was defined as at least one contact (ANC1+), aligning with current Ethiopian national guidelines to assess service access rather than maximum coverage (ANC4+), as commonly used in low- and middle-income countries for feasibility. These were measured at an individual level and referred to mothers who had received at least one ANC follow-up visits, delivery at a health facility by skilled professional and attended at least one PNC follow-up visits with health professionals within seven days of delivery. PNC was defined as at least one visit within seven days post-delivery to capture early contact in rural settings, despite WHO’s 48-hour emphasis, with this choice justified and noted as a limitation. Baseline ANC results refer to prior pregnancy attendance.

#### Secondary outcomes.

The secondary outcomes were measured at the individual level and included knowledge about maternal health care, attitude, and self-efficacy toward maternal health care practice, and reported maternal morbidity. Maternal health care knowledge is the extent of understanding about maternal health, including pregnancy, labor, delivery, and postpartum care. It was assessed using a set of 16 questions covering various aspects of pregnancy, labor, delivery, and the postpartum period. Correct responses indicated a stronger understanding, and the total knowledge score was calculated by summing the correct answers, with a higher score reflecting greater overall knowledge. It was assessed using 16 true/false or yes/no items, with each correct response scored as 1 and each incorrect response scored as 0. The total possible knowledge score therefore ranged from 0 to 16, with higher scores indicating greater knowledge.

Attitude toward maternal health care practices is the degree of positive or negative perception toward maternal care utilization. It was measured using 10 items on a 5-point Likert scale, where 1 represented ‘strongly disagree’ and 5 represented ‘strongly agree.’ Negatively worded items were reverse-coded, and the combined scores were analyzed, with higher scores indicating a more positive attitude toward maternal care utilization.

Self-efficacy in maternal care refers to an individual’s confidence in their ability to perform maternal care practices. It was assessed using 10 items on a 5-point Likert scale, where 1 signified ‘strongly disagree’ and 5 signified ‘strongly agree.’ After reverse-coding negatively phrased items, the total score was analyzed, with higher scores reflecting increased self-efficacy in practicing maternal care.

Maternal Morbidity (Illness) refers to the occurrence of maternal health complications during pregnancy, labor, or postpartum which was slf reported. Assessed based on self-reported experiences of illness or complications, including preeclampsia, postpartum hemorrhage, infections (e.g., puerperal sepsis, UTIs), and anemia. The presence or absence of maternal morbidity was recorded, with frequency analysis for comparison. Maternal morbidity as a binary outcome based on self-reported occurrence of complications during pregnancy, childbirth, or the postpartum period, including conditions such as preeclampsia, postpartum hemorrhage, infection, and anemia. These are clinically complex diagnoses and were not confirmed through clinical examination or diagnostic testing. The recall period has been covering the pregnancy-to-postpartum period assessed at endline. Maternal morbidity outcomes were not clinically validated.

Perceived barriers measurement: Perceived barriers were assessed using structured Likert-scale questions on service quality and waiting times.Internal consistency of the main constructs was examined using Cronbach’s alpha [35]., and all constructs were found to have adequate Cronbach`s alpha coefficient values. Cronbach’s alpha coefficient values for knowledge, attitude, and self-efficacy were 0.81, 0.92, and 0.86, respectively (Table 1).

**Table 1:** Measurement scales for secondary outcomes in maternal health care.

Construct	Number of Items	Scale	Example Items	Cronbach’s Alpha
Knowledge	16	Correct/Incorrect	Severe headache is a danger sign during pregnancy	0.81
Attitude	10	5-point Likert (1 = Strongly Disagree, 5 = Strongly Agree)	Antenatal care visits are essential for a healthy pregnancy.“	0.92
Self-Efficacy	10	5-point Likert (1 = Strongly Disagree, 5 = Strongly Agree)	I feel confident in recognizing pregnancy complications.	0.86

### Changes to trial outcomes

Although the published protocol defined maternal health service usage as ANC8+, institutional delivery, and PNC within 7 days, we amended ANC utilization to ANC1+ to reflect feasibility in rural Ethiopia. This amendment was approved by the ethics committee and documented in trial records.

### Adverse event monitoring and reporting

Adverse events, including maternal deaths, were systematically monitored throughout the study per the trial protocol. Serious adverse events were promptly documented and reported to the Institutional Review Board. All maternal deaths were reviewed by an independent district clinical review committee to assess potential links to the intervention. Of the three maternal deaths observed, one occurred intrapartum and two within seven days postpartum (none during the antenatal period prior to delivery); causes were obstetric complications unrelated to SMS exposure.

### Data collection procedure and management

Ten data collectors with relevant health-related college degrees and experience were recruited, along with two public health experts to supervise the data collection. Two days of training were provided to data collectors and supervisors, covering the questionnaire, measurement methods, ethical considerations, and data collection tools. Both data collectors and supervisors were blinded to cluster assignments. Supervisors ensured data completeness during collection, and data collectors corrected any missing items. Baseline data were entered twice to manage entry errors, while endline data were collected using the KoBo Toolbox. Confidentiality was maintained throughout, with personal identifiers removed from the database. Paper questionnaires were securely stored, and electronic data were kept on a password-protected computer accessible only to the research team. A pre-test conducted on 5% of the sample outside the study clusters led to relevant modifications.

Data were collected at two intervals: baseline in March 2023 and endline in February 2024. Both phases utilized interviewer-administered structured questionnaires. Baseline data were collected using a paper-based method, while endline data were collected using the KoBo toolkit. The English version of the questionnaire was translated into Afan Oromo and then back into English by an independent language expert to verify accuracy. The questionnaire included sections on sociodemographic factors, obstetric history, information sources, text message usage, maternal health service utilization, knowledge of maternal health services and danger signs, attitudes toward maternal health services, self-efficacy, and maternal morbidity. The same questionnaire items and structure were retained across both time points. Standardized training, translation and back-translation, and harmonized skip logic were used to improve consistency.

Although the primary outcomes were self-reported, efforts were made to enhance data validity through outcome verification. This was handled by reviewing participants’ ANC cards and, where available, cross-checking the reported number and timing of ANC visits, place of delivery, and PNC attendance with corresponding entries in delivery and postnatal registration books at nearby health facilities. About 82% of the reported outcomes were verified using these records. Approximately 82% of reported service utilization outcomes were verified using facility records. For participants without documentation, self-reported data were retained after confirming internal consistency through probing questions (e.g., number and timing of visits, facility attended, timing of delivery or postnatal check-up). The proportion of verified outcomes was similar across trial arms, minimizing differential misclassification bias. A summary of the data collection methods is presented in Table 2 below.

**Table 2:** Overview of data collection and quality assurance.

Aspect	Details
Personnel	10 data collectors (health-related degrees/experience) + 2 public health expert supervisors
Training	2 days on questionnaire, methods, ethics, tools; blinding to clusters
Data Collection	Baseline (Mar 2023): Paper-based, double-entry
	Endline (Feb 2024): KoBo Toolbox
Quality Control	Supervisors checked completeness; pre-test on 5% sample; 82% outcomes verified via facility records
Validity Checks	ANC cards, delivery/PNC books; probing for unverified self-reports
Data Management	Identifiers removed; secure storage (paper/electronic, password-protected)
Translation	English → Afan Oromo → back-translation by expert

### Data analysis

The data were verified for completeness during data collection, coded, and entered into EpiData 4.6 using the double-data entry method. Endline data were retrieved from KoBo Toolbox. For data analysis, we used Statistical Package for the Social Sciences (SPSS) version 27. Data cleaning was performed by running simple frequency distributions to check for internal consistency. We used frequencies and percentages to summarize descriptive statistics for categorical variables, while means and standard deviations were used for continuous variables.

This study adopted an intention-to-treat (ITT) framework as the primary analysis, consistent with best practices for pragmatic cluster-randomized trials, with per-protocol (PP) analysis as a secondary sensitivity check. Adherence for PP was defined operationally as receipt of ≥80% of scheduled SMS messages, per system delivery logs. Multiple imputation using chained equations (under missing-at-random assumption) was applied to handle missing data while preserving the ITT estimand; it was not restricted to PP participants, ensuring analytical consistency.

To account for the repeated measures design (baseline/endline on the same individuals) and clustering, generalized linear mixed models (GLMM; logit link for binary outcomes) and linear mixed models (LMM; for continuous outcomes) were used, incorporating a random intercept for health post (cluster-level) and a participant-level random effect (or equivalent unstructured covariance) to capture within-individual correlation over time. Fixed effects included time, intervention group, and the time-by-intervention interaction, with baseline covariates (e.g., age, education, parity) and stratification factors included as prespecified; intracluster correlation coefficients (ICCs) were estimated, and robust standard errors were applied due to the small number of clusters (n = 21). No post hoc modifications were made.

A secondary analysis of complete cases was performed as a sensitivity check, and statistical significance was determined with a p-value less than 0.05, adjusted using the sequential Bonferroni method. In addition to the main analysis, descriptive ancillary analysis evaluated changes in perceived barriers to maternal health service utilization and improvements in birth preparedness practices between baseline and endline across study arms. Descriptive statistics, including percentages and frequencies, were used to summarize the findings. All statistical models, including mixed-effects specifications and covariate adjustments for time and intervention-by-time interaction, were prespecified in the protocol. No post hoc analytic changes were introduced.

### Ethical consideration

The Jimma University Institutional Review Board approved the study (ref no- JUIH/IRB/316/23). Ethical approval was obtained prior to participant recruitment and prior to intervention initiation. Participants gave informed consent before the intervention and assessments. Consent details, including study objectives and participant roles, were explained in the local language. Confidentiality was ensured by encrypting phone numbers, accessible only to server technicians and researchers, with data shared after full de-identification. The completed Human Participants Research Checklist form is included as supporting information under the file ‘Other`. To address potential confidentiality risks in this mobile messaging intervention, participants were informed during the consent process about the possibility of shared phone access, particularly in households where phones are not individually owned. Message content was carefully designed to avoid sensitive or stigmatizing information, and participants could opt out of receiving messages at any time without penalty. Data security was ensured through encryption of phone numbers at rest, restricted access via role-based permissions, and de-identification of data prior to analysis.

## Results

### Participant flow

At baseline, 588 participants were surveyed. Sixteen participants were excluded at follow-up: 3 due to maternal deaths (1 in gain-framed, 1 in loss-framed, 1 in control), which occurred postpartum and were not judged related to the intervention according to local health office reports and trial adverse event monitoring, and 13 due to relocation (9 gain-framed, 4 loss-framed). Final analyses included 196 in gain-framed, 192 in loss-framed, and 188 in control arms. Attrition was 2.0%, 4.0%, and 4.1% respectively. The participant flow through the trial is shown in the CONSORT flowchart (Fig 1).

**Fig 1:**
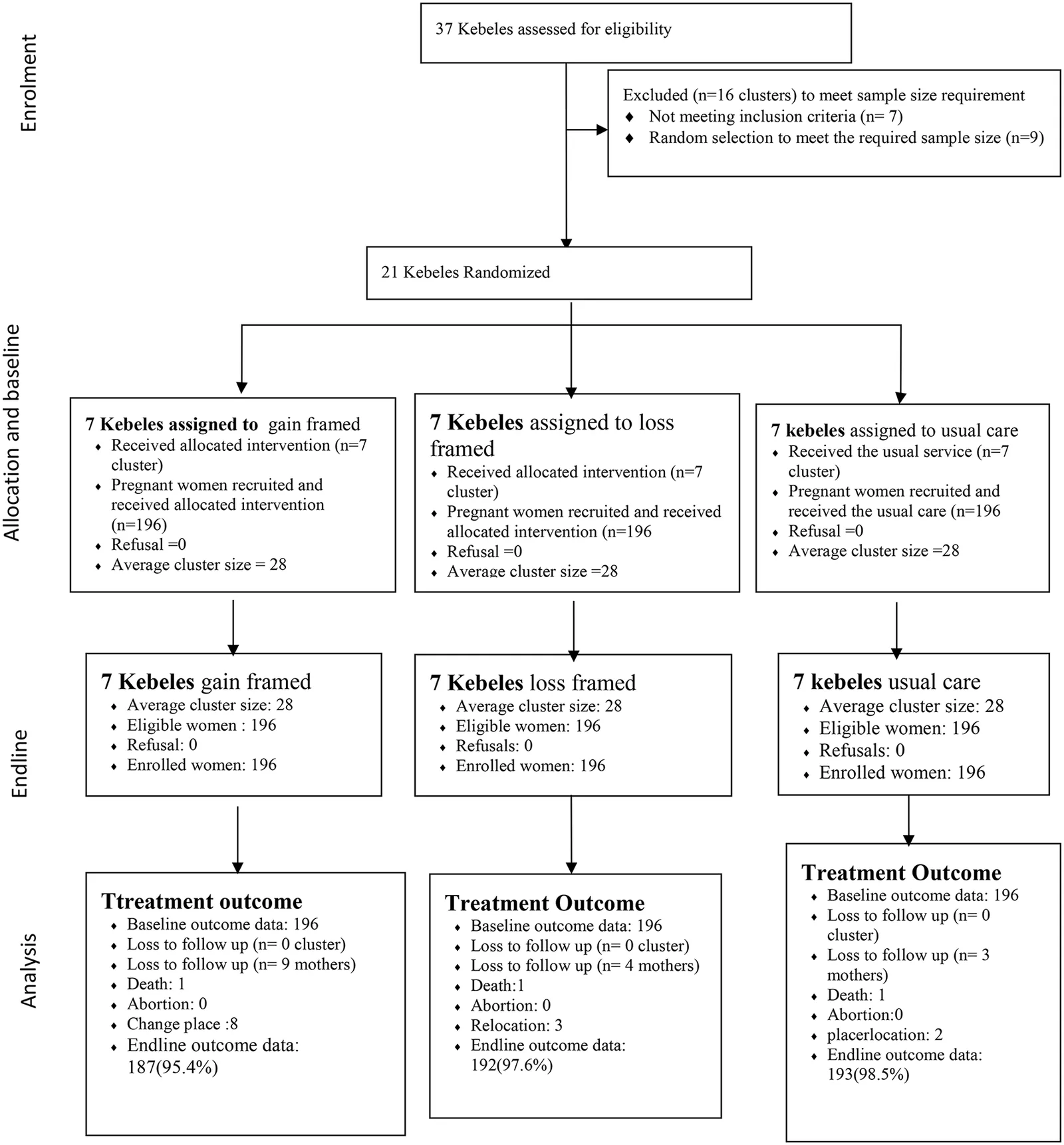
CONSORT flow diagram of clusters and participants in the trial. The diagram shows assessment of 37 kebeles, exclusion of 16, and randomization of 21 kebeles into three arms (gain-framed, loss-framed, and usual care; 7 per arm). All clusters received their allocated interventions. Endline outcome data were available for 187 women (95.4%) in the gain-framed arm, 192 (97.6%) in the loss-framed arm, and 193 (98.5%) in the usual care arm. Reasons for loss to follow-up are indicated in the figure.

### Baseline characteristics of participants

The study included 588 participants, equally allocated to the gain-framed, loss-framed and control arms (n = 196 each). Most participants were aged 20–34 years (90.5%), were Muslim (91.7%), and had completed primary education (51.4%). Almost all were married (94.0%) and housewives (88.8%). Most husbands had primary education (65.50%) and worked as farmers (81.00%).

A majority of participants lived in rural areas (55.10%). Around half (54.08%) of the study participants could read text messages. Most participants (95.10%) were multiparous. The average parity among participants was 3.2 children. There were observable differences among the arms in maternal age, education, marital status, and husband’s occupation (Table 3).

**Table 3:** Baseline characteristics of study participants Jimma Zone, Ethiopia.

Participant characteristics	Gain framed (n = 196)	Loss frame (n = 196)	Control(n = 196)	Overall (n = 588)	Max SMD
**Maternal age in years**					
15–19	8 (4.1)	5 (2.6)	11 (5.6)	24 (4.1)	0.07
20–34	170 (86.7)	183 (93.4)	179 (91.3)	532 (90.5)	0.07
35–44	18 (9.2)	8 (4.1)	6 (3.1)	32 (5.4)	0.12
**Religion**					
Muslim	186 (94.9)	168 (85.7)	185 (94.4)	539 (91.7)	**0.21**
Others^a^	10 (5.1)	28 (14.3)	11 (5.6)	49 (8.3)	**0.21**
**Education status**					
No formal education	45 (23)	49 (25)	53 (27)	147 (25)	0.08
Can read and write	11 (5.6)	17 (8.7)	10 (51)	38 (6.5)	0.05
Primary education	112 (57.1)	92 (46.9)	98 (50)	302 (51.4)	0.11
Secondary education	16 (8.2)	23 (11.7)	22 (11.2)	61 (10.4)	0.06
College and above	12 (6.1)	15 (7.7)	13 (6.6)	40 (6.8)	0.03
**Marital Status**					
Married	196 (100)	169 (86.2)	188 (95.9)	553 (94)	**0.25**
Other^b^	0 (0)	27 (13.8)	8 (4.1)	35 (6)	**0.25**
**Occupation**					
Housewife	186 (94.9)	159 (81.1)	177 (90.3)	522 (88.8)	**0.18**
Merchant	3 (1.5)	16(8.2)	14 (7.1)	33 (5.6)	0.15
Government employee	6 (3.1)	12 (6.1)	0 (0)	18 (3.1)	0.14
Other^c^	1 (0.5)	9 (4.6)	5 (2.6)	15 (2.6)	0.11
**Husband Education status**					
No formal education	13 (6.6)	16 (9.5)	33 (17.6)	62 (11.2)	**0.19**
Can read and write	11 (5.6)	7 (4.1)	15 (8)	33 (6.0)	0.07
Primary education	146 (74.5)	112 (66.3)	104 (55.3)	362 (65.5)	**0.19**
Secondary education	14 (7.1)	18 (10.7)	31 (16.5)	63 (11.4)	0.15
College and above	12 (6.1)	16 (9.5)	10 (2.7)	33 (6.0)	0.13
**Husband Occupation**					
Farmer	170 (86.7)	114 (67.5)	164 (87.2)	448 (81.0)	**0.22**
Merchant	11 (5.6)	30 (17.8)	13 (6.9)	54 (9.8)	**0.19**
Government employee	10 (5.1)	17 (10.1)	3 (1.6)	30 (5.4)	0.15
Other^d^	5 (2.6)	8 (4.7)	8 (4.3)	21 (3.8)	0.06
**Cluster level**					
Rural resident (%)	110 (56)	98 (50)	117 (60)	325 (100)	0.1
Pregnant women per cluster (Mean ± SD)	272 ± 92	255 ± 90.6	240 ± 104.7	256 ± 96.7	0.15
**Family members able to read**					
Yes	79(52.70)	80(49.40)	40(71.40)	199 (54.08)	**0.2**
No	71(47.30)	82(50.60)	16(28.60)	169 (45.92)	**0.2**
**Parity**					
Primiparous	3(1.50)	7(3.60)	19(10.00)	29 (4.90)	**0.22**
Multiparous	193(98.50)	189(96.40)	177(90.00)	559 (95.10)	**0.22**

^a^30 Orthodox, 2 catholic, 17 protestant: ^b^ 33 Single, 1 divorced, 1 separated: ^c^ 11 daily laborer, 4 students: ^d^ 9 daily laborer, 6 merchants, 4 students, 3 non-government organization.

There were notable baseline imbalances across arms in maternal age, education, marital status, and husband’s occupation, which were considered in the adjusted models (see Tables 3–5). Standardized mean differences and covariate-adjusted models were applied to account for these imbalances.

**Table 4:** Effectiveness of interventions on improving maternal health service utilization (ANC, SBA and PNC).

Outcome	Frame	Pre n (%)	Post n (%)	OR (97.5% CI)	P value	ICC
Antenatal care	Gain	*168 (85.71)*	*176 (89.80)*	5.40 [1.95, 14.94]	P < .001	0.47
	Loss	178 (90.82)	184 (93.88)	7.14 [2.40, 21.26]	P < .001	
	Control	168 (85.71)	148 (75.51)	1	—	
Institutional births	Gain	134 (68.37)	168 (85.71)	7.53 (2.20 to 25.79)	P < .001	0.41
	Loss	143 (72.96)	175 (89.29)	7.31 (2.13 to 25.09)	P < .001	
	Control	162 (82.65)	119 (60.71)	1	—	
Postnatal care	Gain	128 (65.31)	146 (74.49)	4.83 (1.71 to 13.67)	P = .001	0.43
	Loss	115 (58.67)	144 (73.47)	4.07 (1.46 to 11.39)	P = .002	
	Control	113 (57.65)	88 (44.90)	1	—	

**Footnote:** OR = Odds Ratio from a generalized linear mixed model (GLMM) comparing each intervention arm (gain-framed or loss-framed) vs the control arm at endline. The model adjusts for the baseline value of the respective outcome, specified covariates and includes a random intercept to account for clustering (intracluster correlation coefficient [ICC] shown). Baseline sample size: total n = 588 (each arm n = 196). Endline sample size: total n = 572 (gain-framed = 187; loss-framed = 192; control = 193). ICC = intraclass correlation coefficient: estimates the proportion of total variance in outcomes attributable to differences between clusters rather than individuals. ICC values ranged from ~0.22 to ~0.48, indicating moderate clustering (i.e., cluster-level factors explain approximately 22%−48% of the variance in antenatal care visits, facility deliveries and postnatal care utilization). Pre measures refer to prior pregnancy history, whereas post measures refer to outcomes in the index pregnancy. Specifically, baseline ANC, SBA, and PNC indicate attendance or use in previous pregnancies, while endline ANC, SBA, and PNC indicate attendance or use during the current study pregnancy.

**Table 5:** Effectiveness of interventions on secondary outcomes (Knowledge about maternal health service, and danger signs during pregnancy, labor, and delivery), Attitude toward maternal health services, Self-efficacy toward maternal health service utilization, and Maternal morbidity).

Outcome	Frame	Pre mean ± SD or n (%)	Post mean ± SD or n (%)	aMD/OR (97.5% CI)	P value	ICC
Knowledge about MHS	Gain	36.13 ± 4.05	41.27 ± 6.89	5.44 (1.66 to 9.22)	P = .001	0.29
	Loss	38.71 ± 3.17	42.45 ± 5.89	7.31 (3.53 to 11.10)	P < .001	
	Control	32.88 ± 5.07	33.67 ± 3.95	0	—	
Attitude toward MHS	Gain	42.15 ± 3.44	45.62 ± 5.28	6.82 (3.52 to 10.12)	P < .001	0.12
	Loss	39.38 ± 5.64	42.21 ± 8.00	3.77 (0.47 to 7.07)	P = .001	
	Control	36.47 ± 4.80	37.62 ± 8.02	0	—	
Self-efficacy toward MHS	Gain	43.46 ± 3.27	45.95 ± 6.60	1.77 (−1.59 to 5.12)	P = .419	0.18
	Loss	42.24 ± 3.85	44.33 ± 5.32	0.33 (−3.02 to 3.68)	P = 1.000	
	Control	42.33 ± 4.25	43.62 ± 6.99	0	—	
Maternal Morbidity	Gain	160 (23.9)	50 (7.5)	OR 4.75 (2.42 to 13.60)	P < .001	0.21
	Loss	132 (19.7)	64 (9.6)	OR 3.83(1.72 to 9.40)	P < .001	
	Control	135 (20.1)	129 (19.3)	1	—	

Footnote: aMD = adjusted mean difference; OR = odds ratio; SD = standard deviation; CI = confidence interval; ICC = intracluster correlation coefficient. Continuous outcomes (knowledge, attitude, self-efficacy) were analysed with a GMM adjusting for baseline value, covariates, and clustering (random intercept). Maternal morbidity (binary) was analysed by GLMM logistic link, similarly adjusted. Baseline n = 588 (196 per arm); endline n = 572 (gain = 187; loss = 192; control = 193). The outcome variable was coded as 1 = no maternal morbidity; therefore, an odds ratio greater than 1 indicates lower odds of maternal morbidity. These results are based on self-reported maternal morbidity and should be interpreted with caution, as they may be influenced by recall or reporting bias.

Baseline ANC values reported reflect any attendance during earlier pregnancies, resolving prior inconsistencies with methods. Multiple primary outcomes (ANC1 + , skilled birth attendance, PNC within 7 days) were pre specified; Bonferroni-adjusted significance was applied across three comparisons, consistent with the sample size calculation and analysis plan.

Baseline characteristics were generally well balanced across the three study arms, with most standardized mean differences below 0.15; modest imbalances were observed in religion, marital status, husband’s education and occupation, family literacy, and parity, but overall the groups were comparable at enrollment.

### Effectiveness of interventions on improving antenatal care (ANC), skilled birth attendance (SBA), and postnatal care (PNC)

“Pre” represents the baseline status of participants at enrollment (16–20 weeks gestation) for any ANC attendance, SBA intention, and PNC uptake readiness, while “Post” represents the measured outcome at the endline survey after intervention completion.

**Antenatal Care (ANC):** Utilization increased in both intervention arms. In the gain-framed arm, ANC rose from 85.71% at baseline to 89.80% at the endline. In the loss-framed armit increased from 90.82% to 93.88%;in the control arm it decreased from 85.71% to 75.51%. The decline in control arm may reflect external factors, such as service disruptions or differences in measurement mode (paper baseline vs KoBo endline), and should be interpreted cautiously. Compared with control, the gain-framed arm had an OR of 5.40 (97.5% CI: 1.95 to 14.94). The loss-framed arm had OR of 7.14 (97.5% CI: 2.40 to 21.26) compared to the control arm (Table 4).

**Skilled Birth Attendance:** Utilization increased significantly in the both intervention arms. The gain-framed arm showed an increase from 68.37% at baseline to 85.71% at endline, and the loss-framed arm from 72.96% to 89.29.0%. In the control arm it decreased from 82.65% to 60.71%. The gain-framed arm had OR of 7.53 (97.5% CI: 2.20 to 25.79) compared to the control arm. The loss-framed arm had OR fo 7.31 (97.5% CI: 2.13 to 25.09) compared to the control arm. Observed ICCs indicate strong cluster effects, reducing effective sample size and widening confidence intervals, limiting precision of effect estimates(Table 4).

**Postnatal Care (PNC):** Utilization improved in both intervention arms. In gain-framed arm PNC increasing from 65.31% at baseline to 74.49% at endlineIn loss-framed arm it increased from 58.67% to 73.47%. Inthe control arm it decreased from 57.65% to 44.90%. The gain-framed arm had OR of 4.83 (97.5% CI: 1.71 to 13.67) compared to the control arm. The O loss-framed arm had OR of4.07 (97.5% CI: 1.46 to 11.39) compared to the control arm (Table 4).

High baseline utilization of ANC and SBA means the absolute increase is modest; large relative ORs are partly due to control-arm decline and clustering. These findings should be interpreted cautiously.

### Intervention effect on secondary outcomes

**Knowledge about Maternal Health Services (MHS):** The intervention significantly increased participants’ knowledge of maternal health services in both the gain-framed and loss-framed groups compared to the control group. At baseline, mean knowledge scores were 36.13 (± 4.05) in the gain-framed group, 38.71 (± 3.17) in the loss-framed group, and 32.88 (± 5.07) in the control group. By endline, scores rose to 41.27 (± 6.89) in the gain-framed group, 42.45 (± 5.89) in the loss-framed group, and modestly to 33.67 (± 3.95) in the control group.

After adjusting for baseline knowledge and potential confounders, both intervention groups showed a statistically significant improvement in knowledge of maternal health services. The adjusted mean difference (aMD) in knowledge was 5.44 (97.5% CI: 1.66 to 9.22, p = .001) in the gain-framed arm and 7.31 (97.5% CI: 3.53 to 11.10) in the loss-framed arm compared to the control group (Table 5).

**Attitude toward MHS:** Participants’ attitudes towards maternal health services improved significantly in both the gain-framed and loss-framed groups. At baseline, attitude scores averaged 42.15 (± 3.44) in the gain-framed group, 39.38 (± 5.64) in the loss-framed group, and 36.47 (± 4.80) in the control group. At endline, scores increased to 45.62 (± 5.28) in the gain-framed group, 42.21 (± 8.00) in the loss-framed group, and 37.62 (± 8.02) in the control group. After adjustment for baseline attitude scores and potential confounders, both intervention arms showed a statistically significant change in attitude towards maternal health services. The adjusted mean difference (aMD) in attitude score was 6.82 (97.5% CI: 3.52 to 10.12, p < 0.001) in the gain-framed arm (Table 5).

**Self-efficacy toward MHS:** The intervention groups showed improvements in self-efficacy for maternal health services, though these were smaller than the changes seen in knowledge and attitude. At baseline, self-efficacy scores averaged 43.46 (± 3.27) in the gain-framed group, 42.24 (± 3.85) in the loss-framed group, and 42.33 (± 4.25) in the control group. At the endline, the scores were 45.95 (±6.60) for the gain-framed arm, 44.33 (±5.32) for the loss-framed arm, and 43.62 (±6.99) for the control arm. After adjusting for baseline self-efficacy scores and potential confounders, no statistically significant self-efficacy improvement was seen in either the gain-framed arm (aMD: 1.77; 97.5% CI: −1.59 to 5.12) or the loss-framed arm (aMD: 0.33; 97.5% CI: −3.02 to 3.68), relative to the control arm (Table 5). Baseline imbalances in knowledge and attitudes were accounted for in adjusted models; self-efficacy changes were limited.

**Maternal Morbidity:** Maternal morbidity declined markedly in the intervention groups. At baseline, maternal morbidity rates were 23.9% (n = 160) in the gain-framed group, 19.7% (n = 132) in the loss-framed group, and 20.1% (n = 135) in the control group. By endline, rates had dropped to 7.5% (n = 50) in the gain-framed arm,9.6% (n = 64) in the loss-framed arm, and 19.3% (n = 129) in the control group. There was a significant reduction in maternal morbidity in the gain-framed arm (OR: 4.75; 97.5% CI, 2.42 to 13.60) and the loss-framed arm (OR: 3.83; 97.5% CI, 1.72 to 9.40) compared to the control arm. These large declines should be interpreted cautiously because morbidity was self-reported and complex to diagnose. ORs were coded with “1 = morbidity present”; misinterpretation is avoided by explicitly reporting coding (Table 5).

### Associations between maternal knowledge, attitudes, self-efficacy, and morbidity with utilization of maternal health services

Women with high self-efficacy were somewhat more likely to attend antenatal care (OR = 1.43; 97.5% CI: 1.00–2.05; p = .055). Reported maternal morbidity also increased the odds of antenatal care attendance (OR = 1.99; 97.5% CI: 1.34–2.97; p = .001). For facility delivery, greater knowledge (OR = 1.94; 97.5% CI: 1.43–2.63; p < .0001), more positive attitudes (OR = 1.58; 97.5% CI: 1.17–2.14; p = .003), and higher self-efficacy (OR = 1.39; 97.5% CI: 1.04–1.86; p = .028) were each independently associated with significantly higher odds of delivering in a facility. Pregnancy or childbirth-related maternal morbidity was also linked with higher odds of facility delivery (OR = 3.14; 97.5% CI: 2.25–4.39; p < .0001). Finally, both self-efficacy (OR = 1.40; 97.5% CI: 1.09–1.80; p = .009) and morbidity (OR = 2.73; 97.5% CI: 2.10–3.55; p < .0001) predicted uptake of postnatal care (Table 6).

**Table 6:** The relationship between secondary outcomes (knowledge, attitude, self-efficacy, and maternal morbidity) and maternal health service practice.

Outcomes	Predictors		OR(97.5 CI)	P.Value
ANC attendance	Self-efficacy	High self-efficacy	1.43 (1.00–2.05)	.055
		Low self-efficacy	1	
	Maternal Morbidity	Yes	1.99 (1.34–2.97)	.001
		No	1	
Institutional childbirth	Knowledge	Knowledgeable	1.94 (1.43–2.63)	<.0001
		Less Knowledgeable	1	
	Attitude	Favorable attitude	1.58 (1.17–2.14)	.003
		Unfavorable attitude	1	
	Self-efficacy	High self-efficacy	1.39 (1.04–1.86)	.028
		Low self-efficacy	1	
	Maternal Morbidity	Yes	3.14 (2.25–4.39)	<.0001
		No	1	
Postnatal care	Self-efficacy	High self-efficacy	1.40 (1.09–1.80)	.009
		Low self-efficacy	1	
	Maternal Morbidity	Yes	2.73 (2.10–3.55)	<.0001
		No	1	

### Missing data and sensitivity analysis

The primary and secondary outcomes each had missing data for 16 (1.4%) Participants. We addressed this using multiple imputation in our main analysis to account for the missingness. A sensitivity analysis using a complete-case approach yielded consistent and statistically significant results, reinforcing the robustness of our findings (Table 7).

**Table 7:** Sensitivity analyses of primary and secondary outcomes for complete cases in a study of effectiveness of mobile phone messaging-based message framing.

Outcome	Frame	Pre n (%)	Post n (%)	OR (97.5% CI)	P value	ICC
Antenatal care	Gain	*168 (85.71)*	*176 (89.80)*	5.54 [2.01, 15.30]	P < .001	0.48
	Loss	178 (90.82)	184 (93.88)	7.16 [2.41, 21.29]	P < .001	
	Control	168 (85.71)	148 (75.51)	1	—	
Institutional births	Gain	134 (68.37)	168 (85.71)	7.61 (2.23 to 25.92)	P < .001	0.41
	Loss	143 (72.96)	175 (89.29)	7.21 (2.11 to 24.61)	P < .001	
	Control	162 (82.65)	119 (60.71)	1	—	
Postnatal care	Gain	128 (65.31)	146 (74.49)	4.95 (1.76 to 13.94)	P = .001	0.43
	Loss	115 (58.67)	144 (73.47)	4.00 (1.43 to 11.16)	P = .002	
	Control	113 (57.65)	88 (44.90)	1	—	
Knowledge about MHS	Gain	36.13 ± 4.05	41.27 ± 6.89	5.31 (1.61 to 9.00)	P = .001	0.24
	Loss	38.71 ± 3.17	42.45 ± 5.89	7.23 (3.53 to 10.92)	P < .001	
	Control	32.88 ± 5.07	33.67 ± 3.95	0	—	
Attitude toward MHS	Gain	42.15 ± 3.44	45.62 ± 5.28	6.71 (3.44 to 9.97)	P = .02	0.10
	Loss	39.38 ± 5.64	42.21 ± 8.00	3.71 (0.45 to 6.98)	P < .001	
	Control	36.47 ± 4.80	37.62 ± 8.02	0	—	
Self-efficacy toward MHS	Gain	43.46 ± 3.27	45.95 ± 6.60	1.68 (−1.60 to 4.97)	P = .45	0.15
	Loss	42.24 ± 3.85	44.33 ± 5.32	0.31 (−2.98 to 3.59)	P = 1.00	
	Control	42.33 ± 4.25	43.62 ± 6.99	0	—	
Maternal Morbidity	Gain	160 (23.9)	50 (7.5)	4.75 (2.16 to 10.46)	P < .001	0.56
	Loss	132 (19.7)	64 (9.6)	3.83(1.75 to 8.39)	P < .001	
	Control	135 (20.1)	129 (19.3)	1	—	

#### Harms or unintended effects.

In the study, participants were monitored for any potential harms or unintended effects from the mobile phone messaging interventions. The gain-framed group reported mild anxiety in 1.5% of participants, while the loss-framed group experienced confusion in 2.0% of participants, with one individual opting out of the messages. No significant adverse events were reported, and the observed effects did not impact the study outcomes.

#### Maternal deaths and clinical outcomes.

Three maternal deaths occurred during the study period, evenly distributed across arms (one each in the gain-framed, loss-framed, and control arms) with no clustering pattern. One death was intrapartum and two occurred within seven days postpartum; none were antenatal. Independent district clinical review confirmed no causal relationship with SMS exposure, attributing all to obstetric complications unrelated to the intervention content or delivery.

#### Adherence reporting.

Adherence was defined as receipt of ≥80% of scheduled SMS messages. In the gain-framed arm, 92% (180/196) met this criterion; in the loss-framed arm, 89% (174/196). Per-protocol analyses yielded results consistent with intention-to-treat findings.

## Discussion

This study assessed the impact of mobile-phone messaging interventions, using both gain and loss-framed approaches, on key outcomes: antenatal care (ANC), skilled birth attendance (SBA), postnatal care (PNC), and maternal health knowledge, attitudes, self-efficacy, and morbidity. The trial was powered to detect a 15 percentage point increase in maternal health service utilization. The observed improvements in ANC, SBA, and PNC utilization exceeded this prespecified effect size, supporting the robustness of our findings. The findings demonstrated that the intervention was associated with improved utilization of maternal health services (specifically ANC, Skilled Birth Attendance (SBA), and PNC) and was associated with positive shifts in knowledge, and attitudes.

This three-arm cluster randomized controlled trial evaluated the effectiveness of gain- and loss-framed mobile phone messaging interventions on maternal health service utilization in rural Jimma Zone, Ethiopia. The findings indicate statistically significant associations between framed messaging interventions and improvements in antenatal care attendance, Skilled Birth Attendance (SBA), and early postnatal care utilization compared with usual care.

Although cluster randomization enhances internal validity, the relatively small number of clusters per arm increases the possibility of baseline imbalance across key determinants of maternal health service utilization. Indeed, minor imbalances in selected psychosocial variables were observed at baseline, alongside high observed intracluster correlation coefficients (ICCs). To mitigate this concern, adjusted mixed-effects regression models were employed, incorporating relevant baseline covariates. Nevertheless, residual confounding cannot be completely excluded, and findings should be interpreted as evidence of association within this specific implementation context rather than definitive proof of large causal effects generalizable across settings. The observed intra-cluster correlation coefficients were higher than those assumed during sample size planning. The planning value was based on prior facility-level studies with low ICCs for single binary outcomes, whereas our community-based trial assessed more complex behavioral outcomes, which likely increased cluster-level variability. Consequently, the ICC was underestimated at the design stage, reducing the effective sample size and contributing to wider confidence intervals.

In terms of specific outcomes, ANC use was higher in both the gain and loss-framed groups compared to the control group, but did not differ significantly between the two intervention groups. Skilled birth attendant was higher in both intervention arms. PNC utilization was also significantly higher in both the gain- and loss-framed groups compared to the control group, indicating the overall association of the intervention with promoting the use of maternal health services. Likewise, a study conducted in the United States found that gain-framed messages were more effective in increasing intentions to use condoms, while loss-framed messages were more effective in reducing intentions o have sex with higher-risk partners [36].

The substantial increases in skilled birth attendant and other outcomes were, during our study period, community-based activities such as monthly conferences for pregnant women and male partner engagement, door-to-door registration of pregnant women by volunteers, and community bylaws enforcing a “zero-tolerance” sanction for home delivery were in operation. In addition, broader health‐system improvements like increased outreach, enhanced facility readiness and normative shifts may also have contributed. The decline in maternal health service utilization in the control arm can be attributed to the presence of health interventions or activities, such as campaigns, at baseline, which significantly increased service utilization. However, this attribution introduces potential internal validity concerns, as time-varying contextual factors—including changes in district-level campaigns and community mobilization activities—may have influenced baseline–endline comparisons. The observed intervention effect may partly reflect the removal of concurrent external support rather than solely the additive effect of SMS messaging. Additionally, if community bylaws or enforcement practices were active during the study period, this raises potential for contamination or differential exposure across clusters. However, in the absence of such interventions at endline, utilization declined because the initial momentum generated by external efforts was no longer sustained. The current study showed that the intervention was associated with improved participants’ knowledge of the MHS maternal health service. Our study also found that attitude scores improved in the gain-framed arms. Similarly, a study by Divdar et al. reported significant improvements in oral health knowledge, attitudes, intentions, self-efficacy, and practices among the intervention group compared to the control group, although no significant difference was observed between gain and loss-framed messages [18]. In another trial assessing the effectiveness of a message-framing intervention on complementary feeding-related behaviours among mothers with infants aged 4–8 months, both gain and loss-framed messages equally increased knowledge, self-efficacy, and adherence to appropriate complementary feeding onset, with greater improvements than in the control group [20]. Similarly, a study examining the impact of message framing on oral health behaviours found that both gain and loss-framed interventions effectively improved students’ knowledge of dental hygiene [36]. Another study, which focused on youth participating in a nutrition program, found that both gain and loss-framed messages improved knowledge about nutrition and physical activity [37].

From a theoretical perspective, the modest differentiation between gain- and loss-framed effects in this trial may reflect several boundary conditions highlighted in framing literature, including the predominance of preventive behaviors (ANC, facility delivery, PNC) rather than pure detection behaviors, and the presence of strong existing norms favoring facility-based care in some communities [37]. In such contexts, both gain and loss frames may primarily function to reinforce intentions rather than create large divergences in behavior, which could explain why effects were broadly similar across arms [38]. The lack of significant change in self-efficacy, despite improvements in knowledge and attitudes, may indicate potential ceiling effects among women who already perceived some capability, or the constraining influence of structural barriers (e.g., distance, costs, gendered decision-making) and cultural norms that limit the translation of cognitive shifts into perceived control and action. Stage-based models of behavior change also suggest that framing may differentially influence individuals at varying stages; women already in preparation or action stages for ANC or facility delivery might respond more to reminders and reinforcement, whereas those in earlier stages may require more intensive support to shift self-efficacy and agency. These considerations underscore that framing effects are likely contingent on behavioral type (preventive versus detection), baseline psychosocial levels, and sociocultural context, and they support the need to tailor framed messages not only to the behavior but also to the recipient’s stage and environment [39].

The intervention was associated with lower self-reported maternal morbidity; however, these outcomes were not clinically validated, while the control arm remained largely unchanged (noting that maternal morbidity was self-reported and not clinically adjudicated, and therefore subject to recall bias, reporting bias, and potential misclassification). In a controlled trial, both message-framing approaches improved health outcomes, but the loss-framed condition showed a greater reduction in complications — suggesting that the urgency implied by loss framing may affect results differently [40]. These results suggest descriptive patterns indicating reductions in reported maternal morbidity associated with both gain- and loss-framed messages, warranting further investigation; alternative explanations include improved symptom recognition, differential interpretation of symptoms, increased care-seeking leading to different labeling of complications, and social desirability bias. The magnitude of change is ambitious for a behavioral SMS intervention without a direct clinical component, and strong causal interpretation is cautioned in the absence of objective verification. These results suggest that both gain and loss-framed messages can significantly reduce maternal morbidity. Taken together, these patterns are consistent with the possibility that framed messaging may support reductions in reported maternal morbidity, but they do not provide definitive evidence of direct clinical impact. The reduction was attributed to increased antenatal care attendance, better adherence to recommended health behaviours, and more frequent skilled birth attendant. The large reductions likely reflect shifts in perception due to improved awareness and expectations rather than systemic service changes.

The study found that higher maternal knowledge, more positive attitudes, greater self-efficacy, and prior maternal morbidity each strongly increased the likelihood of attending antenatal care, delivering in a health facility, and using postnatal services. This finding aligns with several studies from Ethiopia and other low- and middle-income countries. A cross-sectional survey in Arba Minch found that low self-efficacy and low knowledge were strongly correlated with low ANC utilization and increased intervention during delivery [41]. Similarly, in the study by Teklemariam et al., experiencing obstetric complications increased rural Ethiopian women’s knowledge of and attitude toward maternity services, and this led to greater use of maternity waiting homes [42].

Overall the study indicate that both gain and loss-framed message strategies were associated with increased maternal health service utilization, knowledge, attitudes, and reported morbidity reduction, though their effects varied across specific maternal health practices. Descriptive patterns suggest loss-framed messages were particularly effective in increasing ANC visits, maternal knowledge and reported maternal morbidity reduction, while gain-framed messages were suggest to be more effective in promoting skilled birth attendants, PNC utilization and attitude. However, neither framing significantly influenced self-efficacy. Primary analyses compared each intervention arm to the control arm and did not include formal head-to-head superiority testing between gain- and loss-framed groups. Similarly, a meta-analysis of 53 studies found that loss-framed appeals were more persuasive in messages encouraging disease detection behaviours and gain-framed messages are often more effective for promoting behaviours associated with achieving positive health outcomes, such as physical activity or healthy eating In terms of disease prevention, gain-framed messaging highlights the promise of a safer, more certain outcome, making it more effective than loss-framed messaging [43].

### Implications

Our study adds valuable insights to the literature on message framing, particularly within the context of maternal health services in Ethiopia. We found that individualized messages delivered via mobile phones were associated with improvements in maternal health service utilization, knowledge, and attitudes among pregnant women, though effects on self-efficacy were less clear.

Higher maternal knowledge, more positive attitudes, and prior maternal morbidity were associated with greater uptake of ANC, facility delivery, and PNC. This suggests that educational and empowerment components remain critical for interventions aiming to increase maternal health service utilization. The differential trends observed between gain- and loss-framed messages highlight the potential for tailoring message framing to the stage of care, the mindset of the target population, and the local service environment, rather than relying on framing alone.

Based on these findings, program managers and policymakers may consider piloting tailored mobile messaging as a complementary strategy within broader maternal health programs, while acknowledging contextual limitations. The study districts were purposively selected, mobile network coverage was adequate, and concurrent community mobilization and bylaws were in operation. Therefore, the effectiveness of similar interventions in more resource-constrained, less mobilized, or differently organized settings remains uncertain.

The study also indicate that effectiveness is not simply a matter of “gain vs loss” messaging, but rather hinges on how the framing aligns with the specific stage of care, the target population’s mindset, and the broader service context. Sustaining improvements may require ongoing reinforcement rather than one-time or short-term campaigns, emphasizing the importance of long-term engagement strategies and integration with other health system activities. Future research should replicate these findings using larger numbers of clusters, intention-to-treat analyses, and in diverse settings to better understand the conditions under which message framing is most effective and to guide context-specific policy recommendations.

In summary, while tailored mobile messaging shows promise as a supportive tool for maternal health programs, its adoption into policy should be conditional, context-sensitive, and complemented by robust evaluation and reinforcement strategies.

### Strengths and limitations

#### Strengthen.

This study’s strengths include the use of a cluster randomized controlled trial (RCT) design, which minimized the risk of information contamination and the employment of allocation concealment during cluster enrollment. The automated messaging system ensured the timely delivery of interventions at a minimal cost. Data were validated through a dual approach: firstly, by comparing collected data with facility records during the data collection phase; and secondly, through a meticulous re-analysis of the raw data against facility records. Approximately 82% of the reported outcomes were cross-checked against participants’ cards. For the remaining participants without documentation, self-reported data were retained after confirming internal consistency through probing questions regarding the number and timing of visits, the facility attended, and the timing of delivery or postnatal check-ups. This rigorous verification process ensured the accuracy and practical applicability of the results.

To ensure analytical precision, baseline differences were addressed through adjustment for baseline use in all primary analyses. Specifically, adjusted mixed-effects models included key prognostic baseline covariates such as maternal age, parity, education, previous service use, and selected psychosocial measures (knowledge, attitudes, and self-efficacy), which were prespecified based on theory and prior evidence. In most cases, covariate adjustment led to similar conclusions as unadjusted analyses, with modest changes in effect size estimates and narrower confidence intervals, suggesting improved precision rather than a reversal of findings. This approach aligns with FDA and EMA guidance that adjustment for important prognostic baseline covariates can enhance efficiency and stabilize chance imbalances without invalidating inference. At the same time, we acknowledge that in a trial with a relatively small number of clusters per arm, statistical adjustment cannot fully resolve baseline imbalances or eliminate residual confounding, and findings should therefore be interpreted with appropriate caution [44].

Additionally, although the study had a relatively small number of clusters, which can limit generalizability, the design carefully accounted for clustering in the analysis. High observed intra-cluster correlation coefficients (ICCs) relative to initial assumptions are acknowledged as a factor that may have reduced the precision of effect estimates and should be considered when interpreting the findings.

Overall, the combination of rigorous data verification, allocation concealment, automated intervention delivery, and covariate-adjusted analysis supports the reliability of the study findings while transparently acknowledging methodological challenges inherent in small-cluster trials.

#### Limitations.

However, the study was limited by its inability to generalize findings to individuals without mobile phones, potential recall bias, and the lack of a monitoring interface for the automated system. Besides, a shift in data collection tools occurred during the study. Although the two tools used the same question structure and data officers received rigorous training to have harmonized procedures, this type of change may have caused information bias. In addition, the relatively small number of clusters per arm and the high intracluster correlation coefficients reduce statistical power and precision, increasing the possibility that some observed associations are unstable. Baseline imbalances in psychosocial predictors despite randomization, together with potential contamination and differential exposure arising from concurrent community mobilization activities and bylaws, may also have influenced the observed effects.

While ANC1 + prioritizes access per Ethiopian guidelines, it may underestimate quality care compared to ANC4 + benchmarks. The 7-day PNC window, though practical for rural under-recording, deviates from WHO’s 48-hour ideal, potentially affecting comparability; future studies should explore voice-based or facility-verified metrics for inclusivity.

The study relied on self-reported maternal morbidity and self-reported service utilization, which are subject to recall error, misclassification, and social desirability bias, particularly in a context where participants and data collectors could not be blinded to allocation. These factors may have led women to over report desirable behaviors or underreport barriers and complications. Future studies should consider evaluating the implementation outcomes and cost-effectiveness of such interventions using larger numbers of clusters, objective or clinically verified outcome measures where feasible, and designs that more fully account for contextual changes over time and minimize contamination.

The switch from paper-based baseline data collection to electronic endline surveys may have introduced mode effects, although efforts were made to minimize this through standardized procedures.

## Conclusion

The study indicates that mobile phone messaging-based gain- and loss-framed approaches were associated with improvements in maternal health service utilization in Jimma Zone, Ethiopia within the constraints of a three-arm cluster randomized design with a relatively small number of clusters, some baseline imbalances, high intracluster correlation, and reliance on self-reported outcomes. Both gain-framed and loss-framed interventions led to substantial increases in antenatal care (ANC), institutional births, and postnatal care (PNC). While both gain- and loss-framed messaging was associated with improved maternal health service utilization, the observed differences between gain- and loss-framed arms should be interpreted as descriptive patterns rather than statistically established superiority, as primary analyses compared each intervention arm to the control arm rather than formally testing head-to-head differences.

Loss-framed messages appeared to be associated with greater increase in antenatal care use, knowledge, and reductions in self-reported maternal morbidity, whereas gain-framed messages appeared more closely associated with higher skilled birth attendants, postnatal utilization and more positive attitudes. Neither framing strategy significantly affected self-efficacy. Both interventions were associated with substantial improvements in maternal knowledge of maternal health services, while attitudes improved more notably in the gain-framed arm. Maternal morbidity rates in terms of self-reported complications decreased in the intervention arms, while the control arm remained relatively unchanged; however, these morbidity outcomes were based on self-report rather than clinical adjudication and should therefore be interpreted cautiously, as they may reflect changes in reporting, symptom recognition, or recall rather than definitive clinical reductions.

Taken together, these findings suggest that mobile phone-based gain- and loss-framed messaging interventions have the potential to support improved maternal health service utilization and reduced self-reported maternal morbidity in this rural Ethiopian setting, but they do not conclusively establish large causal effects or guarantee generalizability to other contexts. The trial was implemented in purposively selected districts with adequate mobile network coverage and concurrent community mobilization activities, and generalization beyond similar health system and sociocultural conditions—particularly to areas with weaker infrastructure or different community engagement—is therefore limited and warrants cautious interpretation. Further research in diverse settings, with larger numbers of clusters and more objective outcome measures where feasible, is needed to confirm effectiveness and scalability.

From a theoretical perspective, the study contributes to framing theory by suggesting that gain- and loss-framed messages may operate differently across specific maternal health behaviors (ANC, facility delivery, PNC) and psychosocial domains (knowledge, attitudes, self-efficacy), underscoring the importance of aligning message framing with behavioral stage, perceived risk, and contextual factors. These insights highlight the value of tailoring not only the content but also the framing of mobile health messages to the targeted behavior and audience context, complementing the programmatic implications for digital maternal health interventions.

## Supporting information

S1 Protocol Study protocol. Trial protocol, published on July 23, 2024, in JMIR Research Protocols, describes the procedure of a three-arm, cluster-randomized controlled trial conducted in rural Jimma Zone, Ethiopia, to evaluate the effectiveness of gain- and loss-framed mobile messages to improve maternal health and newborn care service utilization.
(DOCX)

S2 Checklist CONSORT 2010 checklist. S2 document is the last CONSORT 2010 checklist, modified to a three-arm cluster randomised trial comparing gain‑ and loss‑framed mobile phone messaging to increase utilization of maternal health services in rural Jimma Zone, Ethiopia.
(DOCX)

S3 Messages Types and frequencies of messages. This consisted of 78 specially tailored SMS messages—39 gain-framed and 39 loss-framed—on ten high-priority maternal and newborn health issues (e.g., antenatal care, nutrition, danger signs, birth preparedness, breastfeeding, immunization, and cord care). Messages were framed according to the gestational age and postpartum status of each woman. They were sent automatically weekly, beginning at study recruitment (16–20 weeks gestation) and extending through the postnatal period, to provide routine and timely interaction.
(DOCX)

## Abbreviations

ANC: Antenatal Care
CEmONC: Comprehensive emergency obstetric and newborn care
CI: Confidence interval
CONSORT: Consolidated Standards of Reporting Trials
cRCT: Cluster randomized controlled trial
DE: Design Effect
DHIS2: District Health Information System 2
eANC: Early antenatal care
EDD: Expected date of delivery
EMA: European Medicines Agency
FDA: Food and Drug Administration
GA: Gestational Age
GLMM: Generalized linear mixed model
HF: Health facility
HSTP: Health Sector Transformation Plan
ICC: Interclass correlation
IRB: Institutional Review Board
JUIH: Jimma University Institute of Health
LMM: Linear mixed model
LMP: Last menstrual period
MHSU: Maternal Health Service Utilization
MHS: Maternal Health Service
OR: Odds ratio
PNC: Postnatal care
RCT: Randomized Controlled Trial
SDG: Sustainable Development Goal
SMS: Short message service
SPSS: Software package for social science
VPN: Virtual private network
