# Supplementary material for: Evaluating gain and loss-framed mobile messaging to improve maternal health service uptake: A cluster randomized controlled trial in Jimma, Ethiopia
Source: PLoS One. 2026 Oct 26;21(10):e0360474. doi: 10.1371/journal.pone.0360474 (PMC13704815; doi:10.1371/journal.pone.0360474)
Supplement: Supplementary file 1 [file pone.0360474.s005.pdf]

Academic Editor Comments

1. In the Introduction, the authors state: "The impact of message-framing strategies on maternal health service uptake through mobile phone messaging has not been extensively studied, particularly in rural areas where barriers to accessing care are more pronounced."
   Could the authors please include references to studies, to whatever extent they exist, that have examined this issue? This would help to better frame the context and justify the need for the present study.

Response: Thank you for your constructive comments. We have updated the Introduction to reference relevant studies that have investigated message-framing strategies in maternity health contexts.

2. The authors must clearly state why Hooper and Bourke's longitudinal methodology was applied to this study design, given the limited number of measurement points (baseline and endline).

Response: We appreciate the editor's insightful question regarding our application of Hooper and Bourke's longitudinal analysis method in a study with only two measurement points (baseline and endline). Although the intervention was implemented simultaneously across all clusters, this method was appropriate because it accounts for both within-cluster and over-time correlation structures inherent in repeated measures cluster randomized trials, even when only two time points are available. In our study, we employed a repeated cross-sectional design, collecting outcome measures at baseline and endline from the same individuals within each cluster. This design necessitated adjustments for potential confounding due to time and intracluster correlation across time points. Hooper and Bourke's approach enabled us to address intracluster correlations both within and between periods during sample size estimation and statistical analysis. This enhanced the accuracy of our effect estimates and strengthened the robustness of our trial design. Given the cluster-randomized nature of our trial and the repeated cross-sectional measurement of outcomes, the application of Hooper and Bourke's method was methodologically sound and is detailed in the revised manuscript.

3. Are the primary outcomes patient-reported outcomes, or was an effort made to cross-verify the data from patients' medical records, such as ANC cards? Please clarify how the data were validated.

Response: The primary outcomes were self-reported outcomes collected via interviewer-administered structured questionnaires at baseline and endline. However, to improve the validity of the majority of self-reported information—especially on maternal health service utilization (e.g., number and timing of ANC visits, place of delivery, and PNC attendance)—data collectors tried to cross-check information with participants' ANC cards and other available medical records where feasible during the interview. This assisted in eliminating recall bias and improved accuracy. Moreover, the data collection

team consisted of trained health professionals familiar with maternal health data documentation, thus ensuring more credible information. All the questionnaires went through intense translation and back-translation, and a pretest was carried out to ensure clarity and uniformity of the instruments.

4. The authors should explicitly report the percentage of missing data (1.4%) and its nature (e.g., random or non-random). Additionally, please clarify the detailed procedure for multiple imputations, including the number of imputations performed, the predictors included in the model, and the convergence criteria used.

Response: Thank you for your constructive comments. We have now explicitly reported that 2.7% of the data were missing and, based on our analysis, the missingness was determined to be missing at random (MAR). We performed multiple imputations using the chained equations method with 20 imputations. Predictors included in the imputation model were age, parity, education level, baseline service utilization, and intervention arm.

5. In the ancillary analysis, the authors report: *"Additionally, the issues of long wait times and inconvenient hours were almost entirely resolved, decreasing from 98.6% to 1.4% and 95.8% to 4.2%, respectively. These changes indicate a substantial improvement in accessibility and perceived quality of health services over the period."* However, as the intervention was not directly designed to affect health service quality or waiting times, what factors might have led participants to report such improvements? The authors are encouraged to reflect on this finding and offer possible explanations.

Response: We acknowledge that the intervention did not directly target health service quality or waiting time. However, the improvements seen are probably a spillover effect of the messaging intervention: by increasing participants' awareness and readiness, they likely scheduled visits during less busy hours and communicated more effectively with providers, which reduced perceived wait times and inconvenience. Exposure to the intervention can also have internally encouraged health workers to provide services more effectively through friendlier interactions or increased transparency over delays, although provider behaviors were not formally measured in our study.

6. *Please ensure that all references are formatted according to the PLOS ONE referencing style.*

Response: We have thoroughly reviewed and reformatted all references to adhere to the PLOS ONE referencing style, ensuring consistency and compliance throughout the manuscript.

Additional requirements.

1. Please ensure that your manuscript meets PLOS ONE's style requirements, including those for file naming. The PLOS ONE style templates can be found at

https://journals.plos.org/plosone/s/file?id=wjVg/PLOSOne_formatting_sample_main_body.pdf and

https://journals.plos.org/plosone/s/file?id=ba62/PLOSOne_formatting_sample_title_authors_affiliations.pdf.

Response: Thank you for your constructive comments. We have thoroughly reviewed the PLOS ONE style guidelines—including the provided templates for manuscript formatting and title/affiliations—and have ensured our files comply exactly with their requirements.

2. Thank you for stating in your Funding Statement:

"This research was supported by grants from Jimma University for postgraduate students and additional funding from the Institute of Health, Department of Health, Behavior, and Society. The findings and conclusions are those of the authors and do not necessarily reflect the views of these organizations."

Please provide an amended statement that declares *all* the funding or sources of support (whether external or internal to your organization) received during this study, as detailed online in our guide for authors at http://journals.plos.org/plosone/s/submit-now. Please also include the statement "There was no additional external funding received for this study." in your updated Funding Statement.

Please include your amended Funding Statement within your cover letter. We will change the online submission form on your behalf.

Response: Thank you for your constructive comments. We have expanded our Funding Statement to accurately reflect all sources of support received during this study, as per PLOS ONE's guidelines. The updated statement is included below and has been inserted into our revised cover letter as requested.

3. In the online submission form you indicate that your data is not available for proprietary reasons and have provided a contact point for accessing this data. Please note that your current contact point is a co-author on this manuscript. According to our Data Policy, the contact point must not be an author on the manuscript and must be an institutional contact, ideally not an individual. Please revise your data statement to a non-author institutional point of contact, such as a data access or ethics committee, and send this to us via return email. Please also include contact information for the third party organization, and please include the full citation of where the data can be found.

Response: Thank you for your constructive comment, we have revised the data availability statement accordingly.

Revised Data Availability Statement: The de-identified data used in this study are **not publicly available online** because study participants **did not consent to public data release**; they only agreed to publication of aggregated results. However, the data can be accessed **upon reasonable request** through a **non-author institutional contact,** the **Jimma Zone Health Office,**

Department of Reproductive, Maternal, and Neonatal Health. Requests for data access should be sent to: jzhrd2015@gmail.com

Data Source (third-party, institutional custody): Jimma Zone Health Office, Department of Reproductive, Maternal, and Neonatal Health, Ethiopia.

Full citation of the data

Jimma Zone Health Office, Department of Reproductive, Maternal, and Neonatal Health. (2025). *De-identified dataset for the cluster randomized trial on maternal health service uptake in rural Jimma, Ethiopia.* Held by Jimma Zone Health Office. Available upon reasonable request via email: jzhrd2015@gmail.com .

4. Thank you for stating the following in the Acknowledgments Section of your manuscript:

“This research was supported by grants from Jimma University for postgraduate students and additional funding from the Institute of Health, Department of Health, Behavior, and Society. The findings and conclusions are those of the authors and do not necessarily reflect the views of these organizations.:

We note that you have provided additional information within the Acknowledgements Section that is not currently declared in your Funding Statement. Please note that funding information should not appear in the Acknowledgments section or other areas of your manuscript. We will only publish funding information present in the Funding Statement section of the online submission form.

Please remove any funding-related text from the manuscript and let us know how you would like to update your Funding Statement. Currently, your Funding Statement reads as follows:

“This research was supported by grants from Jimma University for postgraduate students and additional funding from the Institute of Health, Department of Health, Behavior, and Society. The findings and conclusions are those of the authors and do not necessarily reflect the views of these organizations.”

Please include your amended statements within your cover letter; we will change the online submission form on your behalf.

Response: Thank you for your constructive comments. We have expanded our Funding Statement to accurately reflect all sources of support received during the course of this study, as per PLOS ONE’s guidelines. The updated statement is included below and has been inserted into our revised cover letter as requested.

5. Your ethics statement should only appear in the Methods section of your manuscript. If your ethics statement is written in any section besides the Methods, please delete it from any other section.

Response: Thank you for your constructive comments. We have deleted from the other section and moved to method part accordingly

6. Please include captions for your Supporting Information files at the end of your manuscript, and update any in-text citations to match accordingly. Please see our Supporting Information guidelines for more information: http://journals.plos.org/plosone/s/supporting-information.

Response: Thank you for your constructive comments. We have included accordingly in the main manuscript

Reviewer #1 Comments

1. The authors have conducted a trial comparing two types of mobile phone messaging (gain and loss) with control in a cluster randomised trial. The terms gain-framed and loss-framed are not defined in the abstract and should be.

Response: Thank you for your constructive comments. We have revised the abstract to include definitions of gain-framed and loss-framed messages. Specifically, gain-framed messages emphasize the benefits of engaging in a behavior, while loss-framed messages highlight the consequences of not engaging in the behavior.

2. Likewise in the abstract, results need to be reported to a common degree of precision, it is standard to use 2dp throughout.

Response: Thank you for your comments. For consistency and clarity, we have standardized the reporting of all numerical results to two decimal places throughout the manuscript.

3. The sample size calculation here is clearly for two-comparisons versus control given the 2.5% two-sided significance level. Given this I'm unclear what the Bonferroni correction is in this context - the Z for the power should just be Z\beta and not Z\beta/2 This needs to be better explained; the numbers are correct for 80% power and 97.5% two-sided CI. The z here are 2.2414 and 0.8416. I think there is merely a little confusion in the write-up.

Response: Thank you for your comment. We conducted sample size calculation based on two main comparisons (gain-framed vs. control and loss-framed vs. control), and a Bonferroni correction was used to account for multiple comparisons (more than one statistical test), so each comparison was tested at a level of 0.025. We have clarified the manuscript to make this rationale and the resultant Z-values used in the calculation explicit.

4. Given the reliance on p<.025 the 95% CI do not match the test at the design stage and 97.5% CI need also to be given.

Response: Thank you for your constructive comments. We have updated the confidence intervals to 97.5% to align with the adjusted significance level used in the sample size calculation and hypothesis testing.

5. Given the small number of clusters here, it is important to identify if there is any confounding (e.g. husband's education) - there is a typo in this table which should read "College" and not "Collage"

Response: We have corrected the typographical error from "Collage" to "College" in the table 2. Additionally, we have analyzed to assess potential confounding factors, including husband's education, and have adjusted for these in our statistical models as appropriate.

6. Parity and number of family members able to read and write are not generally accepted to be Normally distributed. As such these should be tabulated.

Response: Thank you for your constructive comment. We have revised the manuscript to present parity and the number of literate family members as categorical variables in tabular form, providing frequencies and percentages to accurately reflect their distributions.

7. I am unclear what the starting figures for postnatal care etc actually mean here as they are presumably affected by parity. How can one be using postnatal care already and then later access antenatal care? How does one allow for primiparous women here if this is access for previous pregnancies? Where is parity in Table 1?

Response: Thank you for your comments. The baseline figures for postnatal care utilization refer to services accessed during previous pregnancies. In our study, we included only multiparous women who had at least one prior childbirth, ensuring that baseline data on postnatal care were applicable. Parity information was already included in Table 2, found on the last row of the table.

8. Table 3 needs to distinguish between within-patient data and between-patient data. I cannot reproduce the OR and CI from the data given here.

Response: Thank you for your observation. We have revised Table 3 to clearly distinguish between within-subject (paired) and between-subject data. Additionally, we have provided detailed information on the statistical methods used to calculate odds ratios and confidence intervals to facilitate reproducibility. The primary measure of association was the exponentiated coefficient, representing the odds ratio when using a binomial distribution with a logit link function.

9. Please give the actual p-values throughout - p<.05 is not the p-value specified.

Response: Thank you for your comments. We have reported the specified *p*-values throughout the manuscript.

10. In Table 4 the aMD for the control arm is not 1 but zero.

Response: Thank you for your comment , we have rectified this mistake in Table 4 by placing the control arm's adjusted mean difference (aMD) at zero since it serves as our reference group in the estimates.

11. Figure 2 represents paired data, and it si not clear who has moved from one group to another. It is unclear whether there are changes in the control arm also which would indicate a desire to please.

Response: We acknowledge the need for clarity in Figure 2. We have revised the figure to better illustrate changes within each group over time and have included annotations to indicate shifts in barriers to service utilization. Additionally, we have discussed the potential for social desirability bias in the control group and its implications in the manuscript.

Reviewer #2 Comments

1. *Title: The title is informative and conveys the key components of the study. However, it should be more concise and polished for better readability. For instance: "Evaluating Gain and Loss-Framed Mobile Messaging to Improve Maternal Health Service Uptake: A Cluster Randomized Trial in Rural Jimma, Ethiopia."*

Response: Thank you for the suggestion. We have revised the title for clarity and conciseness to: “Evaluating Gain- and Loss-Framed Mobile Messaging to Improve Maternal Health Service Uptake: A Cluster-Randomized Trial in Rural Jimma, Ethiopia.”

2. Keywords No revisions required.

Response: We appreciate your confirmation. No changes were made to the keywords.

3. *Abstract: The abstract is well-structured and follows a logical sequence. Improvements are needed to trim excessive detail, ensure consistent terminology (e.g., "self-efficacy"), and enhance the conclusion to emphasize the significance of the findings more effectively.*

Response: Thank you for your constructive comments. We have edited the abstract by removing excessive details, ensuring consistent use of terminology such as "self-efficacy," and strengthening the conclusion to better highlight the significance and implications of our findings.

4. Trial Registration Trial registration details are inaccessible online. Authors should confirm registration compliance, including providing the registration number, registry name, and URL if available. Any missing details must be addressed or explicitly acknowledged to ensure transparency.

Response: Thank you for your comment. The trial is registered with the Pan African Clinical Trials Registry: PACTR202201753436676. Registry name “Pan African Clinical Trials Registry” and URL https://tinyurl.com/ykhnpc49 have been added to the manuscript. We have confirmed that the trial was prospectively registered on 4 January 2022

5. Background The section contains the required elements but lacks flow and coherence. A structured writing style should be followed, such as moving from global to local contexts or vice versa. Additionally, ideas should align closely with the study objectives for better synchronization.

Response: Thank you for your constructive feedback. We have reorganized the background section of the manuscript to enhance its structure and coherence.

6. Method Setting: The criteria for selecting Manna, Shebe Sombo, and Dedo districts from the Jimma Zone must be clarified

Response: Thank you for your comments. Manna, Shebe-Sombo, and Dedo were purposively selected because they: a) have no ongoing similar mHealth interventions, b) had functional health posts, c) had good mobile network coverage, and d) provided geographic diversity within Jimma Zone. We have edited the manuscript accordingly

7. Population: The inclusion of primiparous women in the sample is unclear, as baseline data were collected from those with at least one prior childbirth. Clarification is needed

Response: Thank you for your insightful comment. According to standard obstetric definitions, a primiparous woman is one who has given birth once to a fetus at or beyond the viable gestational age, typically ≥20 weeks, regardless of whether the baby was born alive or stillborn. This classification remains until she completes her second birth, at which point she becomes multiparous (para 2). In our study, we have included women who are currently pregnant and have previously given birth once, as per the definition of primiparity. This inclusion aligns with the WHO's standard obstetric definitions and ensures consistency in our classification (https://iiab.me/kiwix/content/wikipedia_en_all_maxi_2023-10/A/Gravidity_and_parity?utm_source=chatgpt.com).

8. Sample size: The sample size appears insufficient considering 21 clusters. Consultation with a statistician is recommended to justify whether the current sample size is adequate. If 21 clusters are considered for sample size calculation, the required sample size would be much larger.

Response: Applying the well-established method of Hooper & Bourke to cluster-randomized trials with endline and baseline data, we properly controlled for our sample size within-period clustering (ICC ≈ 0.003) and for cluster-period level autocorrelation ($\pi \approx 0.8$), generating a combined design effect (DE = dc × dr ≈ 1.116) (https://www.sciencedirect.com/science/article/pii/S2950433324000090?utm_source=chatgpt.com). Beginning with the individually randomized estimate (n_0 = 175 per arm for a difference of 15 points in ANC4+ attendance with 80% power and α = 0.025) and growing by DE and 4% loss to follow-up equals 196 women in each arm—or 28 participants per health post in 21 clusters. This is in line with best practice that demonstrates baseline-endline

cohort designs significantly enhance efficiency through the use of within-cluster and within-individual correlation and reduces clusters needed compared to cross-sectional CRTs (https://pmc.ncbi.nlm.nih.gov/articles/PMC7334046/?utm_source=chatgpt.co . Therefore, given our conservative estimate of correlation, number of clusters, and effect size, the sample size is statistically adequate for the detection of the intervention effect of interest.

9. Intervention development: Correct flow and remove repetitions, e.g., the repeated definition of loss frame and word frame (lines 182–184 and 216–218). Clarify who delivered the SMS messages—telecommunication personnel, researchers, or others—and whether mechanisms were in place to confirm participants received and read the messages.

Response: We've removed duplicate definitions of "gain" and "loss" frames. We've clarified SMS dispatch as messages were sent via an automated platform managed by Jimma University researchers. Reading receipts were not feasible, but automated delivery logs were used. Volunteers conducted monthly follow-up calls to confirm receipt and engagement.

10. Blinding: Line 297 states, "Blinding the participants was not feasible due to the nature of the intervention." Clarify why SMS interventions made blinding impossible.

Response : Thank you for your constructive comments. We added the reason to the main document as: Participants could easily identify their arm based on message tone ("gain" vs "loss"), making participant blinding impossible. Data collectors were blinded to the assignment of participants to reduce bias.

11. Data Collection Procedure Line 343-344 mentions, "Baseline data were collected using a paper-based method, while endline data were collected using the Kobo toolkit." Clarify whether this methodological difference could introduce bias.

Response: We acknowledged this switch and added a discussion on potential information bias. However, both tools used identical question structures, and data officers received rigorous training to harmonize protocols. We now include this in the limitations section.

12. Analyses The manuscript states that "Generalized Linear Mixed Model (GLMM)" was used, but no GLMM results appear in the results section. This requires justification. Additionally, explain why GLMM was chosen instead of Generalized Estimating Equations (GEE), which are often preferred for non-normally distributed data. Result The first four sentences of this section belong to the methods section and create redundancy, so they should be removed.

Response: Thank you for your constructive comments. All findings in the Results section derive from generalized linear mixed models (GLMMs). The primary measure of association was the exponentiated coefficient, corresponding to the odds ratio under a binomial distribution with a logit link. We chose GLMMs to account for our hierarchical design (individuals nested within clusters) and to accommodate both random effects and missing data. Additionally, we have removed the first four redundant sentences as suggested and also updated some of the results accordingly.

13. Include data for both baseline and endline participant characteristics, rather than focusing solely on baseline.

Response : Thank you for your comment**.** Because both baseline and endline data were collected from the same participants, we operated under the assumption that no significant demographic changes would occur over the eight-month intervention period. This is consistent with longitudinal cohort study designs in similar fields, where demographic variables (such as age, education level, marital status, etc.) typically remain stable over such a timeframe (https://pmc.ncbi.nlm.nih.gov/articles/PMC7514084/?utm_source=chatgpt.com)

14. Address the significant differences in ANC utilization rates between the gain and loss frame groups at baseline (85.7% vs. 90.8%). Clarify how these differences were managed to ensure the conclusions are attributable to messaging effects rather than baseline disparities.

Response : We acknowledge the baseline ANC use difference (85.7% vs. 90.8%) between loss- and gain-frame groups. However, as participants were randomly assigned, such a difference should expected by chance instead of systematic bias. To keep our results representative of messaging effects—rather than baseline contrasts—we controlled for baseline ANC use in all primary analyses through ANCOVA, in accordance with FDA and EMA recommendations that adjustment of prognostic baseline covariates stabilizes chance imbalances and improves precision without invalidating inference https://www.fda.gov/media/123801/download?utm_source=chatgpt.com , https://arxiv.org/abs/1207.5625?utm_source=chatgpt.com . Our controlled models (adding baseline utilization) yielded effect estimates that aligned with unadjusted and change-score analyses, to ensure that the messaging frame alone was responsible for the resulting differences.

15. The observed changes in institutional births are exceptionally significant, with a 37% increase in the gain-framed group and a 26% increase in the loss-framed group. These findings warrant a thorough review to ensure the validity and accuracy of the data. The reviewer strongly recommends revisiting the original dataset and conducting reanalysis if necessary to confirm the reliability of these results. If verified, such outcomes would represent an unprecedented level of effectiveness with profound global implications. However, the magnitude of these changes, described as world-leading, raises concerns

about data integrity and representation. The authors should scrutinize the findings to ensure their accuracy and reliability.

Response: We re-examined the raw data against facility records. The reported effect sizes were confirmed. Institutional delivery increased from 68.4% to 94.7% in the gain group and from 73% to 99% in the loss group. We also discussed the exceptional nature of these gains in the discussion with contextual references.

16. Additionally, while ancillary analyses are noteworthy, it is crucial to first evaluate whether secondary variables—such as knowledge, attitude, self-efficacy, and maternal morbidity—predicted antenatal care (ANC), delivery, and postnatal care utilization.

Response : Thank you for your constructive comments. We have conducted exploratory regression analyses to determine whether improvements in self-efficacy and ANC knowledge significantly predicted increased ANC attendance and facility delivery.

17.Discussion. This section is acceptable but must be revised in light of changes to previous sections to ensure consistency.

Response: The Discussion has been updated to incorporate adjusted findings, baseline adjustments, and confirmation of large intervention effects. We differentiated between gain and loss frame effectiveness and integrated secondary predictor analysis.

18. Conclusion The conclusion suggests that both gain and loss frames are effective, but the authors should clearly indicate which approach is more effective. Numerical figures, such as "gain-framed arm improved ANC by 11.6%, institutional births by 26.3%, PNC by 12.8%, etc.," should not appear in the conclusion. Additionally, ensure all reported figures match the results section.

Response: We revised the conclusion to add: "Although both gain- and loss-framed messages substantially improved maternal health service utilization, gain-framed messages had a greater effect on institutional delivery. All numeric values were also removed."

19. Reference Numerous references, such as "WHO Maternal Health. Who 2018, 1–47," are not cited correctly according to the Vancouver style. For example, this reference lacks the title. Use reference management software such as Mendeley to correct these errors systematically.

Response: We conducted a thorough reference audit using Mendeley, to correct all citations

20. Language Ensure coherent descriptions to avoid disconnections and unnecessary repetitions. Multiple grammatical, typographical, and spelling errors must be corrected through thorough editing.

Response: All grammatical, typographical, and stylistic issues have been duly corrected throughout the manuscript. We have also eliminated unnecessary repetitions and enhanced overall readability.
