# Supplementary material for: Evaluating gain and loss-framed mobile messaging to improve maternal health service uptake: A cluster randomized controlled trial in Jimma, Ethiopia
Source: PLoS One. 2026 Oct 26;21(10):e0360474. doi: 10.1371/journal.pone.0360474 (PMC13704815; doi:10.1371/journal.pone.0360474)
Supplement: Supplementary file 2 [file pone.0360474.s006.pdf]

ACADEMIC EDITOR:

Comment 1: The sample size calculation is still not reproducible. Please show the numeric values substituted into the equation (control proportion, expected difference, ICC, autocorrelation, Z-values).

Response : Thank you for your helpful comment. We have now included the full numeric substitution in the sample size formula for the cluster-randomized trial within the main manuscript to ensure that the calculation is fully reproducible.

Comment 2: In Table 3 and elsewhere, it is unclear whether odds ratios are based on endline data only or adjusted for baseline. Define the effect sizes clearly in table footnotes.

Response : Thank you for your comment. We apologise for the previous lack of clarity regarding the derivation of the odds ratios in Table 3 and elsewhere. We confirm that the estimates are derived from a generalized linear mixed model (GLMM) comparing each intervention arm (gain-framed and loss-framed) with the control arm at endline, and the models adjust for the baseline value of the respective outcome, relevant covariates and clustering. We have updated the manuscript accordingly: the table footnotes now clearly define the effect sizes . Baseline sample size: total n = 588 (each arm n = 196). Endline sample size: total n = 572 (gain-framed = 187; loss-framed = 192; control = 193).

Comment 3: Outcome verification remains unclear. Please indicate the proportion of outcomes cross-checked against ANC cards or explain how this was handled.

Response : Thank you for your comment. We have now clarified the outcome verification process in the revised manuscript. Approximately 82% of the reported outcomes were cross-checked against participants' cards. For the remaining participants without documentation, self-reported data were retained after confirming internal consistency through probing questions regarding the number and timing of visits, the facility attended, and the timing of delivery or postnatal check-ups.

Comment 4: Baseline and endline demographics should be presented in a simple table, which can be included as a supplementary file if needed.

Response : Thank you for the helpful comment. Given that no significant demographic changes are expected within the eight-month intervention period, we believe presenting only baseline characteristics is sufficient to describe the comparability of groups at the start of the study. Therefore, we have retained the baseline demographics table, which summarizes participants’ key characteristics across study arms, and did not include endline demographics to avoid unnecessary repetition.

Comment 5: The significant gains in institutional delivery and other outcomes remain unusual. While you noted spillover and social desirability bias, the Discussion should more fully acknowledge other possible explanations (e.g., concurrent health campaigns, system-level changes) and adopt a more cautious interpretation.

Response : Thank you very much for your valuable feedback. During our study period there were community-based activities such as monthly conferences for pregnant women and male partner engagement, door-to-door registration of pregnant women by volunteers, and community bylaws enforcing a `zero tolerance` sanction for home delivery. In addition, broader health-system improvements like increased outreach, facility readiness, normative shifts may have played a role.

Comment 6: The Methods and Results remain overly dense, with long sentences and extensive technical detail. Streamlining would improve readability.

Response : Thank you for your valuable feedback. We recognise that the Methods and Results sections were too dense, and have now streamlined both sections to improve readability.

Comment 7: The Discussion should be restructured so that strengths, limitations, and implications are presented in separate, clearly defined paragraphs.

Response : Thank you for the valuable suggestion. The discussion section has been restructured to enhance clarity and organization. Specifically, the strengths, limitations, and implications of the study are now presented in distinct, clearly defined paragraphs to improve readability and ensure that each aspect is discussed comprehensively.

Comment 8: The manuscript still contains phrasing and grammatical inconsistencies. A final round of English language editing is recommended to improve clarity and flow.

Response : Thank you very much for your feedback. We have fully edited the manuscript—polishing sentence structure, phrasing, grammar, and readability. We believe the revised version now reads more clearly and flows more smoothly.

Reviewer #1:

Comment 1: It may feel a small point but in a 3 arm study, randomisation is surely 1:1:1 if all arms are due to have equal size.

Response : Thank you for pointing this out. You are absolutely correct that in a 3-arm trial where all arms are intended to be of equal size, the allocation ratio should indeed be 1:1:1 (i.e., each participant has an equal chance of being assigned to any of the three arms). We have corrected it accordingly.

Comment 2: I'm really unclear that in the abstract the name of the confidence intervals has changed from 95% to 97.5% but the confidence intervals remain the same. Were

these originally 97.5% CI (i.e. with long(OR)+/- 2.24 x s.e.) or were they set +/- 1.96 se - in which case they were 95% CI and the CI need to change to match the fact that they need to be wider to reflect multiplicity and the Bonferroni correction.

Response : Thank you for your constructive comment. We sincerely apologize for the oversight. We have re-calculated the confidence intervals at the 97.5% confidence level to reflect the Bonferroni correction and have updated the Abstract, Methods, and Results sections accordingly with the correct numerical bounds and clarity of reporting.

Comment 3: The sample size calculation does not make the values put into the equation explicit as promised in the response.

Response : Thank you for your comment. We have now explicitly included all the parameter values used in our sample-size calculation in the revised Methods section to ensure transparency and reproducibility—giving the assumptions for power, alpha, effect size, design effect and attrition rate.

Comment 4: Table 3 does not define what the OR is measuring - is it just on the post, or is it adjusted for pre as well? Likewise for other tables - -please define the actual effect size being calculated here.

Response : Thank you for the insightful comment. The odds ratios (ORs) presented in Table 3 and subsequent tables represent post-intervention effects that are adjusted for baseline values. Specifically, the ORs were derived from generalized linear mixed models (GLMMs) that accounted for clustering at the kebele level and controlled for baseline outcome measures. Thus, the reported ORs reflect the adjusted effect sizes comparing each intervention arm (gain- or loss-framed) to the control group after controlling for pre-intervention differences.
