# Supplementary material for: Evaluating gain and loss-framed mobile messaging to improve maternal health service uptake: A cluster randomized controlled trial in Jimma, Ethiopia
Source: PLoS One. 2026 Oct 26;21(10):e0360474. doi: 10.1371/journal.pone.0360474 (PMC13704815; doi:10.1371/journal.pone.0360474)
Supplement: Supplementary file 3 [file pone.0360474.s008.pdf]

PLOS ONE

Effectiveness of Mobile Phone Messaging Based Gain and Loss-Framed Approaches for Improving Maternal Health Service Uptake in Rural Jimma Zone, Ethiopia: A Three-Arm Cluster Randomized Controlled Trial

--Manuscript Draft--

**Manuscript Number:**	PONE-D-25-10242
**Article Type:**	Clinical Trial
**Full Title:**	Effectiveness of Mobile Phone Messaging Based Gain and Loss-Framed Approaches for Improving Maternal Health Service Uptake in Rural Jimma Zone, Ethiopia: A Three-Arm Cluster Randomized Controlled Trial
**Short Title:**	Mobile Phone Messaging Based Gain and Loss-Framed Approaches for Improving Maternal Health Service Uptake
**Corresponding Author:**	Gebeyehu Bulcha Duguma, MPH, PhD<br>Jimma University<br>Jimma, ETHIOPIA
**Keywords:**	Message framing; digital health; mobile phone messaging; maternal health; cluster randomized controlled trials; Ethiopia.
**Abstract:**	Background: Despite reductions in maternal mortality in Ethiopia, rural areas still struggle with accessing maternal health services. Innovative solutions, such as mobile phone-based gain or loss-framed messaging, offer promise for improving maternal health outcomes. This study aims to evaluate the effectiveness of these messaging strategies in promoting maternal health in Jimma Zone, Ethiopia.<br>Methods: A three-arm cluster-randomized controlled trial design was used. Twenty-one kebeles (clusters) were randomly assigned to the gain-framed group (Arm 1), the loss-framed group (Arm 2), and the control arm, using simple randomization with a 1:1 allocation ratio. A total of 588 pregnant women with a gestational age of 16–20 weeks were randomly selected, with 196 participants in each study arm. The recruitment of intervention participants was conducted in March 2023, and the intervention follow-up took place from May 2023 to December 2023. The intervention effect on the primary outcomes (antenatal care, institutional delivery and postnatal care) was analyzed with a generalized linear mixed model, and the secondary outcomes (knowledge, attitude, self efficacy and maternal morbidity) were analyzed using a linear mixed model.<br>Result: The gain-framed arm showed significantly higher utilization for antenatal care (OR: 2.6; 95% CI: 1.30 to 5.14), and postnatal care (OR: 2.6; 95% CI: 1.36 to 5.06), all compared to the control arm. The loss-framed arm also showed higher utilization for overall maternal health services (OR: 2.7; 95% CI: 1.49 to 4.98), antenatal care (OR: 4.1; 95% CI: 1.99 to 8.52), institutional births (OR: 3.3; 95% CI: 1.17 to 9.32), and postnatal care (OR: 2.0; 95% CI: 1.02 to 3.73), all compared to the control arm. Knowledge and attitudes toward maternal health services improved significantly in both intervention arms. Self-efficacy saw modest improvement, while maternal morbidity decreased in both intervention arms compared to the control.<br>Conclusion: Both gain-framed and loss-framed messages led to significant increases in maternal health services utilization, knowledge, attitude, and self-efficacy, while also reducing maternal morbidity. Loss-framed messaging demonstrated greater effectiveness in improving antenatal care and institutional births, while gain-framed messaging was more effective in enhancing postnatal care utilization.<br>Trial registration: Pan African Clinical Trials Registry (PACTR), PACTR202201753436676, registered on January 4, 2022.<br>Keywords: Message framing; digital health; mobile phone messaging; maternal health; cluster randomized controlled trials; Ethiopia.
**Order of Authors:**	Gebeyehu Bulcha Duguma, MPH, PhD
	Hordofa Gutema, MPH, PhD
	Mulusew Gerbaba
	Demisew Amenu, MD, PhD

Powered by Editorial Manager® and ProduXion Manager® from Aries Systems Corporation

	Zewdie Birhanu, MPH, PhD
**Additional Information:**	
**Question**	**Response**
**Financial Disclosure**<br><br>Enter a financial disclosure statement that describes the sources of funding for the work included in this submission. Review the submission guidelines for detailed requirements. View published research articles from *PLOS ONE* for specific examples.<br><br>This statement is required for submission and **will appear in the published article** if the submission is accepted. Please make sure it is accurate.<br><br>**Funded studies**<br>Enter a statement with the following details:<br>• Initials of the authors who received each award<br>• Grant numbers awarded to each author<br>• The full name of each funder<br>• URL of each funder website<br>• Did the sponsors or funders play any role in the study design, data collection and analysis, decision to publish, or preparation of the manuscript?<br><br>Did you receive funding for this work?	Yes
Please add funding details.<br>as follow-up to "**Financial Disclosure**<br><br>Enter a financial disclosure statement that describes the sources of funding for the work included in this submission. Review the submission guidelines for detailed requirements. View published research articles from *PLOS ONE* for specific examples.<br><br>This statement is required for submission and **will appear in the published article** if the submission is accepted. Please make sure it is accurate.	This research was supported by grants from Jimma University for postgraduate students and additional funding from the Institute of Health, Department of Health, Behavior, and Society. The findings and conclusions are those of the authors and do not necessarily reflect the views of these organizations.

**Funded studies**<br>Enter a statement with the following details:<br>• Initials of the authors who received each award<br>• Grant numbers awarded to each author<br>• The full name of each funder<br>• URL of each funder website<br>• Did the sponsors or funders play any role in the study design, data collection and analysis, decision to publish, or preparation of the manuscript?<br><br>Did you receive funding for this work?"	
Please select the country of your main research funder (please select carefully as in some cases this is used in fee calculation).<br>as follow-up to "**Financial Disclosure**<br><br>Enter a financial disclosure statement that describes the sources of funding for the work included in this submission. Review the submission guidelines for detailed requirements. View published research articles from *PLOS ONE* for specific examples.<br><br>This statement is required for submission and **will appear in the published article** if the submission is accepted. Please make sure it is accurate.<br><br>**Funded studies**<br>Enter a statement with the following details:<br>• Initials of the authors who received each award<br>• Grant numbers awarded to each author<br>• The full name of each funder<br>• URL of each funder website<br>• Did the sponsors or funders play any role in the study design, data collection and analysis, decision to publish, or preparation of the manuscript?<br><br>Did you receive funding for this work?"	ETHIOPIA - ET
**Competing Interests**	The authors declare that they do not have competing interests.

Use the instructions below to enter a competing interest statement for this submission. On behalf of all authors, disclose any competing interests that could be perceived to bias this work—acknowledging all financial support and any other relevant financial or non-financial competing interests.<br><br>This statement is required for submission and **will appear in the published article** if the submission is accepted. Please make sure it is accurate and that any funding sources listed in your Funding Information later in the submission form are also declared in your Financial Disclosure statement.<br><br>View published research articles from *PLOS ONE* for specific examples.<br><br>**NO authors have competing interests**<br><br>Enter: *The authors have declared that no competing interests exist.*<br><br>**Authors with competing interests**<br><br>Enter competing interest details beginning with this statement:<br><br>*I have read the journal's policy and the authors of this manuscript have the following competing interests: [insert competing interests here]*<br><br>* typeset	
**Ethics Statement**<br><br>Enter an ethics statement for this submission. This statement is required if the study involved:<br><br>• Human participants<br>• Human specimens or tissue<br>• Vertebrate animals or cephalopods<br>• Vertebrate embryos or tissues	The Jimma University Institutional Review Board approved the study (ref no-JUIH/IRB/316/23). Participants gave informed consent before the intervention and assessments. Consent details, including study objectives and participant roles, were explained in the local language. Confidentiality was ensured by encrypting phone numbers, accessible only to server technicians and researchers, with data shared after full de-identification.

• Field research<br><br>Write "N/A" if the submission does not require an ethics statement.<br><br>General guidance is provided below. Consult the submission guidelines for detailed instructions. **Make sure that all information entered here is included in the Methods section of the manuscript.**	

Powered by Editorial Manager® and ProduXion Manager® from Aries Systems Corporation

**Format for specific study types**<br><br>**Human Subject Research (involving human participants and/or tissue)**<br>• Give the name of the institutional review board or ethics committee that approved the study<br>• Include the approval number and/or a statement indicating approval of this research<br>• Indicate the form of consent obtained (written/oral) or the reason that consent was not obtained (e.g. the data were analyzed anonymously)<br><br>**Animal Research (involving vertebrate animals, embryos or tissues)**<br>• Provide the name of the Institutional Animal Care and Use Committee (IACUC) or other relevant ethics board that reviewed the study protocol, and indicate whether they approved this research or granted a formal waiver of ethical approval<br>• Include an approval number if one was obtained<br>• If the study involved *non-human primates*, add *additional details* about animal welfare and steps taken to ameliorate suffering<br>• If anesthesia, euthanasia, or any kind of animal sacrifice is part of the study, include briefly which substances and/or methods were applied<br><br>**Field Research**<br><br>Include the following details if this study involves the collection of plant, animal, or other materials from a natural setting:<br>• Field permit number<br>• Name of the institution or relevant body that granted permission	
**Data Availability**<br><br>Authors are required to make all data underlying the findings described fully available, without restriction, and from the time of publication. PLOS allows rare exceptions to address legal and ethical concerns. See the PLOS Data Policy and FAQ for detailed information.	Yes - all data are fully available without restriction

A Data Availability Statement describing where the data can be found is required at submission. Your answers to this question constitute the Data Availability Statement and **will be published in the article**, if accepted.<br><br>**Important:** Stating 'data available on request from the author' is not sufficient. If your data are only available upon request, select 'No' for the first question and explain your exceptional situation in the text box.<br><br>Do the authors confirm that all data underlying the findings described in their manuscript are fully available without restriction?	
**Describe where the data may be found in full sentences. If you are copying our sample text, replace any instances of XXX with the appropriate details.**<br><br>• If the data are **held or will be held in a public repository**, include URLs, accession numbers or DOIs. If this information will only be available after acceptance, indicate this by ticking the box below. For example: *All XXX files are available from the XXX database (accession number(s) XXX, XXX.).*<br>• If the data are all contained **within the manuscript and/or Supporting Information files**, enter the following: *All relevant data are within the manuscript and its Supporting Information files.*<br>• If neither of these applies but you are able to provide **details of access elsewhere**, with or without limitations, please do so. For example:<br><br>*Data cannot be shared publicly because of [XXX]. Data are available from the XXX Institutional Data Access / Ethics Committee (contact via XXX) for researchers who meet the criteria for access to confidential data.*<br><br>*The data underlying the results presented in the study are available from (include the name of the third party*	Data used for this analysis can be provided by the authors upon reasonable request to the corresponding author, Gebeyehu Bulcha (gebeyehubulcha@gmail.com).

and contact information or URL).<br>• This text is appropriate if the data are owned by a third party and authors do not have permission to share the data.<br><br>* typeset	
Additional data availability information:	

Powered by Editorial Manager® and ProduXion Manager® from Aries Systems Corporation

Manuscript
Click here to access/download;Manuscript;Manuscript_Effectiveness_01.docx

Effectiveness of Mobile Phone Messaging Based Gain and Loss-Framed Approaches for Improving Maternal Health Service Uptake in Rural Jimma Zone, Ethiopia: A Three-Arm Cluster Randomized Controlled Trial

Gebeyehu Bulcha [1,2*], Hordofa Gutema [1], Mulusew Gerbaba[3], Demisew Amenu [4], Zewdie Birhanu [1]

[1]Department of Health, Behavior, and Society, Faculty of Public Health, Institutes of Health, Jimma University, Jimma, Ethiopia
[2]Oromia Regional State Health Bureau, Jimma Zone Health Office, Department of Maternal, Newborn and Child Health, Jimma, Ethiopia
[3]Data Science and Evaluation, African Population and Health Research Center, Nairobi, Kenya
[4]Department of Obstetrics and Gynecology, Faculty of Medical Sciences, Institutes of Health, Jimma University, Jimma, Ethiopia

Trial Registration: Pan African Clinical Trials Registry (PACTR), PACTR202201753436676**,** registered on January 4, 2022**.**

* Corresponding Author
Email: Gebeyehu Bulcha Duguma, email: gebeyehubulcha@gmail.com

Abstract

Background: Despite reductions in maternal mortality in Ethiopia, rural areas still struggle with accessing maternal health services. Innovative solutions, such as mobile phone-based gain or loss-framed messaging, offer promise for improving maternal health outcomes. This study aims to evaluate the effectiveness of these messaging strategies in promoting maternal health in Jimma Zone, Ethiopia.

Methods: A three-arm cluster-randomized controlled trial design was used. Twenty-one kebeles (clusters) were randomly assigned to the gain-framed group (Arm 1), the loss-framed group (Arm 2), and the control arm, using simple randomization with a 1:1 allocation ratio. A total of 588 pregnant women with a gestational age of 16–20 weeks were randomly selected, with 196 participants in each study arm. The recruitment of intervention participants was conducted in March 2023, and the intervention follow-up took place from May 2023 to December 2023. The intervention effect on the primary outcomes (antenatal care, institutional delivery and postnatal care) was analyzed with a generalized linear mixed model, and the secondary outcomes (knowledge, attitude, self efficacy and maternal morbidity) were analyzed using a linear mixed model.

Result: The gain-framed arm showed significantly higher utilization for antenatal care (OR: 2.6; 95% CI: 1.30 to 5.14), and postnatal care (OR: 2.6; 95% CI: 1.36 to 5.06), all compared to the control arm. The loss-framed arm also showed higher utilization for overall maternal health services (OR: 2.7; 95% CI: 1.49 to 4.98), antenatal care (OR: 4.1; 95% CI: 1.99 to 8.52), institutional births (OR: 3.3; 95% CI: 1.17 to 9.32), and postnatal care (OR: 2.0; 95% CI: 1.02 to 3.73), all compared to the control arm. Knowledge and attitudes toward maternal health services improved significantly in both intervention arms. Self-efficacy saw modest improvement, while maternal morbidity decreased in both intervention arms compared to the control.

Conclusion: Both gain-framed and loss-framed messages led to significant increases in maternal health services utilization, knowledge, attitude, and self-efficacy, while also reducing maternal morbidity. Loss-framed messaging demonstrated greater effectiveness in improving antenatal care and institutional births, while gain-framed messaging was more effective in enhancing postnatal care utilization.

Trial registration: Pan African Clinical Trials Registry (PACTR), **PACTR202201753436676**, registered on **January 4, 2022**.

Keywords: Message framing; digital health; mobile phone messaging; maternal health; cluster randomized controlled trials; Ethiopia.

Background

Maternal health remains a pressing global health priority due to the significant burden of disease and mortality among this population group [1] . Maternal mortality rates are still high globally, with the majority of these deaths occurring in low- and middle-income countries where sexual, reproductive, maternal, and adolescent health facilities are often inadequate [2]. In many regions of Africa, the Maternal Mortality Ratio is very high, accounting for approximately 69% of the global maternal deaths. Major barriers to accessing quality maternal health care contribute to persistent disparities in maternal health outcomes in most countries of the African region [3]. Most maternal deaths are due to preventable factors such as infectious diseases, complications during pregnancy, labor, childbirth, and the postpartum period [4].

In Ethiopia, maternal mortality has declined over the years, with the ratio decreasing from 871 per 100,000 live births in 2000 to 412 per 100,000 in 2016, and further to 267 per 100,000 in 2020 [5]. Despite these advances, maternal mortality remains far from the Sustainable Development Goal (SDG) target of 70 deaths per 100,000 live births [6]. According to the Ethiopian Demographic and Health Survey (EDHS) 2019, only 43% of women had at least four antenatal care visits, only 48% had their birth attended to by a skilled attendant, and 34% reported receiving postnatal care within two days after delivery [7]. To address these challenges, the Ethiopian government has implemented various health sector strategies, including the Health Sector Transformation Plan (HSTP), which aims to improve maternal health outcomes through comprehensive health services and community-based interventions. Despite these efforts, gaps in maternal health service uptake persist, particularly in rural areas where logistical barriers, lack of awareness, and cultural factors impede access to care [8].

Strengthening health services, ensuring skilled attendance at every birth, providing hospital care for emergencies, and offering comprehensive prenatal, childbirth, and postnatal care are essential for promoting maternal survival and laying the foundations for long-term health [9]. Educating and equipping mothers with the skills and methods to access appropriate care have been identified as major determinants of enhanced maternal health service utilization and can greatly improve maternal health outcomes [10].

One potential solution for lowering the current extraordinarily high maternal death rates in low-income countries is to introduce innovative healthcare delivery methods, such as mobile phone-based messaging[11]. Mobile phone-based interventions hold great promise for enhancing healthcare uptake, particularly in resource-limited settings. Research has shown that mobile phone messaging has the potential to improve the maternal-infant continuum of care [12], self-management of diabetes, weight loss, physical activity, quitting smoking, and adherence to medication in antiretroviral therapy [13], and it has also been suggested as an effective way to educate women about their pregnancies [14].

Studies have demonstrated that persuasive messages can induce attitude changes, which, in turn, lead to changes in intention and behavior. The framing of a message affects the level of persuasion it elicits. Message framing can evoke fear and persuade individuals to engage in desired behaviors by emphasizing either the benefits of engaging in the behavior or the drawbacks of not engaging. Decision-makers react differently to various, though objectively comparable, explanations of the same issue depending on whether the emphasis is on the advantages or disadvantages of a behavior. A positively framed message highlights behavioral outcomes with a positive valence, while a negatively framed message depicts outcomes with a negative resonance[15,16].

Gain-framed messages focus on the benefits of engaging in specific health behaviors, while loss-framed messages focus on the risks or negative consequences of not doing so [17]. In some contexts, gain-framed messages have been found effective in encouraging preventive health behaviors by highlighting the positive outcomes of engaging in specific actions, while loss-framed messages emphasize the negative consequences of not engaging in those behaviors [18,19]. There are no systematic studies that have investigated the effectiveness of gain- and loss-framed messages in maternal health service use. However, research has examined the effect of message framing on other health behaviors, such as child nutrition, immunization, and overall health promotion.These studies have shown mixed results regarding the relative effectiveness of gain- versus loss-framed messages in improving maternal health behaviors and outcomes. For example, a randomized controlled trial in Iran found that loss-framed messages were more effective than gain-framed messages in improving maternal attitudes, behavior toward the diversity and appropriateness of continuing complementary feeding, and the timely onset of

complementary feeding in infants. The study concluded that loss-framed messages had a greater impact on maternal behaviors and perceptions regarding infant feeding practices [20]. In contrast, another study found that gain-framed messages promoting parenting practices to reduce children's sugar-sweetened beverage intake—such as role modeling healthy beverage intake and making healthy beverages available—were associated with higher motivation scores than loss-framed messages for all parents and most subgroups [19].

The impact of message-framing strategies on maternal health service uptake through mobile phone messaging has not been extensively studied, particularly in rural areas where barriers to accessing care are more pronounced. Given the high maternal mortality rates in rural population and the need for effective, low-cost interventions to improve maternal health services uptake, exploring the impact of gain and loss-framed mobile phone messaging is of paramount importance. By conducting a three-arm cluster-randomized controlled trial (cRCT), this study tested which type of message framing (gain or loss) has a greater effect in improving ANC attendance, institutional delivery, PNC, maternal knowledge, maternal attitudes, self-efficacy, and reduce maternal morbidity.

Methods and Materials

The trial protocol has been published previously [21], and the trial was registered on January 4, 2022, with Clinical Trials (trial identifier PACTR202201753436676) and can be accessed at https://pactr.samrc.ac.za. The full study protocol approved by the ethics committee is available in Supporting Information **(S1 Protocol).**

Setting

The study was conducted in three districts of Jimma Zone, Oromia, Ethiopia: Manna, Shebe Sombo, and Dedo of Jimma Zone. Jimma Zone is characterized by poor health infrastructure, with a low health worker density according to WHO standards (3.04 health workers per 1,000 population), a very low number of health facilities providing Comprehensive emergency obstetric and newborn care (CEmONC) (9), and a high maternal mortality ratio (412 per 100,000 live births). According to the 2023-2024 Jimma Zone Health Office District health information system 2(DHIS2) administrative report, 57% of women in Dedo received early antenatal care (eANC) visits before 12 weeks, 99% were tested for syphilis, 68% attended four ANC visits, and 28% attended eight ANC visits. In Manna, 68% of women received eANC visits before 12 weeks, 91% were tested for syphilis, 89% attended four ANC visits, and 25% attended eight ANC visits. In Shebe Sombo, 65% of women received eANC visits before 12 weeks, 81% were tested for syphilis, 82% attended four ANC visits, and 14% attended eight ANC visits. These three districts were selected from 21 districts in Jimma Zone after confirming that none of them had any current initiatives aimed at increasing the use of maternal health services[22].

Design

A three-arm, parallel-grouped, cluster randomized controlled trial with 1:1 allocation ratio was used to evaluate the effect of mobile phone messaging-based gain- and loss-framed interventions for improving maternal health service uptake. The baseline survey was conducted in March 2023, intervention was rolled out from May 2023 to December 2023, and the endline was conducted in February 2024. The design and methods used for the trial were described in detail in the published protocol and other publications from this trial [21]. This study adhered to the CONSORT (Consolidated Standards of Reporting Trials) guidelines for reporting clinical trials. We followed these guidelines to ensure that our methodology and reporting are transparent and comprehensive. The CONSORT checklist for this study is provided as Supporting Information **(S2 checklist)**. No changes were made to the methods after the commencement of the trial. The

study was conducted as originally planned, with no modifications to the eligibility criteria, intervention protocols, outcome measures, or data collection methods.

Participants

Twenty-one kebeles (clusters) were randomly selected from the three districts identified forthe study. Pregnant women with a gestational age (GA) of 16-20 weeks, regardless of their antenatal care status for the current pregnancy, were eligible for the trial if they or a family member owned a mobile phone, could provide written informed consent, and had lived in the selected Kebeles for more than six months. Pregnant women or their family members who were unable to read mobile phone messages or planned to relocate from the study cluster during the follow-up period were excluded from the intervention. Baseline data were collected from primiparous and multiparous women who had at least one child before the current pregnancy, ensuring that the baseline reflected their previous childbirth experiences.There were no changes to the trial outcomes or methods, including eligibility criteria, after the trial commenced.

Intervention development

We developed the intervention messages and the messaging system, which automatically dispatched these messages. Participants in the intervention received a total of 39 gain-framed or loss-framed messages over the eight-month period at a frequency of 1–2 messages per week. The messages were developed based on the concept of message framing, with half of them framed as gain messages—emphasizing the benefits of maternal and newborn health care practices—and the other half as loss messages—emphasizing the consequences of not utilizing maternal health services and practicing newborn care. These messages were aligned with specific aspects of the intervention that directly impact maternal and newborn health. They were developed based on evidence-based recommendations and tailored to the health behaviors targeted in the intervention.The content of the messages was evaluated by mothers and experts before being used in the intervention.

Initially, 120 messages (60 in each frame) were drafted in English, each within a 160-character limit, based on Ethiopian Ministry of Health guidelines and World Health Organization recommendations on maternal and newborn health care services. After discussion with the research team, 32 messages (16 from each frame) were discarded. The remaining 88 messages

were translated into Afan Oromo and used for content evaluation. Mothers evaluated these 88 messages through a card sorting activity, and 10 messages (5 from each frame) that were not well understood were discarded. After the card sorting activity, 8 experts assessed the remaining 78 messages (39 from each frame) using 10 statements. The experts suggested modifications for 14 messages (13 loss-framed and 1 gain-framed), and all of these messages were revised based on their suggestions. Finally, 39 messages for each frame were retained, covering ten thematic areas: nutrition, lifestyle changes, danger signs, birth preparedness and complication readiness, breastfeeding practices, labor, newborn care, immunization, and the utilization of antenatal and postnatal care.

A system to manage the schedule and dispatch the messages automatically was designed locally by software programmers. Messages were tailored for pregnant women and sent three times a week, for a total of 39 messages per person. The short code was named 'Ulfa Mijuu,' meaning 'Safe Pregnancy,' to enhance recognition, memorability, and serve as a call to action. The software program features an interface for entering participants' names, weeks of gestation, mobile numbers, message types, message categories, language preferences, and for generating reports and feedback. Although the system was designed with an interface that allows midwives or other health workers to provide feedback on whether pregnant women received the advised services, this feature was not utilized in this intervention due to resource constraints.

The intervention was rolled out from May 2023 to December 2023. The recruitment of intervention participants was conducted from March 1 to March 14, 2024, and the intervention follow-up took place from May 2023 to December 2023**.** During this period, messages were delivered to participants according to their assigned group (gain-framed, loss-framed, or control). Both the gain-framed and loss-framed interventions utilized the same platform and target audience. While the gain-framed messages emphasized benefits and the loss-framed messages highlighted risks, the consistent delivery method allowed us to isolate the effects of message framing on service uptake.

Gain-framed message

Automated gain-framed messages were delivered to the women in this group. These messages were personalized based on each woman's gestational age (GA), expected delivery date (EDD), and postnatal timeline and were sent from the point of ANC enrolment through the end of the

postnatal period to the designated phone numbers. Additionally, women in this group received the standard maternal care offered at the health facilities. A summary of message types and frequencies is provided in Supporting Information (S3 Messages)

Loss-framed messages

Automated loss-framed messages were delivered to the women in this group. These messages were personalized based on each woman's gestational age, expected delivery date, and postnatal timeline and were sent from the point of ANC enrolment through the end of the postnatal period to the designated phone numbers. Additionally, women in this group received the standard maternal care offered at the health facilities (S2 Messages).

Control arm

Participants in this arm received the usual ANC and PNC services at health facilities. According to the latest ANC guidelines, pregnant mothers are recommended to have eight ANC visits and three PNC visits. These visits should include counseling on lifestyle changes, recognizing danger signs and symptoms, birth preparedness, and readiness for complications, as well as other topics like family planning, cord care, bathing, breastfeeding, child immunization, and cervical screening [23].

Sample size determination

We used methods proposed by Hooper and Bourke for sample size calculation [24] assuming a simple parallel group with a baseline outcome assessment and one follow-up assessment.

Three trial arms (gain-framed, loss-framed, and control) were included in the study. The sample size, based on individual randomization, was calculated using the following assumptions: for P1, the proportion of women attending ANC4+ among the control arm was 23.27% [25], the power (1-β) was set at 0.8, and the two-sided alpha was 0.025. The effect size/absolute difference in the proportions of ANC4+ > 4 was 0.15, and Zα/2 was adjusted for multiple comparisons using a Bonferroni correction =2.24. The initial sample size (no) was calculated as follows:

$$no = \frac{(Z\alpha/2 + Z\beta/2) \cdot p1(1-p1) + p2(1-p2)}{(p1-p2)^2} = 175$$

Two design effects were used to inflate the sample size under individual randomization, considering the intracluster correlation coefficient (ICC) within each period and the ICC between periods. The design effect (dc) due to cluster randomization was calculated by considering the following parameters: within-period ICC (p) of 0.003 [26] and number of clusters per arm (n ×dc×dr)÷m= 7. We calculated the design effect as dc=1+(m−1)ρ = 1.12. The design effect (dr) due to repeated assessments was calculated using the following assumptions: within-period ICC and a cluster (0.003), autocorrelation coefficient (π) of 0.8, accounting for a 20% decrease in correlation from within to between different periods [27], and correlation between sample means from the same cluster at different times (r) determined as r=mpΠ/1+(m-1)p. The design effect resulting from repeated assessments was calculated as dr=(1-r2) =0.996. To obtain the final sample size per arm, we multiplied the sample size for individual randomization with both design effects as (no×dc×dr) = 188. Considering a 4% loss to follow-up, the final sample size for each arm was 196. This resulted in 28 women per health post for a total sample size of 588. No formal stopping guidelines were established for this trial. The study was designed to run until the planned end date, and it was completed as scheduled.

Randomization

Random Sequence Generation

Random selection was used to choose six primary healthcare units (two from each district), with the health post (clusters) within these units serving as the randomization units for the trial. Clusters were randomized using simple randomization with a 1:1 allocation ratio. From 30 eligible kebeles, 21 were randomly selected for the trial. The research team designated the gain frame group as Arm 1, the loss frame group as Arm 2, and the control arm as Arm 3 before the randomization.

Allocation concealment

During the cluster randomization process, we created an alphabetically ordered list of the 21 clusters, numbered from 1 to 21. Random numbers were generated using Microsoft Excel, and the clusters were arranged in ascending order based on these random numbers. The first 7 clusters were allocated to Arm 1, the next 7 to Arm 2, and the remaining 7 to Arm 3, ensuring random allocation. Additionally, allocation concealment was maintained during participant

recruitment. Midwives responsible for recruiting participants were unaware of the cluster allocation. Participants were also not informed about the specific allocation group they belonged to at the time of recruitment. This approach minimized the risk of selection bias, as neither the recruiters nor the participants had any information about the intervention group at recruitment . However, given the nature of the intervention, complete blinding was not feasible during the follow-up period, which could introduce some degree of performance or detection bias. The potential effects of unblinding on the results are acknowledged as a limitation, as participants' awareness of receiving an intervention might have influenced their healthcare-seeking behaviour [28].

Blinding

Clusters were randomly assigned to Arm 1, Arm 2, or Arm 3 by a statistician who was unaware of the group assignments. Within the randomized clusters, midwives, supported by local volunteers, conducted home-to-home screenings to assess pregnant women's eligibility. Gestational age (GA) was determined using either the last menstrual period (LMP) or, if LMP was unknown, fundal height measurement. After confirming eligibility, a list of eligible pregnant women was prepared for each cluster, and 28 participants were randomly selected for the intervention. Pregnant women were enrolled after providing informed consent, sharing a phone number, and agreeing to receive text messages on their or their family's phone throughout the intervention period. The interviewers who collected the data were blinded to the intervention assignments; however, blinding the participants was not feasible due to the nature of the intervention.

Outcomes

Primary Outcomes

The primary outcomes were antenatal care, institutional delivery and postnatal service utilization] . These were measured at an individual level and referred to mothers who had received at least eight ANC follow-up visits, delivery at a health facility, and attended PNC follow-up visits with health professionals within seven days of delivery

Secondary outcomes

The secondary outcomes were measured at the individual level and included knowledge about maternal health care, attitude and self-efficacy toward maternal health care practice, and maternal morbidity. Maternal health care knowledge is the extent of understanding about maternal health, including pregnancy, labor, delivery, and postpartum care.It was assessed using a set of 16 questions covering various aspects of pregnancy, labor, delivery, and the postpartum period. Correct responses indicated a stronger understanding, and the total knowledge score was calculated by summing the correct answers, with a higher score reflecting greater overall knowledge.

Attitude toward maternal health care practices is the degree of positive or negative perception toward maternal care utilization. It was measured using 10 items on a 5-point Likert scale, where 1 represented 'strongly disagree' and 5 represented 'strongly agree.' Negatively worded items were reverse-coded, and the combined scores were analyzed, with higher scores indicating a more positive attitude toward maternal care utilization.

Self-efficacy in maternal care refers to an individual's confidence in their ability to perform maternal care practices. It was assessed using 10 items on a 5-point Likert scale, where 1 signified 'strongly disagree' and 5 signified 'strongly agree.' After reverse-coding negatively phrased items, the total score was analyzed, with higher scores reflecting increased self-efficacy in practicing maternal care. .

Maternal Morbidity (Illness) is the occurrence of maternal health complications during pregnancy, labor, or postpartum. Assessed based on self-reported experiences of illness or complications, including preeclampsia, postpartum hemorrhage, infections (e.g., puerperal sepsis, UTIs), and anemia. The presence or absence of maternal morbidity was recorded, with frequency analysis for comparison.

Internal consistency of the main constructs was examined using Cronbach's alpha, and all constructs were found to have adequate Cronbach alpha coefficient values. Cronbach's alpha coefficient values for knowledge, attitude, and self-efficacy were 0.81, 0.92, and 0.86 respectively (Table 1) [29].

Table 1, Measurement Scales for Secondary Outcomes in Maternal Health Care

Construct	Number of Items	Scale	Example Items	Cronbach's Alpha
Knowledge	16	Correct/Incorrect	Severe headache is a danger sign during pregnancy	0.81
Attitude	10	5-point Likert (1 = Strongly Disagree, 5 = Strongly Agree)	Antenatal care visits are essential for a healthy pregnancy."	0.92
Self-Efficacy	10	5-point Likert (1 = Strongly Disagree, 5 = Strongly Agree)	I feel confident in recognizing pregnancy complications.	0.86

Changes to Trial Outcomes

There were no changes to the trial outcomes after the commencement of the trial. The trial fully followed the protocol with all the elements such as the study setting, design, inclusion criteria, intervention implementation, control group conditions, and sample size all following the initial plan.

Data collection procedure and management

Data were collected at two intervals: baseline in March 2023 and endline in February 2024. Both phases utilized interviewer-administered structured questionnaires. Baseline data were collected using a paper-based method, while endline data were collected using the Kobo toolkit. The English version of the questionnaire was translated into Afan Oromo and then back into English by an independent language expert to verify accuracy. The questionnaire included sections on sociodemographic factors, obstetric history, information sources, text message usage, maternal health service utilization, knowledge of maternal health services and danger signs, attitudes toward maternal health services, self-efficacy, and maternal morbidity. A pre-test conducted on 5% of the sample outside the study clusters led to relevant modifications. Records of participants lost to follow-up, including reasons and pregnancy outcomes, were maintained.

Ten data collectors with relevant health-related college degrees and experience were recruited, along with two public health experts to supervise the data collection. Training for data collectors and supervisors lasted two days and covered the questionnaire, measurement methods, ethical

considerations, and data collection tools. Both data collectors and supervisors were blinded to cluster assignments. Supervisors ensured data completeness during collection, and any missing items were corrected by data collectors. Baseline data were entered twice to manage entry errors, while endline data were obtained from KoBo Toolbox. Confidentiality was maintained throughout, with personal identifiers removed from the database. Paper questionnaires were securely stored, and electronic data were kept on a password-protected computer accessible only to the research team.

Data Analysis

The data were verified for completeness during data collection, coded, and entered into EpiData 4.6 using the double-data entry method. Endline data were retrieved from KoBo Toolbox. For data analysis, we used Statistical Package for the Social Sciences (SPSS) version 27. Data cleaning was performed by running simple frequency distributions to check for internal consistency. We used frequencies and percentages to summarize descriptive statistics for categorical variables, while means and standard deviations were used for continuous variables Per Protocol (PP) Analysis approach was used to evaluate the effectiveness of the SMS-based interventions on maternal health services utilization, including only participants who adhered to the intervention protocol as intended.. Multiple imputations based on chained equations were conducted for missing data at both baseline and follow-up. The effects of the intervention on primary outcomes were assessed using a Generalized Linear Mixed Model (GLMM) to account for clustering and participant-level intracluster correlation. Secondary outcomes were analyzed using a Linear Mixed Model (LMM). Both models included a random intercept for kebele, with ICC calculated for primary and secondary outcomes. Fixed effects for time and intervention by time were included to control for baseline differences. A secondary analysis of complete cases was performed as a sensitivity check, and statistical significance was determined with a p-value less than 0.05, adjusted using the sequential Bonferroni method.

In addition to the main analysis, an ancillary analysis evaluated changes in perceived barriers to maternal health service utilization and improvements in birth preparedness practices between baseline and endline across study arms. Data on these indicators were collected at both time points to assess trends over time. Descriptive statistics, including percentages and frequencies, were used to summarize the findings.

Results

Participant flow

The recruitment of intervention participants were carried out in March 2023. Intervention follow-up were done from May 2023 to December 2023. The intervention was successfully delivered to 14 randomly selected kebeles, each of which received their designated treatment and was included in the final analysis. No criteria for early stopping were established for this trial; the trial was conducted as planned and completed its full course. At baseline, the average cluster sample size was 196 across all three study arms. By the endline survey, data had been collected from 572 participants: 187 (31.8%) in the gain-framed arm, 192 (32.7%) in the loss-framed arm, and 193 (32.8%) in the control arm. Initially, 588 participants were surveyed at baseline. However, 16 participants (gain-framed = 9, loss-framed = 4, control = 3) were excluded at follow-up due to maternal deaths (n = 3) or relocation (n = 13). Consequently, complete outcome data were available for 572 participants (97.3%) at the endline assessment. The follow-up of study participants throughout the trial is presented according to the Consolidated Standards of Reporting Trials (CONSORT) guidelines in the flowchart (Figure 1).

Baseline characteristics of Participants

The study included 588 participants, equally distributed across the Gain-framed, Loss-framed, and Control arms. The majority of participants were aged 20–34 years (90.5%), identified as Muslim (91.7%), and had attained primary education (51.4%). Nearly all were married (94%) and housewives (88.8%). Most of their husbands had primary education (65.5%) and worked as farmers (81%).

Participants primarily resided in rural areas (55.1%), with an average of 2.58 literate family members. The average parity among participants was 3.2 children. Notable differences were observed among the study arms regarding maternal age, education level, marital status, and husbands' occupations, indicating variations in demographic and socioeconomic characteristics. (Table 2).

Table 2. Baseline characteristics of participants Jimma Zone, Ethiopia

Participant characteristics	Gain framed (n=196)	Loss frame (n=196)	Control(n=196)	Overall (n=196)
Maternal age in years				
15 – 19	8(4.1)	5 (2.6)	11 (5.6)	24(4.1)
20 – 34	170 (86.7)	183 (93.4)	179 (91.3)	532(90.5)
35 – 44	18 (9.2)	8 (4.1)	6 (3.1)	32(5.4)
**Religion**				
Muslim	186 (94.9)	168 (85.7)	185 (94.4)	539(91.7)
Others[a]	10 (5.1)	28 (14.3)	11 (5.6)	49(8.3)
**Education status**				
No formal education	45 (23)	49 (25)	53 (27)	147(25)
Can read and write	11 (5.6)	17 (8.7)	10 (51)	38(6.5)
Primary education	112 (57.1)	92 (46.9)	98 (50)	302(51.4)
Secondary education	16 (8.2)	23 (11.7)	22 (11.2)	61(10.4)
Collage and above	12 (6.1)	15 (7.7)	13 (6.6)	40(6.8)
**Marital Status**				
Married	196 (100)	169 (86.2)	188 (95.9)	553(94)
Other[b]	0 (0)	27 (13.8)	8 (4.1)	35(6)
**Occupation**				
Housewife	186 (94.9)	159 (81.1)	177 (90.3)	522(88.8)
Merchant	3 (1.5)	16(8.2)	14 (7.1)	33(5.6)
Government employee	6 (3.1)	12 (6.1)	0 (0)	18(3.1)
Other[c]	1 (0.5)	9 (4.6)	5 (2.6)	15(2.6)
**Husband Education status**				
No formal education	13 (6.6)	16 (9.5)	33 (17.6)	62(11.2)
Can read and write	11 (5.6)	7 (4.1)	15 (8)	33(6.0)
Primary education	146 (74.5)	112 (66.3)	104 (55.3)	362(65.5)
Secondary education	14 (7.1)	18 (10.7)	31 (16.5)	63(11.4)
Collage and above	12 (6.1)	16 (9.5)	10 (2.7)	33(6.0)

**Husband Occupation**				
Farmer	170 (86.7)	114 (67.5)	164 (87.2)	448(81.0)
Merchant	11 (5.6)	30 (17.8)	13 (6.9)	54(9.8)
Government employee	10 (5.1)	17 (10.1)	3 (1.6)	30(5.4)
Other[d]	5 (2.6)	8 (4.7)	8 (4.3)	21(3.8)
**Cluster level**				
Rural resident (%)	110 (56)	98 (50)	117 (60)	325(100)
Pregnant women percluster (Mean ± SD)	272 ± 92	255 ± 90.6	240 ± 104.7	256± 96.7
Family members able to read (Mean ± SD)	2.35 ± 0.62	2.37 ± 0.45	3 ± 0.62	2.58±0.64
Parity (Mean ± SD)	3.27 ± 0.36	3.16 ± 0.60	3.04 ± 0.50	3.2± 0.50

[a] 30 Orthodox, 2 catholic, 17 protestant: [b] 33 Single, 1 divorced, 1 separated: [c] 11 daily laborer , 4 students: [d] 9 daily laborer , 6 merchants, 4 students, 3 non-government organization.

Effectiveness of Interventions on Improving antenatal care (ANC), Skilled birth attendance (SBA), and postnatal care (PNC)

Antenatal Care (ANC): Antenatal care (ANC) utilization showed a marked increase in the intervention arms. In the gain-framed arm, ANC utilization rose from 85.7% at baseline to 97.3% at the endline. Similarly, in the loss-framed arm, it increased from 90.8% to 98.4%, while in the control arm, it decreased from 85.7% to 79.8%. The proportion of ANC utilization among participants in the gain-framed arm (OR: 2.6; 95% CI: 1.30 to 5.14) was significantly higher than among those in the control arm. The proportion of ANC utilization among participants in the loss-framed arm (OR: 4.1; 95% CI: 1.99 to 8.52) was also significantly higher than among those in the control arm (Table 3).

Institutional Births: Institutional births increased significantly in the intervention arms. The gain-framed arm showed an increase from 68.4% at baseline to 94.7% at endline, and the loss-framed arm from 73.0% to 99.0%. In contrast, the control arm showed a decrease from 82.7% to 57.0%. The proportion of institutional births among participants in the loss-framed arm (OR: 3.3; 95% CI: 1.17 to 9.32) was significantly higher than among those in the control arm (Table 3).

Postnatal Care (PNC): Postnatal care utilization improved as well, with the gain-framed arm increasing from 65.3% at baseline to 78.1% at endline, and the loss-framed group from 58.7% to 75.8%. The control arm saw a decrease from 57.7% to 45.6%. The proportion of PNC utilization among participants in the gain-framed arm was 2.6 times higher (OR: 2.6; 95% CI: 1.36 to 5.06) than those in the control arm. The proportion of PNC utilization among participants in the loss-framed arm was also significantly higher (OR: 2.0; 95% CI: 1.02 to 3.73) than among those in the control arm (Table 3).

Table 3. **Effectiveness of interventions on improving maternal health service utilization (ANC, SBA and PNC)**

	Gain frame n(%)	Loss frame n(%)	Control n(%)	Overall n(%)
**Antenatal care**				
Pre-intervention use	168(85.7)	178(90.8)	168(85.7)	514(87.4)
Post-intervention use	182(97.3)	189(98.4)	148(79.8)	519(90.7)
OR (95% CI)	2.6 (1.30 to 5.14)	4.1 (1.99 to 8.52)	1	
ICC	0.22			
**Institutional births**				
Pre-intervention use	134(68.4)	143(73.0)	162(82.7)	439(74.7)
Post-intervention use	177(94.7)	190(99.0)	110(57.0)	477(83.4)
OR (95% CI)	2.5 (0.89 to 7.03)	3.3 (1.17 to 9.32)	1	
ICC	0.48			
**Postnatal care**				
Pre-intervention use	128(65.3)	115(58.7)	113(57.7)	356(60.5)
Post-intervention use	146(78.1)	144(75.8)	88(45.6)	378(66.3)
OR (95% CI)	2.6 (1.36 to 5.06)	2.0 (1.02 to 3.73)	1	
ICC	0.23			

Intervention effect on secondary outcomes

Knowledge about Maternal Health Services (MHS): The intervention significantly improved participants' knowledge about MHS in both the gain-framed and loss-framed arms compared to the control arm. At baseline, the mean knowledge scores were 36.13 (±4.05) for the gain-framed arm, 38.71 (±3.17) for the loss-framed arm, and 32.88 (±5.07) for the control arm. At the endline, these scores increased to 41.27 (±6.89) for the gain-framed arm and 42.45 (±5.89) for the loss-framed arm, while the control arm saw a modest increase to 33.67 (±3.95). After adjustment for baseline knowledge scores and potential confounders, both intervention arms showed a statistically significant change in knowldge towards maternal health services. The adjusted mean difference (aMD) in knowledge scores was 5.21 (95% CI: 2.89 to 14.87, $p < 0.05$) in the gain-framed arm and 7.55 (95% CI: 5.21 to 21.99, $p < 0.05$) in the loss-framed arm, relative to the control group (Table 4).

Attitude toward MHS: Participants' attitudes toward MHS improved significantly in both the gain-framed and loss-framed arms. The baseline attitude scores were 42.15 (±3.44) for the gain-framed arm, 39.38 (±5.64) for the loss-framed arm, and 36.47 (±4.80) for the control arm. By the endline, the scores increased to 45.62 (±5.28) for the gain-framed arm, 42.21 (±8.00) for the loss-framed arm, and 37.62 (±8.02) for the control arm. After adjustment for baseline attitude scores and potential confounders, both intervention arms showed a statistically significant change in attitude towards maternal health services. The adjusted mean difference (aMD) in attitude score was 6.72 (95% CI: 3.21 to 15.68, $p < 0.05$) in the gain-framed arm and 3.38 (95% CI: 1.42 to 8.21, $p < 0.05$) in the loss-framed arm, compared to the control arm. (Table 4).

Self-efficacy toward MHS: The intervention arms exhibited improvements in self-efficacy toward MHS, though the changes were not as pronounced as those in knowledge and attitude. The baseline self-efficacy scores were 43.46 (±3.27) for the gain-framed arm, 42.24 (±3.85) for the loss-framed arm, and 42.33 (±4.25) for the control arm. At the endline, the scores were 45.95 (±6.60) for the gain-framed arm, 44.33 (±5.32) for the loss-framed arm, and 43.62 (±6.99) for the control arm. After adjusting for baseline self-efficacy scores and potential confounders, no statistically significant self-efficacy improvement was seen in either the gain-framed arm (aMD:

1.87; 95% CI: 0.69 to 4.79) or the loss-framed arm (aMD: 1.12; 95% CI: 0.32 to 3.38), relative to the control arm (Table 4).

Maternal Morbidity: There was a notable reduction in maternal morbidity in the intervention arms. At baseline, maternal morbidity rates were 23.9% (n=160) in the gain-framed arm, 19.7% (n=132) in the loss-framed arm, and 20.1% (n=135) in the control arm. By the endline, these rates dropped to 7.5% (n=50) in the gain-framed arm and 9.6% (n=64) in the loss-framed arm, whereas the control arm had a rate of 19.3% (n=129). There was a significant reduction in maternal morbidity in the gain-framed arm (OR: 1.9; 95% CI, 1.09 to 3.38) and the loss-framed arm (OR: 2.3; 95% CI, 1.31 to 4.06) compared to the control arm (Table 4).

Table 4. Effectiveness of interventions on secondary outcomes (Knowledge about maternal health service, and danger signs during pregnancy, labor, and delivery), Attitude toward maternal health services, Self-efficacy toward maternal health service utilization, and Maternal morbidity)

	Gain frame<br>n(%)	Loss frame<br>n(%)	Control<br>n(%)	Overall<br>n(%)
Knowledge about MHS				
Pre-intervention	36.13± 4.05	38.71± 3.17	32.88± 5.07	107.72± 12.29
Post-intervention	41.27± 6.89	42.45± 5.89	33.67± 3.95	117.39± 16.74
aMD (95% CI)	5.21 (2.89 to 14.87)	7.55 (5.21 to 21.99)	1	
ICC	0.21			
Attitude toward MHS				
Pre-intervention	42.15 ± 3.44	39.38 ± 5.64	36.47 ± 4.80	118.00 ± 13.88
Post-intervention	45.62 ± 5.28	42.21 ± 8.00	37.62 ± 8.02	125.45 ± 21.30
aMD (95% CI)	6.72 (3.21 to 15.68)	3.38 (1.42 to 8.21)	1	
ICC	0.12			
Self-efficacy toward MHS				
Pre-intervention	43.46± 3.27	42.24± 3.85	42.33± 4.25	43.46± 3.27
Post-intervention	45.95± 6.60	44.33± 5.32	43.62± 6.99	45.95± 6.60
aMD (95% CI)	1.87 (0.69 to 4.79)	1.12 (0.32 to 3.38)	1	
ICC	0.18			

Maternal Morbidity				
Pre-intervention	160(23.9)	132(19.7)	135(20.1)	427(63.7)
Post-intervention	50(7.5)	64(9.6)	129(19.3)	243(36.3)
Odds ratio (95% CI)	1.9 (1.09 to 3.38)	2.3 (1.31 to 4.06)	1	
ICC	0.21			

Ancillary analysis

Experience of barriers across study arms

Figure 2 shows a significant reduction in various barriers to accessing maternal health services from baseline to endline. At baseline, 70.1% of respondents did not think it was necessary to seek care, which decreased to 29.9% by the endline. The most notable improvement was in the perception of poor-quality services, which saw a drastic reduction from 97.1% to 2.9%. The use of home remedies also declined from 75% to 25%. Awareness of where to seek care improved, with the percentage of respondents not knowing where to go decreasing from 59% to 41%. Time constraints were less of a concern, dropping from 66.4% to 33.6%. Additionally, the issues of long wait times and inconvenient hours were almost entirely resolved, decreasing from 98.6% to 1.4% and 95.8% to 4.2%, respectively. These changes indicate a substantial improvement in accessibility and perceived quality of health services over the period.

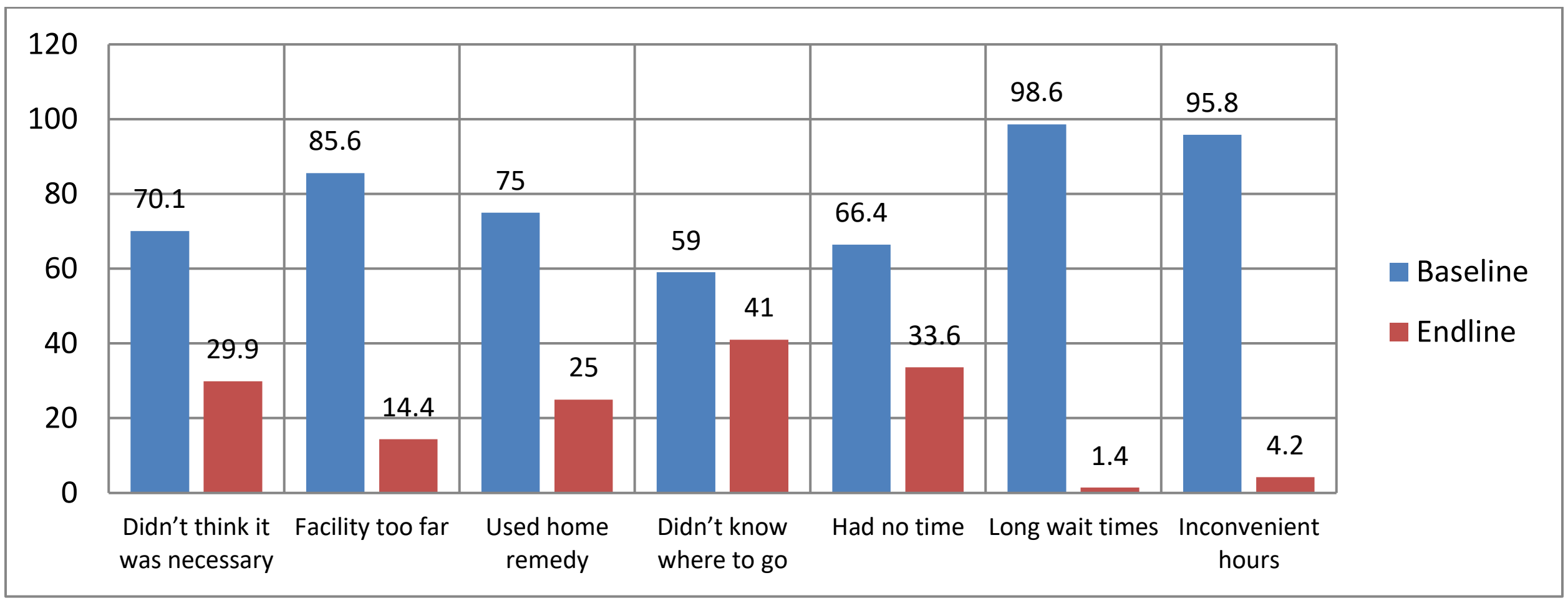

Figure 2. Factors Affecting Maternal Health Service Utilization

Experiences of Birth preparedness across study arms

There were improvements in several maternal birth preparedness indicators from baseline to endline. The percentage of respondents planning to give birth at a health facility (HF) increased from 59.5% at baseline to 70.5% at the endline. Those making arrangements for childbirth rose from 44.9% to 55.1%. The percentage of respondents saving money for delivery saw a

significant increase from 72.7% to 94.6%. Similarly, organizing transport improved from 43.8% to 56.2%, and identifying a skilled delivery attendant rose from 66.2% to 73.4% (Figure 3).

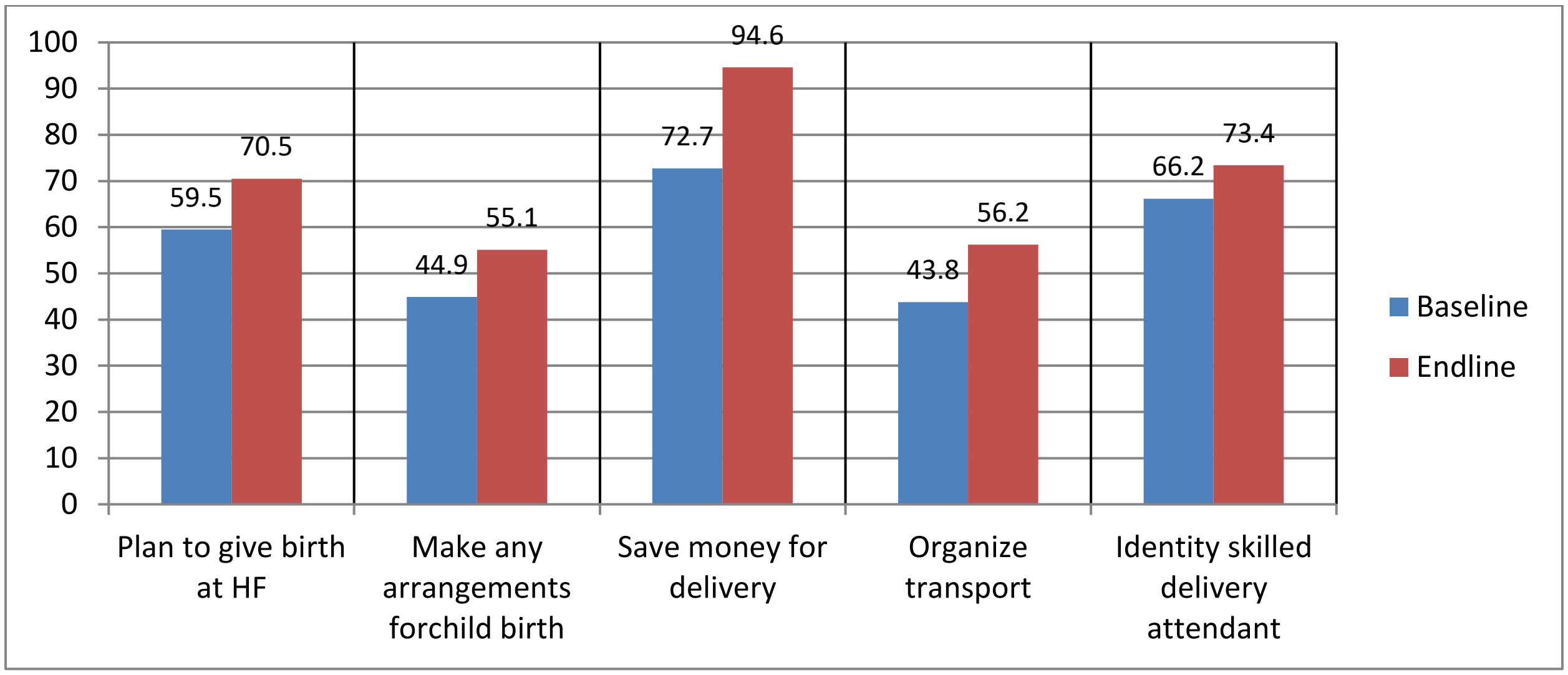

Figure 3. Experiences of Birth Preparedness across study arms

Harms or unintended effects

In the study, participants were monitored for any potential harms or unintended effects from the mobile phone messaging interventions. The gain-framed group reported mild anxiety in 1.5% of participants, while the loss-framed group experienced confusion in 2.0% of participants, with one individual opting out of the messages. No significant adverse events were reported, and the observed effects did not impact the study outcomes.

Discussion

The study evaluated the effect of mobile phone-based gain- and loss-framed messaging interventions on antenatal care (ANC), skilled birth attendance (SBA), postnatal care (PNC), knowledge, attitudes, self-efficacy toward maternal health services (MHS), and maternal morbidity. The intervention had a significant impact on the utilization of maternal healthcare services, including ANC, institutional delivery, and PNC.

ANC use was higher in both the gain- and loss-framed groups compared to the control group but did not differ significantly between the two intervention arms. Institutional births increased significantly in the loss-framed arm, with participants in this group being significantly more likely to give birth in a health facility compared to those in the control arm. PNC utilization was also significantly higher in both the gain- and loss-framed groups compared to the control arm, indicating the overall success of the intervention in promoting the use of maternal health services. Similarly, a study conducted in Iran assessing the effect of message framing on breastfeeding self-efficacy among nulliparous women found that both gain-framed and loss-framed messages increased self-efficacy at weeks four and eight after delivery in the intervention groups. However, no significant difference was observed between gain-framed and loss-framed messages [30]. Likewise, a study conducted in the United States found that gain-framed messages were more effective in increasing intentions to use condoms, while loss-framed messages were more effective in reducing intentions to have sex with higher-risk partners [31]. This study reveals that both gain-framed and loss-framed messaging strategies were effective in significantly increasing the likelihood of maternal health serice utilization. The decline in maternal health service utilization in the control arm can be attributed to the presence of health interventions or activities, such as campaigns, at baseline, which significantly increased service uptake. However, in the absence of such interventions at endline, utilization declined because the initial momentum generated by external efforts was no longer sustained. This suggests that without continuous reinforcement through targeted interventions, maternal health service use may revert to previous levels. Therefore, a long-term engagement strategy is essential to sustain improvements in maternal health outcomes.

The current study showed that the intervention significantly improved participants' knowledge of MHS. Our study also found that attitude scores improved in the gain-framed and loss-framed arms. Similarly, a study by Divdar et al. reported significant improvements in oral health knowledge, attitudes, intentions, self-efficacy, and practices among the intervention group compared to the control group, although no significant difference was observed between gain- and loss-framed messages [18].

In another trial assessing the effectiveness of a message-framing intervention on complementary feeding-related behaviors among mothers with infants aged 4–8 months, both gain- and loss-framed messages equally increased knowledge, self-efficacy, and adherence to appropriate complementary feeding onset, with greater improvements than in the control group [20]. Similarly, a study examining the impact of message framing on oral health behaviors found that both gain- and loss-framed interventions effectively improved students' knowledge of dental hygiene [32]. Another study focused on youth participating in a nutrition program found that both gain- and loss-framed messages improved knowledge about nutrition and physical activity [33].

There was a notable reduction in maternal morbidity in both gain and loss framed the arms, while the control arm remained relatively unchanged. Similarly, in on controlled trial, both the framing approaches improved the state of health but with more suppression of complications marked in the loss-framed condition, which demonstrates that the sense of urgency from loss framing is capable of differentially affecting the outcomes[34]. These results suggest that both gain and loss-framed messages can significantly reduce maternal morbidity. The reduction was attributed to increased antenatal care attendance, better adherence to recommended health behaviors, and more frequent facility-based deliveries.

The results of this study indicate that both gain- and loss-framed message strategies increased maternal health service utilization, knowledge, attitudes, and morbidity reduction, though their effects varied across specific maternal health practices. Loss-framed messages were particularly effective in increasing ANC visits and institutional deliveries, while gain-framed messages were more effective in promoting PNC utilization. However, neither framing significantly influenced self-efficacy. Additionally, skilled birth attendance (SBA) did not show significant improvement in the gain-framed group. These findings highlight the nuanced impact of message framing on

maternal health interventions and suggest that tailored strategies may be necessary to optimize outcomes along the continuum of care. Similarly, a meta-analysis of 53 studies found that loss-framed appeals were more persuasive in messages encouraging disease detection behaviors and gain-framed messages are often more effective for promoting behaviors associated with achieving positive health outcomes, such as physical activity or healthy eating In terms of disease prevention, gain-framed messaging highlights the promise of a safer, more certain outcome, making it more effective than loss-framed messaging [35].

Implications

Our study contributes unique findings to the literature on message framing, particularly in the context of maternal health services in Ethiopia. The use of individually tailored messages delivered via mobile phones demonstrated significant improvements in maternal health service utilization, knowledge, attitude, and self-efficacy. Program managers and policymakers should consider incorporating such interventions into health communication strategies to enhance maternal health outcomes. Further research is needed to explore the underlying factors contributing to the varying effectiveness of gain and loss-framed messages across different contexts and health behaviors.

Strengths and Limitations

This study's strengths include the use of a cluster randomized controlled trial (RCT) design, which minimized the risk of information contamination and the employment of allocation concealment during cluster enrollment. The automated messaging system ensured the timely delivery of interventions at a minimal cost. However, the study was limited by its inability to generalize findings to individuals without mobile phones, potential recall bias, and the lack of a monitoring interface for the automated system. Future studies should consider evaluating the implementation outcomes and cost-effectiveness of such interventions.

Conclusion

The study demonstrates the significant impact of mobile phone messaging-based gain- and loss-framed approaches on maternal health service utilization in Jimma Zone, Ethiopia. Both gain-

framed and loss-framed interventions lined to substantial increases antenatal care (ANC), institutional births, and postnatal care (PNC). Specifically, the gain-framed arm showed improvements in ANC by 11.6%, institutional births by 26.3%, and PNC by 12.8%, while the loss-framed arm increased MHSU by 18.8%, ANC by 7.6%, institutional births by 26%, and PNC by 17.1%. Both interventions significantly enhanced maternal knowledge and attitudes toward maternal health services, although self-efficacy improved to a lesser extent. Maternal morbidity rates decreased notably in the intervention arms, with reductions of 16.4% in the gain-framed group and 10.1% in the loss-framed group, while the control arm remained relatively unchanged. These findings underscore the effectiveness of tailored messaging interventions in resource-limited settings, with gain-framed messages particularly excelling in promoting ANC utilization and institutional births, and loss-framed messages proving similarly effective across various metrics. The significant improvements in maternal health service utilization, knowledge, attitude, and reduction in maternal morbidity highlight the potential of these interventions to enhance maternal health practices in similar contexts. Thus, the study conclusively demonstrates that mobile phone-based gain and loss-framed messaging interventions significantly enhance maternal health service uptake and improve maternal health outcomes in Jimma Zone, Ethiopia.

Ethical consideration

The Jimma University Institutional Review Board approved the study (ref no-JUIH/IRB/316/23). The ethical approval letter is available in Supporting Information **(S4 Ethical Approval)**. Participants gave informed consent before the intervention and assessments. Consent details, including study objectives and participant roles, were explained in the local language. Confidentiality was ensured by encrypting phone numbers, accessible only to server technicians and researchers, with data shared after full de-identification. The Human Participants Research Checklist form that is filled out is included as supporting information **(S5 checklist).**

Authors` Contributions

GB played a key role in the inception, design, data collection, analysis, and drafting of the manuscript. HG contributed to the project by assisting with the inception, design, and data collection, and by providing critical feedback on the manuscript. MG and DA participated in the design, data gathering, and review of the manuscript. ZB was involved in the design, data

collection, and provided a critical review of the manuscript. All authors reviewed and approved the final version of the manuscript.

Conflicts of interests

The authors declare that they do not have competing interests.

Abbreviations

ANC	Antenatal Care
CEmONC	Comprehensive emergency obstetric and newborn care
CI	Confidence interval
CONSORT	Consolidated Standards of Reporting Trails
cRCT	Cluster randomized controlled trial
DE	Design Effect
DHIS2	District Health Information System 2
EDD	Expected date of delivery
eANC	Early antenatal care
GA	Gestational Age
GLMM	Generalized linear mixed model
HF	Health facility
HSTP	Health Sector Transformation Plan
ICC	Interclass correlation
IRB	Institutional Review Board
JUIH	Jimma University Institute of Health
LMM	Linear mixed model
LMP	Last menstrual period
MHSU	Maternal Health Service Utilization
MHS	Maternal health service
OR	Odds ratio
PNC	Postnatal care
RCT	Randomized controlled trial
SDG	Sustainable development goal
SMS	Short message service

SPSS	Software package for social science
VPN	Virtual private network

Acknowledgment

We extend our sincere gratitude to the Jimma Zone Health Office and the Oromia Regional Health Bureau for their invaluable support throughout the design, implementation, and evaluation of this project.

Funding

This research was supported by grants from Jimma University for postgraduate students and additional funding from the Institute of Health, Department of Health, Behavior, and Society. The findings and conclusions are those of the authors and do not necessarily reflect the views of these organizations.

Availability of data and materials

Data used for this analysis can be provided by the authors upon reasonable request to the corresponding author, Gebeyehu Bulcha (gebeyehubulcha@gmail.com).

Consent for publication

Not applicable

References

1. WHO Maternal Health. *Who* **2018**, 1–47.
2. United Nations Population Fund, (UNFPA) Transformative Result: Ending Preventable Maternal Deaths. *United Nations Popul. Fund* **2020**, 1.
3. World Health Organisation Maternal Mortality: The Urgency of a Systemic and Multisectoral Approach in Mitigating Maternal Deaths in Africa. *Anal. Fact Sheet* **2023**, 1–11.
4. Mahmood, M.A.; Mufidah, I.; Scroggs, S.; Siddiqui, A.R.; Raheel, H.; Wibdarminto, K.; Dirgantoro, B.; Vercruyssen, J.; Wahabi, H.A. Root-Cause Analysis of Persistently High Maternal Mortality in a Rural District of Indonesia : Role of Clinical Care Quality and Health Services Organizational Factors. **2018**, *2018*.
5. Mangiaterra, V.; Bucagu, M.; Sabbatucci, F. Maternal Health. *Sustain. Dev. Goals Ser.* **2023**, *Part F2781*, 59–65, doi:10.1007/978-3-031-33851-9_9.
6. United Nations The 17 Goals. *Glob. goals Sustain. Dev.* **2018**, 15–18.
7. Ethiopian Public Health Institute (EPHI) [Ethiopia] and ICF *Ethiopia Mini Demographic and Health Survey 2019: Final Report*; 2021; ISBN 2511127547.
8. FMoH-Ethiopia Health Sector Translaformation Plan II (HSTP II). *Ethiop. Minist. Heal.* **2021**, *25*, 96.
9. Simpson, K.R. Surgeon General's Call to Action to Improve Maternal Health in America. *MCN Am. J. Matern. Nurs.* **2021**, *46*, 184, doi:10.1097/NMC.0000000000000715.
10. Yadav, A.K.; Jena, P.K. Education , Employment , Economic Status and Empowerment : Implications for Maternal Health Care Services Utilization in India. **2020**, doi:10.1002/pa.2259.
11. Bossman, E.; Johansen, M.A.; Zanaboni, P. MHealth Interventions to Reduce Maternal and Child Mortality in Sub-Saharan Africa and Southern Asia: A Systematic Literature Review. *Front. Glob. Women's Heal.* **2022**, *3*, doi:10.3389/fgwh.2022.942146.
12. Coleman, J.; Black, V.; Thorson, A.E.; Eriksen, J. Evaluating the Effect of Maternal MHealth Text Messages on Uptake of Maternal and Child Health Care Services in South Africa: A Multicentre Cohort Intervention Study. *Reprod. Health* **2020**, *17*, 160, doi:10.1186/s12978-020-01017-3.
13. Hall, A.K.; Cole-Lewis, H.; Bernhardt, J.M. Mobile Text Messaging for Health: A Systematic Review of Reviews. *Annu. Rev. Public Health* **2015**, *36*, 393–415, doi:10.1146/annurev-publhealth-031914-122855.
14. Huang, S.; Li, M. Piloting a MHealth Intervention to Improve Newborn Care Awareness among Rural Cambodian Mothers: A Feasibility Study. *BMC Pregnancy Childbirth* **2017**, *17*, 1–7, doi:10.1186/s12884-017-1541-z.
15. Carfora, V.; Catellani, P. The Effect of Persuasive Messages in Promoting Home-Based Physical

Activity During COVID-19 Pandemic. *Front. Psychol.* **2021**, *12*, 1–14, doi:10.3389/fpsyg.2021.644050.

16. Grappi, S.; Bergianti, F.; Gabrielli, V.; Baghi, I. The Effect of Message Framing on Young Adult Consumers' Sustainable Fashion Consumption: The Role of Anticipated Emotions and Perceived Ethicality. *J. Bus. Res.* **2024**, *170*, 114341, doi:https://doi.org/10.1016/j.jbusres.2023.114341.
17. Mikels, J.A.; Young, N.A.; Liu, X.; Stine-Morrow, E.A.L. Getting to the Heart of the Matter in Later Life: The Central Role of Affect in Health Message Framing. *Gerontologist* **2021**, *61*, 756–762, doi:10.1093/geront/gnaa128.
18. Divdar, M.; Araban, M.; Heydarabadi, A.B.; Cheraghian, B.; Stein, L.A.R. Effectiveness of Message-Framing to Improve Oral Health Behaviors and Dental Plaque among Pregnant Women. *Arch. Public Heal.* **2021**, *79*, 1–8, doi:10.1186/s13690-021-00640-1.
19. Zahid, A.; Reicks, M. Gain-Framed Messages Were Related to Higher Motivation Scores for Sugar-Sweetened Beverage Parenting Practices than Loss-Framed Messages. *Nutrients* **2018**, *10*, doi:10.3390/nu10050625.
20. Rafieyan-kopaei, Z.; Fathian-dastgerdi, Z.; Tarrahi, M.J.; Zamani-Alavijeh, F. Effectiveness of Message-Framing Intervention on Complementary Feeding Related Behaviors among Mothers with Infants Aged 4 – 8 Months : A 3-Arm Randomized Controlled Trial. *Ital. J. Pediatr.* **2019**, *45*, 1–13.
21. Bulcha, G.; Abdissa, H.G.; Noll, J.; Sori, D.A.; Koricha, Z.B. Effectiveness of a Mobile Phone Messaging-Based Message Framing Intervention for Improving Maternal Health Service Uptake and Newborn Care Practice in Rural Jimma Zone, Ethiopia: Protocol for a Cluster Randomized Controlled Trial. *JMIR Res. Protoc.* **2024**, *13*, e52395, doi:10.2196/52395.
22. Jimma Zone Health Office *Annual DHIS2 Administrative Report Bulletin*; 2024;
23. Hanson, J.; Global, A.; Cdc, R. National Antenatal Care Guidelines. *Minist. Heal. Rwanda* **2020**, 1–113.
24. Bourke, L.; Hooper, R.; Bourke, L. Cluster Randomised Trials with Repeated Cross Sections : Alternatives to Cluster Randomised Trials with Repeated Cross Sections : Alternatives to Parallel Group Designs. **2015**, doi:10.1136/bmj.h2925.
25. Atnafu, A.; Otto, K.; Herbst, C.H. The Role of MHealth Intervention on Maternal and Child Health Service Delivery: Findings from a Randomized Controlled Field Trial in Rural Ethiopia. *mHealth* **2017**, *3*, 39, doi:10.21037/mhealth.2017.08.04.
26. Taljaard, M.; Donner, A.; Villar, J.; Wojdyla, D.; Velazco, A.; Bataglia, V.; Faundes, A.; Langer, A.; Narváez, A.; Valladares, E.; et al. Intracluster Correlation Coefficients from the 2005 WHO Global Survey on Maternal and Perinatal Health: Implications for Implementation Research.

Paediatr. Perinat. Epidemiol. **2008**, *22*, 117–125, doi:10.1111/j.1365-3016.2007.00901.x.

27. Hooper, R.; Teerenstra, S.; de Hoop, E.; Eldridge, S. Sample Size Calculation for Stepped Wedge and Other Longitudinal Cluster Randomised Trials. *Stat. Med.* **2016**, *35*, 4718–4728, doi:10.1002/sim.7028.
28. Bruce, C.L.; Iflaifel, M.; Montgomery, A.; Ogollah, R.; Sprange, K.; Partlett, C. Choosing and Evaluating Randomisation Methods in Clinical Trials: A Qualitative Study. *Trials* **2024**, *25*, 1–11, doi:10.1186/s13063-024-08005-z.
29. Bulcha, G.; Gutema, H.; Amenu, D.; Birhanu, Z. Maternal Health Service Utilization in the Jimma Zone, Ethiopia: Results from a Baseline Study for Mobile Phone Messaging Interventions. *BMC Pregnancy Childbirth* **2024**, *24*, 1–16, doi:10.1186/s12884-024-06683-w.
30. Kebede, A.S.; Ajayi, I.O.; Arowojoluc, A.O. Effect of Enhanced Reminders on Postnatal Clinic Attendance in Addis Ababa , Ethiopia : A Cluster Randomized Controlled Trial. *Glob. Health Action* **2019**, *12*.
31. Brokowski C, A.M. 乳鼠心肌提取 HHS Public Access. *Physiol. Behav.* **2019**, *176*, 139–148, doi:10.1007/s10508-015-0679-x.The.
32. Divdar, M.; Araban, M.; Heydarabadi, A.B.; Cheraghian, B.; Stein, L.A.R. Effectiveness of Message-Framing to Improve Oral Health Behaviors and Dental Plaque among Pregnant Women. *Arch. Public Heal.* **2021**, *79*, 1–17, doi:10.1186/s13690-021-00640-1.
33. Fetter, D.S.; Dharmar, M.; Lawry-Hall, S.; Pressman, J.; Chapman, J.; Scherr, R.E. The Influence of Gain-Framed and Loss-Framed Health Messages on Nutrition and Physical Activity Knowledge. *Glob. Pediatr. Heal.* **2019**, *6*, doi:10.1177/2333794X19857405.
34. Gao, R.; Guo, H.; Liu, Y.; Pang, Y.; Zhang, X.; Lian, X.; Yu, T.; Zhu, L.; Li, F. The Effects of Message Framing on Self-Management Behavior among People with Type 2 Diabetes: A Randomized Controlled Trial. *Int. J. Nurs. Stud.* **2023**, *142*, 104491, doi:10.1016/j.ijnurstu.2023.104491.
35. O'Keefe, D.J.; Jensen, J.D. The Relative Persuasiveness of Gain-Framed and Loss-Framed Messages for Encouraging Disease Detection Behaviors: A Meta-Analytic Review. *J. Commun.* **2009**, *59*, 296–316, doi:10.1111/j.1460-2466.2009.01417.x.

Figure Click here to access/download;Figure;Fig. 1.pdf

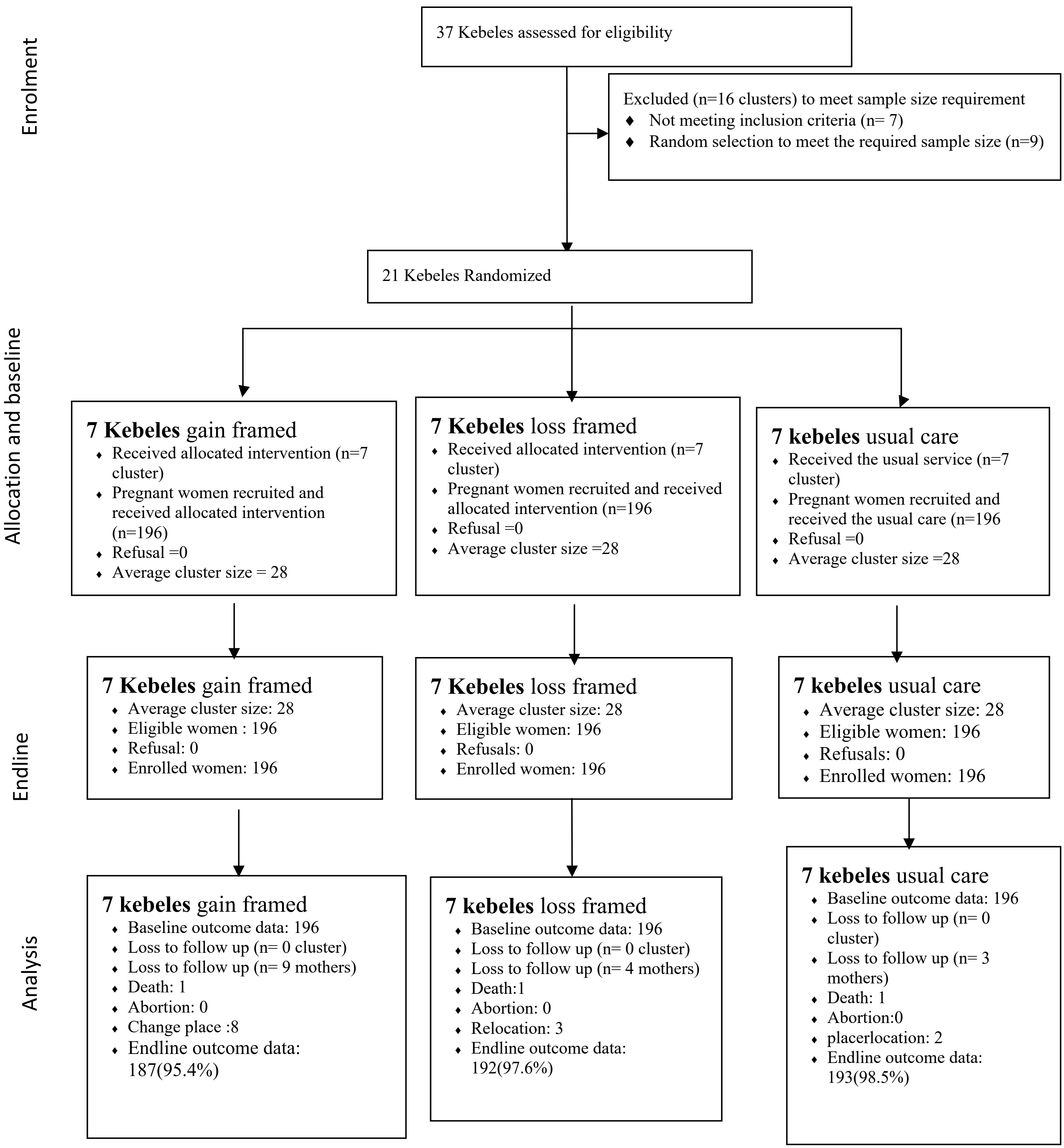

Figure 1. CONSORT Participant flow diagram

Click here to access/download
Supporting Information
S1 Protocol.docx

Click here to access/download
Supporting Information
S2 Checklist.docx

Click here to access/download
Supporting Information
S3 Messages.docx

Click here to access/download
Other
S4 Ethical Approval.pdf

Click here to access/download
Other
S5 Checklist.docx

Click here to access/download
Other
Response.docx
