# Supplementary material for: Evaluating gain and loss-framed mobile messaging to improve maternal health service uptake: A cluster randomized controlled trial in Jimma, Ethiopia
Source: PLoS One. 2026 Oct 26;21(10):e0360474. doi: 10.1371/journal.pone.0360474 (PMC13704815; doi:10.1371/journal.pone.0360474)
Supplement: Supplementary file 5 [file pone.0360474.s002.docx]

CONSORT 2010 Checklist for Reporting a Three-Arm Cluster Randomized Controlled Trial on the Effectiveness of Mobile Phone Messaging with Gain and Loss-Framed Approaches for Enhancing Maternal Health Service Uptake in Rural Jimma Zone, Ethiopia_*_

| **Section/Topic** | **Item No** | **Checklist item** | **Reported on page No**** |
| --- | --- | --- | --- |
| **Title and abstract** | 1a | Identification as a randomised trial in the title | 1 |
|  | 1b | Structured summary of trial design, methods, results, and conclusions _(for_ _specific_ _guidance_ _see_ _CONSORT_ _for_ _abstracts)_ | Abstract page |
| **Introduction**  Background and | 2a | Scientific background and explanation of rationale | 3-5 |
| objectives | 2b | Specific objectives or hypotheses | 2,5 |
| **Methods**  Trial design | 3a | Description of trial design (such as parallel, factorial) including allocation ratio | 6, 7__ |
|  | 3b | Important changes to methods after trial commencement (such as eligibility criteria), with reasons | 6, 7 |
| Participants | 4a | Eligibility criteria for participants | 7 |
|  | 4b | Settings and locations where the data were collected | 6 |
| Interventions | 5 | The interventions for each group with sufficient details to allow replication, including how and when they were | 7-9 |
|  |  | actually administered |  |
| Outcomes | 6a | Completely defined pre-specified primary and secondary outcome measures, including how and when they | 11-12 |
|  |  | were assessed |  |
|  | 6b | Any changes to trial outcomes after the trial commenced, with reasons | 12 |
| Sample size | 7a | How sample size was determined | 9-10 |
|  | 7b | When applicable, explanation of any interim analyses and stopping guidelines | 10 |
| Randomisation: |  |  |  |
| Sequence | 8a | Method used to generate the random allocation sequence | 10,11 |
| generation | 8b | Type of randomisation; details of any restriction (such as blocking and block size) | 10,11 |
| Allocation | 9 | Mechanism used to implement the random allocation sequence (such as sequentially numbered containers), |  |
| concealment |  | describing any steps taken to conceal the sequence until interventions were assigned | 10,11 |
| mechanism |  |  |  |
| Implementation | 10 | Who generated the random allocation sequence, who enrolled participants, and who assigned participants to |  |
|  |  | interventions | 11 |
| Blinding | 11a | If done, who was blinded after assignment to interventions (for example, participants, care providers, those | 11 |

|  |  | assessing outcomes) and how |  |
| --- | --- | --- | --- |
|  | 11b | If relevant, description of the similarity of interventions | 8,9 |
| Statistical methods | 12a | Statistical methods used to compare groups for primary and secondary outcomes | 14 |
|  | 12b | Methods for additional analyses, such as subgroup analyses and adjusted analyses | 14 |
| **Results**  Participant flow (a | 13a | For each group, the numbers of participants who were randomly assigned, received intended treatment, and |  |
| diagram is strongly |  | were analysed for the primary outcome | Figure 1 |
| recommended) | 13b | For each group, losses and exclusions after randomisation, together with reasons | Figure 1 |
| Recruitment | 14a | Dates defining the periods of recruitment and follow-up | 6 |
|  | 14b | Why the trial end or was stopped | 10 |
| Baseline data | 15 | A table showing baseline demographic and clinical characteristics for each group | 16,17 |
| Numbers analysed | 16 | For each group, number of participants (denominator) included in each analysis and whether the analysis was |  |
|  |  | by original assigned groups | Tables 3-4 |
| Outcomes and | 17a | For each primary and secondary outcome, results for each group, and the estimated effect size and its |  |
| estimation |  | precision (such as 95% confidence interval) | Tables 3-4 |
|  | 17b | For binary outcomes, presentation of both absolute and relative effect sizes is recommended | Tables 3-4 |
| Ancillary analyses | 18 | Results of any other analyses performed, including subgroup analyses and adjusted analyses, distinguishing |  |
|  |  | pre-specified from exploratory | 23,24 |
| Harms | 19 | All important harms or unintended effects in each group _(for_ _specific_ _guidance_ _see_ _CONSORT_ _for_ _harms)_ | 24 |
| **Discussion**  Limitations | 20 | Trial limitations, addressing sources of potential bias, imprecision, and, if relevant, multiplicity of analyses | 27 |
| Generalisability | 21 | Generalisability (external validity, applicability) of the trial findings | 25-27 |
| Interpretation | 22 | Interpretation consistent with results, balancing benefits and harms, and considering other relevant evidence | 25-27 |
| **Other information**  Registration | 23 | Registration number and name of trial registry | 2,6 |
| Protocol | 24 | Where the full trial protocol can be accessed, if available | 2,6 |
| Funding | 25 | Sources of funding and other support (such as supply of drugs), role of funders | 30 |
