# Supplementary material for: Evaluating gain and loss-framed mobile messaging to improve maternal health service uptake: A cluster randomized controlled trial in Jimma, Ethiopia
Source: PLoS One. 2026 Oct 26;21(10):e0360474. doi: 10.1371/journal.pone.0360474 (PMC13704815; doi:10.1371/journal.pone.0360474)
Supplement: Supplementary file 6 [file pone.0360474.s003.docx]

# Message types and frequency of messages sent to study participants

| **S.no** | **Loss framed messages (Total message =39, n=196)** | **Freq.** | **Gain framed messages (Total Message =39, n=196)** | **Freq.** |
| --- | --- | --- | --- | --- |
| 1 | Eight antenatal contacts are recommended. If you do not follow recommended and regular, antenatal care visits, you and your baby will not be best protected. | 1 | Eight antenatal contacts are recommended. If you follow recommended and regular antenatal care visits, you and your baby will be best protected | 1 |
| 2 | Three postnatal contacts are recommended. If you do not follow recommended and regular, postnatal care visits, you and your baby will not be best protected. | 1 | Three postnatal contacts are recommended. If you follow recommended and regular postnatal care visits, you and your baby will be best protected | 1 |
| 3 | Not eating food with high of whole grains, vegetables, fruits, low fat dairy, and lean protein will increase your chance of having ill and low birth weight baby | 1 | Eat food with high whole grains, vegetables, fruits, low fat dairy, and lean protein to, have a healthy and average weight baby at birth. | 1 |
| 4 | Alcohol intake may increase the chance of occurrence of birth defects, low intelligence and behavioural or learning disabilities to your baby. | 1 | If you avoid alcohol, you will prevent occurrence birth defects, low intelligence and behavioral or learning disabilities to your baby. | 1 |
| 5 | Not taking daily Iron-folic acid supplementation or not eating food rich of iron increase chance of anemia, puerperal sepsis, low birth weight, and preterm birth. | 1 | Taking Iron-folic acid supplementation daily or eating food rich of iron prevents anemia, puerperal sepsis, low birth weight, and preterm birth. | 1 |
| 6 | Consuming raw meat will not only increase the risk of toxoplasmosis, tapeworm, schistosomiasis, but also of suffering their consequence. | 1 | Avoid eating raw meat to prevent your risk of getting toxoplasmosis, tapeworm, schistosomiasis and suffering their consequence. | 1 |
| 7 | Failing to go health facility as a result of severe headache, fever, nausea and vomiting increase chance of getting obstetric and perinatal complications. | 1 | Going to health facility as a result of severe headache, fever, nausea and vomiting lower chances of getting obstetric and perinatal complications. | 1 |
| 8 | Consuming unwashed vegetables and fruit, raw milk, processed/packed food can make you ill. | 1 | Avoid unwashed vegetables and fruit, raw milk, processed/packed food for your health and your baby | 1 |
| 9 | Consumption of more than 3 cups coffee per day will increase your risk of pregnancy loss and having baby with low birth weight | 1 | Consumption of less than 3 cups coffee per will decrease your risk of pregnancy loss and having baby with low birth weight. | 1 |
| 10 | If you don’t contact health worker when you notice decreased/lost fetal movement, you cannot learn what is abnormal and do not get early treatment | 1 | If you contact health worker when you notice decreased/lost fetal movement, you can learn what is abnormal and get early treatment | 1 |
| 11 | By not going to health facility as a result of dizziness, blurred vision, vaginal bleeding and abdominal pain, you will fail to save yours and your baby’s life | 1 | Going to health facility as result of blurred vision, vaginal bleeding and abdominal pain, you will save yours and your baby’s life | 1 |
| 12 | Not eating one extra food at each meal during pregnancy can lead to have less resistance against diseases, have ill baby, stillbirths and low-birth-weight | 1 | Eating one extra food at each meal during pregnancy can help to have resistance against diseases, healthy baby, reduced risk of stillbirths and low-birth-weight | 1 |
| 13 | If you do not wash your hands with soap before you prepare meals, after using the toilet and after handling animals, you will have an infection | 1 | Washing your hands with soap before you prepare meals, after using the toilet and after handling animals protects you from infection | 1 |
| 14 | Failing to go to health facility immediately when there is sudden gush of fluid per vagina increase risk of infection that will cost your and your baby’s life | 1 | Immediately going to health facility as a result of sudden gush of fluid per vagina will decrease risk of infection so that you save yours and your baby’s life | 1 |
| 15 | If you fail to consume meat, fish, eggs, poultry, milk and yoghurt, your baby will not grow well, become ill and you may not have good pregnancy outcome | 1 | Eat meat, fish, eggs, poultry, milk, yoghurt so your baby will grow well, healthy and you will have good pregnancy outcome | 1 |
| 16 | If you don’t have a skilled attendant during child birth, you will not have safe delivery of the baby and placenta, so that it cost yours and your baby’s life | 1 | If you have a skilled birth attendant during child birth, you will have safe delivery of baby and placenta so that you can save your and your baby’s life. | 1 |
| 17 | If you do not consume iodized salt in your food, your baby's brain may not develop well. | 1 | If you consume iodized salt in your food, your baby's brain will develop well. | 1 |
| 18 | If you don’t go to health facility when you have prolonged labour, hands and legs come out first and bleeding, you will fail to save yours and your baby’s life | 1 | By going to health facility when you have prolonged labour, hands and legs come out first, and bleeding, you will save yours and your baby’s life. | 1 |
| 19 | If you don’t plan to give birth at health facility, you won’t get management of complications and fail to prevent it’s life-threatening consequences | 1 | If you plan to give birth at health facility, you will take advantage of getting management complications and prevent it’s life-threatening consequences | 1 |
| 20 | Failing to find out ambulance service phone number and the fastest way to health facility could lower chance of yours and your baby’s survival during emergency | 1 | If you find out ambulance service phone number and the fastest way to health facility, the chance of yours and your baby survival during emergency will be high | 1 |
| 21 | If you don’t make ready 2 cloths (1 to dry and 1 to wrap after birth) and small cloth to cover the head, you can’t keep the baby warm and prevent illness. | 1 | If you make ready 2 cloths (1 to dry and 1 to wrap after birth) and small cloth to cover the head, you can keep the baby warm and prevent illness. | 1 |
| 22 | Choosing not to put your new baby to the breast in the first hour he/she is born will lowers a baby’s protection from many diseases and illnesses. | 1 | Choosing to put your new baby to the breast in the first hour he/she is born will in increases a baby’s protection from many diseases and illnesses. | 1 |
| 23 | If you and your family don’t start saving money, you won’t have extra money to use for emergency transportation to health facility during child birth | 1 | If you and your family start saving money, you will have extra money to use for emergency transportation to health facility during child birth | 1 |
| 24 | If you fail to give a baby a creamy first milk, your baby will not get high amount of nutrient so that the baby will not be protected from many disease | 1 | Giving a creamy first milk will provide your baby with high amount of nutrient so that your baby will be protected from many disease | 1 |
| 25 | If you don’t vaccinate your child according to a schedule, you will not take advantage of lifelong immunization and protect your child against diseases | 1 | By vaccinating your child according to a schedule, you will take advantage of lifelong immunization and be able to protect your child against diseases | 1 |
| 26 | Choosing not breastfeed baby is failing to take advantage of having mix of **vitamins,** protein, and fat so that baby won’t be protected from diseases and illnesses | 1 | Choosing to breastfeed baby is taking advantage of having mix of **vitamins,** protein, and fat so that baby will be protected from diseases and illnesses | 1 |
| 27 | If you don’t give only breast milk to your baby for the first 6 months, your baby will have [**infections**](https://www.webmd.com/cold-and-flu/ear-infection/default.htm), respiratory illnesses, and [**diarrhea**](https://www.webmd.com/digestive-disorders/digestive-diseases-diarrhea) | 1 | If you give only breast milk to your baby for the first 6 months, your baby will have fewer [**infections**](https://www.webmd.com/cold-and-flu/ear-infection/default.htm)**,** respiratory illnesses, and [**diarrhea**](https://www.webmd.com/digestive-disorders/digestive-diseases-diarrhea) | 1 |
| 28 | Skipping the first dose of vaccine against polio at birth will increases the chance of your baby infected by polio virus which cause physical disability | 1 | Getting the first dose of polio vaccines at birth will protect your baby from getting polio virus which cause physical disability | 1 |
| 29 | If you don’t sponge the cord with clean water, covered it and put anything on it, you will not prevent cord infection which make your baby very ill. | 1 | If you sponge the cord with clean water, leave it uncovered to dry and put nothing on it, you will prevent cord infection which make your baby very ill. | 1 |
| 30 | Failing to make sure your baby got vaccination against TB will increases the risk that your baby become sick due to TB infection | 1 | Getting vaccine against TB at birth protects your baby from getting TB infections for lifelong after taking it as soon as he/she is born | 1 |
| 31 | Not going to health facility immediately as the baby eyes, palms and feet turn yellow, the baby will not be treated on time and it will cost your baby’s life. | 1 | Taking a baby to health facility immediately as the baby eyes, palms and feet turn yellow, help to get timely treatment so that you will save your baby’s life | 1 |
| 32 | Not go to health facility immediately as there is strong contractions and jelly-like substance on underwear will make you not have safe delivery | 1 | If you go to health facility immediately as you feel strong contractions and jelly-like substance on underwear you will safe delivery | 1 |
| 33 | By not putting your baby on your bare chest under a blanket, you cannot effectively keep your warmed and be healthy. | 1 | By putting your baby on your bare chest under a blanket, you can effectively keep your warmed and be healthy. | 1 |
| 34 | By not going to health facility when the baby is having trouble breathing and too hot or cold, the problem will not early recognized and treated on time | 1 | If you take a baby to health facility when the baby is having trouble breathing and too hot or cold, the problem will early recognized and treated on time | 1 |
| 35 | If you bath a baby during the first 24 hours his/her life, not wrapping up and covering him/her head well will affect the health of a baby | 1 | Avoiding bathing a baby during first 24 hours of life, wrapping up and covering head well, will help to take an advantage of keeping a baby healthy. | 1 |
| 36 | If you don’t go to health facility immediately when there is profuse blood loss, swelling of calves and shortness of breath you will not be treated timely | 1 | If you go to health facility immediately profuse blood loss, swelling of calves, shortness of breath will be treated in a timely manner | 1 |
| 37 | Low back pain is a sign that the baby is low down, ready to get born. By fail to go to health facility on time, you will have bad pregnancy outcome | 1 | Low back pain is a sign that the baby is low down, ready to get born. By going to health facility on time, you will have better pregnancy outcome | 1 |
| 38 | The greatest disadvantages of not going to health facility when there is severe headaches, blurred vision and high fever after birth is failing to save oneself | 1 | One of the greatest advantages of going to health facility when there is severe headaches, blurred vision and high fever after birth s saving oneself | 1 |
| 39 | Not eating 2 extra foods at each meal during breastfeeding is failing to resistant against diseases, have healthy baby and get lost nutrients through breastmilk | 1 | Eating 2 extra food at each meal during breast feeding is becoming resistant against diseases, have healthy baby and get the lost nutrients through breastmilk | 1 |
