# Supplementary material for: Evaluating gain and loss-framed mobile messaging to improve maternal health service uptake: A cluster randomized controlled trial in Jimma, Ethiopia
Source: PLoS One. 2026 Oct 26;21(10):e0360474. doi: 10.1371/journal.pone.0360474 (PMC13704815; doi:10.1371/journal.pone.0360474)
Supplement: Supplementary file 7 [file pone.0360474.s004.docx]

| **Section** | **Reviewer’s Comment (Revised)** |
| --- | --- |
| **Title** | The title is informative and conveys the key components of the study. However, it should be more concise and polished for better readability. For instance: "Evaluating Gain and Loss-Framed Mobile Messaging to Improve Maternal Health Service Uptake: A Cluster Randomized Trial in Rural Jimma, Ethiopia." |
| **Keywords** | No revisions required. |
| **Abstract** | The abstract is well-structured and follows a logical sequence. Improvements are needed to trim excessive detail, ensure consistent terminology (e.g., "self-efficacy"), and enhance the conclusion to emphasize the significance of the findings more effectively. |
| **Trial Registration** | Trial registration details are inaccessible online. Authors should confirm registration compliance, including providing the registration number, registry name, and URL if available. Any missing details must be addressed or explicitly acknowledged to ensure transparency. |
| **Background** | The section contains the required elements but lacks flow and coherence. A structured writing style should be followed, such as moving from global to local contexts or vice versa. Additionally, ideas should align closely with the study objectives for better synchronization. |
| **Method** | **Setting:** The criteria for selecting Manna, Shebe Sombo, and Dedo districts from the Jimma Zone must be clarified  **Population:** The inclusion of primiparous women in the sample is unclear, as baseline data were collected from those with at least one prior childbirth. Clarification is needed  **Sample size:** The sample size appears insufficient considering 21 clusters. Consultation with a statistician is recommended to justify whether the current sample size is adequate. If 21 clusters are considered for sample size calculation, the required sample size would be much larger.  **Intervention development:** Correct flow and remove repetitions, e.g., the repeated definition of loss frame and word frame (lines 182–184 and 216–218). Clarify who delivered the SMS messages—telecommunication personnel, researchers, or others—and whether mechanisms were in place to confirm participants received and read the messages.  **Blinding:** Line 297 states, “Blinding the participants was not feasible due to the nature of the intervention.” Clarify why SMS interventions made blinding impossible. |
| **Data Collection Procedure** | Line 343-344 mentions, “Baseline data were collected using a paper-based method, while endline data were collected using the Kobo toolkit.” Clarify whether this methodological difference could introduce bias. |
| **Analyses** | The manuscript states that “Generalized Linear Mixed Model (GLMM)” was used, but no GLMM results appear in the results section. This requires justification. Additionally, explain why GLMM was chosen instead of Generalized Estimating Equations (GEE), which are often preferred for non-normally distributed data. |
| **Result** | The first four sentences of this section belong to the methods section and create redundancy, so they should be removed.  Include data for both baseline and endline participant characteristics, rather than focusing solely on baseline.  Address the significant differences in ANC utilization rates between the gain and loss frame groups at baseline (85.7% vs. 90.8%). Clarify how these differences were managed to ensure the conclusions are attributable to messaging effects rather than baseline disparities.  The observed changes in institutional births are exceptionally significant, with a 37% increase in the gain-framed group and a 26% increase in the loss-framed group. These findings warrant a thorough review to ensure the validity and accuracy of the data. The reviewer strongly recommends revisiting the original dataset and conducting reanalysis if necessary to confirm the reliability of these results. If verified, such outcomes would represent an unprecedented level of effectiveness with profound global implications. However, the magnitude of these changes, described as world-leading, raises concerns about data integrity and representation. The authors should scrutinize the findings to ensure their accuracy and reliability.  Additionally, while ancillary analyses are noteworthy, it is crucial to first evaluate whether secondary variables—such as knowledge, attitude, self-efficacy, and maternal morbidity—predicted antenatal care (ANC), delivery, and postnatal care utilization. |
| **Discussion** | This section is acceptable but must be revised in light of changes to previous sections to ensure consistency. |
| **Conclusion** | The conclusion suggests that both gain and loss frames are effective, but the authors should clearly indicate which approach is more effective. Numerical figures, such as “gain-framed arm improved ANC by 11.6%, institutional births by 26.3%, PNC by 12.8%, etc.,” should not appear in the conclusion. Additionally, ensure all reported figures match the results section. |
| **Reference** | Numerous references, such as “WHO Maternal Health. Who 2018, 1–47,” are not cited correctly according to the Vancouver style. For example, this reference lacks the title. Use reference management software such as Mendeley to correct these errors systematically. |
| **Language** | Ensure coherent descriptions to avoid disconnections and unnecessary repetitions. Multiple grammatical, typographical, and spelling errors must be corrected through thorough editing. |
