# Supplementary material for: Evaluating gain and loss-framed mobile messaging to improve maternal health service uptake: A cluster randomized controlled trial in Jimma, Ethiopia
Source: PLoS One. 2026 Oct 26;21(10):e0360474. doi: 10.1371/journal.pone.0360474 (PMC13704815; doi:10.1371/journal.pone.0360474)
Supplement: Supplementary file 8 [file pone.0360474.s007.docx]

# Suggestions

## First Thought:

Please add line numbers in the manuscript so that it is easier for reviewers to refer to the lines on which they are commenting. Without line numbers, it is hard to track for both reviewers and authors to add/address the comments.

## Introduction

1. The background clearly addresses burden of maternal mortality globally and in Ethiopia, and the authors appropriately highlight the persistent gap between current indicators and the SDG targets. However, while the epidemiological justification is adequate, the section reads more as a descriptive overview than as a tightly argued rationale for a theory-driven experimental study.

Suggestion: The introduction should move beyond presenting statistics and more clearly build a conceptual argument leading to the necessity of comparing gain- and loss-framed messaging in this specific context.

2. The theoretical foundation of message framing seems to be insufficiently developed. Although the manuscript mentions persuasive messaging and framing effects, it does not explicitly anchor the study in established behavioral theories such as Prospect Theory, Protection Motivation Theory, or the Health Belief Model. The distinction between gain- and loss-framed messaging is described at a definitional level, but the mechanisms through which framing is expected to influence maternal health service uptake are not articulated.

Suggestion: the paper will highly benefit if a clearer explanation of why one framing strategy might be more effective for preventive maternal health behaviors, and how this expectation is grounded in theory is mentioned rather than general persuasion literature.

3. The discussion of preventive versus detection behaviors is introduced but not integrated into the argument. You noted that framing effectiveness depends on the type of target behavior, yet they do not clearly classify antenatal care attendance, institutional delivery, and postnatal care within this framework. If these are considered preventive behaviors, existing theory often predicts stronger effects for gain-framed messages.

Suggestion: You should clarify this classification and state whether their hypotheses are directional or exploratory.

4. The research gap is present but not sharply articulated. The manuscript states that the effect of message-framing strategies on maternal healthcare service utilization via mobile phone messaging has not been extensively studied, particularly in LMICs. However, this claim remains broad. It would strengthen the argument to specify whether prior studies were non-experimental, conducted in high-income settings, focused only on attitudes rather than service uptake, or lacked rural populations, or this kind of trial has been established service uptake for boosting other types of healthcare visit among service takers.

Suggestion: The novelty of conducting a three-arm cluster-randomized controlled trial in rural Ethiopia should be emphasized more clearly and positioned as a distinct contribution to the literature.

7. The manuscript includes multiple grammatical errors, typographical inconsistencies, and formatting issues that must be corrected. Examples include duplicated phrases, incorrect punctuation spacing, subject–verb disagreement, and fragmented sentence flow.

Suggestion: Check for typos all across the manuscript

8. The scope of outcomes appears ambitious and potentially overextended. The study aims to evaluate effects not only on service utilization and psychosocial constructs such as knowledge, attitudes, and self-efficacy, but also on maternal morbidity. The background does not sufficiently justify how message framing alone could plausibly affect clinical morbidity within the study timeframe, nor does it explain whether the trial is powered for such outcomes. This raises concerns about conceptual overreach and the risk of underpowered secondary outcomes.

## Methods

1. Trial registration and protocol transparency are strengths, but the manuscript should report the registration more precisely, including whether registration was prospective relative to first enrollment and intervention start. The trial is registered on January 4, 2022, yet the baseline survey occurs in March 2023 and the intervention in May–December 2023, so it appears prospectively registered; however, the reader should not have to infer this. Also, “fullstuy protocol” and related wording errors should be corrected, and the manuscript should explicitly state whether any deviations from protocol occurred beyond the claim of “no changes,” especially given some inconsistencies later in the methods narrative.
2. The setting description is informative but requires clearer separation of what is descriptive context versus what is used for selection criteria. Districts were purposively selected due to “no ongoing similar interventions,” functional posts, network coverage, and geographic diversity; this is reasonable, but it limits generalizability and should be framed explicitly as such in the methods. Additionally, the statement that Jimma Zone has “poor health infrastructure” and a “low health worker density according to WHO standards (3.04 per 1,000)” would benefit from a citation and clarification because the interpretation depends on which WHO benchmark is being applied and to which cadre definition.
3. There is a notable timeline inconsistency that needs correction because it affects credibility of implementation. In the ‘Intervention Development Section’, the manuscript states recruitment of intervention participants was conducted from March 1 to March 14, 2024, yet the intervention follow-up is May–December 2023 and endline survey is February 2024. Recruitment cannot occur after endline and after the intervention period. This appears to be a serious typographical or reporting error and should be corrected with exact dates for recruitment, enrollment, randomization, and first message delivery.
4. The trial design is described as a three-arm parallel cRCT with baseline in March 2023 and endline in February 2024, implying a cohort followed over time. However, the text later suggests baseline data were collected from primiparous and multiparous women “reflecting previous childbirth experiences,” which is confusing because participants are pregnant at 16–20 weeks gestation at baseline. You should clearly state whether baseline and endline data were collected from the same women (a cohort) or whether there was any replacement or cross-sectional sampling at endline. This is crucial for choosing correct statistical models and for interpreting attrition.
5. Eligibility criteria are broadly appropriate, but the exclusion of women (or family members) unable to read messages raises equity concerns and may systematically exclude the most vulnerable. If the intervention requires literacy, you should at minimum acknowledge selection bias and consider whether alternative delivery modes (voice, interactive voice response, or community facilitation) were considered. Additionally, the wording around residence (“lived in the selected health posts”) should be corrected to “within the health post catchment area,” and “family member owns a phone” should be operationalized because it may introduce privacy and autonomy issues for pregnant women that could influence outcomes.
6. The intervention description contains internal inconsistencies that must be resolved. In one place, it says participants received 39 messages over eight months at a frequency of 1–2 per week, but later it says messages were sent three times per week totaling 39 messages per participant. Three messages per week over eight months would far exceed 39 messages. The dose, schedule, and tailoring logic (by gestational week, EDD, postnatal timeline) should be specified precisely, including the trigger point (“from ANC enrolment”) and what happens if enrolment occurs late.

Suggestion: A clear intervention fidelity framework is expected, including delivery logs, success/failure rates, and what proportion of scheduled messages were actually delivered and received.

7. The message development process is described in detail, which is commendable, but methodological rigor could be improved by specifying how the “10 evaluation statements” used by experts were developed, whether content validity indices were computed, and whether the card-sorting results were quantified. The manuscript should also clarify if gain- and loss-framed messages were matched on topic, length, readability, and timing so that framing is truly the only manipulated dimension. Otherwise, differences could be due to content, not frame.
8. The control condition is described as “usual care,” but it includes guidance about eight ANC visits and three PNC visits that reflects updated recommendations. The manuscript should clarify what usual care actually looked like in the study districts during 2023–2024, because “usual care” often deviates from guidelines. If health facilities in practice commonly delivered fewer visits or different counseling content, describing guideline recommendations does not adequately define the comparator. This is especially important because the primary outcome is defined as achieving at least eight ANC visits, which may be difficult in this context and could risk floor effects.
9. Randomization and allocation concealment need clearer reporting. The manuscript describes random selection of primary healthcare units and then random selection of clusters, but it is not fully clear whether randomization was stratified by district or PHCU, which is important given potential district-level differences. The allocation method using Excel random numbers and ordering clusters is acceptable, but the description also says a statistician randomly assigned clusters, creating ambiguity about who actually conducted randomization and whether the process was independent.

Suggestion: You should clearly state who generated the sequence, who enrolled clusters, and who assigned clusters to arms, ideally in CONSORT-style role separation.

10. Participant sampling within clusters is insufficiently detailed. The manuscript states that 28 participants were randomly selected from lists of eligible women, but it does not describe the random selection method, handling of refusals, or what happened if fewer than 28 eligible women were available at a health post. The recruitment process using “local volunteers” also requires ethical and procedural clarification, including training, supervision, and how confidentiality was preserved during home-to-home screening.
11. Blinding is partially addressed, but there are contradictions and important implications for detection bias. Participants could infer group assignment due to message tone, and performance bias is likely, yet the manuscript treats this as an unavoidable limitation without describing mitigation strategies. Also, it states midwives were unaware of cluster allocation during recruitment, but after clusters are assigned and the intervention starts, local providers and participants may become aware of exposure, potentially influencing care-seeking and reporting. More detail is needed on whether data collectors were geographically separated from implementers and how blinding was maintained in practice, especially during endline verification at facilities.
12. Outcome definitions require refinement for validity and comparability. Defining ANC utilization as “at least eight ANC visits” is aligned with newer WHO guidance, but many LMIC studies use ANC4+; the rationale for ANC8 as the primary endpoint must be justified in relation to feasibility and baseline rates. PNC utilization is defined as visits “within seven days of delivery,” whereas earlier background and guideline language often refers to within 48 hours or within two days; You should justify the chosen window and specify whether it is “at least one PNC visit within seven days” or “completed recommended PNC schedule.” If multiple primary outcomes are used, the multiplicity strategy should be stated clearly upfront and aligned with the sample size and analysis plan.
13. Measurement of maternal morbidity is particularly concerning. It is assessed via self-reported illnesses and complications (including preeclampsia, postpartum hemorrhage, infections, anemia), which are clinically complex diagnoses and may not be accurately self-reported without confirmation. If morbidity is retained as a secondary outcome, the authors should specify operational definitions, recall periods, validation procedures, and whether any clinical record verification occurred. Without this, morbidity findings risk substantial misclassification bias.
14. The sample size section shows effort and transparency, but there are technical and reporting issues. The stated “Bonferroni-adjusted Zα/2 = 2.24” does not correspond to α = 0.05 for two-sided tests; it implies a smaller alpha, and the authors should explicitly state the number of comparisons being adjusted and the exact adjusted alpha per comparison. There are also typographical issues in the formula (e.g., “Zβ/2”) and minor numerical inconsistencies (175 vs 174.88). More importantly, the repeated-measures design effect (dr) is presented in a way that reduces sample size (dr < 1), which can be correct under certain assumptions, but it is unusual for authors to apply it without clearly stating whether baseline adjustment is via ANCOVA-type modeling, difference-in-differences, or mixed models and how the cluster autocorrelation was sourced or justified.
15. The analytic approach raises a major methodological concern: using a Per-Protocol analysis as the primary approach is not standard for pragmatic cluster RCTs and risks bias if adherence is related to outcomes. Researchers typically expect intention-to-treat (ITT) as the primary analysis, with PP as secondary or sensitivity. If PP is maintained, you must define adherence operationally (e.g., proportion of messages delivered, read, or acknowledged) and show how adherence data were measured. Additionally, the use of multiple imputation “at both baseline and follow-up” alongside PP analysis needs careful justification because imputation commonly aligns with ITT estimands and prespecified missingness assumptions; otherwise, the estimand is unclear.
16. Model specification should be expanded and corrected where necessary. The manuscript states that baseline and endline outcomes were collected from the same individuals, yet it proposes GLMM/LMM with a random intercept for health post only. If repeated measures on individuals are present, the model should include a participant-level random effect (or an appropriate covariance structure) to account for within-person correlation over time. The text also mentions fixed effects for time and intervention-by-time, which is appropriate, but you should specify link functions for binary outcomes, whether robust standard errors were used, how ICCs were estimated, and whether district or PHCU was included as a stratification factor or covariate.
17. The data collection and verification procedures are a relative strength, particularly the attempt to verify outcomes using ANC cards and facility registers for about 82% of participants. However, the manuscript should describe how verification was performed in a blinded manner and whether verification rates differed by arm, as differential verification could bias results. The shift from paper-based baseline to KoBo endline introduces potential mode effects; the authors should state whether the questionnaires were identical, whether skip logic differed, and how consistency across modes was ensured beyond translation/back-translation.
18. Ethical considerations are largely adequate, but the section could be strengthened by addressing confidentiality risks inherent in messaging interventions, especially when phones may belong to family members. The manuscript should explain how consent handled shared phone access, how sensitive content was managed, and whether participants could opt out or stop messages at any point. Also, the statement that phone numbers were encrypted and accessible only to technicians and researchers is good, but it would benefit from brief technical clarification (e.g., hashing, encryption at rest, access control) without becoming overly technical.

To sum up, the methods are promising and contain many expected components, but major revisions are required to correct timeline contradictions, resolve intervention-dose inconsistencies, clarify cohort structure and sampling, strengthen randomization/blinding reporting, refine outcome definitions, and align analysis with standard RCT estimands and appropriate mixed-model structures.

One important methodological concern relates to potential baseline imbalance in determinants of maternal health service utilization across clusters and study arms. Given the relatively small number of clusters per arm (n=7), simple randomization without matching or constrained allocation increases the risk that clusters differed at baseline in key predictors such as parity, prior ANC attendance, prior facility delivery, baseline knowledge, attitudes, and self-efficacy. These factors are strong determinants of ANC uptake and institutional delivery independent of messaging exposure. Women who are already positively disposed toward maternal health services, or who have prior pregnancy experience, may seek care regardless of message framing, thereby diluting or confounding the true intervention effect. The manuscript does not indicate whether cluster-level characteristics were considered during randomization or whether balance on these critical variables was assessed prior to intervention rollout.

To strengthen internal validity, you should provide detailed baseline comparability tables at both individual and cluster levels, including parity, prior service utilization, baseline ANC status at enrollment, and baseline knowledge, attitude, and self-efficacy scores. If any imbalance exists, analytic adjustment should be clearly specified within the mixed-model framework. Furthermore, given that responsiveness to framing may plausibly differ by baseline motivation or prior experience, exploration of effect modification (e.g., intervention × parity or intervention × baseline knowledge interactions) would be valuable. Without demonstrating adequate baseline balance or robust adjustment for these predictors, it remains difficult to attribute observed differences in service utilization solely to message framing rather than to pre-existing differences in maternal health-seeking propensity across clusters.

Addressing these issues will substantially improve internal validity, transparency, and confidence in the causal interpretation of framing effects.

## Result

1. The participant flow is generally clear and attrition is low, which is a strength for a community-based cRCT. However, the reporting of exclusions due to “maternal deaths (n=3)” is concerning and requires substantially clearer documentation. The manuscript should clarify whether these deaths occurred during pregnancy, childbirth, or postpartum, whether they were evenly distributed by arm, and whether any relationship to intervention exposure was assessed, even if only descriptively. For Trial Studies, maternal deaths during a maternal health trial cannot be treated as routine loss-to-follow-up; at minimum you should indicate whether an independent review or adverse event reporting process was followed and whether the trial registration/protocol specified adverse event handling.

2. The baseline characteristics section reveals important imbalances across arms that directly threaten causal interpretation, and the authors acknowledge “observable differences” but do not adequately address their implications. For example, marital status and occupation differ markedly across arms (e.g., 100% married in gain vs 86.2% in loss), and there appear to be inconsistencies/typos within the table (e.g., “Can read and write” in control shown as “10 (51)” which is clearly erroneous). These imbalances matter because education, marital stability, occupation, and partner characteristics are well-known predictors of health service utilization and may correlate with phone access and ability to act on messages. In a cRCT with only 7 clusters per arm, such baseline differences should prompt either covariate-constrained randomization (not done) or very explicit analytic adjustment. At minimum, you should report standardized mean differences and specify exactly which covariates were included in the adjusted models reported in Tables 3–4.

3. The baseline values of primary outcomes raise major interpretability concerns. ANC “utilization” at baseline is already extremely high (85–91%) in all arms, yet the defined primary endpoint in the methods was “at least eight ANC visits.” It is unclear whether baseline ANC in Table 3 is the same definition (ANC8+) or whether it reflects any ANC attendance, ANC initiation, or some other threshold. This is critical because if baseline ANC is measured while women are at 16–20 weeks gestation, “eight ANC visits” is not feasible at baseline, so the baseline metric cannot logically be ANC8+. The manuscript must explicitly define what “Pre” and “Post” represent for ANC, SBA, and PNC, and ensure the definition aligns with the methods. As currently written, the results suggest a definitional mismatch and potentially invalid comparisons.

4. The direction and magnitude of effects reported for primary outcomes are unusually large and partly driven by a substantial decline in the control arm (e.g., ANC from 85.7% to 75.5%, facility delivery from 82.7% to 60.7%, PNC from 57.7% to 44.9%). These difference is not subtle, rather a matter of serious concern. Such declines in usual care over one year are possible but require explanation because they inflate relative effects and could reflect external shocks, measurement differences between baseline and endline, reporting bias, or differential verification rather than a true intervention impact. The authors should discuss potential secular changes in service provision, strikes, conflict, supply disruptions, or differences in measurement mode (paper baseline vs KoBo endline), and they should show whether verification rates differed by arm. Without contextual explanation, the dramatic control-arm decline raises suspicion of systematic error or non-comparability between time points.

5. The ICC values reported for primary outcomes are very high (approximately 0.22–0.48 and up to ~0.47 in Table 3), indicating strong cluster-level effects. This directly contradicts the extremely low ICC used in the sample size calculation (ρ=0.003). This is a major issue: the trial appears to have been powered under an assumption of minimal clustering, yet observed clustering is moderate-to-high. The authors must reconcile this discrepancy, explain why the ICC assumption was so low, and discuss implications for precision and type I error. Even if they used mixed models, underestimated ICC in planning can still mean inadequate power or unstable estimates, particularly with only 21 clusters.

6. The reporting of effect estimates needs clearer alignment with the analytic model. The footnote states the ORs come from GLMM comparing each intervention arm vs control “at endline” and adjusting for baseline values and specified covariates, but the covariates are not listed, and it remains unclear whether the model uses repeated measures properly (time × arm interaction) or is simply endline-adjusted. Because baseline imbalance is present and because outcomes changed markedly in the control group, the model specification (difference-in-differences vs ANCOVA) materially affects interpretation. Journal would expect explicit reporting of the fixed effects included, the link function, whether participant-level repeated measures were modeled (random effect for participant), and whether robust standard errors were used given the small number of clusters.

7. The secondary outcome results show large improvements in knowledge and attitude in both intervention arms, which is plausible, but again baseline differences are substantial (e.g., knowledge and attitude are already higher in the intervention arms than in control at baseline). This makes it essential to show adjusted estimates transparently and to provide baseline-adjusted effect sizes (e.g., standardized mean differences) and not only aMD. Moreover, the knowledge scoring appears inconsistent with the methods: the methods describe 16 knowledge questions summed to a score, yet mean scores reported are in the 30s and 40s, suggesting either a different scoring range or transformation. The authors must clarify how knowledge scores were scaled and what the possible minimum/maximum values are.

8. The maternal morbidity findings are substantial and require strong caution. A reduction from ~20–24% to ~7–10% in intervention arms with no meaningful reduction in control suggests a very large effect of messaging on clinical outcomes, which is difficult to attribute causally given that morbidity was self-reported and diagnostically complex (e.g., preeclampsia, postpartum hemorrhage, anemia). This result may reflect reporting bias, differential interpretation of symptoms, differential recall, or increased care-seeking leading to different labeling of symptoms. Additionally, the OR direction is confusing: if morbidity declines, the OR should reflect reduced odds in intervention vs control, yet the table reports OR > 1 without specifying the modeled event (morbidity vs no morbidity). The authors must explicitly state what outcome was coded as “1” in the logistic model and ensure OR interpretation is correct.

9. The association analyses in Table 5 contain an apparent coding error that questions credibility. For example, the table shows “Maternal morbidity: No 1.99; Yes 1,” which implies that “No morbidity” increases odds of ANC, but the narrative says morbidity increases odds of ANC. Similar inconsistencies appear for facility delivery and PNC. This must be corrected because it affects substantive interpretation and suggests either reversed reference categories or mislabeling in the table. Also, these association analyses risk post-treatment bias if knowledge/attitude/self-efficacy were influenced by the intervention and then used as predictors of utilization; you should clarify whether these predictors are baseline measures, endline measures, or pooled, and interpret accordingly.

The results suggest potentially strong intervention effects, but the current reporting contains multiple internal inconsistencies (baseline outcome feasibility, scoring ranges, OR direction for morbidity, and mis-coded categories in Table 5), along with serious threats to validity (baseline imbalance, control-arm decline, high clustering not anticipated). Before the conclusions can be accepted, you must correct table errors, define outcomes consistently, transparently specify covariate adjustment. Your first concern is methodologically very strong and should absolutely be raised.

The baseline tables clearly show that knowledge and attitude scores were already higher in the intervention arms compared to control before the intervention began. That is not a trivial issue. When an intervention group starts at a higher baseline level on constructs that are strongly associated with the outcomes (as Table 5 suggests they are), two problems arise.

First, it suggests imperfect randomization or imbalance across clusters.

Second, it creates the risk that the observed post-intervention differences are partially attributable to pre-existing predisposition rather than the framing intervention itself.

Even if the authors adjust for baseline scores in the mixed models, large baseline imbalance in a small-cluster trial can still bias estimates, especially when clustering is strong and when covariate adjustment is not perfectly specified. Moreover, higher baseline knowledge and attitudes may correlate with other unmeasured advantages (e.g., better access, more supportive husbands, higher literacy), which are not fully captured by statistical adjustment. This is particularly important because the association analysis shows that knowledge and attitude are significantly related to service utilization. In such a scenario, the intervention effect may be overestimated if baseline predisposition is not fully accounted for. Without that, the causal interpretation remains vulnerable.

Second concern regarding the very wide confidence intervals is also important. The large 97.5% confidence intervals such as ORs with ranges like 2.20 to 25.79 or 2.40 to 21.26 indicate substantial statistical uncertainty around the point estimates. Wide confidence intervals typically arise from one or more of the following:

small effective sample size (which in cluster trials is driven more by number of clusters than number of individuals),

high intracluster correlation (which is reported as moderate to high), or

large variability in cluster-level effects.

In this trial, only 7 clusters per arm were used, and the observed ICCs are high (up to ~0.48), meaning much of the variance is at the cluster level. That dramatically reduces the effective sample size and inflates standard errors, leading to wide confidence intervals. In practical terms, although the point estimates suggest very strong effects (OR 5–7), the true effect could plausibly be much smaller or much larger within those intervals. For example, an OR of 7.5 with a CI of 2.2 to 25.8 means the estimate is unstable and highly sensitive to cluster-level variation. This reduces precision and raises concerns about overinterpretation of magnitude. The results may be statistically significant, but they are not precise.

My first concern goes to internal validity (possible baseline confounding and inflated effect sizes), while my second concern goes to precision and stability of the estimates (limited clusters and high ICC producing wide intervals). Both are legitimate high-level methodological concerns for a high impact journal.

## Ancillary Findings

1. The ancillary analysis as written is largely speculative and reads as post hoc interpretation rather than evidence-based analysis. The you state that the intervention “appears to have produced meaningful improvements” in service quality and waiting times, but they also acknowledge that service quality and provider behavior were not directly measured and the intervention was not intended to improve service quality. Causal language such as “produced improvements” is not acceptable when the constructs were not measured objectively and when no analytic strategy is presented to isolate intervention-related changes from secular trends. At most, you can report changes in participant perceptions and explicitly frame them as perceived barriers, not actual system-level quality improvement, and you should remove mechanistic explanations about health workers becoming “internally motivated” unless supported by data.

2. The barrier results presented (e.g., “poor-quality services” declining from 97.1% to 2.9%, long wait times from 98.6% to 1.4%, inconvenient hours from 95.8% to 4.2%) are implausibly large for an intervention that delivered SMS messages to pregnant women and did not include any health system or provider-facing component. Such extreme shifts strongly suggest measurement artifacts, social desirability bias, changes in question wording/administration between baseline and endline, or a reporting/analysis error. Importantly, the figure description indicates the results are aggregated baseline vs endline percentages, not stratified by arm. If the changes are pooled across all arms, they cannot be attributed to the intervention and may simply reflect time trends, seasonal effects, or changes in service delivery unrelated to the trial. You need to present these barrier outcomes by trial arm with appropriate cluster-adjusted models, or explicitly label them as descriptive secular changes not attributable to the intervention.

3. The manuscript also risks serious internal inconsistency: earlier results showed the control arm declining substantially in ANC, facility delivery, and PNC utilization, yet the ancillary analysis claims barriers such as distance, waiting times, and service hours were “almost entirely resolved.” If barriers truly improved so dramatically across the study period, a decline in utilization in the control arm becomes harder to reconcile. This contradiction reinforces the likelihood that these “barrier” measures are not stable, are subject to bias, or were not measured consistently across time. You should explain how these ancillary indicators were measured, whether the exact same items and response options were used at both time points, and whether data collection mode changes (paper vs KoBo) could have affected responses.

4. Birth preparedness indicators similarly show large improvements, particularly saving money for delivery rising from 72.7% to 94.6%. While improvements in preparedness are plausible as a pathway from messaging to service uptake, the current presentation lacks the minimum analytic requirements. You should report these changes by arm and test arm-by-time effects with cluster-adjusted models. If you intend these as mechanisms, you should explicitly frame them as mediator candidates and avoid causal claims unless mediation analysis was prespecified and appropriately performed. Otherwise, they should be labeled as exploratory descriptive findings and interpreted cautiously.

5. The harms/unintended effects section is a useful inclusion, but it is under-specified for a trial context. “Mild anxiety” and “confusion” need operational definitions (how assessed, by what instrument or question, when measured, and whether systematically solicited or passively reported). The statement that participants were “monitored” is vague; monitoring implies a structured approach, yet no method is described. Given that loss-framed messaging can plausibly induce anxiety, it is important to describe safeguards, opt-out procedures, frequency of complaints, and whether any participants discontinued ANC or disengaged because of distress. Also, the sentence “the observed effects did not impact the study outcomes” is not evidenced and should be removed or supported with data.

These ancillary findings, as currently written, weaken the manuscript because they over-interpret descriptive trends and introduce implausible claims without rigorous measurement or arm-stratified analysis. You should either (i) re-analyze these ancillary outcomes by arm with appropriate cluster-adjusted models and report them as exploratory, or (ii) move them to supplementary materials with restrained interpretation, clearly distinguishing perceived barriers from objective service quality changes and avoiding unsupported causal mechanisms.

## Discussion

1. The discussion appropriately summarizes the principal findings; however, it continues to overstate causal certainty in light of the methodological issues identified earlier. Statements such as “the intervention significantly improved utilization” and “both gain and loss-framed messages can significantly reduce maternal morbidity” are stronger than the design and data fully justify. Given the baseline imbalances in psychosocial variables, the high observed ICCs, the relatively small number of clusters, and the unexplained decline in the control arm, conclusions should be framed more cautiously. You should emphasize that the findings indicate an association within the context of this cluster-randomized design rather than definitive proof of large causal effects across settings.

2. The explanation offered for the decline in the control arm introduces a substantial internal validity concern. The authors attribute this decline to baseline campaigns and community mobilization activities that were not sustained at endline. If true, this represents a time-varying contextual confounder affecting the baseline–endline comparison. In that case, the intervention effect may partly reflect the withdrawal of external support rather than solely the addition of messaging. This possibility should be acknowledged explicitly as a limitation rather than used to reinforce the intervention’s apparent superiority. Moreover, if community bylaws and enforcement against home delivery were active during the study period, the potential for contamination or differential exposure across clusters should be discussed.

3. The interpretation of maternal morbidity reduction is particularly overstated. The manuscript attributes the reduction to increased ANC attendance and improved adherence, yet morbidity was self-reported and not clinically adjudicated. The magnitude of decline reported is large and biologically ambitious for an SMS-based behavioral intervention without a direct clinical component. Alternative explanations such as reporting bias, improved symptom interpretation, differential recall, or misclassification should be discussed. Without objective clinical verification, the claim that message framing “reduced maternal morbidity” is too strong and risks undermining credibility.

4. The comparative framing conclusions require more statistical restraint. The manuscript states that loss-framed messages were particularly effective for ANC and morbidity while gain-framed messages were more effective for SBA and PNC. However, the results section does not clearly present direct statistical comparisons between gain and loss arms. If head-to-head contrasts were not formally tested, these statements imply differences that may not be statistically supported. The discussion should clarify whether such comparisons were conducted and, if not, rephrase these conclusions to reflect descriptive trends rather than definitive superiority.

## Implication

The implications section is forward-looking but somewhat overgeneralized. While the findings suggest promise for tailored mobile messaging, recommending integration into policy strategies should be tempered by acknowledgment of contextual specificity. The districts were purposively selected, mobile coverage was adequate, and concurrent community activities were in operation. External validity to more resource-constrained or less mobilized settings remains uncertain. Policy recommendations should be framed as conditional and suggest replication with larger numbers of clusters and intention-to-treat analyses as primary estimands.

## Strengths

The strengths section appropriately highlights verification of outcomes and allocation concealment, but it does not fully address key methodological challenges. While adjustment for baseline covariates is mentioned, this does not entirely resolve baseline imbalance concerns in small-cluster trials. The reference to FDA and EMA guidance strengthens the rationale for covariate adjustment, yet the manuscript should clarify exactly which covariates were included and whether adjustment meaningfully altered effect sizes. Additionally, the high observed ICCs relative to the assumptions used in sample size planning are not discussed in the strengths or limitations, despite their implications for precision and generalizability.

## Limitations

The limitations section is currently underdeveloped relative to the complexity of the findings. Beyond recall bias and tool changes, the authors should explicitly acknowledge: the small number of clusters per arm; high intracluster correlation; baseline imbalances in psychosocial predictors; potential contamination from concurrent community activities; reliance on self-reported morbidity; inability to blind participants; and the risk of social desirability bias in reporting service utilization and barriers. Without a more transparent and comprehensive limitations discussion, the manuscript appears overly confident relative to its design constraints.

In overall, the discussion would benefit from a stronger theoretical integration. While you cite literature on gain versus loss framing, they do not deeply engage with why framing effects were modestly differentiated in this context or why self-efficacy did not improve significantly despite changes in knowledge and attitudes. A more nuanced theoretical reflection considering ceiling effects, cultural context, stage of behavior change, and preventive versus detection behaviors would elevate the manuscript and align it more closely with the standards expected. The discussion section contains important insights and promising findings, but it requires substantial moderation of causal claims, clearer acknowledgment of design limitations, and more rigorous linkage between theory, methods, and interpretation to meet publication standards.

## Conclusion

1. The conclusion is currently too definitive relative to the methodological constraints of the study. Phrases such as “the study conclusively demonstrates” and “significantly enhance maternal health outcomes” overstate the strength of inference that can reasonably be drawn from a cluster trial with a small number of clusters, baseline imbalances, high observed ICCs, and reliance on self-reported morbidity. Conclusions should reflect statistical association within the study context rather than absolute proof. The wording should be moderated to state that the findings “suggest” or “indicate” effectiveness in this setting, rather than conclusively demonstrating it.

2. The comparative claims regarding gain versus loss framing also exceed the evidence presented. The conclusion states that loss-framed messages were “more effective” for certain outcomes and gain-framed messages for others. However, unless formal head-to-head statistical comparisons between the two intervention arms were conducted and reported, such superiority claims are not fully justified. If the analyses only compared each intervention arm to control, then differences between gain and loss should be described as descriptive patterns rather than statistically established differences.

3. The statement that maternal morbidity was “reduced” by the intervention should be carefully reconsidered. Because morbidity was self-reported and not clinically adjudicated, and given the magnitude of change observed, the conclusion should refer to reductions in reported maternal morbidity rather than implying direct clinical impact. Without objective outcome validation, stronger clinical language may invite skepticism from reviewers and readers.

4. The conclusion would benefit from clearer acknowledgment of contextual boundaries. The study was conducted in purposively selected districts with concurrent community mobilization activities and adequate mobile network coverage. Therefore, generalization to other regions especially those with weaker infrastructure or different sociocultural dynamics should be framed cautiously. Rather than broad claims about “similar contexts,” it would be more appropriate to suggest that mobile phone-based framed messaging shows promise and warrants further evaluation in diverse settings with larger cluster samples.

5. Finally, the conclusion should more clearly integrate the theoretical contribution of the study. Instead of primarily emphasizing outcome improvements, it could briefly highlight that framing effects appear to operate differently across specific maternal health behaviors and psychosocial domains, reinforcing the importance of aligning message framing with behavioral stage and context. This would strengthen the scholarly contribution beyond programmatic implications.

Finally, the conclusion should be revised to temper causal language, avoid overgeneralization, clarify the limits of comparative framing claims, and acknowledge reliance on self-reported outcomes. A more balanced and cautiously framed conclusion will enhance the credibility and impact of the manuscript.

# Verdict: Major Revision Needed
