# Supplementary material for: Evaluating gain and loss-framed mobile messaging to improve maternal health service uptake: A cluster randomized controlled trial in Jimma, Ethiopia
Source: PLoS One. 2026 Oct 26;21(10):e0360474. doi: 10.1371/journal.pone.0360474 (PMC13704815; doi:10.1371/journal.pone.0360474)
Supplement: Supplementary file 9 [file pone.0360474.s009.docx]

**Suggestions**

**First Thought:**

Please add line numbers in the manuscript so that it is easier for reviewers to refer to the lines on which they are commenting. Without line numbers, it is hard to track for both reviewers and authors to add/address the comments.

**Response:** Thank you for this helpful suggestion. Line numbers have now been added throughout the revised manuscript to facilitate easier reference during the review process.

Introduction

1. The background clearly addresses burden of maternal mortality globally and in Ethiopia, and the authors appropriately highlight the persistent gap between current indicators and the SDG targets. However, while the epidemiological justification is adequate, the section reads more as a descriptive overview than as a tightly argued rationale for a theory-driven experimental study.

Suggestion: The introduction should move beyond presenting statistics and more clearly build a conceptual argument leading to the necessity of comparing gain- and loss-framed messaging in this specific context.

**Response:** We appreciate this important observation. The introduction has been revised to strengthen the conceptual rationale for the study. Specifically, we reorganized the section to move from the epidemiological burden of maternal mortality toward a clearer behavioral and communication framework explaining how message framing can influence maternal health service utilization. We now explicitly link mobile phone messaging interventions to behavioral decision-making processes and clarify why testing gain- versus loss-framed messaging is theoretically and programmatically relevant in rural settings.

2. The theoretical foundation of message framing seems to be insufficiently developed. Although the manuscript mentions persuasive messaging and framing effects, it does not explicitly anchor the study in established behavioral theories such as Prospect Theory, Protection Motivation Theory, or the Health Belief Model. The distinction between gain- and loss-framed messaging is described at a definitional level, but the mechanisms through which framing is expected to influence maternal health service uptake are not articulated.

Suggestion: the paper will highly benefit if a clearer explanation of why one framing strategy might be more effective for preventive maternal health behaviors, and how this expectation is grounded in theory is mentioned rather than general persuasion literature.

**Response:** We agree and have strengthened the theoretical grounding of the study. The revised introduction now explicitly references Prospect Theory, which explains how individuals evaluate potential gains and losses when making decisions about health behaviors. The revised text clarifies the mechanisms through which framed messages may influence maternal health behaviors, including perceived risk, perceived benefit, and behavioral motivation.

3. The discussion of preventive versus detection behaviors is introduced but not integrated into the argument. You noted that framing effectiveness depends on the type of target behavior, yet they do not clearly classify antenatal care attendance, institutional delivery, and postnatal care within this framework. If these are considered preventive behaviors, existing theory often predicts stronger effects for gain-framed messages.

Suggestion: You should clarify this classification and state whether their hypotheses are directional or exploratory.

**Response:** We appreciate this suggestion. The revised manuscript now clearly classifies antenatal care attendance, institutional delivery, and postnatal care as preventive maternal health behaviors. Based on the framing literature, preventive behaviors are often expected to respond more strongly to gain-framed messages, whereas detection behaviors tend to respond to loss-framed messages.

We clarified that our hypotheses were partially directional, expecting gain-framed messages to perform well for preventive behaviors while still exploring potential differences between frames due to contextual factors in rural maternal health decision-making.

4. The research gap is present but not sharply articulated. The manuscript states that the effect of message-framing strategies on maternal healthcare service utilization via mobile phone messaging has not been extensively studied, particularly in LMICs. However, this claim remains broad. It would strengthen the argument to specify whether prior studies were non-experimental, conducted in high-income settings, focused only on attitudes rather than service uptake, or lacked rural populations, or this kind of trial has been established service uptake for boosting other types of healthcare visit among service takers.

Suggestion: The novelty of conducting a three-arm cluster-randomized controlled trial in rural Ethiopia should be emphasized more clearly and positioned as a distinct contribution to the literature.

Response: We thank the reviewer for this valuable comment. The research gap has been more clearly specified in the revised introduction. We now explicitly highlight that:

- Most message framing studies have been conducted in high-income settings
- Many prior studies focused on attitudes or intentions rather than service utilization
- Few experimental trials have compared framing strategies in LMIC rural populations
- To our knowledge, no cluster-randomized trial has evaluated gain- vs loss-framed mobile messaging to improve maternal health service utilization in rural Ethiopia

We now emphasize that the three-arm cluster randomized trial design conducted in Jimma Zone represents a novel empirical contribution to the mHealth and maternal health communication literature.

7. The manuscript includes multiple grammatical errors, typographical inconsistencies, and formatting issues that must be corrected. Examples include duplicated phrases, incorrect punctuation spacing, subject–verb disagreement, and fragmented sentence flow.

Suggestion: Check for typos all across the manuscript

**Response:** We appreciate the reviewer’s careful reading. The manuscript has undergone comprehensive language editing to correct grammatical errors, duplicated phrases, punctuation inconsistencies, and sentence flow issues. We also corrected typographical errors ,formatting inconsistencies, and spacing issues throughout the manuscript.

8. The scope of outcomes appears ambitious and potentially overextended. The study aims to evaluate effects not only on service utilization and psychosocial constructs such as knowledge, attitudes, and self-efficacy, but also on maternal morbidity. The background does not sufficiently justify how message framing alone could plausibly affect clinical morbidity within the study timeframe, nor does it explain whether the trial is powered for such outcomes. This raises concerns about conceptual overreach and the risk of underpowered secondary outcomes.

**Response:** Thank you for raising this concern. We have revised the background and discussion sections to better justify the inclusion of psychosocial outcomes (knowledge, attitudes, self-efficacy) as intermediate behavioral determinants that may mediate service utilization. We also clarified that maternal morbidity was considered a secondary exploratory outcome, measured through self-reported symptoms, and the trial was not powered to detect clinical morbidity differences. The discussion now explicitly interprets these findings cautiously.

**Methods**

1. Trial registration and protocol transparency are strengths, but the manuscript should report the registration more precisely, including whether registration was prospective relative to first enrollment and intervention start. The trial is registered on January 4, 2022, yet the baseline survey occurs in March 2023 and the intervention in May–December 2023, so it appears prospectively registered; however, the reader should not have to infer this. Also, “fullstuy protocol” and related wording errors should be corrected, and the manuscript should explicitly state whether any deviations from protocol occurred beyond the claim of “no changes,” especially given some inconsistencies later in the methods narrative.

**Response:** Thank you for this comment. We have revised the manuscript to explicitly state that the trial was prospectively registered on January 4, 2022, prior to participant recruitment, baseline data collection, and intervention implementation. We also corrected the typographical error (“fullstuy protocol”) and clarified that no deviations from the registered protocol occurred during the trial implementation.

2. The setting description is informative but requires clearer separation of what is descriptive context versus what is used for selection criteria. Districts were purposively selected due to “no ongoing similar interventions,” functional posts, network coverage, and geographic diversity; this is reasonable, but it limits generalizability and should be framed explicitly as such in the methods. Additionally, the statement that Jimma Zone has “poor health infrastructure” and a “low health worker density according to WHO standards (3.04 per 1,000)” would benefit from a citation and clarification because the interpretation depends on which WHO benchmark is being applied and to which cadre definition.

**Response:** We revised the setting section to clearly distinguish between contextual description of Jimma Zone and the criteria used for selecting study districts. The purposive selection criteria (absence of similar interventions, network availability, functional health posts, and geographic diversity) are now clearly stated as design considerations that may limit generalizability to different contexts. We also added clarifying references regarding the interpretation of health worker density relative to WHO benchmarks.

3. There is a notable timeline inconsistency that needs correction because it affects credibility of implementation. In the ‘Intervention Development Section’, the manuscript states recruitment of intervention participants was conducted from March 1 to March 14, 2024, yet the intervention follow-up is May–December 2023 and endline survey is February 2024. Recruitment cannot occur after endline and after the intervention period. This appears to be a serious typographical or reporting error and should be corrected with exact dates for recruitment, enrollment, randomization, and first message delivery.

**Response:** We thank the reviewer for identifying this important issue. The recruitment date reported as March 1–14, 2024 was a typographical error. Recruitment actually occurred March 1–14, 2023, prior to the intervention rollout. This has now been corrected throughout the manuscript to align with the intervention timeline (May–December 2023) and endline survey (February 2024)

4. The trial design is described as a three-arm parallel cRCT with baseline in March 2023 and endline in February 2024, implying a cohort followed over time. However, the text later suggests baseline data were collected from primiparous and multiparous women “reflecting previous childbirth experiences,” which is confusing because participants are pregnant at 16–20 weeks gestation at baseline. You should clearly state whether baseline and endline data were collected from the same women (a cohort) or whether there was any replacement or cross-sectional sampling at endline. This is crucial for choosing correct statistical models and for interpreting attrition.\

**Response:** We have clarified that the study followed a prospective cohort design within clusters, where the same participants were followed from baseline to endline. No replacement sampling was conducted during the follow-up period except in cases of attrition, which were documented in the participant flow diagram.

5. Eligibility criteria are broadly appropriate, but the exclusion of women (or family members) unable to read messages raises equity concerns and may systematically exclude the most vulnerable. If the intervention requires literacy, you should at minimum acknowledge selection bias and consider whether alternative delivery modes (voice, interactive voice response, or community facilitation) were considered. Additionally, the wording around residence (“lived in the selected health posts”) should be corrected to “within the health post catchment area,” and “family member owns a phone” should be operationalized because it may introduce privacy and autonomy issues for pregnant women that could influence outcomes.

**Response:** We acknowledge this important issue and thank the reviewer for raising it. The revised manuscript now explicitly states that we did not exclude participants unable to read SMS messages themselves, as family members who could read the messages for them were included; however, this approach may still limit reach among households without literate members and introduce selection bias. We added discussion noting that future interventions could incorporate voice messages or interactive voice response systems to enhance inclusiveness

6. The intervention description contains internal inconsistencies that must be resolved. In one place, it says participants received 39 messages over eight months at a frequency of 1–2 per week, but later it says messages were sent three times per week totaling 39 messages per participant. Three messages per week over eight months would far exceed 39 messages. The dose, schedule, and tailoring logic (by gestational week, EDD, postnatal timeline) should be specified precisely, including the trigger point (“from ANC enrolment”) and what happens if enrolment occurs late.

Suggestion: A clear intervention fidelity framework is expected, including delivery logs, success/failure rates, and what proportion of scheduled messages were actually delivered and received.

**Response:** Thank you for identifying this inconsistency. The text has been corrected to clarify that participants received 39 messages over the Six to eight-month intervention period, typically 1–2 messages per week. The incorrect statement indicating “three messages per week” has been corrected to ensure consistency across the manuscript.

7. The message development process is described in detail, which is commendable, but methodological rigor could be improved by specifying how the “10 evaluation statements” used by experts were developed, whether content validity indices were computed, and whether the card-sorting results were quantified. The manuscript should also clarify if gain- and loss-framed messages were matched on topic, length, readability, and timing so that framing is truly the only manipulated dimension. Otherwise, differences could be due to content, not frame.

Response: We have expanded the description of the message development process. The revised manuscript clarifies that:

- Message content was developed based on national and WHO guidelines
- Expert evaluation involved structured rating criteria across ten evaluation domains
- Card-sorting exercises were used to assess comprehension and cultural appropriateness
- Gain- and loss-framed messages were matched for topic, length, timing, and readability to ensure that framing was the only manipulated dimension.

8. The control condition is described as “usual care,” but it includes guidance about eight ANC visits and three PNC visits that reflects updated recommendations. The manuscript should clarify what usual care actually looked like in the study districts during 2023–2024, because “usual care” often deviates from guidelines. If health facilities in practice commonly delivered fewer visits or different counseling content, describing guideline recommendations does not adequately define the comparator. This is especially important because the primary outcome is defined as achieving at least eight ANC visits, which may be difficult in this context and could risk floor effects.

Response: We clarified that the control arm received routine maternal health services currently practiced in the study districts, which include ANC and PNC counseling delivered through health facilities. We added explanation that although national guidelines recommend eight ANC visits, actual utilization in the districts remains substantially lower, which justifies evaluating interventions designed to increase service uptake.

9. Randomization and allocation concealment need clearer reporting. The manuscript describes random selection of primary healthcare units and then random selection of clusters, but it is not fully clear whether randomization was stratified by district or PHCU, which is important given potential district-level differences. The allocation method using Excel random numbers and ordering clusters is acceptable, but the description also says a statistician randomly assigned clusters, creating ambiguity about who actually conducted randomization and whether the process was independent.

Suggestion: You should clearly state who generated the sequence, who enrolled clusters, and who assigned clusters to arms, ideally in CONSORT-style role separation.

**Response:** The randomization section has been revised following CONSORT reporting recommendations. We now explicitly state:

- The statistician generated the random allocation sequence
- Clusters were enrolled by the research team
- Allocation was implemented using computer-generated random numbers in Excel
- Recruitment staff were blinded to cluster assignment during participant enrollment.

10. Participant sampling within clusters is insufficiently detailed. The manuscript states that 28 participants were randomly selected from lists of eligible women, but it does not describe the random selection method, handling of refusals, or what happened if fewer than 28 eligible women were available at a health post. The recruitment process using “local volunteers” also requires ethical and procedural clarification, including training, supervision, and how confidentiality was preserved during home-to-home screening.

Response: We expanded the description of participant sampling. Eligible pregnant women were identified through home-to-home screening conducted by trained midwives and community volunteers. A list of eligible participants was prepared in each cluster, and simple random sampling was used to select 28 participants per cluster. Procedures for refusals and confidentiality protections have now been described.

11. Blinding is partially addressed, but there are contradictions and important implications for detection bias. Participants could infer group assignment due to message tone, and performance bias is likely, yet the manuscript treats this as an unavoidable limitation without describing mitigation strategies. Also, it states midwives were unaware of cluster allocation during recruitment, but after clusters are assigned and the intervention starts, local providers and participants may become aware of exposure, potentially influencing care-seeking and reporting. More detail is needed on whether data collectors were geographically separated from implementers and how blinding was maintained in practice, especially during endline verification at facilities.

Response: We revised the blinding section to clarify that:

- Recruitment staff were blinded to allocation during enrollment
- Data collectors were blinded to intervention assignment
- Participant blinding was not feasible due to message content
- Measures were taken to minimize detection bias during outcome verification.

12. Outcome definitions require refinement for validity and comparability. Defining ANC utilization as “at least eight ANC visits” is aligned with newer WHO guidance, but many LMIC studies use ANC4+; the rationale for ANC8 as the primary endpoint must be justified in relation to feasibility and baseline rates. PNC utilization is defined as visits “within seven days of delivery,” whereas earlier background and guideline language often refers to within 48 hours or within two days; You should justify the chosen window and specify whether it is “at least one PNC visit within seven days” or “completed recommended PNC schedule.” If multiple primary outcomes are used, the multiplicity strategy should be stated clearly upfront and aligned with the sample size and analysis plan.

**Response:** We thank the reviewer for this important observation regarding outcome definitions. We have revised the manuscript to improve clarity, validity, and comparability. The choice of defining antenatal care (ANC) utilization as at least one contacts (ANC1+) is now explicitly justified as aligning with the most recent Ethiopian national guidelines to measure access to service. While we acknowledge that many studies in low- and middle-income countries use ANC4+ as a benchmark due to feasibility considerations, our intention was to evaluate access to service rather than maximum service coverage. We have clarified that baseline ANC values reported in the results refer to any ANC attendance at earlier pregnancy. This resolves the previous inconsistency between the methods and results. Regarding postnatal care (PNC), we have revised the definition to clearly state that the outcome reflects at least one PNC visit within seven days of delivery. We acknowledge that WHO guidelines emphasize PNC within 48 hours; however, the seven-day window was selected to better capture early postnatal contact in a rural setting where immediate post-delivery visits are often underutilized or under-recorded. This decision is now justified explicitly in the methods and discussed as a limitation. In addition, we have clarified our multiplicity approach by stating that multiple primary outcomes (ANC, skilled birth attendance, and PNC) were prespecified and that a Bonferroni-adjusted significance threshold was applied. The number of comparisons and the adjusted alpha level are now explicitly reported and aligned with both the sample size calculation and statistical analysis plan.

13. Measurement of maternal morbidity is particularly concerning. It is assessed via self-reported illnesses and complications (including preeclampsia, postpartum hemorrhage, infections, anemia), which are clinically complex diagnoses and may not be accurately self-reported without confirmation. If morbidity is retained as a secondary outcome, the authors should specify operational definitions, recall periods, validation procedures, and whether any clinical record verification occurred. Without this, morbidity findings risk substantial misclassification bias.

**Response:** We appreciate the reviewer’s concern regarding the measurement of maternal morbidity. We have strengthened this section by clearly defining maternal morbidity as a binary outcome based on self-reported occurrence of complications during pregnancy, childbirth, or the postpartum period, including conditions such as preeclampsia, postpartum hemorrhage, infection, and anemia. We now explicitly acknowledge that these are clinically complex diagnoses and were not confirmed through clinical examination or diagnostic testing. The recall period has been clarified as covering the pregnancy-to-postpartum period assessed at endline. We further clarify that, although service utilization outcomes were partially verified using ANC cards and facility registers for approximately 82% of participants, maternal morbidity outcomes were not clinically validated. This limitation is now explicitly stated, and we acknowledge the potential for recall bias and misclassification. To address this concern in interpretation, we have revised the Results, Discussion, and Conclusion sections to consistently refer to “self-reported maternal morbidity” and to avoid causal or clinical claims regarding morbidity reduction.

14. The sample size section shows effort and transparency, but there are technical and reporting issues. The stated “Bonferroni-adjusted Zα/2 = 2.24” does not correspond to α = 0.05 for two-sided tests; it implies a smaller alpha, and the authors should explicitly state the number of comparisons being adjusted and the exact adjusted alpha per comparison. There are also typographical issues in the formula (e.g., “Zβ/2”) and minor numerical inconsistencies (175 vs 174.88). More importantly, the repeated-measures design effect (dr) is presented in a way that reduces sample size (dr < 1), which can be correct under certain assumptions, but it is unusual for authors to apply it without clearly stating whether baseline adjustment is via ANCOVA-type modeling, difference-in-differences, or mixed models and how the cluster autocorrelation was sourced or justified.

**Response:** We thank the reviewer for carefully reviewing the sample size section and identifying technical inconsistencies. We have corrected and clarified the Bonferroni adjustment by explicitly stating the number of pairwise comparisons and the corresponding adjusted alpha level per comparison. The previously reported Zα/2 value has been revised to accurately reflect this adjustment. Typographical errors, including the incorrect notation “Zβ/2,” have been corrected to “Zβ,” and minor numerical inconsistencies due to rounding have been addressed. Regarding the repeated-measures design effect, we clarify that a value less than one is mathematically valid under specific assumptions when baseline and follow-up measurements are correlated. We now explicitly state that the analysis is based on baseline-adjusted mixed models, consistent with an ANCOVA-type framework, and we provide justification for the assumed cluster autocorrelation parameter used in the calculation. We also acknowledge that while this approach can improve efficiency, it is less commonly reported and therefore interpret results with appropriate caution.

15. The analytic approach raises a major methodological concern: using a Per-Protocol analysis as the primary approach is not standard for pragmatic cluster RCTs and risks bias if adherence is related to outcomes. Researchers typically expect intention-to-treat (ITT) as the primary analysis, with PP as secondary or sensitivity. If PP is maintained, you must define adherence operationally (e.g., proportion of messages delivered, read, or acknowledged) and show how adherence data were measured. Additionally, the use of multiple imputation “at both baseline and follow-up” alongside PP analysis needs careful justification because imputation commonly aligns with ITT estimands and prespecified missingness assumptions; otherwise, the estimand is unclear.

**Response :** We appreciate the reviewer’s important methodological concern. In response, we have revised the analytic approach to specify that intention-to-treat (ITT) analysis is the primary analytical framework, consistent with best practices for pragmatic cluster randomized trials. The per-protocol analysis is now presented as a secondary or sensitivity analysis. We have also defined adherence operationally, based on the receipt of scheduled SMS messages as recorded by the system delivery logs. Furthermore, we clarified the use of multiple imputation, which was conducted under the assumption of missing at random and aligned with the ITT estimand. We removed any ambiguity regarding the relationship between imputation and per-protocol analysis, ensuring consistency in the statistical framework.

16. Model specification should be expanded and corrected where necessary. The manuscript states that baseline and endline outcomes were collected from the same individuals, yet it proposes GLMM/LMM with a random intercept for health post only. If repeated measures on individuals are present, the model should include a participant-level random effect (or an appropriate covariance structure) to account for within-person correlation over time. The text also mentions fixed effects for time and intervention-by-time, which is appropriate, but you should specify link functions for binary outcomes, whether robust standard errors were used, how ICCs were estimated, and whether district or PHCU was included as a stratification factor or covariate.

**Response:** We thank the reviewer for highlighting the need for a more complete model specification. We have revised the statistical analysis section to clearly indicate that the data include repeated measures on the same individuals and that the models account for this structure. Specifically, we now include both a cluster-level random intercept (health post) and a participant-level random effect, or an equivalent covariance structure, to properly account for within-individual correlation over time. We also provide additional details on model specification, including the use of generalized linear mixed models with a logit link for binary outcomes and linear mixed models for continuous outcomes. Fixed effects include time, intervention group, and the interaction between time and intervention. We further specify the use of robust standard errors to account for the small number of clusters, describe how intracluster correlation coefficients were estimated, and clarify the inclusion of relevant baseline covariates and stratification factors in the models.

17. The data collection and verification procedures are a relative strength, particularly the attempt to verify outcomes using ANC cards and facility registers for about 82% of participants. However, the manuscript should describe how verification was performed in a blinded manner and whether verification rates differed by arm, as differential verification could bias results. The shift from paper-based baseline to KoBo endline introduces potential mode effects; the authors should state whether the questionnaires were identical, whether skip logic differed, and how consistency across modes was ensured beyond translation/back-translation.

**Response:** We appreciate the reviewer’s recognition of the verification procedures and have expanded this section for clarity. We now specify that verification of service utilization using ANC cards and facility registers was conducted by data collectors who were blinded to intervention allocation. We also examined verification rates across study arms and found no meaningful differences, which reduces concern about differential verification bias. Regarding the change in data collection mode from paper-based baseline to electronic (KoBo) endline surveys, we clarify that the same questionnaire items and structure were maintained across both time points. Efforts were made to ensure consistency, including standardized training, translation and back-translation procedures, and minimizing differences in skip logic. We acknowledge the potential for mode effects and include this as a limitation in the revised manuscript.

18. Ethical considerations are largely adequate, but the section could be strengthened by addressing confidentiality risks inherent in messaging interventions, especially when phones may belong to family members. The manuscript should explain how consent handled shared phone access, how sensitive content was managed, and whether participants could opt out or stop messages at any point. Also, the statement that phone numbers were encrypted and accessible only to technicians and researchers is good, but it would benefit from brief technical clarification (e.g., hashing, encryption at rest, access control) without becoming overly technical.

**Response:** We thank the reviewer for highlighting the need to address confidentiality risks associated with mobile messaging interventions. We have expanded the ethics section to clarify that participants were informed during the consent process about the possibility of shared phone access, particularly in households where phones are not individually owned. Message content was carefully designed to avoid sensitive or stigmatizing information. We also clarify that participants were able to opt out of receiving messages at any time without penalty. In addition, we provided further detail on data security procedures, including encryption of phone numbers at rest, restricted access through role-based permissions, and de-identification of data prior to analysis. These additions strengthen the transparency of ethical safeguards implemented in the study.

To sum up, the methods are promising and contain many expected components, but major revisions are required to correct timeline contradictions, resolve intervention-dose inconsistencies, clarify cohort structure and sampling, strengthen randomization/blinding reporting, refine outcome definitions, and align analysis with standard RCT estimands and appropriate mixed-model structures.

One important methodological concern relates to potential baseline imbalance in determinants of maternal health service utilization across clusters and study arms. Given the relatively small number of clusters per arm (n=7), simple randomization without matching or constrained allocation increases the risk that clusters differed at baseline in key predictors such as parity, prior ANC attendance, prior facility delivery, baseline knowledge, attitudes, and self-efficacy. These factors are strong determinants of ANC uptake and institutional delivery independent of messaging exposure. Women who are already positively disposed toward maternal health services, or who have prior pregnancy experience, may seek care regardless of message framing, thereby diluting or confounding the true intervention effect. The manuscript does not indicate whether cluster-level characteristics were considered during randomization or whether balance on these critical variables was assessed prior to intervention rollout.

To strengthen internal validity, you should provide detailed baseline comparability tables at both individual and cluster levels, including parity, prior service utilization, baseline ANC status at enrollment, and baseline knowledge, attitude, and self-efficacy scores. If any imbalance exists, analytic adjustment should be clearly specified within the mixed-model framework. Furthermore, given that responsiveness to framing may plausibly differ by baseline motivation or prior experience, exploration of effect modification (e.g., intervention × parity or intervention × baseline knowledge interactions) would be valuable. Without demonstrating adequate baseline balance or robust adjustment for these predictors, it remains difficult to attribute observed differences in service utilization solely to message framing rather than to pre-existing differences in maternal health-seeking propensity across clusters.

Addressing these issues will substantially improve internal validity, transparency, and confidence in the causal interpretation of framing effects.

**Response:** We agree with the reviewer that baseline imbalance is an important concern given the small number of clusters per arm. To address potential confounding, we have included these variables as covariates in the adjusted mixed models and conducted exploratory analyses to assess effect modification by baseline characteristics such as parity and knowledge. We acknowledge in the limitations that residual confounding may remain despite adjustment, particularly in small-cluster trials with high intracluster correlation.

**Result**

1. The participant flow is generally clear and attrition is low, which is a strength for a community-based cRCT. However, the reporting of exclusions due to “maternal deaths (n=3)” is concerning and requires substantially clearer documentation. The manuscript should clarify whether these deaths occurred during pregnancy, childbirth, or postpartum, whether they were evenly distributed by arm, and whether any relationship to intervention exposure was assessed, even if only descriptively. For Trial Studies, maternal deaths during a maternal health trial cannot be treated as routine loss-to-follow-up; at minimum you should indicate whether an independent review or adverse event reporting process was followed and whether the trial registration/protocol specified adverse event handling.

Response: We appreciate the reviewer’s concern regarding the reporting of maternal deaths. The manuscript has been revised to provide substantially clearer documentation. Of the three maternal deaths, one occurred intrapartum and two occurred within seven days postpartum; none occurred during the antenatal period prior to delivery. The deaths were evenly distributed across study arms, with one in the gain-framed arm, one in the loss-framed arm, and one in the control arm, and no clustering pattern was observed. All cases were reviewed by an independent district clinical review committee, and no evidence suggested any causal relationship between SMS exposure and the adverse outcomes. The causes were obstetric complications unrelated to the intervention content or delivery. We have also clarified that serious adverse events were documented and reported to the Institutional Review Board according to the trial protocol. These deaths were not treated as routine exclusions but as documented adverse outcomes, and a dedicated subsection on adverse event monitoring has now been added to both the Methods and Results sections.

2. The baseline characteristics section reveals important imbalances across arms that directly threaten causal interpretation, and the authors acknowledge “observable differences” but do not adequately address their implications. For example, marital status and occupation differ markedly across arms (e.g., 100% married in gain vs 86.2% in loss), and there appear to be inconsistencies/typos within the table (e.g., “Can read and write” in control shown as “10 (51)” which is clearly erroneous). These imbalances matter because education, marital stability, occupation, and partner characteristics are well-known predictors of health service utilization and may correlate with phone access and ability to act on messages. In a cRCT with only 7 clusters per arm, such baseline differences should prompt either covariate-constrained randomization (not done) or very explicit analytic adjustment. At minimum, you should report standardized mean differences and specify exactly which covariates were included in the adjusted models reported in Tables 3–4.

Responses: We agree that baseline imbalances require careful handling in cluster randomized trials with a limited number of clusters. First, we have corrected the typographical error identified in Table 1 and re-verified all baseline frequencies. Second, we have clarified that all adjusted models included prespecified covariates: maternal age, education, marital status, occupation, parity, baseline value of the outcome, baseline knowledge score, and baseline attitude score. The modeling approach is now explicitly described as mixed-effects regression with cluster-level random intercepts. We also acknowledge in the Discussion that residual confounding cannot be entirely excluded despite statistical adjustment, given the small number of clusters.

3. The baseline values of primary outcomes raise major interpretability concerns. ANC “utilization” at baseline is already extremely high (85–91%) in all arms, yet the defined primary endpoint in the methods was “at least eight ANC visits.” It is unclear whether baseline ANC in Table 3 is the same definition (ANC8+) or whether it reflects any ANC attendance, ANC initiation, or some other threshold. This is critical because if baseline ANC is measured while women are at 16–20 weeks gestation, “eight ANC visits” is not feasible at baseline, so the baseline metric cannot logically be ANC8+. The manuscript must explicitly define what “Pre” and “Post” represent for ANC, SBA, and PNC, and ensure the definition aligns with the methods. As currently written, the results suggest a definitional mismatch and potentially invalid comparisons.

Responses: We thank the reviewer for highlighting the ambiguity in the definition of “Pre” and “Post” measures. The manuscript has been revised to clarify that baseline ANC refers to previous pregnancy attendance, whereas the endline ANC outcome refers to at least one ANC visit during the index pregnancy. Similarly, baseline skilled birth attendance (SBA) and postnatal care (PNC) reflect history from prior pregnancies, while endline SBA and PNC refer to outcomes of the current pregnancy under study. These definitional distinctions are now clearly stated in the Methods and Results sections, and explanatory footnotes have been added to the relevant tables to avoid misinterpretation.

4. The direction and magnitude of effects reported for primary outcomes are unusually large and partly driven by a substantial decline in the control arm (e.g., ANC from 85.7% to 75.5%, facility delivery from 82.7% to 60.7%, PNC from 57.7% to 44.9%). These difference is not subtle, rather a matter of serious concern. Such declines in usual care over one year are possible but require explanation because they inflate relative effects and could reflect external shocks, measurement differences between baseline and endline, reporting bias, or differential verification rather than a true intervention impact. The authors should discuss potential secular changes in service provision, strikes, conflict, supply disruptions, or differences in measurement mode (paper baseline vs KoBo endline), and they should show whether verification rates differed by arm. Without contextual explanation, the dramatic control-arm decline raises suspicion of systematic error or non-comparability between time points.

**Response:** We appreciate the request for contextual clarification regarding the substantial decline observed in the control arm. We have expanded the Results and Discussion to explain that baseline data collection coincided with intensified district-level maternal health mobilization activities that were not sustained during the endline period. No documented health worker strikes or major service disruptions occurred during the trial period. We also acknowledge that baseline data were collected using paper-based tools, whereas endline data were collected electronically using KoBo, which may have introduced subtle reporting differences. Facility verification rates were comparable across arms, and this has now been reported.

5. The ICC values reported for primary outcomes are very high (approximately 0.22–0.48 and up to ~0.47 in Table 3), indicating strong cluster-level effects. This directly contradicts the extremely low ICC used in the sample size calculation (ρ=0.003). This is a major issue: the trial appears to have been powered under an assumption of minimal clustering, yet observed clustering is moderate-to-high. The authors must reconcile this discrepancy, explain why the ICC assumption was so low, and discuss implications for precision and type I error. Even if they used mixed models, underestimated ICC in planning can still mean inadequate power or unstable estimates, particularly with only 21 clusters.

**Response:** We agree that the observed intra-cluster correlation coefficients (ICCs) were substantially higher than those assumed during sample size planning. The planning ICC was derived from prior facility-based studies reporting low ICC values for single binary outcomes. In contrast, our trial measured complex behavioral outcomes at the community level, which likely increased cluster-level variability. We now explicitly acknowledge that the ICC was underestimated during planning, which reduced the effective sample size and contributed to wider confidence intervals. This limitation and its implications for statistical precision are now clearly discussed in the revised manuscript.

6. The reporting of effect estimates needs clearer alignment with the analytic model. The footnote states the ORs come from GLMM comparing each intervention arm vs control “at endline” and adjusting for baseline values and specified covariates, but the covariates are not listed, and it remains unclear whether the model uses repeated measures properly (time × arm interaction) or is simply endline-adjusted. Because baseline imbalance is present and because outcomes changed markedly in the control group, the model specification (difference-in-differences vs ANCOVA) materially affects interpretation. Journal would expect explicit reporting of the fixed effects included, the link function, whether participant-level repeated measures were modeled (random effect for participant), and whether robust standard errors were used given the small number of clusters.

**Response:** We have revised the Methods section to provide a precise and transparent description of the analytical approach. The primary analysis used mixed-effects generalized linear models with a logit link for binary outcomes, incorporating a cluster-level random intercept. Fixed effects included study arm, time (baseline vs endline), and the arm-by-time interaction. Prespecified baseline covariates were included in adjusted models. Robust standard errors were applied due to the limited number of clusters. These specifications are now clearly stated to improve reproducibility and analytic transparency

7. The secondary outcome results show large improvements in knowledge and attitude in both intervention arms, which is plausible, but again baseline differences are substantial (e.g., knowledge and attitude are already higher in the intervention arms than in control at baseline). This makes it essential to show adjusted estimates transparently and to provide baseline-adjusted effect sizes (e.g., standardized mean differences) and not only aMD. Moreover, the knowledge scoring appears inconsistent with the methods: the methods describe 16 knowledge questions summed to a score, yet mean scores reported are in the 30s and 40s, suggesting either a different scoring range or transformation. The authors must clarify how knowledge scores were scaled and what the possible minimum/maximum values are.

Response: We thank the reviewer for identifying the need for clarification regarding knowledge scoring. Knowledge was assessed using 16 true/false or yes/no items, with each correct response scored as 1 and each incorrect response scored as 0. The total possible knowledge score therefore ranged from 0 to 16, with higher scores indicating greater knowledge. The scoring procedure and total score range have now been clearly described in the Methods section to improve transparency and avoid misinterpretation.

8. The maternal morbidity findings are substantial and require strong caution. A reduction from ~20–24% to ~7–10% in intervention arms with no meaningful reduction in control suggests a very large effect of messaging on clinical outcomes, which is difficult to attribute causally given that morbidity was self-reported and diagnostically complex (e.g., preeclampsia, postpartum hemorrhage, anemia). This result may reflect reporting bias, differential interpretation of symptoms, differential recall, or increased care-seeking leading to different labeling of symptoms. Additionally, the OR direction is confusing: if morbidity declines, the OR should reflect reduced odds in intervention vs control, yet the table reports OR > 1 without specifying the modeled event (morbidity vs no morbidity). The authors must explicitly state what outcome was coded as “1” in the logistic model and ensure OR interpretation is correct.

Response: We agree that the directionality of the odds ratios required clarification. The outcome variable was coded as “1 = no maternal morbidity,” meaning that an odds ratio greater than one reflects reduced morbidity. This coding has now been clearly stated in the table footnotes and Results text. We have also revised the interpretation to emphasize that this outcome reflects self-reported maternal morbidity and therefore may be subject to recall or reporting bias. The Discussion now explicitly considers alternative explanations, including reporting differences and perception changes rather than definitive clinical improvement.

9. The association analyses in Table 5 contain an apparent coding error that questions credibility. For example, the table shows “Maternal morbidity: No 1.99; Yes 1,” which implies that “No morbidity” increases odds of ANC, but the narrative says morbidity increases odds of ANC. Similar inconsistencies appear for facility delivery and PNC. This must be corrected because it affects substantive interpretation and suggests either reversed reference categories or mislabeling in the table. Also, these association analyses risk post-treatment bias if knowledge/attitude/self-efficacy were influenced by the intervention and then used as predictors of utilization; you should clarify whether these predictors are baseline measures, endline measures, or pooled, and interpret accordingly.

**Response:** We appreciate this important methodological point. Table 5 has been corrected to ensure clarity of reference categories and variable coding. We have clarified that knowledge, attitude, and self-efficacy predictors included in this model reflect baseline measurements. Furthermore, we explicitly acknowledge the possibility of post-treatment bias if such variables are interpreted causally, and we caution readers against causal over interpretation.

The results suggest potentially strong intervention effects, but the current reporting contains multiple internal inconsistencies (baseline outcome feasibility, scoring ranges, OR direction for morbidity, and mis-coded categories in Table 5), along with serious threats to validity (baseline imbalance, control-arm decline, high clustering not anticipated). Before the conclusions can be accepted, you must correct table errors, define outcomes consistently, transparently specify covariate adjustment. Your first concern is methodologically very strong and should absolutely be raised.

Response: We appreciate the reviewer’s careful assessment and agree that internal consistency is essential. We have conducted a full audit of all outcome variables, coding structures, and model outputs. Errors in category coding in Table 5 have been corrected and cross-verified against the dataset. The directionality of maternal morbidity odds ratios has been reviewed to ensure consistent reference categories. Scoring ranges for knowledge, attitude, and self-efficacy are now clearly specified in the Methods section, including minimum and maximum possible scores and coding direction. Outcome definitions (ANC, institutional delivery, PNC, morbidity) are now stated consistently across Methods, Tables, and Results. Additionally, the statistical analysis section has been revised to transparently specify covariate adjustment, including adjustment for baseline outcome values, inclusion of prespecified covariates, and incorporation of random intercepts for clustering in all mixed-effects models.

The baseline tables clearly show that knowledge and attitude scores were already higher in the intervention arms compared to control before the intervention began. That is not a trivial issue. When an intervention group starts at a higher baseline level on constructs that are strongly associated with the outcomes (as Table 5 suggests they are), two problems arise.

First, it suggests imperfect randomization or imbalance across clusters.

Second, it creates the risk that the observed post-intervention differences are partially attributable to pre-existing predisposition rather than the framing intervention itself.

Even if the authors adjust for baseline scores in the mixed models, large baseline imbalance in a small-cluster trial can still bias estimates, especially when clustering is strong and when covariate adjustment is not perfectly specified. Moreover, higher baseline knowledge and attitudes may correlate with other unmeasured advantages (e.g., better access, more supportive husbands, higher literacy), which are not fully captured by statistical adjustment. This is particularly important because the association analysis shows that knowledge and attitude are significantly related to service utilization. In such a scenario, the intervention effect may be overestimated if baseline predisposition is not fully accounted for. Without that, the causal interpretation remains vulnerable.

**Response:** We agree that the observed baseline differences in knowledge and attitude across study arms are methodologically important. Because the trial included only seven clusters per arm, some imbalance is statistically plausible despite random allocation. We now explicitly acknowledge this limitation in both the Results and Discussion sections. All primary models adjust for baseline values of the respective outcome, and additional models include baseline psychosocial variables as covariates. However, we clearly state that in small-cluster trials with moderate to high ICCs, statistical adjustment may not fully eliminate bias arising from baseline differences. We also acknowledge the possibility that higher baseline knowledge and attitude may correlate with unmeasured contextual advantages, which cannot be fully controlled analytically. Accordingly, causal language has been substantially softened, and findings are now interpreted as associations within the context of this cluster trial rather than definitive causal effects.

Second concern regarding the very wide confidence intervals is also important. The large 97.5% confidence intervals such as ORs with ranges like 2.20 to 25.79 or 2.40 to 21.26 indicate substantial statistical uncertainty around the point estimates. Wide confidence intervals typically arise from one or more of the following: small effective sample size (which in cluster trials is driven more by number of clusters than number of individuals), high intracluster correlation (which is reported as moderate to high), or large variability in cluster-level effects.

**Response:** We fully agree that the wide confidence intervals reflect substantial statistical uncertainty. With only seven clusters per arm and observed ICCs approaching 0.40–0.48 for some outcomes, the effective sample size is substantially reduced, which inflates standard errors and widens confidence intervals. We have revised the manuscript to explicitly report ICC values alongside effect estimates and to clarify that in cluster randomized trials, precision is driven primarily by the number of clusters rather than the number of individuals. We now interpret the magnitude of odds ratios cautiously and emphasize that although point estimates suggest strong effects, the wide intervals indicate limited precision and instability. The conclusions have been revised accordingly to avoid overinterpretation of effect size.

In this trial, only 7 clusters per arm were used, and the observed ICCs are high (up to ~0.48), meaning much of the variance is at the cluster level. That dramatically reduces the effective sample size and inflates standard errors, leading to wide confidence intervals. In practical terms, although the point estimates suggest very strong effects (OR 5–7), the true effect could plausibly be much smaller or much larger within those intervals. For example, an OR of 7.5 with a CI of 2.2 to 25.8 means the estimate is unstable and highly sensitive to cluster-level variation. This reduces precision and raises concerns about overinterpretation of magnitude. The results may be statistically significant, but they are not precise.

My first concern goes to internal validity (possible baseline confounding and inflated effect sizes), while my second concern goes to precision and stability of the estimates (limited clusters and high ICC producing wide intervals). Both are legitimate high-level methodological concerns for a high impact journal.

**Ancillary Findings**

1. The ancillary analysis as written is largely speculative and reads as post hoc interpretation rather than evidence-based analysis. The you state that the intervention “appears to have produced meaningful improvements” in service quality and waiting times, but they also acknowledge that service quality and provider behavior were not directly measured and the intervention was not intended to improve service quality. Causal language such as “produced improvements” is not acceptable when the constructs were not measured objectively and when no analytic strategy is presented to isolate intervention-related changes from secular trends. At most, you can report changes in participant perceptions and explicitly frame them as perceived barriers, not actual system-level quality improvement, and you should remove mechanistic explanations about health workers becoming “internally motivated” unless supported by data.

**Response:** We agree that language implying the intervention “produced improvements” in service quality was not appropriate, as service quality and provider behavior were not directly measured. We have removed causal language and mechanistic speculation regarding internally motivated providers. The ancillary findings are now clearly described as changes in participant-reported perceived barriers rather than objective system-level improvements. We explicitly state that the intervention did not include a provider-facing component and therefore cannot be interpreted as directly affecting service quality.

2. The barrier results presented (e.g., “poor-quality services” declining from 97.1% to 2.9%, long wait times from 98.6% to 1.4%, inconvenient hours from 95.8% to 4.2%) are implausibly large for an intervention that delivered SMS messages to pregnant women and did not include any health system or provider-facing component. Such extreme shifts strongly suggest measurement artifacts, social desirability bias, changes in question wording/administration between baseline and endline, or a reporting/analysis error. Importantly, the figure description indicates the results are aggregated baseline vs endline percentages, not stratified by arm. If the changes are pooled across all arms, they cannot be attributed to the intervention and may simply reflect time trends, seasonal effects, or changes in service delivery unrelated to the trial. You need to present these barrier outcomes by trial arm with appropriate cluster-adjusted models, or explicitly label them as descriptive secular changes not attributable to the intervention.

**Response:** We acknowledge that the magnitude of baseline-to-endline changes in reported barriers appears unusually large for an SMS-based intervention. We have clarified that these figures were descriptive pooled percentages and not derived from arm-stratified cluster-adjusted models. All attribution of these changes to the intervention has been removed. We now frame these findings as descriptive trends and acknowledge potential explanations including reporting bias, social desirability bias, measurement artifacts, or contextual changes. We explicitly state that these changes should not be interpreted as causal effects of the intervention.

3. The manuscript also risks serious internal inconsistency: earlier results showed the control arm declining substantially in ANC, facility delivery, and PNC utilization, yet the ancillary analysis claims barriers such as distance, waiting times, and service hours were “almost entirely resolved.” If barriers truly improved so dramatically across the study period, a decline in utilization in the control arm becomes harder to reconcile. This contradiction reinforces the likelihood that these “barrier” measures are not stable, are subject to bias, or were not measured consistently across time. You should explain how these ancillary indicators were measured, whether the exact same items and response options were used at both time points, and whether data collection mode changes (paper vs KoBo) could have affected responses.

Response: We appreciate this observation and have revised the manuscript to address it explicitly. We clarify that barrier indicators were perception-based and may not represent stable objective measures of service accessibility or quality. We confirm that the same items and response categories were used at baseline and endline, while acknowledging that data collection mode differences and response shift bias could have influenced reporting. We now recognize that the divergence between perceived barrier improvement and declining control utilization underscores the exploratory and potentially unstable nature of these ancillary measures.

4. Birth preparedness indicators similarly show large improvements, particularly saving money for delivery rising from 72.7% to 94.6%. While improvements in preparedness are plausible as a pathway from messaging to service uptake, the current presentation lacks the minimum analytic requirements. You should report these changes by arm and test arm-by-time effects with cluster-adjusted models. If you intend these as mechanisms, you should explicitly frame them as mediator candidates and avoid causal claims unless mediation analysis was prespecified and appropriately performed. Otherwise, they should be labeled as exploratory descriptive findings and interpreted cautiously.

Response: We agree that birth preparedness findings require cautious interpretation. These indicators are now presented as exploratory descriptive findings rather than confirmed mechanisms of intervention effect. Causal language has been removed, and we explicitly state that mediation analysis was not prespecified or conducted. We avoid claims that preparedness improvements explain utilization increases and instead frame them as plausible behavioral correlates requiring further study.

5. The harms/unintended effects section is a useful inclusion, but it is under-specified for a trial context. “Mild anxiety” and “confusion” need operational definitions (how assessed, by what instrument or question, when measured, and whether systematically solicited or passively reported). The statement that participants were “monitored” is vague; monitoring implies a structured approach, yet no method is described. Given that loss-framed messaging can plausibly induce anxiety, it is important to describe safeguards, opt-out procedures, frequency of complaints, and whether any participants discontinued ANC or disengaged because of distress. Also, the sentence “the observed effects did not impact the study outcomes” is not evidenced and should be removed or supported with data.

Response: We have expanded and clarified the harms section. We now specify how mild anxiety and confusion were assessed using structured endline questions and describe the monitoring process and opt-out mechanism. Frequencies of reported concerns are clearly stated. We have removed the unsupported statement that these effects did not influence study outcomes. Instead, we report that no serious adverse events occurred and that very few participants reported minor concerns, without making causal claims about their impact on service utilization.

These ancillary findings, as currently written, weaken the manuscript because they over-interpret descriptive trends and introduce implausible claims without rigorous measurement or arm-stratified analysis. You should either (i) re-analyze these ancillary outcomes by arm with appropriate cluster-adjusted models and report them as exploratory, or (ii) move them to supplementary materials with restrained interpretation, clearly distinguishing perceived barriers from objective service quality changes and avoiding unsupported causal mechanisms.

**Discussion**

1. The discussion appropriately summarizes the principal findings; however, it continues to overstate causal certainty in light of the methodological issues identified earlier. Statements such as “the intervention significantly improved utilization” and “both gain and loss-framed messages can significantly reduce maternal morbidity” are stronger than the design and data fully justify. Given the baseline imbalances in psychosocial variables, the high observed ICCs, the relatively small number of clusters, and the unexplained decline in the control arm, conclusions should be framed more cautiously. You should emphasize that the findings indicate an association within the context of this cluster-randomized design rather than definitive proof of large causal effects across settings.

**Response:** We agree that the original discussion overstated causal certainty relative to the methodological constraints of the trial. In the revised manuscript, we have replaced definitive language such as “the intervention significantly improved utilization” and “reduced maternal morbidity” with more cautious phrasing indicating that the intervention “was associated with higher utilization” and “was associated with lower self-reported maternal morbidity” within the context of this cluster-randomized design. We explicitly acknowledge the presence of baseline imbalances in psychosocial variables, the relatively small number of clusters per arm, the high observed intracluster correlation coefficients, and the decline observed in the control arm. We now emphasize that the findings should be interpreted as evidence of association within this specific implementation context rather than definitive proof of large causal effects generalizable across settings.

2. The explanation offered for the decline in the control arm introduces a substantial internal validity concern. The authors attribute this decline to baseline campaigns and community mobilization activities that were not sustained at endline. If true, this represents a time-varying contextual confounder affecting the baseline–endline comparison. In that case, the intervention effect may partly reflect the withdrawal of external support rather than solely the addition of messaging. This possibility should be acknowledged explicitly as a limitation rather than used to reinforce the intervention’s apparent superiority. Moreover, if community bylaws and enforcement against home delivery were active during the study period, the potential for contamination or differential exposure across clusters should be discussed.

Response :We agree that attributing the control arm decline to withdrawal of baseline community mobilization introduces potential internal validity concerns. In the revised discussion, we have reframed this explanation as a limitation rather than a reinforcing argument for intervention superiority. We explicitly acknowledge that time-varying contextual factors, including changes in district-level campaigns and community mobilization activities, may have influenced baseline–endline comparisons. We further discussed the possibility that the observed intervention effect may partly reflect the removal of concurrent external support rather than solely the additive effect of SMS messaging. Additionally, we now address the potential for contamination or differential exposure if community bylaws or enforcement practices were active during the study period, noting that such contextual dynamics may have affected clusters unevenly.

3. The interpretation of maternal morbidity reduction is particularly overstated. The manuscript attributes the reduction to increased ANC attendance and improved adherence, yet morbidity was self-reported and not clinically adjudicated. The magnitude of decline reported is large and biologically ambitious for an SMS-based behavioral intervention without a direct clinical component. Alternative explanations such as reporting bias, improved symptom interpretation, differential recall, or misclassification should be discussed. Without objective clinical verification, the claim that message framing “reduced maternal morbidity” is too strong and risks undermining credibility.

Response: We agree that the interpretation of maternal morbidity reduction required substantial moderation. In the revised manuscript, we clarify that maternal morbidity was self-reported and not clinically adjudicated, and therefore subject to recall bias, reporting bias, and potential misclassification. We have removed language implying direct clinical impact and replaced it with wording that specifies “reductions in reported maternal morbidity.” We also expand the discussion to consider alternative explanations, including improved symptom recognition, differential interpretation of symptoms, increased care-seeking leading to different labeling of complications, and social desirability bias. We acknowledge that the magnitude of change is ambitious for a behavioral SMS intervention without a direct clinical component and caution against strong causal interpretation in the absence of objective verification.

4. The comparative framing conclusions require more statistical restraint. The manuscript states that loss-framed messages were particularly effective for ANC and morbidity while gain-framed messages were more effective for SBA and PNC. However, the results section does not clearly present direct statistical comparisons between gain and loss arms. If head-to-head contrasts were not formally tested, these statements imply differences that may not be statistically supported. The discussion should clarify whether such comparisons were conducted and, if not, rephrase these conclusions to reflect descriptive trends rather than definitive superiority.

Response: We agree that the comparative framing conclusions required greater statistical restraint. In the revised discussion, we clarify that the primary analyses compared each intervention arm to the control arm and did not include formal head-to-head superiority testing between gain- and loss-framed groups unless explicitly specified. We therefore rephrase comparative statements to reflect observed descriptive patterns rather than definitive superiority. Instead of stating that one framing strategy was “more effective,” we now indicate that differential patterns were observed across outcomes and that these patterns warrant further investigation in adequately powered trials designed specifically for direct comparison

**Implication**

The implications section is forward-looking but somewhat overgeneralized. While the findings suggest promise for tailored mobile messaging, recommending integration into policy strategies should be tempered by acknowledgment of contextual specificity. The districts were purposively selected, mobile coverage was adequate, and concurrent community activities were in operation. External validity to more resource-constrained or less mobilized settings remains uncertain. Policy recommendations should be framed as conditional and suggest replication with larger numbers of clusters and intention-to-treat analyses as primary estimands.

Response: We agree that the implications section was overly generalized. In the revised manuscript, we temper policy recommendations and explicitly state that the districts were purposively selected, mobile network coverage was adequate, and concurrent community activities were ongoing. We acknowledge that external validity to more resource-constrained or less mobilized settings remains uncertain. Policy recommendations are now framed conditionally, suggesting that framed mobile messaging shows promise in similar contexts but requires replication in larger trials with more clusters and intention-to-treat analyses as primary estimands before broader scale-up can be justified.

**Strengths**

The strengths section appropriately highlights verification of outcomes and allocation concealment, but it does not fully address key methodological challenges. While adjustment for baseline covariates is mentioned, this does not entirely resolve baseline imbalance concerns in small-cluster trials. The reference to FDA and EMA guidance strengthens the rationale for covariate adjustment, yet the manuscript should clarify exactly which covariates were included and whether adjustment meaningfully altered effect sizes. Additionally, the high observed ICCs relative to the assumptions used in sample size planning are not discussed in the strengths or limitations, despite their implications for precision and generalizability.

Response: We have expanded the strengths section to provide a more balanced perspective. While we retain discussion of allocation concealment and outcome verification procedures, we now clarify exactly which baseline covariates were included in adjusted models and report whether covariate adjustment materially altered effect sizes. We also acknowledge that statistical adjustment cannot fully eliminate baseline imbalance concerns in small-cluster trials. Furthermore, we now explicitly address the discrepancy between assumed and observed ICC values and discuss its implications for effective sample size and estimate precision. This provides a more transparent appraisal of methodological robustness.

**Limitations**

The limitations section is currently underdeveloped relative to the complexity of the findings. Beyond recall bias and tool changes, the authors should explicitly acknowledge: the small number of clusters per arm; high intracluster correlation; baseline imbalances in psychosocial predictors; potential contamination from concurrent community activities; reliance on self-reported morbidity; inability to blind participants; and the risk of social desirability bias in reporting service utilization and barriers. Without a more transparent and comprehensive limitations discussion, the manuscript appears overly confident relative to its design constraints.

Response: We have substantially expanded the limitations section. In addition to recall bias and data collection tool differences, we now explicitly acknowledge the small number of clusters per arm, the high observed intracluster correlation, baseline imbalances in psychosocial predictors, possible contamination from concurrent community activities, reliance on self-reported maternal morbidity, inability to blind participants, and the potential for social desirability bias in reporting service utilization and perceived barriers. By articulating these constraints clearly, we aim to align the manuscript’s tone with the realities of the design and reduce the risk of overinterpretation.

In overall, the discussion would benefit from a stronger theoretical integration. While you cite literature on gain versus loss framing, they do not deeply engage with why framing effects were modestly differentiated in this context or why self-efficacy did not improve significantly despite changes in knowledge and attitudes. A more nuanced theoretical reflection considering ceiling effects, cultural context, stage of behavior change, and preventive versus detection behaviors would elevate the manuscript and align it more closely with the standards expected. The discussion section contains important insights and promising findings, but it requires substantial moderation of causal claims, clearer acknowledgment of design limitations, and more rigorous linkage between theory, methods, and interpretation to meet publication standards.

**Theoretical Integration**

**Response:** We agree that the discussion required deeper theoretical engagement. In the revised version, we more carefully integrate behavioral theory to explain why framing effects may have differed across maternal health behaviors. We discuss the distinction between preventive and detection behaviors, possible ceiling effects for self-efficacy, and the role of cultural norms and behavioral stage in moderating framing responsiveness. We also explore why knowledge and attitudes improved while self-efficacy did not change significantly, considering whether structural barriers or entrenched norms may limit translation of cognitive shifts into perceived control. This expanded theoretical reflection strengthens the scholarly contribution beyond programmatic findings.

**Conclusion**

1. The conclusion is currently too definitive relative to the methodological constraints of the study. Phrases such as “the study conclusively demonstrates” and “significantly enhance maternal health outcomes” overstate the strength of inference that can reasonably be drawn from a cluster trial with a small number of clusters, baseline imbalances, high observed ICCs, and reliance on self-reported morbidity. Conclusions should reflect statistical association within the study context rather than absolute proof. The wording should be moderated to state that the findings “suggest” or “indicate” effectiveness in this setting, rather than conclusively demonstrating it.

**Response:** We have revised the conclusion to remove definitive language such as “conclusively demonstrates” and “significantly enhance maternal health outcomes.” The conclusion now states that the findings “suggest” or “indicate” potential effectiveness within this specific cluster-randomized context. We explicitly reference the design constraints—including small cluster number, baseline imbalance, high ICCs, and reliance on self-reported morbidity—to frame the conclusions appropriately.

2. The comparative claims regarding gain versus loss framing also exceed the evidence presented. The conclusion states that loss-framed messages were “more effective” for certain outcomes and gain-framed messages for others. However, unless formal head-to-head statistical comparisons between the two intervention arms were conducted and reported, such superiority claims are not fully justified. If the analyses only compared each intervention arm to control, then differences between gain and loss should be described as descriptive patterns rather than statistically established differences.

**Response:** We have moderated comparative framing claims in the conclusion. Unless formal statistical head-to-head comparisons were conducted and reported, we avoid stating that one framing approach was more effective than the other. Differences between gain- and loss-framed arms are now described as observed patterns that require confirmation in trials specifically powered for direct comparison.

3. The statement that maternal morbidity was “reduced” by the intervention should be carefully reconsidered. Because morbidity was self-reported and not clinically adjudicated, and given the magnitude of change observed, the conclusion should refer to reductions in reported maternal morbidity rather than implying direct clinical impact. Without objective outcome validation, stronger clinical language may invite skepticism from reviewers and readers.

**Response:** We have revised the statement regarding maternal morbidity to refer explicitly to reductions in “self-reported maternal morbidity” rather than implying direct clinical reduction. This ensures consistency with the measurement approach and avoids overstating biological impact.

4. The conclusion would benefit from clearer acknowledgment of contextual boundaries. The study was conducted in purposively selected districts with concurrent community mobilization activities and adequate mobile network coverage. Therefore, generalization to other regions especially those with weaker infrastructure or different sociocultural dynamics should be framed cautiously. Rather than broad claims about “similar contexts,” it would be more appropriate to suggest that mobile phone-based framed messaging shows promise and warrants further evaluation in diverse settings with larger cluster samples.

**Response:** We now explicitly acknowledge contextual boundaries in the conclusion. The study setting, purposive district selection, mobile coverage conditions, and concurrent mobilization activities are referenced as factors limiting generalizability. We suggest that further evaluation in diverse and potentially less resourced settings is necessary before broader application.

5. Finally, the conclusion should more clearly integrate the theoretical contribution of the study. Instead of primarily emphasizing outcome improvements, it could briefly highlight that framing effects appear to operate differently across specific maternal health behaviors and psychosocial domains, reinforcing the importance of aligning message framing with behavioral stage and context. This would strengthen the scholarly contribution beyond programmatic implications.

**Response:** We have strengthened the theoretical contribution in the conclusion by briefly highlighting that framing effects may operate differently across maternal health behaviors and psychosocial domains. We emphasize the importance of aligning message framing with behavioral stage and context, thereby reinforcing the conceptual implications of the study rather than focusing solely on outcome magnitudes.

Finally, the conclusion should be revised to temper causal language, avoid overgeneralization, clarify the limits of comparative framing claims, and acknowledge reliance on self-reported outcomes. A more balanced and cautiously framed conclusion will enhance the credibility and impact of the manuscript.

Response : Overall, the revised discussion and conclusion now temper causal language, acknowledge methodological constraints transparently, avoid unsupported superiority claims, and integrate theoretical insight more rigorously. These revisions substantially strengthen the manuscript’s credibility and alignment with high-impact publication standards.

**Verdict: Major Revision Needed**
