# Supplementary material for: Evaluating gain and loss-framed mobile messaging to improve maternal health service uptake: A cluster randomized controlled trial in Jimma, Ethiopia
Source: PLoS One. 2026 Oct 26;21(10):e0360474. doi: 10.1371/journal.pone.0360474 (PMC13704815; doi:10.1371/journal.pone.0360474)
Supplement: Supplementary file 12 [file pone.0360474.s013.docx]

Evaluating Gain and Loss-Framed Mobile Messaging to Improve Maternal Health Service Uptake: A Cluster Randomized Controlled Trial in Jimma, Ethiopia." (PONE-D-25-10242R3).

Dear Editor, Thank you so much for allowing me to review this important manuscript. Since the number of trial papers in our country (Ethiopia) is limited, I appreciate the authors for considering this interventional study to solve the maternal health service utilization problem.

**Comments**

Minor comment on the abstract

1. In the background of the abstract section, what the broad topic is and what your paper aims to explain are included; however, what is missing, unknown, or problematic is not included.

Suggestion: It will be informative if you include one sentence of the research gap in the background of the abstract.

**Response :** We appreciate this observation. We have revised the background of the abstract to explicitly state the research gap, noting that evidence on whether gain‑ versus loss‑framed mobile messages differentially influence maternal health service uptake in rural Ethiopia remains limited.

2. In the conclusion part of the abstract, the use of the word "...was associated..." Indicate weakness in the RCT's finding.

Suggestion: Use active verbs because you applied a well-designed randomized control trial that rules out external factors through randomization; your conclusion should boldly reflect that causal link using active verbs. Instead of saying “were associated with increased maternal health service utilization,” use the phrase "lead to increased maternal health service utilization."

**Response :** Thank you for this important point. We have revised the conclusion to use active verbs, stating that gain‑ and loss‑framed mobile messages *led to* increased maternal health service utilization, reflecting the causal inference supported by our randomized trial design.

**Introduction**

1. This interventional study was on mobile-based messages and how message framing affects maternal services usage; however, nothing was said about mobile phone usage in Ethiopia. How many of the rural women would have access to mobile phones? This data is important for further policy implementation. and for your

Suggestion: It would be better if you include the magnitude of mobile usage and any training in reading the message from literature.

**Response :** We agree with the reviewer. We have added recent national survey data on mobile phone penetration among rural women in Ethiopia, and noted literacy considerations relevant to message comprehension, to strengthen the policy relevance of our study.

2. The introduction is well-informed about the background data of maternal death, the cause of death, which is supported by evidence. The prospect theory also connected with the importance of the intervention and the research gap also discussed. However, lack of consistency in using references and a plausible argument for how the message framing influenced the maternal mortality were not yet included. Regarding inconsistency, what has been cited in the content of the manuscript and document (reference number 20) was repeated in the discussion. In addition, redundancy on page 4, paragraph 4, and paragraph 5, line numbers 6 to 0, was observed.

Suggestion: better if you include a plausible argument for inclusion of morbidity as outcome measurement in relation to your intervention. Check your references and better to clear the inconsistency. Delete the duplicate.

**Response :** Thank you for highlighting this. We have corrected the reference inconsistencies and removed duplicate citations. In addition, we have added a rationale for including maternal morbidity as an outcome, linking it to the potential of communication interventions to influence not only service uptake but also women’s self‑reported health experiences.

Method

On the methodological aspect, registration of the trial and publication of the trial prospectively are strengths. However, if there is any deviation from the publication, an amendment is a possible solution. For example, your primary outcome mentioned in the published protocol was maternal health service usage, which was defined as "maternal health service usage" when mothers receive at least 8 ANC follow-up visits, give birth in a health facility, and visit a health facility for PNC follow-up contact from health professionals within 7 days of delivery. However, you have changed in health service access by measuring receiving one antenatal care. This may be possible, but you have to amend this; instead, you have stated no change in outcome measurement after the protocol was

Published. Page 14, line number 367-369

Possible suggestion: include amendment of the protocol for any change after the publication of the protocol.

**Response :** We thank the reviewer for this important observation. While the trial was conducted as planned with no changes to eligibility criteria, intervention protocols, or data collection procedures, we acknowledge that an amendment was made to the primary outcome definition after protocol registration. Specifically, maternal health service usage was initially defined in the published protocol as receipt of at least eight ANC visits, institutional delivery, and PNC within 7 days. To reflect feasibility in rural Ethiopia, ANC utilization was redefined as at least one ANC visit (ANC1+). This amendment was formally approved by the ethics committee and documented in the trial records. We have now clarified this amendment in the Methods section to ensure transparency and consistency with the published protocol.

1. Describing the limitation in the method part better is avoided. Line number 176-178 on page 7 and page number 11 line number 293-297

**Response :** Thank you. We have removed limitations from the Methods section and relocated them to the Discussion, in line with reporting standards.

2. Redundancy on page 11, line number 303-305 and 306- 308

**Response :** Thank you. We have deleted the redundant sentences on page 11 (lines 303–308) to improve clarity.

Sample size determination

What was the suggested effect size before trial and have you considered this during interpretation of your finding?

**Response :** We appreciate this comment. We have now explicitly reported the assumed effect size (an absolute increase of 15 percentage points in ANC attendance, from 23.27% in the control arm to 38.27% in the intervention arms) that was used in the sample size calculation. In the *Discussion*, we have clarified how this prespecified effect size was considered when interpreting our findings, noting that the observed improvements in ANC, SBA, and PNC utilization met or exceeded the expected difference, thereby reinforcing the robustness of our conclusions.

Result

Comment 1. Comment 1. Minor editorial page 18 , line number 472

**Response :** Corrected as suggested.

Comment 2. Table number 2 line number 495 page 19

**Response :** Corrected as suggested.

Comment 3 : The objective of this table is to show if the study arm is balanced at the baseline; however, there should be either a p-value or more recommended mean/proportion differences. This makes the comparison easier for the reader.

**Response :** Thank you. We have revised Table 3 to include standardized mean differences for baseline characteristics, allowing clearer comparison of balance across study arms.

Comment 4. Too much information at a time makes confusion even, if it has connection better to report only the objective of the trial (primary and secondary outcomes) . I am not sure on the importance of Ancillary analysis.

Suggestion: better to avoid the result below this, otherwise include something about it in the introduction and method part. It makes the paper heavier and difficult to grasp the important finding.

**Response :** The ancillary analyses have been removed from the *Results* section. The section now focuses exclusively on primary and secondary outcomes, with only brief references in the *Methods* where necessary to justify analytic decisions.
