# Supplementary material for: Evaluating gain and loss-framed mobile messaging to improve maternal health service uptake: A cluster randomized controlled trial in Jimma, Ethiopia
Source: PLoS One. 2026 Oct 26;21(10):e0360474. doi: 10.1371/journal.pone.0360474 (PMC13704815; doi:10.1371/journal.pone.0360474)
Supplement: Peer review history

# Peer review: Decision Letter 0

**DOI:** 10.1371/journal.pone.0360474.r001

**Authors:** Poshan Thapa

28 Apr 2025

Dear Dr. Gebeyehu Bulcha,

Thank you for submitting your manuscript to PLOS ONE. After careful consideration, we feel that it has merit but does not fully meet PLOS ONE’s publication criteria as it currently stands. Therefore, we invite you to submit a revised version of the manuscript that addresses **every points**  raised during the review process.

==============================

**ACADEMIC EDITOR:**

1. In the Introduction, the authors state: *"The impact of message-framing strategies on maternal health service uptake through mobile phone messaging has not been extensively studied, particularly in rural areas where barriers to accessing care are more pronounced."*
  Could the authors please include references to studies, to whatever extent they exist, that have examined this issue? This would help to better frame the context and justify the need for the present study.
2. The authors must clearly state why Hooper and Bourke’s longitudinal methodology was applied to this study design, given the limited number of measurement points (baseline and endline).
3. Are the primary outcomes patient-reported outcomes, or was an effort made to cross-verify the data from patients’ medical records, such as ANC cards? Please clarify how the data were validated.
4. The authors should explicitly report the percentage of missing data (2.7%) and its nature (e.g., random or non-random). Additionally, please clarify the detailed procedure for multiple imputations, including the number of imputations performed, the predictors included in the model, and the convergence criteria used.
5. In the ancillary analysis, the authors report: *"Additionally, the issues of long wait times and inconvenient hours were almost entirely resolved, decreasing from 98.6% to 1.4% and 95.8% to 4.2%, respectively. These changes indicate a substantial improvement in accessibility and perceived quality of health services over the period."*
  However, as the intervention was not directly designed to affect health service quality or waiting times, what factors might have led participants to report such improvements? The authors are encouraged to reflect on this finding and offer possible explanations.
6. Please ensure that all references are formatted according to the PLOS ONE referencing style.

==============================

Please submit your revised manuscript by Jun 12 2025 11:59PM. If you will need more time than this to complete your revisions, please reply to this message or contact the journal office at plosone@plos.org. When you're ready to submit your revision, log on to https://www.editorialmanager.com/pone/ and select the 'Submissions Needing Revision' folder to locate your manuscript file.

- A rebuttal letter that responds to each point raised by the academic editor and reviewer(s). You should upload this letter as a separate file labeled 'Response to Reviewers'.
- A marked-up copy of your manuscript that highlights changes made to the original version. You should upload this as a separate file labeled 'Revised Manuscript with Track Changes'.
- An unmarked version of your revised paper without tracked changes. You should upload this as a separate file labeled 'Manuscript'.

If you would like to make changes to your financial disclosure, please include your updated statement in your cover letter. Guidelines for resubmitting your figure files are available below the reviewer comments at the end of this letter.

If applicable, we recommend that you deposit your laboratory protocols in protocols.io to enhance the reproducibility of your results. Protocols.io assigns your protocol its own identifier (DOI) so that it can be cited independently in the future. For instructions see: https://journals.plos.org/plosone/s/submission-guidelines#loc-laboratory-protocols. Additionally, PLOS ONE offers an option for publishing peer-reviewed Lab Protocol articles, which describe protocols hosted on protocols.io. Read more information on sharing protocols at https://plos.org/protocols?utm_medium=editorial-email&utm_source=authorletters&utm_campaign=protocols.

We look forward to receiving your revised manuscript.

Kind regards,

Poshan Thapa, PhD

Academic Editor

PLOS ONE

Journal Requirements:

When submitting your revision, we need you to address these additional requirements.

1. Please ensure that your manuscript meets PLOS ONE's style requirements, including those for file naming. The PLOS ONE style templates can be found at

https://journals.plos.org/plosone/s/file?id=wjVg/PLOSOne_formatting_sample_main_body.pdf and

https://journals.plos.org/plosone/s/file?id=ba62/PLOSOne_formatting_sample_title_authors_affiliations.pdf.

2. Thank you for stating in your Funding Statement:

“This research was supported by grants from Jimma University for postgraduate students and additional funding from the Institute of Health, Department of Health, Behavior, and Society. The findings and conclusions are those of the authors and do not necessarily reflect the views of these organizations.”

Please provide an amended statement that declares *all* the funding or sources of support (whether external or internal to your organization) received during this study, as detailed online in our guide for authors at http://journals.plos.org/plosone/s/submit-now.  Please also include the statement “There was no additional external funding received for this study.” in your updated Funding Statement.

Please include your amended Funding Statement within your cover letter. We will change the online submission form on your behalf.

3. In the online submission form you indicate that your data is not available for proprietary reasons and have provided a contact point for accessing this data. Please note that your current contact point is a co-author on this manuscript. According to our Data Policy, the contact point must not be an author on the manuscript and must be an institutional contact, ideally not an individual. Please revise your data statement to a non-author institutional point of contact, such as a data access or ethics committee, and send this to us via return email. Please also include contact information for the third party organization, and please include the full citation of where the data can be found.

4. Thank you for stating the following in the Acknowledgments Section of your manuscript:

“This research was supported by grants from Jimma University for postgraduate students and additional funding from the Institute of Health, Department of Health, Behavior, and Society. The findings and conclusions are those of the authors and do not necessarily reflect the views of these organizations.:

We note that you have provided additional information within the Acknowledgements Section that is not currently declared in your Funding Statement. Please note that funding information should not appear in the Acknowledgments section or other areas of your manuscript. We will only publish funding information present in the Funding Statement section of the online submission form.

Please remove any funding-related text from the manuscript and let us know how you would like to update your Funding Statement. Currently, your Funding Statement reads as follows:

“This research was supported by grants from Jimma University for postgraduate students and additional funding from the Institute of Health, Department of Health, Behavior, and Society. The findings and conclusions are those of the authors and do not necessarily reflect the views of these organizations.”

Please include your amended statements within your cover letter; we will change the online submission form on your behalf.

5. Your ethics statement should only appear in the Methods section of your manuscript. If your ethics statement is written in any section besides the Methods, please delete it from any other section.

6. Please include captions for your Supporting Information files at the end of your manuscript, and update any in-text citations to match accordingly. Please see our Supporting Information guidelines for more information: http://journals.plos.org/plosone/s/supporting-information.

[Note: HTML markup is below. Please do not edit.]

Reviewers' comments:

Reviewer's Responses to Questions

**Comments to the Author**

1. Is the manuscript technically sound, and do the data support the conclusions?

Reviewer #1: Yes

Reviewer #2: Partly

**********

2. Has the statistical analysis been performed appropriately and rigorously?

Reviewer #1: No

Reviewer #2: Yes

**********

3. Have the authors made all data underlying the findings in their manuscript fully available?

The PLOS Data policy

Reviewer #1: No

Reviewer #2: Yes

**********

4. Is the manuscript presented in an intelligible fashion and written in standard English?

Reviewer #1: Yes

Reviewer #2: No

**********

Reviewer #1: The authors have conducted a trial comparing two types of mobile phone messaging (gain and loss) with control in a cluster randomised trial.

The terms gain-framed and loss-framed are not defined in the abstract and should be.

Likewise in the abstract, results need to be reported to a common degree of precision, it is standard to use 2dp throughout.

The sample size calculation here is clearly for two-comparisons versus control given the 2.5% two-sided significance level. Given this I'm unclear what the Bonferroni correction is in this context - the Z for the power should just be Z\beta and not Z\beta/2 This needs to be better explained; the numbers are correct for 80% power and 97.5% two-sided CI. The z here are 2.2414 and 0.8416. I think there is merely a little confusion in the write-up.

Given the reliance on p<.025 the 95% CI do not match the test at the design stage and 97.5% CI need also to be given.

Given the small number of clusters here, it is important to identify if there is any confounding (e.g. husband's education) - there is a typo in this table which should read "College" and not "Collage"

Parity and number of family members able to read and write are not generally accepted to be Normally distributed. As such these should be tabulated.

I am unclear what the starting figures for postnatal care etc actually mean here as they are presumably affected by parity. How can one be using postnatal care already and then later access antenatal care? How does one allow for primiparous women here if this is access for previous pregnancies? Where is parity in Table 1?

Table 3 needs to distinguish between within patient data and between patient data. I cannot reproduce the OR & CI from the data given here.

Please give the actual p-values throughout - p<.05 is not the p-value specified.

In Table 4 the aMD for the control arm is not 1 but zero.

Figure 2 represents paired data and it si not clear who has moved from one group to another. It is unclear whether there are changes in the control arm also which would indicate a desire to please.

Reviewer #2: Section Reviewer’s Comment (Revised)

Title The title is informative and conveys the key components of the study. However, it should be more concise and polished for better readability. For instance: "Evaluating Gain and Loss-Framed Mobile Messaging to Improve Maternal Health Service Uptake: A Cluster Randomized Trial in Rural Jimma, Ethiopia."

Keywords No revisions required.

Abstract The abstract is well-structured and follows a logical sequence. Improvements are needed to trim excessive detail, ensure consistent terminology (e.g., "self-efficacy"), and enhance the conclusion to emphasize the significance of the findings more effectively.

Trial Registration Trial registration details are inaccessible online. Authors should confirm registration compliance, including providing the registration number, registry name, and URL if available. Any missing details must be addressed or explicitly acknowledged to ensure transparency.

Background The section contains the required elements but lacks flow and coherence. A structured writing style should be followed, such as moving from global to local contexts or vice versa. Additionally, ideas should align closely with the study objectives for better synchronization.

Method Setting: The criteria for selecting Manna, Shebe Sombo, and Dedo districts from the Jimma Zone must be clarified

Population: The inclusion of primiparous women in the sample is unclear, as baseline data were collected from those with at least one prior childbirth. Clarification is needed

Sample size: The sample size appears insufficient considering 21 clusters. Consultation with a statistician is recommended to justify whether the current sample size is adequate. If 21 clusters are considered for sample size calculation, the required sample size would be much larger.

Intervention development: Correct flow and remove repetitions, e.g., the repeated definition of loss frame and word frame (lines 182–184 and 216–218). Clarify who delivered the SMS messages—telecommunication personnel, researchers, or others—and whether mechanisms were in place to confirm participants received and read the messages.

Blinding: Line 297 states, “Blinding the participants was not feasible due to the nature of the intervention.” Clarify why SMS interventions made blinding impossible.

Data Collection Procedure Line 343-344 mentions, “Baseline data were collected using a paper-based method, while endline data were collected using the Kobo toolkit.” Clarify whether this methodological difference could introduce bias.

Analyses The manuscript states that “Generalized Linear Mixed Model (GLMM)” was used, but no GLMM results appear in the results section. This requires justification. Additionally, explain why GLMM was chosen instead of Generalized Estimating Equations (GEE), which are often preferred for non-normally distributed data.

Result The first four sentences of this section belong to the methods section and create redundancy, so they should be removed.

Include data for both baseline and endline participant characteristics, rather than focusing solely on baseline.

Address the significant differences in ANC utilization rates between the gain and loss frame groups at baseline (85.7% vs. 90.8%). Clarify how these differences were managed to ensure the conclusions are attributable to messaging effects rather than baseline disparities.

The observed changes in institutional births are exceptionally significant, with a 37% increase in the gain-framed group and a 26% increase in the loss-framed group. These findings warrant a thorough review to ensure the validity and accuracy of the data. The reviewer strongly recommends revisiting the original dataset and conducting reanalysis if necessary to confirm the reliability of these results. If verified, such outcomes would represent an unprecedented level of effectiveness with profound global implications. However, the magnitude of these changes, described as world-leading, raises concerns about data integrity and representation. The authors should scrutinize the findings to ensure their accuracy and reliability.

Additionally, while ancillary analyses are noteworthy, it is crucial to first evaluate whether secondary variables—such as knowledge, attitude, self-efficacy, and maternal morbidity—predicted antenatal care (ANC), delivery, and postnatal care utilization.

Discussion This section is acceptable but must be revised in light of changes to previous sections to ensure consistency.

Conclusion The conclusion suggests that both gain and loss frames are effective, but the authors should clearly indicate which approach is more effective. Numerical figures, such as “gain-framed arm improved ANC by 11.6%, institutional births by 26.3%, PNC by 12.8%, etc.,” should not appear in the conclusion. Additionally, ensure all reported figures match the results section.

Reference Numerous references, such as “WHO Maternal Health. Who 2018, 1–47,” are not cited correctly according to the Vancouver style. For example, this reference lacks the title. Use reference management software such as Mendeley to correct these errors systematically.

Language Ensure coherent descriptions to avoid disconnections and unnecessary repetitions. Multiple grammatical, typographical, and spelling errors must be corrected through thorough editing.

**********

what does this mean?). If published, this will include your full peer review and any attached files.

If you choose “no”, your identity will remain anonymous but your review may still be made public.

**Do you want your identity to be public for this peer review?**  For information about this choice, including consent withdrawal, please see our Privacy Policy

Reviewer #1: No

Reviewer #2: No

**********

[NOTE: If reviewer comments were submitted as an attachment file, they will be attached to this email and accessible via the submission site. Please log into your account, locate the manuscript record, and check for the action link "View Attachments". If this link does not appear, there are no attachment files.]

While revising your submission, please upload your figure files to the Preflight Analysis and Conversion Engine (PACE) digital diagnostic tool, https://pacev2.apexcovantage.com/. PACE helps ensure that figures meet PLOS requirements. To use PACE, you must first register as a user. Registration is free. Then, login and navigate to the UPLOAD tab, where you will find detailed instructions on how to use the tool. If you encounter any issues or have any questions when using PACE, please email PLOS at figures@plos.org. Please note that Supporting Information files do not need this step.

Attachment Submitted filename: Reviewer Comment Final-1.docx

# Author comment: Author response to Decision Letter 1

**DOI:** 10.1371/journal.pone.0360474.r002

8 Jul 2025

Academic Editor Comments

1. In the Introduction, the authors state: "The impact of message-framing strategies on maternal health service uptake through mobile phone messaging has not been extensively studied, particularly in rural areas where barriers to accessing care are more pronounced."

Could the authors please include references to studies, to whatever extent they exist, that have examined this issue? This would help to better frame the context and justify the need for the present study.

Response: Thank you for your constructive comments. We have updated the Introduction to reference relevant studies that have investigated message-framing strategies in maternity health contexts.

2. The authors must clearly state why Hooper and Bourke’s longitudinal methodology was applied to this study design, given the limited number of measurement points (baseline and endline).

Response: We appreciate the editor's insightful question regarding our application of Hooper and Bourke's longitudinal analysis method in a study with only two measurement points (baseline and endline). Although the intervention was implemented simultaneously across all clusters, this method was appropriate because it accounts for both within-cluster and over-time correlation structures inherent in repeated measures cluster randomized trials, even when only two time points are available. In our study, we employed a repeated cross-sectional design, collecting outcome measures at baseline and endline from the same individuals within each cluster. This design necessitated adjustments for potential confounding due to time and intracluster correlation across time points. Hooper and Bourke's approach enabled us to address intracluster correlations both within and between periods during sample size estimation and statistical analysis. This enhanced the accuracy of our effect estimates and strengthened the robustness of our trial design. Given the cluster-randomized nature of our trial and the repeated cross-sectional measurement of outcomes, the application of Hooper and Bourke's method was methodologically sound and is detailed in the revised manuscript.

3. Are the primary outcomes patient-reported outcomes, or was an effort made to cross-verify the data from patients’ medical records, such as ANC cards? Please clarify how the data were validated.

Response: The primary outcomes were self-reported outcomes collected via interviewer-administered structured questionnaires at baseline and endline. However, to improve the validity of the majority of self-reported information—especially on maternal health service utilization (e.g., number and timing of ANC visits, place of delivery, and PNC attendance)—data collectors tried to cross-check information with participants' ANC cards and other available medical records where feasible during the interview. This assisted in eliminating recall bias and improved accuracy. Moreover, the data collection team consisted of trained health professionals familiar with maternal health data documentation, thus ensuring more credible information. All the questionnaires went through intense translation and back-translation, and a pretest was carried out to ensure clarity and uniformity of the instruments.

4. The authors should explicitly report the percentage of missing data (1.4%) and its nature (e.g., random or non-random). Additionally, please clarify the detailed procedure for multiple imputations, including the number of imputations performed, the predictors included in the model, and the convergence criteria used.

Response: Thank you for your constructive comments. We have now explicitly reported that 2.7% of the data were missing and, based on our analysis, the missingness was determined to be missing at random (MAR). We performed multiple imputations using the chained equations method with 20 imputations. Predictors included in the imputation model were age, parity, education level, baseline service utilization, and intervention arm.

5. In the ancillary analysis, the authors report: "Additionally, the issues of long wait times and inconvenient hours were almost entirely resolved, decreasing from 98.6% to 1.4% and 95.8% to 4.2%, respectively. These changes indicate a substantial improvement in accessibility and perceived quality of health services over the period." However, as the intervention was not directly designed to affect health service quality or waiting times, what factors might have led participants to report such improvements? The authors are encouraged to reflect on this finding and offer possible explanations.

Response: We acknowledge that the intervention did not directly target health service quality or waiting time. However, the improvements seen are probably a spillover effect of the messaging intervention: by increasing participants' awareness and readiness, they likely scheduled visits during less busy hours and communicated more effectively with providers, which reduced perceived wait times and inconvenience. Exposure to the intervention can also have internally encouraged health workers to provide services more effectively through friendlier interactions or increased transparency over delays, although provider behaviors were not formally measured in our study.

6. Please ensure that all references are formatted according to the PLOS ONE referencing style.

Response: We have thoroughly reviewed and reformatted all references to adhere to the PLOS ONE referencing style, ensuring consistency and compliance throughout the manuscript.

Additional requirements.

1. Please ensure that your manuscript meets PLOS ONE's style requirements, including those for file naming. The PLOS ONE style templates can be found at

https://journals.plos.org/plosone/s/file?id=wjVg/PLOSOne_formatting_sample_main_body.pdf and

https://journals.plos.org/plosone/s/file?id=ba62/PLOSOne_formatting_sample_title_authors_affiliations.pdf.

Response: Thank you for your constructive comments. We have thoroughly reviewed the PLOS ONE style guidelines—including the provided templates for manuscript formatting and title/affiliations—and have ensured our files comply exactly with their requirements.

2. Thank you for stating in your Funding Statement:

“This research was supported by grants from Jimma University for postgraduate students and additional funding from the Institute of Health, Department of Health, Behavior, and Society. The findings and conclusions are those of the authors and do not necessarily reflect the views of these organizations.”

Please provide an amended statement that declares *all* the funding or sources of support (whether external or internal to your organization) received during this study, as detailed online in our guide for authors at http://journals.plos.org/plosone/s/submit-now. Please also include the statement “There was no additional external funding received for this study.” in your updated Funding Statement.

Please include your amended Funding Statement within your cover letter. We will change the online submission form on your behalf.

Response: Thank you for your constructive comments. We have expanded our Funding Statement to accurately reflect all sources of support received during this study, as per PLOS ONE’s guidelines. The updated statement is included below and has been inserted into our revised cover letter as requested.

3. In the online submission form you indicate that your data is not available for proprietary reasons and have provided a contact point for accessing this data. Please note that your current contact point is a co-author on this manuscript. According to our Data Policy, the contact point must not be an author on the manuscript and must be an institutional contact, ideally not an individual. Please revise your data statement to a non-author institutional point of contact, such as a data access or ethics committee, and send this to us via return email. Please also include contact information for the third party organization, and please include the full citation of where the data can be found.

Response: Thank you for your constructive comment, we have revised the data availability statement accordingly.

Revised Data Availability Statement: The de identified data used in this study are not publicly available online because study participants did not consent to public data release; they only agreed to publication of aggregated results. However, the data can be accessed upon reasonable request through a non-author institutional contact, the Jimma Zone Health Office, Department of Reproductive, Maternal, and Neonatal Health. Requests for data access should be sent to: jzhrd2015@gmail.com

Data Source (third-party, institutional custody): Jimma Zone Health Office, Department of Reproductive, Maternal, and Neonatal Health, Ethiopia.

Full citation of the data

Jimma Zone Health Office, Department of Reproductive, Maternal, and Neonatal Health. (2025). De identified dataset for the cluster randomized trial on maternal health service uptake in rural Jimma, Ethiopia. Held by Jimma Zone Health Office. Available upon reasonable request via email: jzhrd2015@gmail.com .

4. Thank you for stating the following in the Acknowledgments Section of your manuscript:

“This research was supported by grants from Jimma University for postgraduate students and additional funding from the Institute of Health, Department of Health, Behavior, and Society. The findings and conclusions are those of the authors and do not necessarily reflect the views of these organizations.:

We note that you have provided additional information within the Acknowledgements Section that is not currently declared in your Funding Statement. Please note that funding information should not appear in the Acknowledgments section or other areas of your manuscript. We will only publish funding information present in the Funding Statement section of the online submission form.

Please remove any funding-related text from the manuscript and let us know how you would like to update your Funding Statement. Currently, your Funding Statement reads as follows:

“This research was supported by grants from Jimma University for postgraduate students and additional funding from the Institute of Health, Department of Health, Behavior, and Society. The findings and conclusions are those of the authors and do not necessarily reflect the views of these organizations.”

Please include your amended statements within your cover letter; we will change the online submission form on your behalf.

Response: Thank you for your constructive comments. We have expanded our Funding Statement to accurately reflect all sources of support received during the course of this study, as per PLOS ONE’s guidelines. The updated statement is included below and has been inserted into our revised cover letter as requested.

5. Your ethics statement should only appear in the Methods section of your manuscript. If your ethics statement is written in any section besides the Methods, please delete it from any other section.

Response: Thank you for your constructive comments. We have deleted from the other section and moved to method part accordingly

6. Please include captions for your Supporting Information files at the end of your manuscript, and update any in-text citations to match accordingly. Please see our Supporting Information guidelines for more information: http://journals.plos.org/plosone/s/supporting-information.

Response: Thank you for your constructive comments. We have included accordingly in the main manuscript

Reviewer #1 Comments

1. The authors have conducted a trial comparing two types of mobile phone messaging (gain and loss) with control in a cluster randomised trial. The terms gain-framed and loss-framed are not defined in the abstract and should be.

Response: Thank you for your constructive comments. We have revised the abstract to include definitions of gain-framed and loss-framed messages. Specifically, gain-framed messages emphasize the benefits of engaging in a behavior, while loss-framed messages highlight the consequences of not engaging in the behavior.

2. Likewise in the abstract, results need to be reported to a common degree of precision, it is standard to use 2dp throughout.

Response: Thank you for your comments. For consistency and clarity, we have standardized the reporting of all numerical results to two decimal places throughout the manuscript.

3. The sample size calculation here is clearly for two-comparisons versus control given the 2.5% two-sided significance level. Given this I'm unclear what the Bonferroni correction is in this context - the Z for the power should just be Z\beta and not Z\beta/2 This needs to be better explained; the numbers are correct for 80% power and 97.5% two-sided CI. The z here are 2.2414 and 0.8416. I think there is merely a little confusion in the write-up.

Response: Thank you for your comment. We conducted sample size calculation based on two main comparisons (gain-framed vs. control and loss-framed vs. control), and a Bonferroni correction was used to account for multiple comparisons (more than one statistical test), so each comparison was tested at a level of 0.025. We have clarified the manuscript to make this rationale and the resultant Z-values used in the calculation explicit.

4. Given the reliance on p<.025 the 95% CI do not match the test at the design stage and 97.5% CI need also to be given.

Response: Thank you for your constructive comments. We have updated the confidence intervals to 97.5% to align with the adjusted significance level used in the sample size calculation and hypothesis testing.

5. Given the small number of clusters here, it is important to identify if there is any confounding (e.g. husband's education) - there is a typo in this table which should read "College" and not "Collage"

Response: We have corrected the typographical error from "Collage" to "College" in the table 2. Additionally, we have analyzed to assess potential confounding factors, including husband's education, and have adjusted for these in our statistical models as appropriate.

6. Parity and number of family members able to read and write are not generally accepted to be Normally distributed. As such these should be tabulated.

Response: Thank you for your constructive comment. We have revised the manuscript to present parity and the number of literate family members as categorical variables in tabular form, providing frequencies and percentages to accurately reflect their distributions.

7. I am unclear what the starting figures for postnatal care etc actually mean here as they are presumably affected by parity. How can one be using postnatal care already and then later access antenatal care? How does one allow for primiparous women here if this is access for previous pregnancies? Where is parity in Table 1?

Response: Thank you for your comments. The baseline figures for postnatal care utilization refer to services accessed during previous pregnancies. In our study, we included only multiparous women who had at least one prior childbirth, ensuring that baseline data on postnatal care were applicable. Parity information was already included in Table 2, found on the last row of the table.

8. Table 3 needs to distinguish between within-patient data and between-patient data. I cannot reproduce the OR and CI from the data given here.

Response: Thank you for your observation. We have revised Table 3 to clearly distinguish between within-subject (paired) and between-subject data. Additionally, we have provided detailed information on the statistical methods used to calculate odds ratios and confidence intervals to facilitate reproducibility. The primary measure of association was the exponentiated coefficient, representing the odds ratio when using a binomial distribution with a logit link function.

9. Please give the actual p-values throughout - p<.05 is not the p-value specified.

Response: Thank you for your comments. We have reported the specified p-values throughout the manuscript.

10. In Table 4 the aMD for the control arm is not 1 but zero.

Response: Thank you for your comment , we have rectified this mistake in Table 4 by placing the control arm's adjusted mean difference (aMD) at zero since it serves as our reference group in the estimates.

11. Figure 2 represents paired data, and it si not clear who has moved from one group to another. It is unclear whether there are changes in the control arm also which would indicate a desire to please.

Response: We acknowledge the need for clarity in Figure 2. We have revised the figure to better illustrate changes within each group over time and have included annotations to indicate shifts in barriers to service utilization. Additionally, we have discussed the potential for social desirability bias in the control group and its implications in the manuscript.

Reviewer #2 Comments

1. Title: The title is informative and conveys the key components of the study. However, it should be more concise and polished for better readability. For instance: "Evaluating Gain and Loss-Framed Mobile Messaging to Improve Maternal Health Service Uptake: A Cluster Randomized Trial in Rural Jimma, Ethiopia."

Response: Thank you for the suggestion. We have revised the title for clarity and conciseness to:

“Evaluating Gain and Loss Framed Mobile Messaging to Improve Maternal Health Service Uptake: A Cluster Randomized Trial in Rural Jimma, Ethiopia.”

2. Keywords No revisions required.

Response: We appreciate your confirmation. No changes were made to the keywords.

3. Abstract: The abstract is well-structured and follows a logical sequence. Improvements are needed to trim excessive detail, ensure consistent terminology (e.g., "self-efficacy"), and enhance the conclusion to emphasize the significance of the findings more effectively.

Response: Thank you for your constructive comments. We have edited the abstract by removing excessive details, ensuring consistent use of terminology such as "self-efficacy," and strengthening the conclusion to better highlight the significance and implications of our findings.

4. Trial Registration Trial registration details are inaccessible online. Authors should confirm registration compliance, including providing the registration number, registry name, and URL if available. Any missing details must be addressed or explicitly acknowledged to ensure transparency.

Response: Thank you for your comment. The trial is registered with the Pan African Clinical Trials Registry: PACTR202201753436676. Registry name “Pan African Clinical Trials Registry” and URL https://tinyurl.com/ykhnpc49 have been added to the manuscript. We have confirmed that the trial was prospectively registered on 4 January 2022

5. Background The section contains the required elements but lacks flow and coherence. A structured writing style should be followed, such as moving from global to local contexts or vice versa. Additionally, ideas should align closely with the study objectives for better synchronization.

Response: Thank you for your constructive feedback. We have reorganized the background section of the manuscript to enhance its structure and coherence.

6. Method Setting: The criteria for selecting Manna, Shebe Sombo, and Dedo districts from the Jimma Zone must be clarified

Response: Thank you for your comments. Manna, Shebe Sombo, and Dedo were purposively selected because they: a) have no ongoing similar mHealth interventions,

b) had functional health posts, c) had good mobile network coverage, and d) provided geographic diversity within Jimma Zone. We have edited the manuscript accordingly

7. Population: The inclusion of primiparous women in the sample is unclear, as baseline data were collected from those with at least one prior childbirth. Clarification is needed

Response: Thank you for your insightful comment. According to standard obstetric definitions, a primiparous woman is one who has given birth once to a fetus at or beyond the viable gestational age, typically ≥20 weeks, regardless of whether the baby was born alive or stillborn. This classification remains until she completes her second birth, at which point she becomes multiparous (para 2). In our study, we have included women who are currently pregnant and have previously given birth once, as per the definition of primiparity. This inclusion aligns with the WHO's standard obstetric definitions and ensures consistency in our classification (https://iiab.me/kiwix/content/wikipedia_en_all_maxi_2023-10/A/Gravidity_and_parity?utm_source=chatgpt.com ).

8. Sample size: The sample size appears insufficient considering 21 clusters. Consultation with a statistician is recommended to justify whether the current sample size is adequate. If 21 clusters are considered for sample size calculation, the required sample size would be much larger.

Response: Applying the well-established method of Hooper & Bourke to cluster-randomized trials with endline and baseline data, we properly controlled for our sample size within-period clustering (ICC ≈ 0.003) and for cluster-period level autocorrelation (π ≈ 0.8), generating a combined design effect (DE = dc × dr ≈ 1.116) (https://www.sciencedirect.com/science/article/pii/S2950433324000090?utm_source=chatgpt.com). Beginning with the individually randomized estimate (n₀ = 175 per arm for a difference of 15 points in ANC4+ attendance with 80% power and α = 0.025) and growing by DE and 4% loss to follow-up equals 196 women in each arm—or 28 participants per health post in 21 clusters. This is in line with best practice that demonstrates baseline-endline cohort designs significantly enhance efficiency through the use of within-cluster and within-individual correlation and reduces clusters needed compared to cross-sectional CRTs (https://pmc.ncbi.nlm.nih.gov/articles/PMC7334046/?utm_source=chatgpt.co . Therefore, given our conservative estimate of correlation, number of clusters, and effect size, the sample size is statistically adequate for the detection of the intervention effect of interest.

9. Intervention development: Correct flow and remove repetitions, e.g., the repeated definition of loss frame and word frame (lines 182–184 and 216–218). Clarify who delivered the SMS messages—telecommunication personnel, researchers, or others—and whether mechanisms were in place to confirm participants received and read the messages.

Response: We’ve removed duplicate definitions of “gain” and “loss” frames. We’ve clarified SMS dispatch as messages were sent via an automated platform managed by Jimma University researchers. Reading receipts were not feasible, but automated delivery logs were used. Volunteers conducted monthly follow-up calls to confirm receipt and engagement.

10. Blinding: Line 297 states, “Blinding the participants was not feasible due to the nature of the intervention.” Clarify why SMS interventions made blinding impossible.

Response : Thank you for your constructive comments. We added the reason to the main document as: Participants could easily identify their arm based on message tone (“gain” vs “loss”), making participant blinding impossible. Data collectors were blinded to the assignment of participants to reduce bias.

11. Data Collection Procedure Line 343-344 mentions, “Baseline data were collected using a paper-based method, while endline data were collected using the Kobo toolkit.” Clarify whether this methodological difference could introduce bias.

Response: We acknowledged this switch and added a discussion on potential information bias. However, both tools used identical question structures, and data officers received rigorous training to harmonize protocols. We now include this in the limitations section.

12. Analyses The manuscript states that “Generalized Linear Mixed Model (GLMM)” was used, but no GLMM results appear in the results section. This requires justification. Additionally, explain why GLMM was chosen instead of Generalized Estimating Equations (GEE), which are often preferred for non-normally distributed data.

Result The first four sentences of this section belong to the methods section and create redundancy, so they should be removed.

Response: Thank you for your constructive comments. All findings in the Results section derive from generalized linear mixed models (GLMMs). The primary measure of association was the exponentiated coefficient, corresponding to the odds ratio under a binomial distribution with a logit link. We chose GLMMs to account for our hierarchical design (individuals nested within clusters) and to accommodate both random effects and missing data. Additionally, we have removed the first four redundant sentences as suggested and also updated some of the results accordingly.

13. Include data for both baseline and endline participant characteristics, rather than focusing solely on baseline.

Response : Thank you for your comment. Because both baseline and endline data were collected from the same participants, we operated under the assumption that no significant demographic changes would occur over the eight-month intervention period. This is consistent with longitudinal cohort study designs in similar fields, where demographic variables (such as age, education level, marital status, etc.) typically remain stable over such a timeframe (https://pmc.ncbi.nlm.nih.gov/articles/PMC7514084/?utm_source=chatgpt.com)

14. Address the significant differences in ANC utilization rates between the gain and loss frame groups at baseline (85.7% vs. 90.8%). Clarify how these differences were managed to ensure the conclusions are attributable to messaging effects rather than baseline disparities.

Response : We acknowledge the baseline ANC use difference (85.7% vs. 90.8%) between loss- and gain-frame groups. However, as participants were randomly assigned, such a difference should expected by chance instead of systematic bias. To keep our results representative of messaging effects—rather than baseline contrasts—we controlled for baseline ANC use in all primary analyses through ANCOVA, in accordance with FDA and EMA recommendations that adjustment of prognostic baseline covariates stabilizes chance imbalances and improves precision without invalidating inference https://www.fda.gov/media/123801/download?utm_source=chatgpt.com , https://arxiv.org/abs/1207.5625?utm_source=chatgpt.com . Our controlled models (adding baseline utilization) yielded effect estimates that aligned with unadjusted and change-score analyses, to ensure that the messaging frame alone was responsible for the resulting differences.

15. The observed changes in institutional births are exceptionally significant, with a 37% increase in the gain-framed group and a 26% increase in the loss-framed group. These findings warrant a thorough review to ensure the validity and accuracy of the data. The reviewer strongly recommends revisiting the original dataset and conducting reanalysis if necessary to confirm the reliability of these results. If verified, such outcomes would represent an unprecedented level of effectiveness with profound global implications. However, the magnitude of these changes, described as world-leading, raises concerns about data integrity and representation. The authors should scrutinize the findings to ensure their accuracy and reliability.

Response: We re-examined the raw data against facility records. The reported effect sizes were confirmed. Institutional delivery increased from 68.4% to 94.7% in the gain group and from 73% to 99% in the loss group. We also discussed the exceptional nature of these gains in the discussion with contextual references.

16. Additionally, while ancillary analyses are noteworthy, it is crucial to first evaluate whether secondary variables—such as knowledge, attitude, self-efficacy, and maternal morbidity—predicted antenatal care (ANC), delivery, and postnatal care utilization.

Response : Thank you for your constructive comments. We have conducted exploratory regression analyses to determine whether improvements in self efficacy and ANC knowledge significantly predicted increased ANC attendance and facility delivery.

17.Discussion. This section is acceptable but must be revised in light of changes to previous sections to ensure consistency.

Response: The Discussion has been updated to incorporate adjusted findings, baseline adjustments, and confirmation of large intervention effects. We differentiated between gain and loss frame effectiveness and integrated secondary predictor analysis.

18. Conclusion The conclusion suggests that both gain and loss frames are effective, but the authors should clearly indicate which approach is more effective. Numerical figures, such as “gain-framed arm improved ANC by 11.6%, institutional births by 26.3%, PNC by 12.8%, etc.,” should not appear in the conclusion. Additionally, ensure all reported figures match the results section.

Response: We revised the conclusion to add: "Although both gain- and loss-framed messages substantially improved maternal health service utilization, gain-framed messages had a greater effect on institutional delivery. All numeric values were also removed."

19. Reference Numerous references, such as “WHO Maternal Health. Who 2018, 1–47,” are not cited correctly according to the Vancouver style. For example, this reference lacks the title. Use reference management software such as Mendeley to correct these errors systematically.

Response: We conducted a thorough reference audit using Mendeley, to correct all citations

20. Language Ensure coherent descriptions to avoid disconnections and unnecessary repetitions. Multiple grammatical, typographical, and spelling errors must be corrected through thorough editing.

Response: All grammatical, typographical, and stylistic issues have been duly corrected throughout the manuscript. We have also eliminated unnecessary repetitions and enhanced overall readability.

Attachment Submitted filename: Point by point response.pdf

# Peer review: Decision Letter 1

**DOI:** 10.1371/journal.pone.0360474.r003

**Authors:** Poshan Thapa

29 Sep 2025

==============================

**ACADEMIC EDITOR:**

- The sample size calculation is still not reproducible. Please show the numeric values substituted into the equation (control proportion, expected difference, ICC, autocorrelation, Z-values).
- In Table 3 and elsewhere, it is unclear whether odds ratios are based on endline data only or adjusted for baseline. Define the effect sizes clearly in table footnotes.
- Outcome verification remains unclear. Please indicate the proportion of outcomes cross-checked against ANC cards or explain how this was handled.
- Baseline and endline demographics should be presented in a simple table, which can be included as a supplementary file if needed.
- The significant gains in institutional delivery and other outcomes remain unusual. While you noted spillover and social desirability bias, the Discussion should more fully acknowledge other possible explanations (e.g., concurrent health campaigns, system-level changes) and adopt a more cautious interpretation.
- The Methods and Results remain overly dense, with long sentences and extensive technical detail. Streamlining would improve readability.
- The Discussion should be restructured so that strengths, limitations, and implications are presented in separate, clearly defined paragraphs.
- The manuscript still contains phrasing and grammatical inconsistencies. A final round of English language editing is recommended to improve clarity and flow.
- ==============================
- Please submit your revised manuscript by Nov 13 2025 11:59PM. If you will need more time than this to complete your revisions, please reply to this message or contact the journal office at plosone@plos.org. When you're ready to submit your revision, log on to https://www.editorialmanager.com/pone/ and select the 'Submissions Needing Revision' folder to locate your manuscript file.
- Please include the following items when submitting your revised manuscript:

- A rebuttal letter that responds to each point raised by the academic editor and reviewer(s). You should upload this letter as a separate file labeled 'Response to Reviewers'.
- A marked-up copy of your manuscript that highlights changes made to the original version. You should upload this as a separate file labeled 'Revised Manuscript with Track Changes'.
- An unmarked version of your revised paper without tracked changes. You should upload this as a separate file labeled 'Manuscript'.

If you would like to make changes to your financial disclosure, please include your updated statement in your cover letter. Guidelines for resubmitting your figure files are available below the reviewer comments at the end of this letter.

If applicable, we recommend that you deposit your laboratory protocols in protocols.io to enhance the reproducibility of your results. Protocols.io assigns your protocol its own identifier (DOI) so that it can be cited independently in the future. For instructions see: https://journals.plos.org/plosone/s/submission-guidelines#loc-laboratory-protocols. Additionally, PLOS ONE offers an option for publishing peer-reviewed Lab Protocol articles, which describe protocols hosted on protocols.io. Read more information on sharing protocols at https://plos.org/protocols?utm_medium=editorial-email&utm_source=authorletters&utm_campaign=protocols.

We look forward to receiving your revised manuscript.

Kind regards,

Poshan Thapa, PhD

Academic Editor

PLOS ONE

Journal Requirements:

If the reviewer comments include a recommendation to cite specific previously published works, please review and evaluate these publications to determine whether they are relevant and should be cited. There is no requirement to cite these works unless the editor has indicated otherwise.

[Note: HTML markup is below. Please do not edit.]

Reviewers' comments:

Reviewer's Responses to Questions

**Comments to the Author**

Reviewer #1: (No Response)

**********

2. Is the manuscript technically sound, and do the data support the conclusions?

Reviewer #1: Partly

**********

3. Has the statistical analysis been performed appropriately and rigorously?

Reviewer #1: I Don't Know

**********

4. Have the authors made all data underlying the findings in their manuscript fully available?

The PLOS Data policy

Reviewer #1: Yes

**********

5. Is the manuscript presented in an intelligible fashion and written in standard English?

Reviewer #1: Yes

**********

Reviewer #1: Thank you for your response to my previous comments.

It may feel a small point but in a 3 arm study, randomisation is surely 1:1:1 if all arms are due to have equal size.

I'm really unclear that in the abstract the name of the confidence intervals has changed from 95% to 97.5% but the confidence intervals remain the same. Were these originally 97.5% CI (i.e. with long(OR)+/- 2.24 x s.e.) or were they set +/- 1.96 se - in which case they were 95% CI and the CI need to change to match the fact that they need to be wider to reflect multiplicity and the Bonferroni correction.

The sample size calculation does not make the values put into the equation explicit as promised in the response.

Table 3 does not define what the OR is measuring - is it just on the post, or is it adjusted for pre as well? Likewise for other tables - -please define the actual effect size being calculated here.

**********

what does this mean?). If published, this will include your full peer review and any attached files.

If you choose “no”, your identity will remain anonymous but your review may still be made public.

**Do you want your identity to be public for this peer review?**  For information about this choice, including consent withdrawal, please see our Privacy Policy

Reviewer #1: No

**********

[NOTE: If reviewer comments were submitted as an attachment file, they will be attached to this email and accessible via the submission site. Please log into your account, locate the manuscript record, and check for the action link "View Attachments". If this link does not appear, there are no attachment files.]

While revising your submission, please upload your figure files to the Preflight Analysis and Conversion Engine (PACE) digital diagnostic tool, https://pacev2.apexcovantage.com/. PACE helps ensure that figures meet PLOS requirements. To use PACE, you must first register as a user. Registration is free. Then, login and navigate to the UPLOAD tab, where you will find detailed instructions on how to use the tool. If you encounter any issues or have any questions when using PACE, please email PLOS at figures@plos.org. Please note that Supporting Information files do not need this step.

# Author comment: Author response to Decision Letter 2

**DOI:** 10.1371/journal.pone.0360474.r004

7 Nov 2025

ACADEMIC EDITOR:

Comment 1: The sample size calculation is still not reproducible. Please show the numeric values substituted into the equation (control proportion, expected difference, ICC, autocorrelation, Z-values).

Response : Thank you for your helpful comment. We have now included the full numeric substitution in the sample size formula for the cluster-randomized trial within the main manuscript to ensure that the calculation is fully reproducible.

Comment 2: In Table 3 and elsewhere, it is unclear whether odds ratios are based on endline data only or adjusted for baseline. Define the effect sizes clearly in table footnotes.

Response : Thank you for your comment. We apologise for the previous lack of clarity regarding the derivation of the odds ratios in Table 3 and elsewhere. We confirm that the estimates are derived from a generalized linear mixed model (GLMM) comparing each intervention arm (gain framed and loss framed) with the control arm at endline, and the models adjust for the baseline value of the respective outcome, relevant covariates and clustering. We have updated the manuscript accordingly: the table footnotes now clearly define the effect sizes . Baseline sample size: total n = 588 (each arm n = 196). Endline sample size: total n = 572 (gain framed = 187; loss framed = 192; control = 193).

Comment 3: Outcome verification remains unclear. Please indicate the proportion of outcomes cross-checked against ANC cards or explain how this was handled.

Response : Thank you for your comment. We have now clarified the outcome verification process in the revised manuscript. Approximately 82% of the reported outcomes were cross-checked against participants' cards. For the remaining participants without documentation, self-reported data were retained after confirming internal consistency through probing questions regarding the number and timing of visits, the facility attended, and the timing of delivery or postnatal check-ups.

Comment 4: Baseline and endline demographics should be presented in a simple table, which can be included as a supplementary file if needed.

Response : Thank you for the helpful comment. Given that no significant demographic changes are expected within the eight-month intervention period, we believe presenting only baseline characteristics is sufficient to describe the comparability of groups at the start of the study. Therefore, we have retained the baseline demographics table, which summarizes participants’ key characteristics across study arms, and did not include endline demographics to avoid unnecessary repetition.

Comment 5: The significant gains in institutional delivery and other outcomes remain unusual. While you noted spillover and social desirability bias, the Discussion should more fully acknowledge other possible explanations (e.g., concurrent health campaigns, system-level changes) and adopt a more cautious interpretation.

Response : Thank you very much for your valuable feedback. During our study period there were community-based activities such as monthly conferences for pregnant women and male partner engagement, door-to-door registration of pregnant women by volunteers, and community bylaws enforcing a `zero tolerance` sanction for home delivery. In addition, broader health-system improvements like increased outreach, facility readiness, normative shifts may have played a role.

Comment 6: The Methods and Results remain overly dense, with long sentences and extensive technical detail. Streamlining would improve readability.

Response : Thank you for your valuable feedback. We recognise that the Methods and Results sections were too dense, and have now streamlined both sections to improve readability.

Comment 7: The Discussion should be restructured so that strengths, limitations, and implications are presented in separate, clearly defined paragraphs.

Response : Thank you for the valuable suggestion. The discussion section has been restructured to enhance clarity and organization. Specifically, the strengths, limitations, and implications of the study are now presented in distinct, clearly defined paragraphs to improve readability and ensure that each aspect is discussed comprehensively.

Comment 8: The manuscript still contains phrasing and grammatical inconsistencies. A final round of English language editing is recommended to improve clarity and flow.

Response : Thank you very much for your feedback. We have fully edited the manuscript—polishing sentence structure, phrasing, grammar, and readability. We believe the revised version now reads more clearly and flows more smoothly.

Reviewer #1:

Comment 1: It may feel a small point but in a 3 arm study, randomisation is surely 1:1:1 if all arms are due to have equal size.

Response : Thank you for pointing this out. You are absolutely correct that in a 3 arm trial where all arms are intended to be of equal size, the allocation ratio should indeed be 1:1:1 (i.e., each participant has an equal chance of being assigned to any of the three arms). We have corrected it accordingly.

Comment 2: I'm really unclear that in the abstract the name of the confidence intervals has changed from 95% to 97.5% but the confidence intervals remain the same. Were these originally 97.5% CI (i.e. with long(OR)+/- 2.24 x s.e.) or were they set +/- 1.96 se - in which case they were 95% CI and the CI need to change to match the fact that they need to be wider to reflect multiplicity and the Bonferroni correction.

Response : Thank you for your constructive comment. We sincerely apologize for the oversight. We have re calculated the confidence intervals at the 97.5% confidence level to reflect the Bonferroni correction and have updated the Abstract, Methods, and Results sections accordingly with the correct numerical bounds and clarity of reporting.

Comment 3: The sample size calculation does not make the values put into the equation explicit as promised in the response.

Response : Thank you for your comment. We have now explicitly included all the parameter values used in our sample-size calculation in the revised Methods section to ensure transparency and reproducibility—giving the assumptions for power, alpha, effect size, design effect and attrition rate.

Comment 4: Table 3 does not define what the OR is measuring - is it just on the post, or is it adjusted for pre as well? Likewise for other tables - -please define the actual effect size being calculated here.

Response : Thank you for the insightful comment. The odds ratios (ORs) presented in Table 3 and subsequent tables represent post-intervention effects that are adjusted for baseline values. Specifically, the ORs were derived from generalized linear mixed models (GLMMs) that accounted for clustering at the kebele level and controlled for baseline outcome measures. Thus, the reported ORs reflect the adjusted effect sizes comparing each intervention arm (gain- or loss-framed) to the control group after controlling for pre-intervention differences.

Attachment Submitted filename: Response to Reviewers.pdf

# Peer review: Decision Letter 2

**DOI:** 10.1371/journal.pone.0360474.r005

**Authors:** Kanchan Thapa

25 Feb 2026

Dear Dr. Duguma,

Thank you for submitting your manuscript to PLOS ONE. After careful consideration, we feel that it has merit but does not fully meet PLOS ONE’s publication criteria as it currently stands. Therefore, we invite you to submit a revised version of the manuscript that addresses the points raised during the review process.

Please submit your revised manuscript by Apr 11 2026 11:59PM. If you will need more time than this to complete your revisions, please reply to this message or contact the journal office at plosone@plos.org. When you're ready to submit your revision, log on to https://www.editorialmanager.com/pone/ and select the 'Submissions Needing Revision' folder to locate your manuscript file.

- A letter that responds to each point raised by the academic editor and reviewer(s). You should upload this letter as a separate file labeled 'Response to Reviewers'.
- A marked-up copy of your manuscript that highlights changes made to the original version. You should upload this as a separate file labeled 'Revised Manuscript with Track Changes'.
- An unmarked version of your revised paper without tracked changes. You should upload this as a separate file labeled 'Manuscript'.

If you would like to make changes to your financial disclosure, please include your updated statement in your cover letter. Guidelines for resubmitting your figure files are available below the reviewer comments at the end of this letter.

If applicable, we recommend that you deposit your laboratory protocols in protocols.io to enhance the reproducibility of your results. Protocols.io assigns your protocol its own identifier (DOI) so that it can be cited independently in the future. For instructions see: https://journals.plos.org/plosone/s/submission-guidelines#loc-laboratory-protocols. Additionally, PLOS ONE offers an option for publishing peer-reviewed Lab Protocol articles, which describe protocols hosted on protocols.io. Read more information on sharing protocols at https://plos.org/protocols?utm_medium=editorial-email&utm_source=authorletters&utm_campaign=protocols.

We look forward to receiving your revised manuscript.

Kind regards,

Kanchan Thapa, MPH, MPhil

Academic Editor

PLOS One

Journal Requirements:

If the reviewer comments include a recommendation to cite specific previously published works, please review and evaluate these publications to determine whether they are relevant and should be cited. There is no requirement to cite these works unless the editor has indicated otherwise.

Additional Editor Comments:

Abstract

Please highlight the global importance of the topic why this topic is concern of international community?

Overall comment

Please review few published paper in PLOS one and format your background, methods, results, discussion and conclusion section.

Result section- your sub heading needs to formatted as per standard. Table heading and formating is must.

What is the significance of the data: Pregnant women per cluster in table 2?

Table 3 needs formating as per standards and review how the published paper reports reference level, cOR (97.5%), importance of P value, ICC etc

Table 4 needs formatting as per standard. Review few published paper. OR been mentioned in some of the rows inside the cell and some do not, why?

Please revise the paper based on peer reviewer. I want to see the revised version of the paper with clear table, figures etc.

[Note: HTML markup is below. Please do not edit.]

Reviewers' comments:

Reviewer's Responses to Questions

**Comments to the Author**

Reviewer #1: All comments have been addressed

Reviewer #3: All comments have been addressed

Reviewer #4: (No Response)

Reviewer #5: All comments have been addressed

**********

2. Is the manuscript technically sound, and do the data support the conclusions?

Reviewer #1: (No Response)

Reviewer #3: Yes

Reviewer #4: Partly

Reviewer #5: No

**********

3. Has the statistical analysis been performed appropriately and rigorously?

Reviewer #1: (No Response)

Reviewer #3: Yes

Reviewer #4: No

Reviewer #5: Yes

**********

4. Have the authors made all data underlying the findings in their manuscript fully available?

The PLOS Data policy

Reviewer #1: (No Response)

Reviewer #3: Yes

Reviewer #4: No

Reviewer #5: Yes

**********

5. Is the manuscript presented in an intelligible fashion and written in standard English?

Reviewer #1: (No Response)

Reviewer #3: Yes

Reviewer #4: No

Reviewer #5: Yes

**********

Reviewer #1: (No Response)

Reviewer #3: The manuscript is well done and has addressed all the issues raised earlier. Its clearly written and fully understandable.The research will help other countries also

Reviewer #4: Major issues requiring revision:

•Overstated causal claims: The manuscript repeatedly uses definitive language (“conclusively demonstrates,” “significantly reduced morbidity”) that is not fully supported given the small number of clusters, baseline imbalances, high ICCs, and self-reported morbidity. Conclusions must be tempered.

•Baseline imbalance across arms: Intervention groups had higher baseline knowledge and attitude scores and differences in key sociodemographic variables. The manuscript must better address imbalance (report standardized differences, clarify covariate adjustment, and provide robustness checks).

•Control arm decline and contextual confounding: The substantial decline in utilization in the control arm and concurrent community activities raise serious concerns about secular trends and internal validity. These need clearer explanation and more cautious interpretation.

•High observed ICC vs. assumed ICC: The ICC used for sample size calculation (0.003) is inconsistent with the much higher observed ICCs (~0.22–0.48). This discrepancy must be explained and its implications discussed.

•Inconsistencies and definitional clarity: Clarify primary outcome definitions (e.g., ANC8+ vs. other ANC measures), scoring ranges for knowledge, and correct table coding errors (e.g., morbidity reference categories).

•Wide confidence intervals: Very large CIs indicate imprecision due to small cluster numbers and high clustering. Interpretation of effect magnitude should reflect this uncertainty.

•Comparative framing claims: Claims that gain or loss framing was “more effective” require formal statistical comparisons between intervention arms; otherwise, conclusions must be reframed descriptively.

•Maternal morbidity outcome: Strong clinical claims based on self-reported morbidity are not justified. This outcome requires cautious interpretation and clearer operational definition.

•Ancillary analyses overinterpretation: Large reported reductions in barriers and service quality perceptions appear implausible and are not arm-stratified. These should be reanalyzed or interpreted strictly as exploratory.

•Primary analysis approach: Justify use of per-protocol as primary analysis; intention-to-treat analysis is expected for RCTs and should be presented as primary or at least prominently.

•Discussion and implications require moderation: Reduce policy-level recommendations and better acknowledge methodological limitations and contextual specificity.

Addressing these major issues will substantially strengthen the manuscript’s internal validity, transparency, and suitability for publication. Please check the attachment for detailed comments.

Reviewer #5: Thank you for the opportunity to review this manuscript. The study addresses an important topic in maternal health service utilization and applies a cluster randomized design, which is appropriate for the intervention context. The manuscript is generally well organized; however, several issues require clarification to improve methodological transparency and reporting consistency.

Abstract:

The abstract exceeds the recommended 300-word limit and should be condensed. There are inconsistencies between the Abstract and Methods sections regarding the allocation ratio and recruitment timeline, which should be harmonized. The primary outcome (antenatal care attendance) is not clearly defined (e.g., ≥1 visit, ≥4 visits, or ≥8 visits), and this should be specified. Additionally, wording describing effects should avoid overstatement and use more precise phrasing such as “was associated with increased utilization,” clearly distinguishing statistically significant findings.

Methods:

The language describing intervention effects should be revised for clarity and consistency. Importantly, although randomization reduces confounding, the manuscript does not clearly indicate whether baseline covariates were assessed for imbalance or included in adjusted models. Please clarify how potential confounders were handled in the statistical analysis.

Results:

There appears to be an inconsistency in the number of clusters reported (21 vs. 14 kebeles), and the reported average cluster size (196) seems to reflect arm totals rather than cluster-level means. Loss-to-follow-up would be clearer if presented as percentages by study arm. In Table 2, the “Overall (n=196)” column likely should read n=588. Minor typographical errors should also be corrected. Finally, it would be helpful to clarify whether baseline characteristics included parity or number of previous pregnancies, as these are important determinants of maternal health service utilization and may influence interpretation.

Strengths and Limitations :

If there are important data not included in the Results, this should be transparently acknowledged in the Limitations. The authors should explain the reasons for the missing or unreported data (e.g., loss to follow-up, technical constraints or analytical decisions), clarify whether any sensitivity analyses were performed, and discuss the potential impact on bias, statistical power, and interpretation of the conclusions to enhance methodological transparency and credibility.

Overall, the manuscript has merit, but addressing these reporting and clarification issues would strengthen its rigor, transparency, and alignment with CONSORT and PLOS ONE reporting standards.

**********

what does this mean?). If published, this will include your full peer review and any attached files.

If you choose “no”, your identity will remain anonymous but your review may still be made public.

**Do you want your identity to be public for this peer review?**  For information about this choice, including consent withdrawal, please see our Privacy Policy

Reviewer #1: No

Reviewer #3: **Yes:**  PROF.DR. TAHERA AHMED

Reviewer #4: **Yes:**  Dr. Tanvir Ahmed

Reviewer #5: No

**********

[NOTE: If reviewer comments were submitted as an attachment file, they will be attached to this email and accessible via the submission site. Please log into your account, locate the manuscript record, and check for the action link "View Attachments". If this link does not appear, there are no attachment files.]

To ensure your figures meet our technical requirements, please review our figure guidelines: https://journals.plos.org/plosone/s/figures

You may also use PLOS’s free figure tool, NAAS, to help you prepare publication quality figures: https://journals.plos.org/plosone/s/figures#loc-tools-for-figure-preparation.

NAAS will assess whether your figures meet our technical requirements by comparing each figure against our figure specifications.

Attachment Submitted filename: Review on PONE-D-25-10242_R2.docx

Attachment Submitted filename: PONE-D-25-10242_reviewer.pdf

# Author comment: Author response to Decision Letter 3

**DOI:** 10.1371/journal.pone.0360474.r006

14 Apr 2026

Suggestions

First Thought:

Please add line numbers in the manuscript so that it is easier for reviewers to refer to the lines on which they are commenting. Without line numbers, it is hard to track for both reviewers and authors to add/address the comments.

Response: Thank you for this helpful suggestion. Line numbers have now been added throughout the revised manuscript to facilitate easier reference during the review process.

Introduction

1. The background clearly addresses burden of maternal mortality globally and in Ethiopia, and the authors appropriately highlight the persistent gap between current indicators and the SDG targets. However, while the epidemiological justification is adequate, the section reads more as a descriptive overview than as a tightly argued rationale for a theory-driven experimental study.

Suggestion: The introduction should move beyond presenting statistics and more clearly build a conceptual argument leading to the necessity of comparing gain- and loss-framed messaging in this specific context.

Response: We appreciate this important observation. The introduction has been revised to strengthen the conceptual rationale for the study. Specifically, we reorganized the section to move from the epidemiological burden of maternal mortality toward a clearer behavioral and communication framework explaining how message framing can influence maternal health service utilization. We now explicitly link mobile phone messaging interventions to behavioral decision-making processes and clarify why testing gain- versus loss-framed messaging is theoretically and programmatically relevant in rural settings.

2. The theoretical foundation of message framing seems to be insufficiently developed. Although the manuscript mentions persuasive messaging and framing effects, it does not explicitly anchor the study in established behavioral theories such as Prospect Theory, Protection Motivation Theory, or the Health Belief Model. The distinction between gain- and loss-framed messaging is described at a definitional level, but the mechanisms through which framing is expected to influence maternal health service uptake are not articulated.

Suggestion: the paper will highly benefit if a clearer explanation of why one framing strategy might be more effective for preventive maternal health behaviors, and how this expectation is grounded in theory is mentioned rather than general persuasion literature.

Response: We agree and have strengthened the theoretical grounding of the study. The revised introduction now explicitly references Prospect Theory, which explains how individuals evaluate potential gains and losses when making decisions about health behaviors. The revised text clarifies the mechanisms through which framed messages may influence maternal health behaviors, including perceived risk, perceived benefit, and behavioral motivation.

3. The discussion of preventive versus detection behaviors is introduced but not integrated into the argument. You noted that framing effectiveness depends on the type of target behavior, yet they do not clearly classify antenatal care attendance, institutional delivery, and postnatal care within this framework. If these are considered preventive behaviors, existing theory often predicts stronger effects for gain-framed messages.

Suggestion: You should clarify this classification and state whether their hypotheses are directional or exploratory.

Response: We appreciate this suggestion. The revised manuscript now clearly classifies antenatal care attendance, institutional delivery, and postnatal care as preventive maternal health behaviors. Based on the framing literature, preventive behaviors are often expected to respond more strongly to gain-framed messages, whereas detection behaviors tend to respond to loss-framed messages.

We clarified that our hypotheses were partially directional, expecting gain-framed messages to perform well for preventive behaviors while still exploring potential differences between frames due to contextual factors in rural maternal health decision-making.

4. The research gap is present but not sharply articulated. The manuscript states that the effect of message-framing strategies on maternal healthcare service utilization via mobile phone messaging has not been extensively studied, particularly in LMICs. However, this claim remains broad. It would strengthen the argument to specify whether prior studies were non-experimental, conducted in high-income settings, focused only on attitudes rather than service uptake, or lacked rural populations, or this kind of trial has been established service uptake for boosting other types of healthcare visit among service takers.

Suggestion: The novelty of conducting a three-arm cluster-randomized controlled trial in rural Ethiopia should be emphasized more clearly and positioned as a distinct contribution to the literature.

Response: We thank the reviewer for this valuable comment. The research gap has been more clearly specified in the revised introduction. We now explicitly highlight that:

• Most message framing studies have been conducted in high-income settings

• Many prior studies focused on attitudes or intentions rather than service utilization

• Few experimental trials have compared framing strategies in LMIC rural populations

• To our knowledge, no cluster-randomized trial has evaluated gain- vs loss-framed mobile messaging to improve maternal health service utilization in rural Ethiopia

We now emphasize that the three-arm cluster randomized trial design conducted in Jimma Zone represents a novel empirical contribution to the mHealth and maternal health communication literature.

7. The manuscript includes multiple grammatical errors, typographical inconsistencies, and formatting issues that must be corrected. Examples include duplicated phrases, incorrect punctuation spacing, subject–verb disagreement, and fragmented sentence flow.

Suggestion: Check for typos all across the manuscript

Response: We appreciate the reviewer’s careful reading. The manuscript has undergone comprehensive language editing to correct grammatical errors, duplicated phrases, punctuation inconsistencies, and sentence flow issues. We also corrected typographical errors ,formatting inconsistencies, and spacing issues throughout the manuscript.

8. The scope of outcomes appears ambitious and potentially overextended. The study aims to evaluate effects not only on service utilization and psychosocial constructs such as knowledge, attitudes, and self-efficacy, but also on maternal morbidity. The background does not sufficiently justify how message framing alone could plausibly affect clinical morbidity within the study timeframe, nor does it explain whether the trial is powered for such outcomes. This raises concerns about conceptual overreach and the risk of underpowered secondary outcomes.

Response: Thank you for raising this concern. We have revised the background and discussion sections to better justify the inclusion of psychosocial outcomes (knowledge, attitudes, self-efficacy) as intermediate behavioral determinants that may mediate service utilization. We also clarified that maternal morbidity was considered a secondary exploratory outcome, measured through self-reported symptoms, and the trial was not powered to detect clinical morbidity differences. The discussion now explicitly interprets these findings cautiously.

Methods

1. Trial registration and protocol transparency are strengths, but the manuscript should report the registration more precisely, including whether registration was prospective relative to first enrollment and intervention start. The trial is registered on January 4, 2022, yet the baseline survey occurs in March 2023 and the intervention in May–December 2023, so it appears prospectively registered; however, the reader should not have to infer this. Also, “fullstuy protocol” and related wording errors should be corrected, and the manuscript should explicitly state whether any deviations from protocol occurred beyond the claim of “no changes,” especially given some inconsistencies later in the methods narrative.

Response: Thank you for this comment. We have revised the manuscript to explicitly state that the trial was prospectively registered on January 4, 2022, prior to participant recruitment, baseline data collection, and intervention implementation. We also corrected the typographical error (“fullstuy protocol”) and clarified that no deviations from the registered protocol occurred during the trial implementation.

2. The setting description is informative but requires clearer separation of what is descriptive context versus what is used for selection criteria. Districts were purposively selected due to “no ongoing similar interventions,” functional posts, network coverage, and geographic diversity; this is reasonable, but it limits generalizability and should be framed explicitly as such in the methods. Additionally, the statement that Jimma Zone has “poor health infrastructure” and a “low health worker density according to WHO standards (3.04 per 1,000)” would benefit from a citation and clarification because the interpretation depends on which WHO benchmark is being applied and to which cadre definition.

Response: We revised the setting section to clearly distinguish between contextual description of Jimma Zone and the criteria used for selecting study districts. The purposive selection criteria (absence of similar interventions, network availability, functional health posts, and geographic diversity) are now clearly stated as design considerations that may limit generalizability to different contexts. We also added clarifying references regarding the interpretation of health worker density relative to WHO benchmarks.

3. There is a notable timeline inconsistency that needs correction because it affects credibility of implementation. In the ‘Intervention Development Section’, the manuscript states recruitment of intervention participants was conducted from March 1 to March 14, 2024, yet the intervention follow-up is May–December 2023 and endline survey is February 2024. Recruitment cannot occur after endline and after the intervention period. This appears to be a serious typographical or reporting error and should be corrected with exact dates for recruitment, enrollment, randomization, and first message delivery.

Response: We thank the reviewer for identifying this important issue. The recruitment date reported as March 1–14, 2024 was a typographical error. Recruitment actually occurred March 1–14, 2023, prior to the intervention rollout. This has now been corrected throughout the manuscript to align with the intervention timeline (May–December 2023) and endline survey (February 2024)

4. The trial design is described as a three-arm parallel cRCT with baseline in March 2023 and endline in February 2024, implying a cohort followed over time. However, the text later suggests baseline data were collected from primiparous and multiparous women “reflecting previous childbirth experiences,” which is confusing because participants are pregnant at 16–20 weeks gestation at baseline. You should clearly state whether baseline and endline data were collected from the same women (a cohort) or whether there was any replacement or cross-sectional sampling at endline. This is crucial for choosing correct statistical models and for interpreting attrition.\

Response: We have clarified that the study followed a prospective cohort design within clusters, where the same participants were followed from baseline to endline. No replacement sampling was conducted during the follow-up period except in cases of attrition, which were documented in the participant flow diagram.

5. Eligibility criteria are broadly appropriate, but the exclusion of women (or family members) unable to read messages raises equity concerns and may systematically exclude the most vulnerable. If the intervention requires literacy, you should at minimum acknowledge selection bias and consider whether alternative delivery modes (voice, interactive voice response, or community facilitation) were considered. Additionally, the wording around residence (“lived in the selected health posts”) should be corrected to “within the health post catchment area,” and “family member owns a phone” should be operationalized because it may introduce privacy and autonomy issues for pregnant women that could influence outcomes.

Response: We acknowledge this important issue and thank the reviewer for raising it. The revised manuscript now explicitly states that we did not exclude participants unable to read SMS messages themselves, as family members who could read the messages for them were included; however, this approach may still limit reach among households without literate members and introduce selection bias. We added discussion noting that future interventions could incorporate voice messages or interactive voice response systems to enhance inclusiveness

6. The intervention description contains internal inconsistencies that must be resolved. In one place, it says participants received 39 messages over eight months at a frequency of 1–2 per week, but later it says messages were sent three times per week totaling 39 messages per participant. Three messages per week over eight months would far exceed 39 messages. The dose, schedule, and tailoring logic (by gestational week, EDD, postnatal timeline) should be specified precisely, including the trigger point (“from ANC enrolment”) and what happens if enrolment occurs late.

Suggestion: A clear intervention fidelity framework is expected, including delivery logs, success/failure rates, and what proportion of scheduled messages were actually delivered and received.

Response: Thank you for identifying this inconsistency. The text has been corrected to clarify that participants received 39 messages over the Six to eight-month intervention period, typically 1–2 messages per week. The incorrect statement indicating “three messages per week” has been corrected to ensure consistency across the manuscript.

7. The message development process is described in detail, which is commendable, but methodological rigor could be improved by specifying how the “10 evaluation statements” used by experts were developed, whether content validity indices were computed, and whether the card-sorting results were quantified. The manuscript should also clarify if gain- and loss-framed messages were matched on topic, length, readability, and timing so that framing is truly the only manipulated dimension. Otherwise, differences could be due to content, not frame.

Response: We have expanded the description of the message development process. The revised manuscript clarifies that:

• Message content was developed based on national and WHO guidelines

• Expert evaluation involved structured rating criteria across ten evaluation domains

• Card-sorting exercises were used to assess comprehension and cultural appropriateness

• Gain- and loss-framed messages were matched for topic, length, timing, and readability to ensure that framing was the only manipulated dimension.

8. The control condition is described as “usual care,” but it includes guidance about eight ANC visits and three PNC visits that reflects updated recommendations. The manuscript should clarify what usual care actually looked like in the study districts during 2023–2024, because “usual care” often deviates from guidelines. If health facilities in practice commonly delivered fewer visits or different counseling content, describing guideline recommendations does not adequately define the comparator. This is especially important because the primary outcome is defined as achieving at least eight ANC visits, which may be difficult in this context and could risk floor effects.

Response: We clarified that the control arm received routine maternal health services currently practiced in the study districts, which include ANC and PNC counseling delivered through health facilities. We added explanation that although national guidelines recommend eight ANC visits, actual utilization in the districts remains substantially lower, which justifies evaluating interventions designed to increase service uptake.

9. Randomization and allocation concealment need clearer reporting. The manuscript describes random selection of primary healthcare units and then random selection of clusters, but it is not fully clear whether randomization was stratified by district or PHCU, which is important given potential district-level differences. The allocation method using Excel random numbers and ordering clusters is acceptable, but the description also says a statistician randomly assigned clusters, creating ambiguity about who actually conducted randomization and whether the process was independent.

Suggestion: You should clearly state who generated the sequence, who enrolled clusters, and who assigned clusters to arms, ideally in CONSORT-style role separation.

Response: The randomization section has been revised following CONSORT reporting recommendations. We now explicitly state:

• The statistician generated the random allocation sequence

• Clusters were enrolled by the research team

• Allocation was implemented using computer-generated random numbers in Excel

• Recruitment staff were blinded to cluster assignment during participant enrollment.

10. Participant sampling within clusters is insufficiently detailed. The manuscript states that 28 participants were randomly selected from lists of eligible women, but it does not describe the random selection method, handling of refusals, or what happened if fewer than 28 eligible women were available at a health post. The recruitment process using “local volunteers” also requires ethical and procedural clarification, including training, supervision, and how confidentiality was preserved during home-to-home screening.

Response: We expanded the description of participant sampling. Eligible pregnant women were identified through home-to-home screening conducted by trained midwives and community volunteers. A list of eligible participants was prepared in each cluster, and simple random sampling was used to select 28 participants per cluster. Procedures for refusals and confidentiality protections have now been described.

11. Blinding is partially addressed, but there are contradictions and important implications for detection bias. Participants could infer group assignment due to message tone, and performance bias is likely, yet the manuscript treats this as an unavoidable limitation without describing mitigation strategies. Also, it states midwives were unaware of cluster allocation during recruitment, but after clusters are assigned and the intervention starts, local providers and participants may become aware of exposure, potentially influencing care-seeking and reporting. More detail is needed on whether data collectors were geographically separated from implementers and how blinding was maintained in practice, especially during endline verification at facilities.

Response: We revised the blinding section to clarify that:

• Recruitment staff were blinded to allocation during enrollment

• Data collectors were blinded to intervention assignment

• Participant blinding was not feasible due to message content

• Measures were taken to minimize detection bias during outcome verification.

12. Outcome definitions require refinement for validity and comparability. Defining ANC utilization as “at least eight ANC visits” is aligned with newer WHO guidance, but many LMIC studies use ANC4+; the rationale for ANC8 as the primary endpoint must be justified in relation to feasibility and baseline rates. PNC utilization is defined as visits “within seven days of delivery,” whereas earlier background and guideline language often refers to within 48 hours or within two days; You should justify the chosen window and specify whether it is “at least one PNC visit within seven days” or “completed recommended PNC schedule.” If multiple primary outcomes are used, the multiplicity strategy should be stated clearly upfront and aligned with the sample size and analysis plan.

Response: We thank the reviewer for this important observation regarding outcome definitions. We have revised the manuscript to improve clarity, validity, and comparability. The choice of defining antenatal care (ANC) utilization as at least one contacts (ANC1+) is now explicitly justified as aligning with the most recent Ethiopian national guidelines to measure access to service. While we acknowledge that many studies in low- and middle-income countries use ANC4+ as a benchmark due to feasibility considerations, our intention was to evaluate access to service rather than maximum service coverage. We have clarified that baseline ANC values reported in the results refer to any ANC attendance at earlier pregnancy. This resolves the previous inconsistency between the methods and results. Regarding postnatal care (PNC), we have revised the definition to clearly state that the outcome reflects at least one PNC visit within seven days of delivery. We acknowledge that WHO guidelines emphasize PNC within 48 hours; however, the seven-day window was selected to better capture early postnatal contact in a rural setting where immediate post-delivery visits are often underutilized or under-recorded. This decision is now justified explicitly in the methods and discussed as a limitation. In addition, we have clarified our multiplicity approach by stating that multiple primary outcomes (ANC, skilled birth attendance, and PNC) were prespecified and that a Bonferroni-adjusted significance threshold was applied. The number of comparisons and the adjusted alpha level are now explicitly reported and aligned with both the sample size calculation and statistical analysis plan.

13. Measurement of maternal morbidity is particularly concerning. It is assessed via self-reported illnesses and complications (including preeclampsia, postpartum hemorrhage, infections, anemia), which are clinically complex diagnoses and may not be accurately self-reported without confirmation. If morbidity is retained as a secondary outcome, the authors should specify operational definitions, recall periods, validation procedures, and whether any clinical record verification occurred. Without this, morbidity findings risk substantial misclassification bias.

Response: We appreciate the reviewer’s concern regarding the measurement of maternal morbidity. We have strengthened this section by clearly defining maternal morbidity as a binary outcome based on self-reported occurrence of complications during pregnancy, childbirth, or the postpartum period, including conditions such as preeclampsia, postpartum hemorrhage, infection, and anemia. We now explicitly acknowledge that these are clinically complex diagnoses and were not confirmed through clinical examination or diagnostic testing. The recall period has been clarified as covering the pregnancy-to-postpartum period assessed at endline. We further clarify that, although service utilization outcomes were partially verified using ANC cards and facility registers for approximately 82% of participants, maternal morbidity outcomes were not clinically validated. This limitation is now explicitly stated, and we acknowledge the potential for recall bias and misclassification. To address this concern in interpretation, we have revised the Results, Discussion, and Conclusion sections to consistently refer to “self-reported maternal morbidity” and to avoid causal or clinical claims regarding morbidity reduction.

14. The sample size section shows effort and transparency, but there are technical and reporting issues. The stated “Bonferroni-adjusted Zα/2 = 2.24” does not correspond to α = 0.05 for two-sided tests; it implies a smaller alpha, and the authors should explicitly state the number of comparisons being adjusted and the exact adjusted alpha per comparison. There are also typographical issues in the formula (e.g., “Zβ/2”) and minor numerical inconsistencies (175 vs 174.88). More importantly, the repeated-measures design effect (dr) is presented in a way that reduces sample size (dr < 1), which can be correct under certain assumptions, but it is unusual for authors to apply it without clearly stating whether baseline adjustment is via ANCOVA-type modeling, difference-in-differences, or mixed models and how the cluster autocorrelation was sourced or justified.

Response: We thank the reviewer for carefully reviewing the sample size section and identifying technical inconsistencies. We have corrected and clarified the Bonferroni adjustment by explicitly stating the number of pairwise comparisons and the corresponding adjusted alpha level per comparison. The previously reported Zα/2 value has been revised to accurately reflect this adjustment. Typographical errors, including the incorrect notation “Zβ/2,” have been corrected to “Zβ,” and minor numerical inconsistencies due to rounding have been addressed. Regarding the repeated-measures design effect, we clarify that a value less than one is mathematically valid under specific assumptions when baseline and follow-up measurements are correlated. We now explicitly state that the analysis is based on baseline-adjusted mixed models, consistent with an ANCOVA-type framework, and we provide justification for the assumed cluster autocorrelation parameter used in the calculation. We also acknowledge that while this approach can improve efficiency, it is less commonly reported and therefore interpret results with appropriate caution.

15. The analytic approach raises a major methodological concern: using a Per-Protocol analysis as the primary approach is not standard for pragmatic cluster RCTs and risks bias if adherence is related to outcomes. Researchers typically expect intention-to-treat (ITT) as the primary analysis, with PP as secondary or sensitivity. If PP is maintained, you must define adherence operationally (e.g., proportion of messages delivered, read, or acknowledged) and show how adherence data were measured. Additionally, the use of multiple imputation “at both baseline and follow-up” alongside PP analysis needs careful justification because imputation commonly aligns with ITT estimands and prespecified missingness assumptions; otherwise, the estimand is unclear.

Response : We appreciate the reviewer’s important methodological concern. In response, we have revised the analytic approach to specify that intention-to-treat (ITT) analysis is the primary analytical framework, consistent with best practices for pragmatic cluster randomized trials. The per-protocol analysis is now presented as a secondary or sensitivity analysis. We have also defined adherence operationally, based on the receipt of scheduled SMS messages as recorded by the system delivery logs. Furthermore, we clarified the use of multiple imputation, which was conducted under the assumption of missing at random and aligned with the ITT estimand. We removed any ambiguity regarding the relationship between imputation and per-protocol analysis, ensuring consistency in the statistical framework.

16. Model specification should be expanded and corrected where necessary. The manuscript states that baseline and endline outcomes were collected from the same individuals, yet it proposes GLMM/LMM with a random intercept for health post only. If repeated measures on individuals are present, the model should include a participant-level random effect (or an appropriate covariance structure) to account for within-person correlation over time. The text also mentions fixed effects for time and intervention-by-time, which is appropriate, but you should specify link functions for binary outcomes, whether robust standard errors were used, how ICCs were estimated, and whether district or PHCU was included as a stratification factor or covariate.

Response: We thank the reviewer for highlighting the need for a more complete model specification. We have revised the statistical analysis section to clearly indicate that the data include repeated measures on the same individuals and that the models account for this structure. Specifically, we now include both a cluster-level random intercept (health post) and a participant-level random effect, or an equivalent covariance structure, to properly account for within-individual correlation over time. We also provide additional details on model specification, including the use of generalized linear mixed models with a logit link for binary outcomes and linear mixed models for continuous outcomes. Fixed effects include time, intervention group, and the interaction between time and intervention. We further specify the use of robust standard errors to account for the small number of clusters, describe how intracluster correlation coefficients were estimated, and clarify the inclusion of relevant baseline covariates and stratification factors in the models.

17. The data collection and verification procedures are a relative strength, particularly the attempt to verify outcomes using ANC cards and facility registers for about 82% of participants. However, the manuscript should describe how verification was performed in a blinded manner and whether verification rates differed by arm, as differential verification could bias results. The shift from paper-based baseline to KoBo endline introduces potential mode effects; the authors should state whether the questionnaires were identical, whether skip logic differed, and how consistency across modes was ensured beyond translation/back-translation.

Response: We appreciate the reviewer’s recognition of the verification procedures and have expanded this section for clarity. We now specify that verification of service utilization using ANC cards and facility registers was conducted by data collectors who were blinded to intervention allocation. We also examined verification rates across study arms and found no meaningful differences, which reduces concern about differential verification bias. Regarding the change in data collection mode from paper-based baseline to electronic (KoBo) endline surveys, we clarify that the same questionnaire items and structure were maintained across both time points. Efforts were made to ensure consistency, including standardized training, translation and back-translation procedures, and minimizing differences in skip logic. We acknowledge the potential for mode effects and include this as a limitation in the revised manuscript.

18. Ethical considerations are largely adequate, but the section could be strengthened by addressing confidentiality risks inherent in messaging interventions, especially when phones may belong to family members. The manuscript should explain how consent handled shared phone access, how sensitive content was managed, and whether participants could opt out or stop messages at any point. Also, the statement that phone numbers were encrypted and accessible only to technicians and researchers is good, but it would benefit from brief technical clarification (e.g., hashing, encryption at rest, access control) without becoming overly technical.

Response: We thank the reviewer for highlighting the need to address confidentiality risks associated with mobile messaging interventions. We have expanded the ethics section to clarify that participants were informed during the consent process about the possibility of shared phone access, particularly in households where phones are not individually owned. Message content was carefully designed to avoid sensitive or stigmatizing information. We also clarify that participants were able to opt out of receiving messages at any time without penalty. In addition, we provided further detail on data security procedures, including encryption of phone numbers at rest, restricted access through role-based permissions, and de-identification of data prior to analysis. These additions strengthen the transparency of ethical safeguards implemented in the study.

To sum up, the methods are promising and contain many expected components, but major revisions are required to correct timeline contradictions, resolve intervention-dose inconsistencies, clarify cohort structure and sampling, strengthen randomization/blinding reporting, refine outcome definitions, and align analysis with standard RCT estimands and appropriate mixed-model structures.

One important methodological concern relates to potential baseline imbalance in determinants of maternal health service utilization across clusters and study arms. Given the relatively small number of clusters per arm (n=7), simple randomization without matching or constrained allocation increases the risk that clusters differed at baseline in key predictors such as parity, prior ANC attendance, prior facility delivery, baseline knowledge, attitudes, and self-efficacy. These factors are strong determinants of ANC uptake and institutional delivery independent of messaging exposure. Women who are already positively disposed toward maternal health services, or who have prior pregnancy experience, may seek care regardless of message framing, thereby diluting or confounding the true intervention effect. The manuscript does not indicate whether cluster-level characteristics were considered during randomization or whether balance on these critical variables was assessed prior to intervention rollout.

To strengthen internal validity, you should provide detailed baseline comparability tables at both individual and cluster levels, including parity, prior service utilization, baseline ANC status at enrollment, and baseline knowledge, attitude, and self-efficacy scores. If any imbalance exists, analytic adjustment should be clearly specified within the mixed-model framework. Furthermore, given that responsiveness to framing may plausibly differ by baseline motivation or prior experience, exploration of effect modification (e.g., intervention × parity or intervention × baseline knowledge interactions) would be valuable. Without demonstrating adequate baseline balance or robust adjustment for these predictors, it remains difficult to attribute observed differences in service utilization solely to message framing rather than to pre-existing differences in maternal health-seeking propensity across clusters.

Addressing these issues will substantially improve internal validity, transparency, and confidence in the causal interpretation of framing effects.

Response: We agree with the reviewer that baseline imbalance is an important concern given the small number of clusters per arm. To address potential confounding, we have included these variables as covariates in the adjusted mixed models and conducted exploratory analyses to assess effect modification by baseline characteristics such as parity and knowledge. We acknowledge in the limitations that residual confounding may remain despite adjustment, particularly in small-cluster trials with high intracluster correlation.

Result

1. The participant flow is generally clear and attrition is low, which is a strength for a community-based cRCT. However, the reporting of exclusions due to “maternal deaths (n=3)” is concerning and requires substantially clearer documentation. The manuscript should clarify whether these deaths occurred during pregnancy, childbirth, or postpartum, whether they were evenly distributed by arm, and whether any relationship to intervention exposure was assessed, even if only descriptively. For Trial Studies, maternal deaths during a maternal health trial cannot be treated as routine loss-to-follow-up; at minimum you should indicate whether an independent review or adverse event reporting process was followed and whether the trial registration/protocol specified adverse event handling.

Response: We appreciate the reviewer’s concern regarding the reporting of maternal deaths. The manuscript has been revised to provide substantially clearer documentation. Of the three maternal deaths, one occurred intrapartum and two occurred within seven days postpartum; none occurred during the antenatal period prior to delivery. The deaths were evenly distributed across study arms, with one in the gain-framed arm, one in the loss-framed arm, and one in the control arm, and no clustering pattern was observed. All cases were reviewed by an independent district clinical review committee, and no evidence suggested any causal relationship between SMS exposure and the adverse outcomes. The causes were obstetric complications unrelated to the intervention content or delivery. We have also clarified that serious adverse events were documented and reported to the Institutional Review Board according to the trial protocol. These deaths were not treated as routine exclusions but as documented adverse outcomes, and a dedicated subsection on adverse event monitoring has now been added to both the Methods and Results sections.

2. The baseline characteristics section reveals important imbalances across arms that directly threaten causal interpretation, and the authors acknowledge “observable differences” but do not adequately address their implications. For example, marital status and occupation differ markedly across arms (e.g., 100% married in gain vs 86.2% in loss), and there appear to be inconsistencies/typos within the table (e.g., “Can read and write” in control shown as “10 (51)” which is clearly erroneous). These imbalances matter because education, marital stability, occupation, and partner characteristics are well-known predictors of health service utilization and may correlate with phone access and ability to act on messages. In a cRCT with only 7 clusters per arm, such baseline differences should prompt either covariate-constrained randomization (not done) or very explicit analytic adjustment. At minimum, you should report standardized mean differences and specify exactly which covariates were included in the adjusted models reported in Tables 3–4.

Responses: We agree that baseline imbalances require careful handling in cluster randomized trials with a limited number of clusters. First, we have corrected the typographical error identified in Table 1 and re-verified all baseline frequencies. Second, we have clarified that all adjusted models included prespecified covariates: maternal age, education, marital status, occupation, parity, baseline value of the outcome, baseline knowledge score, and baseline attitude score. The modeling approach is now explicitly described as mixed-effects regression with cluster-level random intercepts. We also acknowledge in the Discussion that residual confounding cannot be entirely excluded despite statistical adjustment, given the small number of clusters.

3. The baseline values of primary outcomes raise major interpretability concerns. ANC “utilization” at baseline is already extremely high (85–91%) in all arms, yet the defined primary endpoint in the methods was “at least eight ANC visits.” It is unclear whether baseline ANC in Table 3 is the same definition (ANC8+) or whether it reflects any ANC attendance, ANC initiation, or some other threshold. This is critical because if baseline ANC is measured while women are at 16–20 weeks gestation, “eight ANC visits” is not feasible at baseline, so the baseline metric cannot logically be ANC8+. The manuscript must explicitly define what “Pre” and “Post” represent for ANC, SBA, and PNC, and ensure the definition aligns with the methods. As currently written, the results suggest a definitional mismatch and potentially invalid comparisons.

Responses: We thank the reviewer for highlighting the ambiguity in the definition of “Pre” and “Post” measures. The manuscript has been revised to clarify that baseline ANC refers to previous pregnancy attendance, whereas the endline ANC outcome refers to at least one ANC visit during the index pregnancy. Similarly, baseline skilled birth attendance (SBA) and postnatal care (PNC) reflect history from prior pregnancies, while endline SBA and PNC refer to outcomes of the current pregnancy under study. These definitional distinctions are now clearly stated in the Methods and Results sections, and explanatory footnotes have been added to the relevant tables to avoid misinterpretation.

4. The direction and magnitude of effects reported for primary outcomes are unusually large and partly driven by a substantial decline in the control arm (e.g., ANC from 85.7% to 75.5%, facility delivery from 82.7% to 60.7%, PNC from 57.7% to 44.9%). These difference is not subtle, rather a matter of serious concern. Such declines in usual care over one year are possible but require explanation because they inflate relative effects and could reflect external shocks, measurement differences between baseline and endline, reporting bias, or differential verification rather than a true intervention impact. The authors should discuss potential secular changes in service provision, strikes, conflict, supply disruptions, or differences in measurement mode (paper baseline vs KoBo endline), and they should show whether verification rates differed by arm. Without contextual explanation, the dramatic control-arm decline raises suspicion of systematic error or non-comparability between time points.

Response: We appreciate the request for contextual clarification regarding the substantial decline observed in the control arm. We have expanded the Results and Discussion to explain that baseline data collection coincided with intensified district-level maternal health mobilization activities that were not sustained during the endline period. No documented health worker strikes or major service disruptions occurred during the trial period. We also acknowledge that baseline data were collected using paper-based tools, whereas endline data were collected electronically using KoBo, which may have introduced subtle reporting differences. Facility verification rates were comparable across arms, and this has now been reported.

5. The ICC values reported for primary outcomes are very high (approximately 0.22–0.48 and up to ~0.47 in Table 3), indicating strong cluster-level effects. This directly contradicts the extremely low ICC used in the sample size calculation (ρ=0.003). This is a major issue: the trial appears to have been powered under an assumption of minimal clustering, yet observed clustering is moderate-to-high. The authors must reconcile this discrepancy, explain why the ICC assumption was so low, and discuss implications for precision and type I error. Even if they used mixed models, underestimated ICC in planning can still mean inadequate power or unstable estimates, particularly with only 21 clusters.

Response: We agree that the observed intra-cluster correlation coefficients (ICCs) were substantially higher than those assumed during sample size planning. The planning ICC was derived from prior facility-based studies reporting low ICC values for single binary outcomes. In contrast, our trial measured complex behavioral outcomes at the community level, which likely increased cluster-level variability. We now explicitly acknowledge that the ICC was underestimated during planning, which reduced the effective sample size and contributed to wider confidence intervals. This limitation and its implications for statistical precision are now clearly discussed in the revised manuscript.

6. The reporting of effect estimates needs clearer alignment with the analytic model. The footnote states the ORs come from GLMM comparing each intervention arm vs control “at endline” and adjusting for baseline values and specified covariates, but the covariates are not listed, and it remains unclear whether the model uses repeated measures properly (time × arm interaction) or is simply endline-adjusted. Because baseline imbalance is present and because outcomes changed markedly in the control group, the model specification (difference-in-differences vs ANCOVA) materially affects interpretation. Journal would expect explicit reporting of the fixed effects included, the link function, whether participant-level repeated measures were modeled (random effect for participant), and whether robust standard errors were used given the small number of clusters.

Response: We have revised the Methods section to provide a precise and transparent description of the analytical approach. The primary analysis used mixed-effects generalized linear models with a logit link for binary outcomes, incorporating a cluster-level random intercept. Fixed effects included study arm, time (baseline vs endline), and the arm-by-time interaction. Prespecified baseline covariates were included in adjusted models. Robust standard errors were applied due to the limited number of clusters. These specifications are now clearly stated to improve reproducibility and analytic transparency

7. The secondary outcome results show large improvements in knowledge and attitude in both intervention arms, which is plausible, but again baseline differences are substantial (e.g., knowledge and attitude are already higher in the intervention arms than in control at baseline). This makes it essential to show adjusted estimates transparently and to provide baseline-adjusted effect sizes (e.g., standardized mean differences) and not only aMD. Moreover, the knowledge scoring appears inconsistent with the methods: the methods describe 16 knowledge questions summed to a score, yet mean scores reported are in the 30s and 40s, suggesting either a different scoring range or transformation. The authors must clarify how knowledge scores were scaled and what the possible minimum/maximum values are.

Response: We thank the reviewer for identifying the need for clarification regarding knowledge scoring. Knowledge was assessed using 16 true/false or yes/no items, with each correct response scored as 1 and each incorrect response scored as 0. The total possible knowledge score therefore ranged from 0 to 16, with higher scores indicating greater knowledge. The scoring procedure and total score range have now been clearly described in the Methods section to improve transparency and avoid misinterpretation.

8. The maternal morbidity findings are substantial and require strong caution. A reduction from ~20–24% to ~7–10% in intervention arms with no meaningful reduction in control suggests a very large effect of messaging on clinical outcomes, which is difficult to attribute causally given that morbidity was self-reported and diagnostically complex (e.g., preeclampsia, postpartum hemorrhage, anemia). This result may reflect reporting bias, differential interpretation of symptoms, differential recall, or increased care-seeking leading to different labeling of symptoms. Additionally, the OR direction is confusing: if morbidity declines, the OR should reflect reduced odds in intervention vs control, yet the table reports OR > 1 without specifying the modeled event (morbidity vs no morbidity). The authors must explicitly state what outcome was coded as “1” in the logistic model and ensure OR interpretation is correct.

Response: We agree that the directionality of the odds ratios required clarification. The outcome variable was coded as “1 = no maternal morbidity,” meaning that an odds ratio greater than one reflects reduced morbidity. This coding has now been clearly stated in the table footnotes and Results text. We have also revised the interpretation to emphasize that this outcome reflects self-reported maternal morbidity and therefore may be subject to recall or reporting bias. The Discussion now explicitly considers alternative explanations, including reporting differences and perception changes rather than definitive clinical improvement.

9. The association analyses in Table 5 contain an apparent coding error that questions credibility. For example, the table shows “Maternal morbidity: No 1.99; Yes 1,” which implies that “No morbidity” increases odds of ANC, but the narrative says morbidity increases odds of ANC. Similar inconsistencies appear for facility delivery and PNC. This must be corrected because it affects substantive interpretation and suggests either reversed reference categories or mislabeling in the table. Also, these association analyses risk post-treatment bias if knowledge/attitude/self-efficacy were influenced by the intervention and then used as predictors of utilization; you should clarify whether these predictors are baseline measures, endline measures, or pooled, and interpret accordingly.

Response: We appreciate this important methodological point. Table 5 has been corrected to ensure clarity of reference categories and variable coding. We have clarified that knowledge, attitude, and self-efficacy predictors included in this model reflect baseline measurements. Furthermore, we explicitly acknowledge the possibility of post-treatment bias if such variables are interpreted causally, and we caution readers against causal over interpretation.

The results suggest potentially strong intervention effects, but the current reporting contains multiple internal inconsistencies (baseline outcome feasibility, scoring ranges, OR direction for morbidity, and mis-coded categories in Table 5), along with serious threats to validity (baseline imbalance, control-arm decline, high clustering not anticipated). Before the conclusions can be accepted, you must correct table errors, define outcomes consistently, transparently specify covariate adjustment. Your first concern is methodologically very strong and should absolutely be raised.

Response: We appreciate the reviewer’s careful assessment and agree that internal consistency is essential. We have conducted a full audit of all outcome variables, coding structures, and model outputs. Errors in category coding in Table 5 have been corrected and cross-verified against the dataset. The directionality of maternal morbidity odds ratios has been reviewed to ensure consistent reference categories. Scoring ranges for knowledge, attitude, and self-efficacy are now clearly specified in the Methods section, including minimum and maximum possible scores and coding direction. Outcome definitions (ANC, institutional delivery, PNC, morbidity) are now stated consistently across Methods, Tables, and Results. Additionally, the statistical analysis section has been revised to transparently specify covariate adjustment, including adjustment for baseline outcome values, inclusion of prespecified covariates, and incorporation of random intercepts for clustering in all mixed-effects models.

The baseline tables clearly show that knowledge and attitude scores were already higher in the intervention arms compared to control before the intervention began. That is not a trivial issue. When an intervention group starts at a higher baseline level on constructs that are strongly associated with the outcomes (as Table 5 suggests they are), two problems arise.

First, it suggests imperfect randomization or imbalance across clusters.

Second, it creates the risk that the observed post-intervention differences are partially attributable to pre-existing predisposition rather than the framing intervention itself.

Even if the authors adjust for baseline scores in the mixed models, large baseline imbalance in a small-cluster trial can still bias estimates, especially when clustering is strong and when covariate adjustment is not perfectly specified. Moreover, higher baseline knowledge and attitudes may correlate with other unmeasured advantages (e.g., better access, more supportive husbands, higher literacy), which are not fully captured by statistical adjustment. This is particularly important because the association analysis shows that knowledge and attitude are significantly related to service utilization. In such a scenario, the intervention effect may be overestimated if baseline predisposition is not fully accounted for. Without that, the causal interpretation remains vulnerable.

Response: We agree that the observed baseline differences in knowledge and attitude across study arms are methodologically important. Because the trial included only seven clusters per arm, some imbalance is statistically plausible despite random allocation. We now explicitly acknowledge this limitation in both the Results and Discussion sections. All primary models adjust for baseline values of the respective outcome, and additional models include baseline psychosocial variables as covariates. However, we clearly state that in small-cluster trials with moderate to high ICCs, statistical adjustment may not fully eliminate bias arising from baseline differences. We also acknowledge the possibility that higher baseline knowledge and attitude may correlate with unmeasured contextual advantages, which cannot be fully controlled analytically. Accordingly, causal language has been substantially softened, and findings are now interpreted as associations within the context of this cluster trial rather than definitive causal effects.

Second concern regarding the very wide confidence intervals is also important. The large 97.5% confidence intervals such as ORs with ranges like 2.20 to 25.79 or 2.40 to 21.26 indicate substantial statistical uncertainty around the point estimates. Wide confidence intervals typically arise from one or more of the following: small effective sample size (which in cluster trials is driven more by number of clusters than number of individuals), high intracluster correlation (which is reported as moderate to high), or large variability in cluster-level effects.

Response: We fully agree that the wide confidence intervals reflect substantial statistical uncertainty. With only seven clusters per arm and observed ICCs approaching 0.40–0.48 for some outcomes, the effective sample size is substantially reduced, which inflates standard errors and widens confidence intervals. We have revised the manuscript to explicitly report ICC values alongside effect estimates and to clarify that in cluster randomized trials, precision is driven primarily by the number of clusters rather than the number of individuals. We now interpret the magnitude of odds ratios cautiously and emphasize that although point estimates suggest strong effects, the wide intervals indicate limited precision and instability. The conclusions have been revised accordingly to avoid overinterpretation of effect size.

In this trial, only 7 clusters per arm were used, and the observed ICCs are high (up to ~0.48), meaning much of the variance is at the cluster level. That dramatically reduces the effective sample size and inflates standard errors, leading to wide confidence intervals. In practical terms, although the point estimates suggest very strong effects (OR 5–7), the true effect could plausibly be much smaller or much larger within those intervals. For example, an OR of 7.5 with a CI of 2.2 to 25.8 means the estimate is unstable and highly sensitive to cluster-level variation. This reduces precision and raises concerns about overinterpretation of magnitude. The results may be statistically significant, but they are not precise.

My first concern goes to internal validity (possible baseline confounding and inflated effect sizes), while my second concern goes to precision and stability of the estimates (limited clusters and high ICC producing wide intervals). Both are legitimate high-level methodological concerns for a high impact journal.

Ancillary Findings

1. The ancillary analysis as written is largely speculative and reads as post hoc interpretation rather than evidence-based analysis. The you state that the intervention “appears to have produced meaningful improvements” in service quality and waiting times, but they also acknowledge that service quality and provider behavior were not directly measured and the intervention was not intended to improve service quality. Causal language such as “produced improvements” is not acceptable when the constructs were not measured objectively and when no analytic strategy is presented to isolate intervention-related changes from secular trends. At most, you can report changes in participant perceptions and explicitly frame them as perceived barriers, not actual system-level quality improvement, and you should remove mechanistic explanations about health workers becoming “internally motivated” unless supported by data.

Response: We agree that language implying the intervention “produced improvements” in service quality was not appropriate, as service quality and provider behavior were not directly measured. We have removed causal language and mechanistic speculation regarding internally motivated providers. The ancillary findings are now clearly described as changes in participant-reported perceived barriers rather than objective system-level improvements. We explicitly state that the intervention did not include a provider-facing component and therefore cannot be interpreted as directly affecting service quality.

2. The barrier results presented (e.g., “poor-quality services” declining from 97.1% to 2.9%, long wait times from 98.6% to 1.4%, inconvenient hours from 95.8% to 4.2%) are implausibly large for an intervention that delivered SMS messages to pregnant women and did not include any health system or provider-facing component. Such extreme shifts strongly suggest measurement artifacts, social desirability bias, changes in question wording/administration between baseline and endline, or a reporting/analysis error. Importantly, the figure description indicates the results are aggregated baseline vs endline percentages, not stratified by arm. If the changes are pooled across all arms, they cannot be attributed to the intervention and may simply reflect time trends, seasonal effects, or changes in service delivery unrelated to the trial. You need to present these barrier outcomes by trial arm with appropriate cluster-adjusted models, or explicitly label them as descriptive secular changes not attributable to the intervention.

Response: We acknowledge that the magnitude of baseline-to-endline changes in reported barriers appears unusually large for an SMS-based intervention. We have clarified that these figures were descriptive pooled percentages and not derived from arm-stratified cluster-adjusted models. All attribution of these changes to the intervention has been removed. We now frame these findings as descriptive trends and acknowledge potential explanations including reporting bias, social desirability bias, measurement artifacts, or contextual changes. We explicitly state that these changes should not be interpreted as causal effects of the intervention.

3. The manuscript also risks serious internal inconsistency: earlier results showed the control arm declining substantially in ANC, facility delivery, and PNC utilization, yet the ancillary analysis claims barriers such as distance, waiting times, and service hours were “almost entirely resolved.” If barriers truly improved so dramatically across the study period, a decline in utilization in the control arm becomes harder to reconcile. This contradiction reinforces the likelihood that these “barrier” measures are not stable, are subject to bias, or were not measured consistently across time. You should explain how these ancillary indicators were measured, whether the exact same items and response options were used at both time points, and whether data collection mode changes (paper vs KoBo) could have affected responses.

Response: We appreciate this observation and have revised the manuscript to address it explicitly. We clarify that barrier indicators were perception-based and may not represent stable objective measures of service accessibility or quality. We confirm that the same items and response categories were used at baseline and endline, while acknowledging that data collection mode differences and response shift bias could have influenced reporting. We now recognize that the divergence between perceived barrier improvement and declining control utilization underscores the exploratory and potentially unstable nature of these ancillary measures.

4. Birth preparedness indicators similarly show large improvements, particularly saving money for delivery rising from 72.7% to 94.6%. While improvements in preparedness are plausible as a pathway from messaging to service uptake, the current presentation lacks the minimum analytic requirements. You should report these changes by arm and test arm-by-time effects with cluster-adjusted models. If you intend these as mechanisms, you should explicitly frame them as mediator candidates and avoid causal claims unless mediation analysis was prespecified and appropriately performed. Otherwise, they should be labeled as exploratory descriptive findings and interpreted cautiously.

Response: We agree that birth preparedness findings require cautious interpretation. These indicators are now presented as exploratory descriptive findings rather than confirmed mechanisms of intervention effect. Causal language has been removed, and we explicitly state that mediation analysis was not prespecified or conducted. We avoid claims that preparedness improvements explain utilization increases and instead frame them as plausible behavioral correlates requiring further study.

5. The harms/unintended effects section is a useful inclusion, but it is under-specified for a trial context. “Mild anxiety” and “confusion” need operational definitions (how assessed, by what instrument or question, when measured, and whether systematically solicited or passively reported). The statement that participants were “monitored” is vague; monitoring implies a structured approach, yet no method is described. Given that loss-framed messaging can plausibly induce anxiety, it is important to describe safeguards, opt-out procedures, frequency of complaints, and whether any participants discontinued ANC or disengaged because of distress. Also, the sentence “the observed effects did not impact the study outcomes” is not evidenced and should be removed or supported with data.

Response: We have expanded and clarified the harms section. We now specify how mild anxiety and confusion were assessed using structured endline questions and describe the monitoring process and opt-out mechanism. Frequencies of reported concerns are clearly stated. We have removed the unsupported statement that these effects did not influence study outcomes. Instead, we report that no serious adverse events occurred and that very few participants reported minor concerns, without making causal claims about their impact on service utilization.

These ancillary findings, as currently written, weaken the manuscript because they over-interpret descriptive trends and introduce implausible claims without rigorous measurement or arm-stratified analysis. You should either (i) re-analyze these ancillary outcomes by arm with appropriate cluster-adjusted models and report them as exploratory, or (ii) move them to supplementary materials with restrained interpretation, clearly distinguishing perceived barriers from objective service quality changes and avoiding unsupported causal mechanisms.

Discussion

1. The discussion appropriately summarizes the principal findings; however, it continues to overstate causal certainty in light of the methodological issues identified earlier. Statements such as “the intervention significantly improved utilization” and “both gain and loss-framed messages can significantly reduce maternal morbidity” are stronger than the design and data fully justify. Given the baseline imbalances in psychosocial variables, the high observed ICCs, the relatively small number of clusters, and the unexplained decline in the control arm, conclusions should be framed more cautiously. You should emphasize that the findings indicate an association within the context of this cluster-randomized design rather than definitive proof of large causal effects across settings.

Response: We agree that the original discussion overstated causal certainty relative to the methodological constraints of the trial. In the revised manuscript, we have replaced definitive language such as “the intervention significantly improved utilization” and “reduced maternal morbidity” with more cautious phrasing indicating that the intervention “was associated with higher utilization” and “was associated with lower self-reported maternal morbidity” within the context of this cluster-randomized design. We explicitly acknowledge the presence of baseline imbalances in psychosocial variables, the relatively small number of clusters per arm, the high observed intracluster correlation coefficients, and the decline observed in the control arm. We now emphasize that the findings should be interpreted as evidence of association within this specific implementation context rather than definitive proof of large causal effects generalizable across settings.

2. The explanation offered for the decline in the control arm introduces a substantial internal validity concern. The authors attribute this decline to baseline campaigns and community mobilization activities that were not sustained at endline. If true, this represents a time-varying contextual confounder affecting the baseline–endline comparison. In that case, the intervention effect may partly reflect the withdrawal of external support rather than solely the addition of messaging. This possibility should be acknowledged explicitly as a limitation rather than used to reinforce the intervention’s apparent superiority. Moreover, if community bylaws and enforcement against home delivery were active during the study period, the potential for contamination or differential exposure across clusters should be discussed.

Response :We agree that attributing the control arm decline to withdrawal of baseline community mobilization introduces potential internal validity concerns. In the revised discussion, we have reframed this explanation as a limitation rather than a reinforcing argument for intervention superiority. We explicitly acknowledge that time-varying contextual factors, including changes in district-level campaigns and community mobilization activities, may have influenced baseline–endline comparisons. We further discussed the possibility that the observed intervention effect may partly reflect the removal of concurrent external support rather than solely the additive effect of SMS messaging. Additionally, we now address the potential for contamination or differential exposure if community bylaws or enforcement practices were active during the study period, noting that such contextual dynamics may have affected clusters unevenly.

3. The interpretation of maternal morbidity reduction is particularly overstated. The manuscript attributes the reduction to increased ANC attendance and improved adherence, yet morbidity was self-reported and not clinically adjudicated. The magnitude of decline reported is large and biologically ambitious for an SMS-based behavioral intervention without a direct clinical component. Alternative explanations such as reporting bias, improved symptom interpretation, differential recall, or misclassification should be discussed. Without objective clinical verification, the claim that message framing “reduced maternal morbidity” is too strong and risks undermining credibility.

Response: We agree that the interpretation of maternal morbidity reduction required substantial moderation. In the revised manuscript, we clarify that maternal morbidity was self-reported and not clinically adjudicated, and therefore subject to recall bias, reporting bias, and potential misclassification. We have removed language implying direct clinical impact and replaced it with wording that specifies “reductions in reported maternal morbidity.” We also expand the discussion to consider alternative explanations, including improved symptom recognition, differential interpretation of symptoms, increased care-seeking leading to different labeling of complications, and social desirability bias. We acknowledge that the magnitude of change is ambitious for a behavioral SMS intervention without a direct clinical component and caution against strong causal interpretation in the absence of objective verification.

4. The comparative framing conclusions require more statistical restraint. The manuscript states that loss-framed messages were particularly effective for ANC and morbidity while gain-framed messages were more effective for SBA and PNC. However, the results section does not clearly present direct statistical comparisons between gain and loss arms. If head-to-head contrasts were not formally tested, these statements imply differences that may not be statistically supported. The discussion should clarify whether such comparisons were conducted and, if not, rephrase these conclusions to reflect descriptive trends rather than definitive superiority.

Response: We agree that the comparative framing conclusions required greater statistical restraint. In the revised discussion, we clarify that the primary analyses compared each intervention arm to the control arm and did not include formal head-to-head superiority testing between gain- and loss-framed groups unless explicitly specified. We therefore rephrase comparative statements to reflect observed descriptive patterns rather than definitive superiority. Instead of stating that one framing strategy was “more effective,” we now indicate that differential patterns were observed across outcomes and that these patterns warrant further investigation in adequately powered trials designed specifically for direct comparison

Implication

The implications section is forward-looking but somewhat overgeneralized. While the findings suggest promise for tailored mobile messaging, recommending integration into policy strategies should be tempered by acknowledgment of contextual specificity. The districts were purposively selected, mobile coverage was adequate, and concurrent community activities were in operation. External validity to more resource-constrained or less mobilized settings remains uncertain. Policy recommendations should be framed as conditional and suggest replication with larger numbers of clusters and intention-to-treat analyses as primary estimands.

Response: We agree that the implications section was overly generalized. In the revised manuscript, we temper policy recommendations and explicitly state that the districts were purposively selected, mobile network coverage was adequate, and concurrent community activities were ongoing. We acknowledge that external validity to more resource-constrained or less mobilized settings remains uncertain. Policy recommendations are now framed conditionally, suggesting that framed mobile messaging shows promise in similar contexts but requires replication in larger trials with more clusters and intention-to-treat analyses as primary estimands before broader scale-up can be justified.

Strengths

The strengths section appropriately highlights verification of outcomes and allocation concealment, but it does not fully address key methodological challenges. While adjustment for baseline covariates is mentioned, this does not entirely resolve baseline imbalance concerns in small-cluster trials. The reference to FDA and EMA guidance strengthens the rationale for covariate adjustment, yet the manuscript should clarify exactly which covariates were included and whether adjustment meaningfully altered effect sizes. Additionally, the high observed ICCs relative to the assumptions used in sample size planning are not discussed in the strengths or limitations, despite their implications for precision and generalizability.

Response: We have expanded the strengths section to provide a more balanced perspective. While we retain discussion of allocation concealment and outcome verification procedures, we now clarify exactly which baseline covariates were included in adjusted models and report whether covariate adjustment materially altered effect sizes. We also acknowledge that statistical adjustment cannot fully eliminate baseline imbalance concerns in small-cluster trials. Furthermore, we now explicitly address the discrepancy between assumed and observed ICC values and discuss its implications for effective sample size and estimate precision. This provides a more transparent appraisal of methodological robustness.

Limitations

The limitations section is currently underdeveloped relative to the complexity of the findings. Beyond recall bias and tool changes, the authors should explicitly acknowledge: the small number of clusters per arm; high intracluster correlation; baseline imbalances in psychosocial predictors; potential contamination from concurrent community activities; reliance on self-reported morbidity; inability to blind participants; and the risk of social desirability bias in reporting service utilization and barriers. Without a more transparent and comprehensive limitations discussion, the manuscript appears overly confident relative to its design constraints.

Response: We have substantially expanded the limitations section. In addition to recall bias and data collection tool differences, we now explicitly acknowledge the small number of clusters per arm, the high observed intracluster correlation, baseline imbalances in psychosocial predictors, possible contamination from concurrent community activities, reliance on self-reported maternal morbidity, inability to blind participants, and the potential for social desirability bias in reporting service utilization and perceived barriers. By articulating these constraints clearly, we aim to align the manuscript’s tone with the realities of the design and reduce the risk of overinterpretation.

In overall, the discussion would benefit from a stronger theoretical integration. While you cite literature on gain versus loss framing, they do not deeply engage with why framing effects were modestly differentiated in this context or why self-efficacy did not improve significantly despite changes in knowledge and attitudes. A more nuanced theoretical reflection considering ceiling effects, cultural context, stage of behavior change, and preventive versus detection behaviors would elevate the manuscript and align it more closely with the standards expected. The discussion section contains important insights and promising findings, but it requires substantial moderation of causal claims, clearer acknowledgment of design limitations, and more rigorous linkage between theory, methods, and interpretation to meet publication standards.

Theoretical Integration

Response: We agree that the discussion required deeper theoretical engagement. In the revised version, we more carefully integrate behavioral theory to explain why framing effects may have differed across maternal health behaviors. We discuss the distinction between preventive and detection behaviors, possible ceiling effects for self-efficacy, and the role of cultural norms and behavioral stage in moderating framing responsiveness. We also explore why knowledge and attitudes improved while self-efficacy did not change significantly, considering whether structural barriers or entrenched norms may limit translation of cognitive shifts into perceived control. This expanded theoretical reflection strengthens the scholarly contribution beyond programmatic findings.

Conclusion

1. The conclusion is currently too definitive relative to the methodological constraints of the study. Phrases such as “the study conclusively demonstrates” and “significantly enhance maternal health outcomes” overstate the strength of inference that can reasonably be drawn from a cluster trial with a small number of clusters, baseline imbalances, high observed ICCs, and reliance on self-reported morbidity. Conclusions should reflect statistical association within the study context rather than absolute proof. The wording should be moderated to state that the findings “suggest” or “indicate” effectiveness in this setting, rather than conclusively demonstrating it.

Response: We have revised the conclusion to remove definitive language such as “conclusively demonstrates” and “significantly enhance maternal health outcomes.” The conclusion now states that the findings “suggest” or “indicate” potential effectiveness within this specific cluster-randomized context. We explicitly reference the design constraints—including small cluster number, baseline imbalance, high ICCs, and reliance on self-reported morbidity—to frame the conclusions appropriately.

2. The comparative claims regarding gain versus loss framing also exceed the evidence presented. The conclusion states that loss-framed messages were “more effective” for certain outcomes and gain-framed messages for others. However, unless formal head-to-head statistical comparisons between the two intervention arms were conducted and reported, such superiority claims are not fully justified. If the analyses only compared each intervention arm to control, then differences between gain and loss should be described as descriptive patterns rather than statistically established differences.

Response: We have moderated comparative framing claims in the conclusion. Unless formal statistical head-to-head comparisons were conducted and reported, we avoid stating that one framing approach was more effective than the other. Differences between gain- and loss-framed arms are now described as observed patterns that require confirmation in trials specifically powered for direct comparison.

3. The statement that maternal morbidity was “reduced” by the intervention should be carefully reconsidered. Because morbidity was self-reported and not clinically adjudicated, and given the magnitude of change observed, the conclusion should refer to reductions in reported maternal morbidity rather than implying direct clinical impact. Without objective outcome validation, stronger clinical language may invite skepticism from reviewers and readers.

Response: We have revised the statement regarding maternal morbidity to refer explicitly to reductions in “self-reported maternal morbidity” rather than implying direct clinical reduction. This ensures consistency with the measurement approach and avoids overstating biological impact.

4. The conclusion would benefit from clearer acknowledgment of contextual boundaries. The study was conducted in purposively selected districts with concurrent community mobilization activities and adequate mobile network coverage. Therefore, generalization to other regions especially those with weaker infrastructure or different sociocultural dynamics should be framed cautiously. Rather than broad claims about “similar contexts,” it would be more appropriate to suggest that mobile phone-based framed messaging shows promise and warrants further evaluation in diverse settings with larger cluster samples.

Response: We now explicitly acknowledge contextual boundaries in the conclusion. The study setting, purposive district selection, mobile coverage conditions, and concurrent mobilization activities are referenced as factors limiting generalizability. We suggest that further evaluation in diverse and potentially less resourced settings is necessary before broader application.

5. Finally, the conclusion should more clearly integrate the theoretical contribution of the study. Instead of primarily emphasizing outcome improvements, it could briefly highlight that framing effects appear to operate differently across specific maternal health behaviors and psychosocial domains, reinforcing the importance of aligning message framing with behavioral stage and context. This would strengthen the scholarly contribution beyond programmatic implications.

Response: We have strengthened the theoretical contribution in the conclusion by briefly highlighting that framing effects may operate differently across maternal health behaviors and psychosocial domains. We emphasize the importance of aligning message framing with behavioral stage and context, thereby reinforcing the conceptual implications of the study rather than focusing solely on outcome magnitudes.

Finally, the conclusion should be revised to temper causal language, avoid overgeneralization, clarify the limits of comparative framing claims, and acknowledge reliance on self-reported outcomes. A more balanced and cautiously framed conclusion will enhance the credibility and impact of the manuscript.

Response : Overall, the revised discussion and conclusion now temper causal language, acknowledge methodological constraints transparently, avoid unsupported superiority claims, and integrate theoretical insight more rigorously. These revisions substantially strengthen the manuscript’s credibility and alignment with high-impact publication standards.

Verdict: Major Revision Needed

Attachment Submitted filename: 3.Review on PONE-D-25-10242_R3.docx

# Peer review: Decision Letter 3

**DOI:** 10.1371/journal.pone.0360474.r007

**Authors:** Werku Etafa

23 Jul 2026

Dear Dr.  Duguma,

Thank you for submitting your manuscript to PLOS ONE. After careful consideration, we feel that it has merit but does not fully meet PLOS ONE’s publication criteria as it currently stands. Therefore, we invite you to submit a revised version of the manuscript that addresses the points raised during the review process.

Please submit your revised manuscript by Sep 06 2026 11:59PM. If you will need more time than this to complete your revisions, please reply to this message or contact the journal office at plosone@plos.org. When you're ready to submit your revision, log on to https://www.editorialmanager.com/pone/ and select the 'Submissions Needing Revision' folder to locate your manuscript file.

- A letter that responds to each point raised by the academic editor and reviewer(s). You should upload this letter as a separate file labeled 'Response to Reviewers'.
- A marked-up copy of your manuscript that highlights changes made to the original version. You should upload this as a separate file labeled 'Revised Manuscript with Track Changes'.
- An unmarked version of your revised paper without tracked changes. You should upload this as a separate file labeled 'Manuscript'.

If you would like to make changes to your financial disclosure, please include your updated statement in your cover letter. Guidelines for resubmitting your figure files are available below the reviewer comments at the end of this letter.

If applicable, we recommend that you deposit your laboratory protocols in protocols.io to enhance the reproducibility of your results. Protocols.io assigns your protocol its own identifier (DOI) so that it can be cited independently in the future. For instructions see: https://journals.plos.org/plosone/s/submission-guidelines#loc-laboratory-protocols. Additionally, PLOS ONE offers an option for publishing peer-reviewed Lab Protocol articles, which describe protocols hosted on protocols.io. Read more information on sharing protocols at https://plos.org/protocols?utm_medium=editorial-email&utm_source=authorletters&utm_campaign=protocols.

As the corresponding author, your ORCID iD is verified in the submission system and will appear in the published article. PLOS supports the use of ORCID, and we encourage all coauthors to register for an ORCID iD and use it as well. Please encourage your coauthors to verify their ORCID iD within the submission system before final acceptance, as unverified ORCID iDs will not appear in the published article. *Only*  the individual author can complete the verification step; PLOS staff *cannot*  verify ORCID iDs on behalf of authors.

We look forward to receiving your revised manuscript.

Kind regards,

Werku Etafa

Academic Editor

PLOS One

Journal Requirements:

If the reviewer comments include a recommendation to cite specific previously published works, please review and evaluate these publications to determine whether they are relevant and should be cited. There is no requirement to cite these works unless the editor has indicated otherwise.

Additional Editor Comments:

The manuscript should adhere to the PLOS ONE manuscript format.

[Note: HTML markup is below. Please do not edit.]

Reviewers' comments:

Reviewer's Responses to Questions

**Comments to the Author**

Reviewer #1: All comments have been addressed

Reviewer #5: All comments have been addressed

Reviewer #6: (No Response)

**********

2. Is the manuscript technically sound, and do the data support the conclusions?

Reviewer #1: (No Response)

Reviewer #5: Yes

Reviewer #6: Yes

**********

3. Has the statistical analysis been performed appropriately and rigorously?

Reviewer #1: (No Response)

Reviewer #5: Yes

Reviewer #6: I Don't Know

**********

4. Have the authors made all data underlying the findings in their manuscript fully available?

The PLOS Data policy

Reviewer #1: (No Response)

Reviewer #5: Yes

Reviewer #6: No

**********

5. Is the manuscript presented in an intelligible fashion and written in standard English?

Reviewer #1: (No Response)

Reviewer #5: Yes

Reviewer #6: (No Response)

**********

Reviewer #1: (No Response)

Reviewer #5: This manuscript addresses an important public health topic by evaluating gain- and loss-framed SMS messages to improve maternal health service utilization in rural Ethiopia. The study has several strengths, including a cluster-randomized controlled trial design, prospective trial registration, an adequate sample size, and appropriate statistical analyses. The findings are relevant for digital health programs in low-resource settings.

However, several issues should be addressed before publication. The Introduction contains repeated information and should present the research gap more clearly. In the Methods section, more information is needed about intervention adherence, message delivery, and the per-protocol analysis. In the Results, some findings, such as the large decline in the control group and the large changes in perceived barriers, need further explanation. In the Discussion, the interpretation should be more cautious. Since maternal morbidity was self-reported, the manuscript should avoid stating that the intervention reduced maternal morbidity and instead describe it as being associated with lower self-reported maternal morbidity. In addition, because no direct statistical comparison was performed between the gain- and loss-framed groups, the manuscript should avoid suggesting that one message framing strategy was more effective than the other.

Finally, the manuscript would benefit from careful proofreading to improve grammar, consistency, and readability.

Reviewer #6: (No Response)

**********

what does this mean?). If published, this will include your full peer review and any attached files.

If you choose “no”, your identity will remain anonymous but your review may still be made public.

**Do you want your identity to be public for this peer review?**  For information about this choice, including consent withdrawal, please see our Privacy Policy

Reviewer #1: No

Reviewer #5: **Yes:**  Zafria Atsna

Reviewer #6: No

**********

[NOTE: If reviewer comments were submitted as an attachment file, they will be attached to this email and accessible via the submission site. Please log into your account, locate the manuscript record, and check for the action link "View Attachments". If this link does not appear, there are no attachment files.]

To ensure your figures meet our technical requirements, please review our figure guidelines: https://journals.plos.org/plosone/s/figures

You may also use PLOS’s free figure tool, NAAS, to help you prepare publication quality figures: https://journals.plos.org/plosone/s/figures#loc-tools-for-figure-preparation.

NAAS will assess whether your figures meet our technical requirements by comparing each figure against our figure specifications.

Attachment Submitted filename: review comment.docx

Attachment Submitted filename: pone.0005443.g002.tif

Attachment Submitted filename: 2. Revised_Manuscript_PLOS_ZA.docx

# Author comment: Author response to Decision Letter 4

**DOI:** 10.1371/journal.pone.0360474.r008

31 Aug 2026

Evaluating Gain and Loss-Framed Mobile Messaging to Improve Maternal Health Service Uptake: A Cluster Randomized Controlled Trial in Jimma, Ethiopia." (PONE-D-25-10242R3).

Dear Editor, Thank you so much for allowing me to review this important manuscript. Since the number of trial papers in our country (Ethiopia) is limited, I appreciate the authors for considering this interventional study to solve the maternal health service utilization problem.

Comments

Minor comment on the abstract

1. In the background of the abstract section, what the broad topic is and what your paper aims to explain are included; however, what is missing, unknown, or problematic is not included.

Suggestion: It will be informative if you include one sentence of the research gap in the background of the abstract.

Response : We appreciate this observation. We have revised the background of the abstract to explicitly state the research gap, noting that evidence on whether gain versus loss framed mobile messages differentially influence maternal health service uptake in rural Ethiopia remains limited.

2. In the conclusion part of the abstract, the use of the word "...was associated..." Indicate weakness in the RCT's finding.

Suggestion: Use active verbs because you applied a well-designed randomized control trial that rules out external factors through randomization; your conclusion should boldly reflect that causal link using active verbs. Instead of saying “were associated with increased maternal health service utilization,” use the phrase "lead to increased maternal health service utilization."

Response : Thank you for this important point. We have revised the conclusion to use active verbs, stating that gain and loss framed mobile messages led to increased maternal health service utilization, reflecting the causal inference supported by our randomized trial design.

Introduction

1. This interventional study was on mobile-based messages and how message framing affects maternal services usage; however, nothing was said about mobile phone usage in Ethiopia. How many of the rural women would have access to mobile phones? This data is important for further policy implementation. and for your

Suggestion: It would be better if you include the magnitude of mobile usage and any training in reading the message from literature.

Response : We agree with the reviewer. We have added recent national survey data on mobile phone penetration among rural women in Ethiopia, and noted literacy considerations relevant to message comprehension, to strengthen the policy relevance of our study.

2. The introduction is well-informed about the background data of maternal death, the cause of death, which is supported by evidence. The prospect theory also connected with the importance of the intervention and the research gap also discussed. However, lack of consistency in using references and a plausible argument for how the message framing influenced the maternal mortality were not yet included. Regarding inconsistency, what has been cited in the content of the manuscript and document (reference number 20) was repeated in the discussion. In addition, redundancy on page 4, paragraph 4, and paragraph 5, line numbers 6 to 0, was observed.

Suggestion: better if you include a plausible argument for inclusion of morbidity as outcome measurement in relation to your intervention. Check your references and better to clear the inconsistency. Delete the duplicate.

Response : Thank you for highlighting this. We have corrected the reference inconsistencies and removed duplicate citations. In addition, we have added a rationale for including maternal morbidity as an outcome, linking it to the potential of communication interventions to influence not only service uptake but also women’s self reported health experiences.

Method

On the methodological aspect, registration of the trial and publication of the trial prospectively are strengths. However, if there is any deviation from the publication, an amendment is a possible solution. For example, your primary outcome mentioned in the published protocol was maternal health service usage, which was defined as "maternal health service usage" when mothers receive at least 8 ANC follow-up visits, give birth in a health facility, and visit a health facility for PNC follow-up contact from health professionals within 7 days of delivery. However, you have changed in health service access by measuring receiving one antenatal care. This may be possible, but you have to amend this; instead, you have stated no change in outcome measurement after the protocol was

Published. Page 14, line number 367-369

Possible suggestion: include amendment of the protocol for any change after the publication of the protocol.

Response : We thank the reviewer for this important observation. While the trial was conducted as planned with no changes to eligibility criteria, intervention protocols, or data collection procedures, we acknowledge that an amendment was made to the primary outcome definition after protocol registration. Specifically, maternal health service usage was initially defined in the published protocol as receipt of at least eight ANC visits, institutional delivery, and PNC within 7 days. To reflect feasibility in rural Ethiopia, ANC utilization was redefined as at least one ANC visit (ANC1+). This amendment was formally approved by the ethics committee and documented in the trial records. We have now clarified this amendment in the Methods section to ensure transparency and consistency with the published protocol.

1. Describing the limitation in the method part better is avoided. Line number 176-178 on page 7 and page number 11 line number 293-297

Response : Thank you. We have removed limitations from the Methods section and relocated them to the Discussion, in line with reporting standards.

2. Redundancy on page 11, line number 303-305 and 306- 308

Response : Thank you. We have deleted the redundant sentences on page 11 (lines 303–308) to improve clarity.

Sample size determination

What was the suggested effect size before trial and have you considered this during interpretation of your finding?

Response : We appreciate this comment. We have now explicitly reported the assumed effect size (an absolute increase of 15 percentage points in ANC attendance, from 23.27% in the control arm to 38.27% in the intervention arms) that was used in the sample size calculation. In the Discussion, we have clarified how this prespecified effect size was considered when interpreting our findings, noting that the observed improvements in ANC, SBA, and PNC utilization met or exceeded the expected difference, thereby reinforcing the robustness of our conclusions.

Result

Comment 1. Comment 1. Minor editorial page 18 , line number 472

Response : Corrected as suggested.

Comment 2. Table number 2 line number 495 page 19

Response : Corrected as suggested.

Comment 3 : The objective of this table is to show if the study arm is balanced at the baseline; however, there should be either a p-value or more recommended mean/proportion differences. This makes the comparison easier for the reader.

Response : Thank you. We have revised Table 3 to include standardized mean differences for baseline characteristics, allowing clearer comparison of balance across study arms.

Comment 4. Too much information at a time makes confusion even, if it has connection better to report only the objective of the trial (primary and secondary outcomes) . I am not sure on the importance of Ancillary analysis.

Suggestion: better to avoid the result below this, otherwise include something about it in the introduction and method part. It makes the paper heavier and difficult to grasp the important finding.

Response : The ancillary analyses have been removed from the Results section. The section now focuses exclusively on primary and secondary outcomes, with only brief references in the Methods where necessary to justify analytic decisions.

Attachment Submitted filename: Response to reviewer comments.docx

# Peer review: Decision Letter 4

**DOI:** 10.1371/journal.pone.0360474.r009

**Authors:** Werku Etafa

21 Sep 2026

Evaluating Gain and Loss-Framed Mobile Messaging to Improve Maternal Health Service Uptake: A Cluster Randomized Controlled Trial in Jimma, Ethiopia.

PONE-D-25-10242R4

Dear Dr.Duguma,

We’re pleased to inform you that your manuscript has been judged scientifically suitable for publication and will be formally accepted for publication once it meets all outstanding technical requirements.

Within one week, you’ll receive an e-mail detailing the required amendments. When these have been addressed, you’ll receive a formal acceptance letter and your manuscript will be scheduled for publication.

An invoice will be generated when your article is formally accepted. Please note, if your institution has a publishing partnership with PLOS and your article meets the relevant criteria, all or part of your publication costs will be covered. Please make sure your user information is up-to-date by logging into Editorial Manager at Editorial Manager® and clicking the ‘Update My Information' link at the top of the page. For questions related to billing, please contact billing support.

If your institution or institutions have a press office, please notify them about your upcoming paper to help maximize its impact. If they’ll be preparing press materials, please inform our press team as soon as possible -- no later than 48 hours after receiving the formal acceptance. Your manuscript will remain under strict press embargo until 2 pm Eastern Time on the date of publication. For more information, please contact onepress@plos.org.

Kind regards,

Werku Etafa

Academic Editor

PLOS One

Additional Editor Comments (optional):

Reviewers' comments:

Reviewer's Responses to Questions

**Comments to the Author**

Reviewer #1: All comments have been addressed

**********

2. Is the manuscript technically sound, and do the data support the conclusions?

Reviewer #1: (No Response)

**********

3. Has the statistical analysis been performed appropriately and rigorously?

Reviewer #1: (No Response)

**********

4. Have the authors made all data underlying the findings in their manuscript fully available?

The PLOS Data policy

Reviewer #1: (No Response)

**********

5. Is the manuscript presented in an intelligible fashion and written in standard English?

Reviewer #1: (No Response)

**********

Reviewer #1: (No Response)

**********

what does this mean?). If published, this will include your full peer review and any attached files.

If you choose “no”, your identity will remain anonymous but your review may still be made public.

**Do you want your identity to be public for this peer review?**  For information about this choice, including consent withdrawal, please see our Privacy Policy

Reviewer #1: No

**********

# Editor report: Acceptance letter

**DOI:** 10.1371/journal.pone.0360474.r010

**Authors:** Werku Etafa

PONE-D-25-10242R4

PLOS One

Dear Dr. Duguma,

I'm pleased to inform you that your manuscript has been deemed suitable for publication in PLOS One. Congratulations! Your manuscript is now being handed over to our production team.

At this stage, our production department will prepare your paper for publication. This includes ensuring the following:

* All references, tables, and figures are properly cited

* All relevant supporting information is included in the manuscript submission,

* There are no issues that prevent the paper from being properly typeset

You will receive further instructions from the production team, including instructions on how to review your proof when it is ready. Please keep in mind that we are working through a large volume of accepted articles, so please give us a few days to review your paper and let you know the next and final steps.

Lastly, if your institution or institutions have a press office, please let them know about your upcoming paper now to help maximize its impact. If they'll be preparing press materials, please inform our press team within the next 48 hours. Your manuscript will remain under strict press embargo until 2 pm Eastern Time on the date of publication. For more information, please contact onepress@plos.org.

You will receive an invoice from PLOS for your publication fee after your manuscript has reached the completed accept phase. If you receive an email requesting payment before acceptance or for any other service, this may be a phishing scheme. Learn how to identify phishing emails and protect your accounts at https://explore.plos.org/phishing.

If we can help with anything else, please email us at customercare@plos.org.

Thank you for submitting your work to PLOS One and supporting open access.

Kind regards,

PLOS One Editorial Office Staff

on behalf of

Mr. Werku Etafa

Academic Editor

PLOS One
